# Mn-mediated sequential three-component domino Knoevenagel/cyclization/Michael addition/oxidative cyclization reaction towards annulated imidazo[1,2-*a*]pyridines

**DOI:** 10.3762/bjoc.14.287

**Published:** 2018-12-19

**Authors:** Olga A Storozhenko, Alexey A Festa, Delphine R Bella Ndoutoume, Alexander V Aksenov, Alexey V Varlamov, Leonid G Voskressensky

**Affiliations:** 1 Organic Chemistry Department, Peoples’ Friendship University of Russia (RUDN University), Miklukho-Maklaya st. 6, 117198 Moscow, Russian Federation; 2 Department of Chemistry, North Caucasus Federal University, Pushkin st. 1a, 355009 Stavropol, Russian Federation

**Keywords:** 2-aminochromene, domino reaction, imidazo[1,2-*a*]pyridine, 2-iminochromene, Michael addition, multicomponent reaction, oxidation, pyridine amination

## Abstract

The sequential three-component reaction between *o*-hydroxybenzaldehydes, *N*-(cyanomethyl)pyridinium salts and a nucleophile towards substituted chromenoimidazopyridines under oxidative conditions has been developed. The employment of Mn(OAc)_3_·2H_2_O or KMnO_4_ as stoichiometric oxidants allowed the use of a wide range of nucleophiles, such as nitromethane, (aza)indoles, pyrroles, phenols, pyrazole, indazole and diethyl malonate. The formation of the target compounds presumably proceeds through a domino Knoevenagel/cyclization/Michael addition/oxidative cyclization reaction sequence.

## Introduction

Domino reactions are well recognized for their ability to effectively synthesize organic compounds, as far as creating two and more chemical bonds in one-step decreases waste, resources and time, and makes the development of methodology of synthesis in a domino fashion a substantial task [1]. Recently, much attention in research was given to domino reactions with an oxidation step, revealing possibilities for shifting the equilibrium by making products more stable or in situ generating reactive intermediates [2–13]. In its turn, multicomponent reactions (MCRs), usually occurring as domino processes with three or more reactants mixed together, became a valuable tool for the synthetic chemistry to produce diverse and complex compounds in an efficient and sustainable way [14–17]. The use of oxidative conditions in MCRs was found to be useful [18], but challenging due to difficulty to match the redox potentials of three or more reactants at a time and employment of a sequential one-pot strategy may become one of the reasonable solutions.

**Figure 1:**
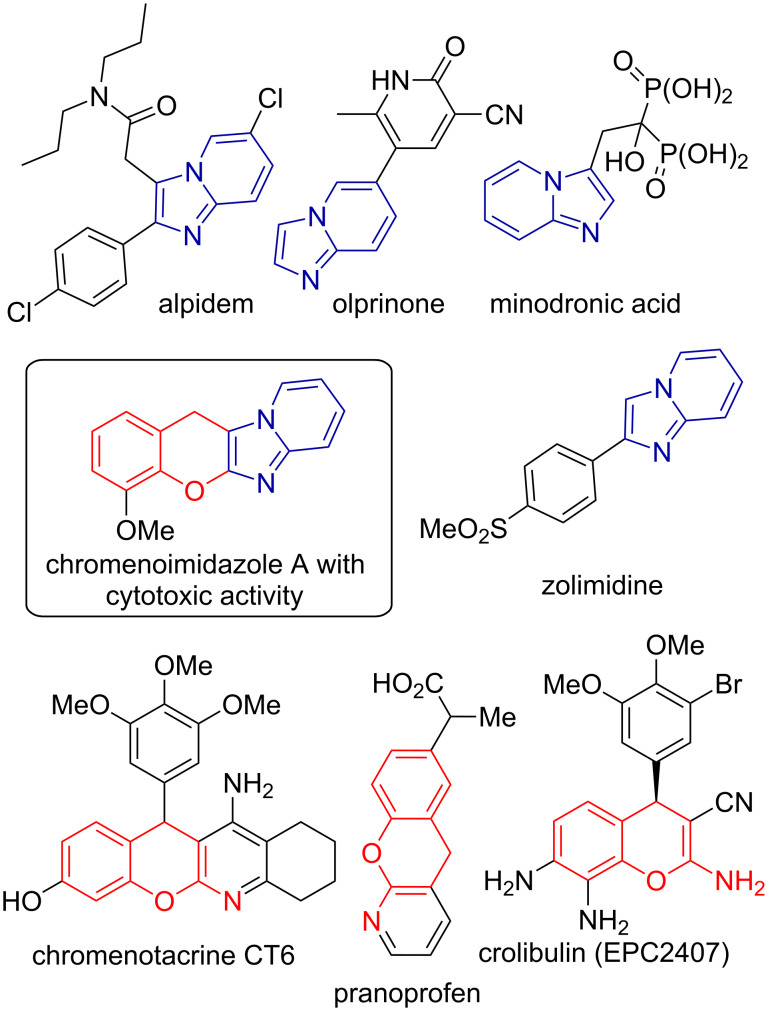
Biologically relevant imidazo[1,2-*a*]pyridines and chromenes.

The vast biological activity of the compounds, bearing the imidazo[1,2-*a*]pyridine scaffold makes this heterocycle of great importance to the fields of medicinal chemistry and biology [19–20], illustrated by the marketed drugs, e.g., alpidem, minodronic acid, olprinone, zolimidine (Figure 1) and some recent examples of the imidazopyridines inhibiting tubulin polymerization [21], NF-κB [22], aldosterone synthase [23], or autotaxin [24]. Whereas many interesting approaches towards the imidazo[1,2-*a*]pyridine core are being published nowadays [25–30], this molecular entity is still a pursued synthetic target and novel routes to diverse imidazopyridines are of value. Another privileged scaffold for drug discovery is 2-aminochromene, which may be found in crolibulin, an antitumor agent, undergoing phase II clinical trials [31], chromenotacrine CT-6, a potential anti-Alzheimer agent [32], and pranoprofen, a marketed anti-inflammatory drug [33]. Combination of chromene and imidazopyridine rings led to the discovery of compound **A** with promising anticancer activity [34], thereby showing the importance of this merged heterocyclic skeleton (Figure 1).

**Scheme 1:**
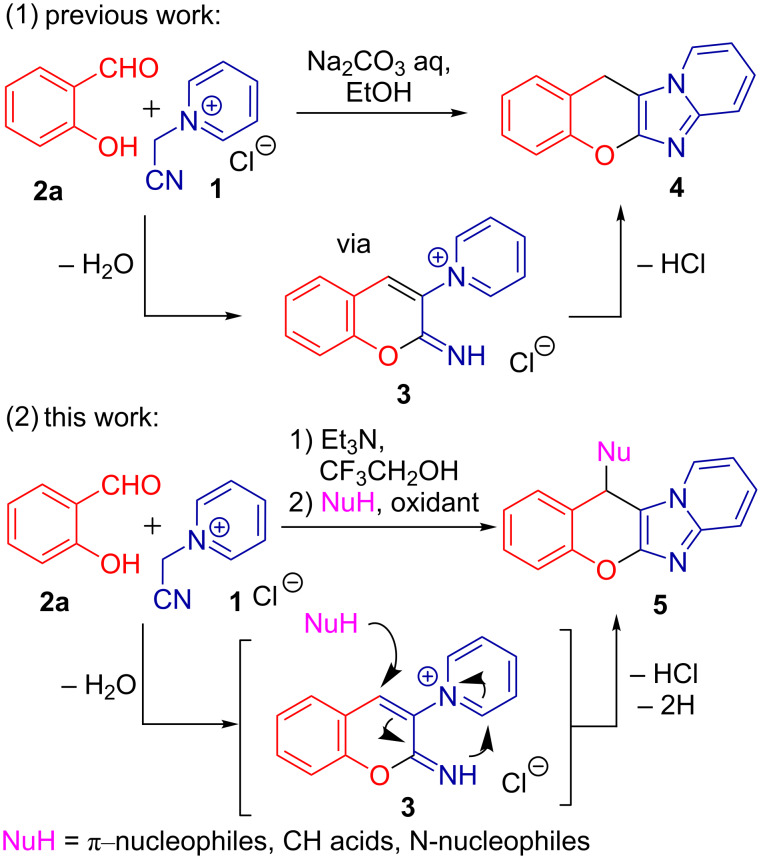
Domino formation of imidazopyridines and current work.

The formation of the chromene and imidazole rings in a single-step procedure was independently discovered by us [35–36] and Proença et al. [37–38], who identified 2-iminochromene **3** to be the key intermediate of the domino sequence (Scheme 1, reaction 1). Taking into account the capability of 2-iminochromenes to perform as Michael acceptors [39–41], we envisioned the diversification of the substitution pattern at the chromene ring to be a realizable and an appealing target, complicated by the need of an oxidant to fulfil the final aromatization. Following our interest in domino [42–43] and MCR chemistry [44–45] and taking an advantage of 2-iminochromene reactivity, herein we report a sequential three-component domino reaction of salicylaldehydes **2** and *N*-(cyanomethyl)pyridinium salts **1** with a broad scope of nucleophiles to produce diversely substituted valuable chromenoimidazopyridines under oxidative conditions (Scheme 1, reaction 2).

## Results and Discussion

To prove the designed concept, the reaction between salicylaldehyde (**2a**), *N*-(cyanomethyl)pyridinium chloride (**1**) and nitromethane as a nucleophile was carried out in ethanol with triethylamine as a base under air atmosphere in a two-step fashion. Firstly, the quaternary salt was stirred with salicylaldehyde in the presence of triethylamine at 0 °C for 30 min, and secondly nitromethane (10 equiv) was added and the mixture was refluxed for 2 h in an open vessel. As a result, the desired product **5a** was isolated in trace amounts as a mixture with compound **4** (Table 1, entry 1). Performing the first step under cooling was found to be essential to avoid cyclization of 2-iminochromene intermediate **3** into the two-component reaction product **4**. It is worth noting, that the conversion of **3** to **4** is a constant side reaction, occurring even at rt and complicating the process. Since the air oxygen was not enough to deliver the needed cyclization, we started to look for an appropriate oxidant. The addition of 1.1 equiv diacetoxyiodobenzene (PIDA) as an external oxidant on the second step and changing the solvent to trifluoroethanol allowed the isolation of the desired product **5a** with 25% yield after 2 h reflux (Table 1, entry 2), while leaving the reaction at rt for 7 days gave the compound **5a** with 30% yield (Table 1, entry 3). Further screening of the oxidants revealed, that the use of molecular iodine gave the desired product with 27% yield (Table 1, entry 4), while employment of NaOCl, NaIO_4_, MnO_2_, H_2_O_2_, or CuI/TBHP was not effective and led to the formation of complex mixtures (Table 1, entries 5–9), and use of CAN did not promote the reaction (Table 1, entry 10). The use of KMnO_4_ which is known as a classical oxidant for pyridine amination [46], gave desired chromenoimidazopyridine **5a** with admissible 47% yield (Table 1, entry 11). The yield of 54% was achieved with Mn(OAc)_3_·2H_2_O (Table 1, entry 12), while increasing the reaction time of the first step gave product **5a** with good 64% yield (Table 1, entry 13). The use of EtOH as a solvent was found inappropriate, as the yield was decreased by 21% (Table 1, entry 14), and reducing the amount of nitromethane lowered the yield by 5% (Table 1, entry 15). Use of DIPEA (Table 1, entry 16), DABCO (Table 1, entry 17) or K_2_CO_3_ (Table 1, entry 18) did not increase the yield of **5a**. Increasing the amount of Et_3_N to 3.8 equiv at the second step allowed to suppress the formation of byproduct **22** (Scheme 6, reaction 2, **IV**) and simplified the isolation of **5a** (Table 1, entry 19, further referred to as optimal conditions), probably due to improved solubility of manganese salt.

**Table 1:** Optimization of reaction conditions with nitromethane nucleophile.

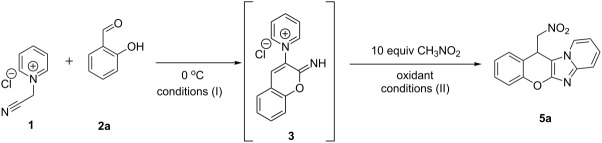				

entry	conditions (I)	oxidant (equiv)	conditions (II)	yield of **5a** (%)

1	Et_3_N (1 equiv), 0 °C, EtOH, 0.5 h	no oxidant	reflux, 2 h	traces
2	Et_3_N (1 equiv), 0 °C, TFE, 0.5 h	PIDA (1.1 equiv)	Et_3_N (2 equiv), reflux, 2 h	25
3	Et_3_N (1 equiv), 0 °C, TFE, 0.5 h	PIDA (1.1 equiv)	Et_3_N (2 equiv), rt, 7 days	30
4	Et_3_N (0.2 equiv), 0 °C, TFE, 1 h	I_2_ (1 equiv)	Et_3_N (0.8 equiv), reflux, 1 h	27
5	Et_3_N (1 equiv), 0 °C, TFE, 0.5 h	NaOCl (5% aq, 3 equiv)	Et_3_N (1 equiv), reflux, 1 h	complex mixture
6	Et_3_N (1 equiv), 0 °C, TFE, 0.5 h	NaIO_4_ (0.5 equiv)	Et_3_N (1 equiv), reflux, 1 h	complex mixture
7	Et_3_N (1 equiv), 0 °C, TFE, 0.5 h	MnO_2_ (2 equiv)	Et_3_N (1 equiv), reflux, 1 h	complex mixture
8	Et_3_N (1 equiv), 0 °C, TFE, 0.5 h	33% aq H_2_O_2_ (2 equiv)	Et_3_N (1 equiv), reflux, 1 h	complex mixture
9	Et_3_N (1 equiv), 0 °C, TFE, 0.5 h	CuI (0.1 equiv)/TBHP (2 equiv, 70% aq)	Et_3_N (1 equiv), reflux, 1 h	complex mixture
10	Et_3_N (1 equiv), 0 °C, TFE, 0.5 h	CAN (2 equiv)	Et_3_N (1 equiv), reflux, 1 h	–
11	Et_3_N (0.2 equiv), 0 °C, TFE, 1 h	KMnO_4_ (1 equiv)	Et_3_N (0.8 equiv), reflux, 1 h	47
12	Et_3_N (1 equiv), 0 °C, TFE, 0.5 h	Mn(OAc)_3_·2H_2_O (2 equiv)	Et_3_N (1 equiv), reflux, 1 h	54
13	Et_3_N (0.2 equiv), 0 °C, TFE, 1 h	Mn(OAc)_3_·2H_2_O (2 equiv)	Et_3_N (1.8 equiv), reflux, 1 h	64
14	Et_3_N (0.2 equiv), 0 °C, ETOH dry, 1 h	Mn(OAc)_3_·2H_2_O (2 equiv)	Et_3_N (1.8 equiv), reflux, 1 h	43
15^a^	Et_3_N (0.2 equiv), 0 °C, TFE, 1 h	Mn(OAc)_3_·2H_2_O (2 equiv)	Et_3_N (1.8 equiv), reflux, 1 h	59
16	DIPEA (0.2 equiv), 0 °C, TFE, 1 h	Mn(OAc)_3_·2H_2_O (2 equiv)	DIPEA (1.8 equiv), reflux, 1 h	55
17	DABCO (0.2 equiv), 0 °C, TFE, 1 h	Mn(OAc)_3_·2H_2_O (2 equiv)	DABCO (1.8 equiv), reflux, 1 h	42
18	K_2_CO_3_ (0.2 equiv), 0 °C, TFE, 1 h	Mn(OAc)_3_·2H_2_O (2 equiv)	K_2_CO_3_ (1.8 equiv), reflux, 1 h	36
19	Et_3_N (0.2 equiv), 0 °C, TFE, 1 h	Mn(OAc)_3_·2H_2_O (2 equiv)	Et_3_N (3.8 equiv), reflux, 1 h	64

^a^5 equiv CH_3_NO_2_ was used instead of 10 equiv.

**Scheme 2:**
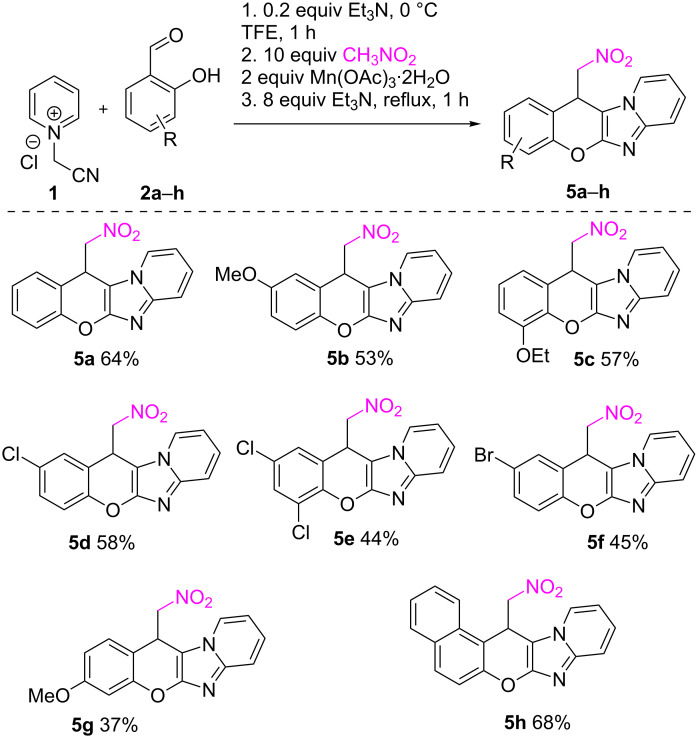
Scope of the reaction between *N*-(cyanomethyl)pyridinium chloride, *o*-hydroxybenzaldehydes, and nitromethane.

To understand the scope of this three-component reaction of nitromethane, the optimized conditions were used with different *o*-hydroxybenzaldehydes to prepare products **5a**–**h** with 37–68% yields, displaying tolerance to diverse substitution patterns in the aldehyde component (Scheme 2).

**Scheme 3:**
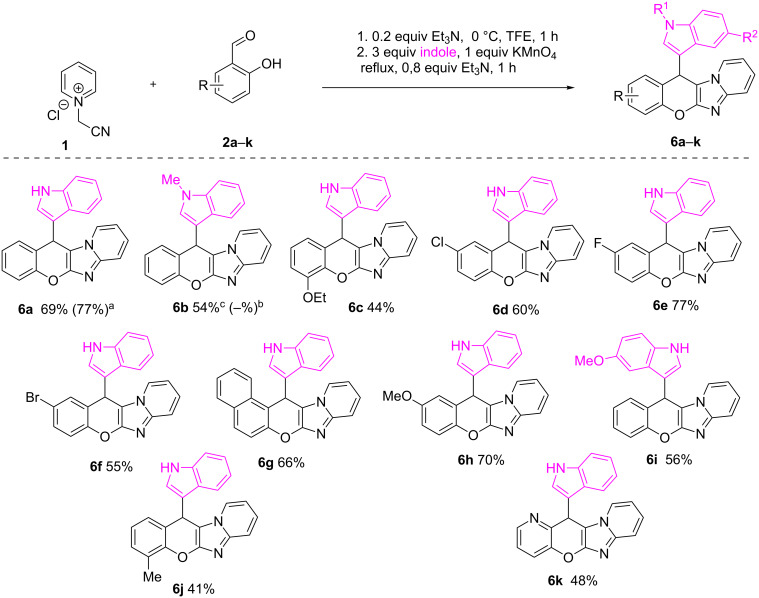
Scope of the reaction of *o*-hydroxybenzaldehydes with *N*-(cyanomethyl)pyridinium chloride and indoles. ^a^2 equiv Mn(OAc)_3_·2H_2_O was used as an oxidant at step 2; ^b^isolated as inseparable mixture with **4**; ^c^step 2 performed at 0 °C for 5 days.

According to the literature [47–52], the introduction of an indole core into a chromene moiety is an appealing task, which prompted us to investigate the possibility to use this nucleophile in the discovered process. The previously optimized conditions worked nicely for the reaction of *N*-(cyanomethyl)pyridinium chloride, *o*-hydroxybenzaldehyde and indole, producing the desired compound **6a** with good 77% yield (Scheme 3, footnote a). Unfortunately, the employment of *N*-methylindole resulted in the formation of the inseparable mixture of the target compound **6b** and the two-component reaction product **4** (Scheme 3, footnote b), showing the need for more general reaction conditions. It occurred that the use of KMnO_4_ as an oxidant was advantageous, though giving **6a** with a slightly lower yield of 69%, but furnishing *N*-methylindole product **6b** with 54% (Scheme 3, footnote c). Further investigation of the reaction scope gave rise to a series of diversely substituted chromenoimidazopyridines **6c**–**k**, demonstrating high synthetic potential of the transformation (Scheme 3).

**Scheme 4:**
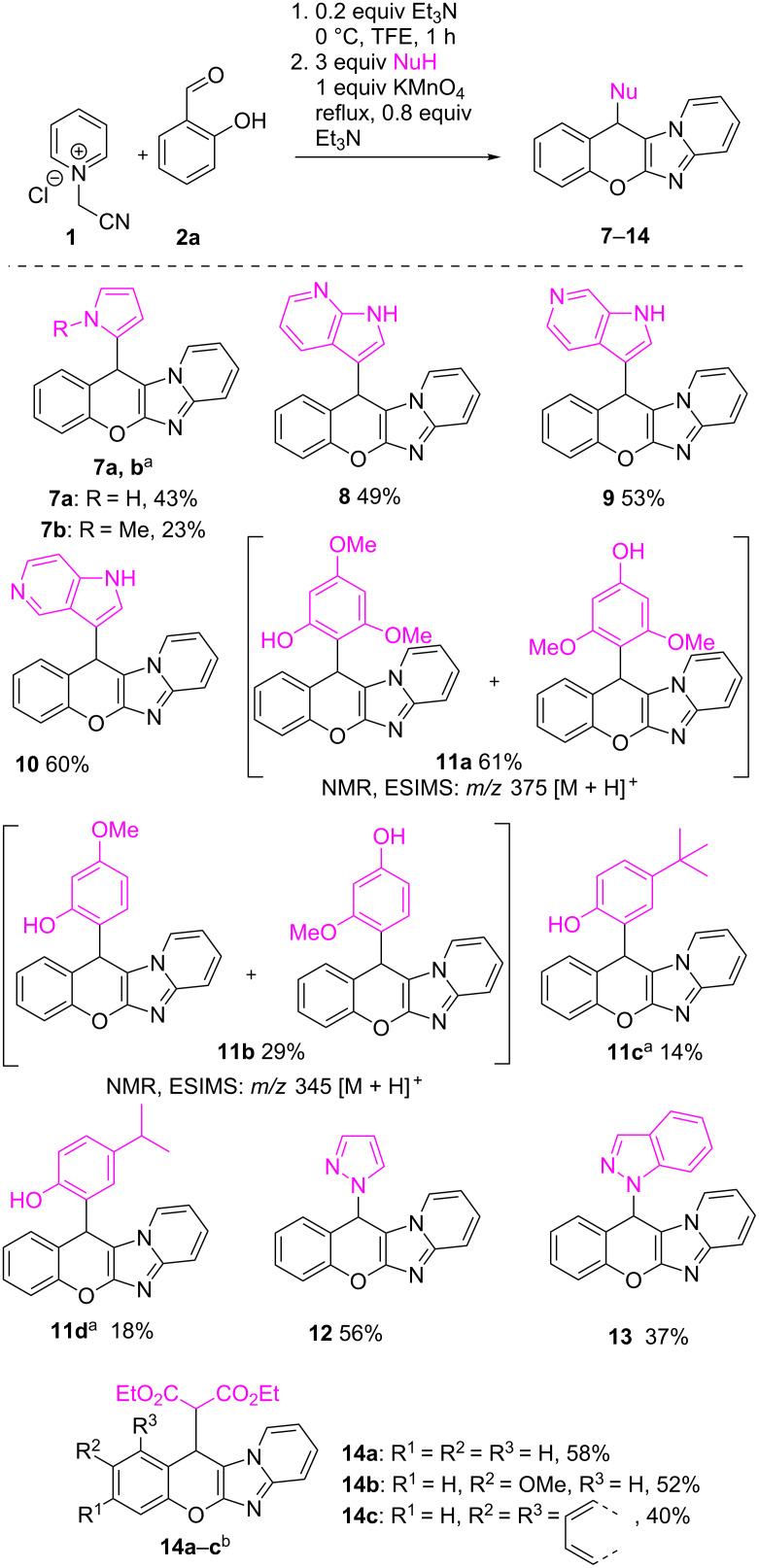
Scope of the nucleophiles in the reaction of *o*-hydroxyarylaldehydes with *N*-(cyanomethyl)pyridinium chloride and various nucleophiles. ^a^The second step was performed at 0 °C, for 5–8 days; ^b^1 equiv EtO_2_CCH_2_CO_2_Et was used at the first step at 0 °C for 2 days, after which KMnO_4_ was added and the reaction mixture was refluxed for 1 h.

To show the generality of the chosen oxidant, broad scope of nucleophiles was tested under selected conditions. Thus, employment of pyrrole as a nucleophile gave product **7a** in 43% yield, and *N*-methylpyrrole facilitated desired compound **7b** in 23% yield (Scheme 4). Isomeric 5-, 6- and 7-azaindoles were found to be appropriate nucleophiles too, producing the corresponding molecules **8**–**10**, in 60%, 53% and 49% yields, respectively.

Such well-known π-nucleophiles as phenols could be used for the reaction, though 3-methoxyphenol and 3,5-dimethoxyphenol gave inseparable mixtures of regioisomers **11a** and **11b** (NMR, LCMS). The reactions with *p*-isopropyl- and *p*-*tert*-butylphenols gave only one isomer, but the yields of the corresponding products **11c** and **11d** were low. Employed as N-nucleophiles, pyrazole and benzopyrazole successfully formed products **12** and **13**, correspondingly, with moderate yields. The possibility to employ CH-acids as nucleophiles was finally demonstrated on diethyl malonate, providing compounds **14a**–**c** in 40–58% yields.

**Scheme 5:**
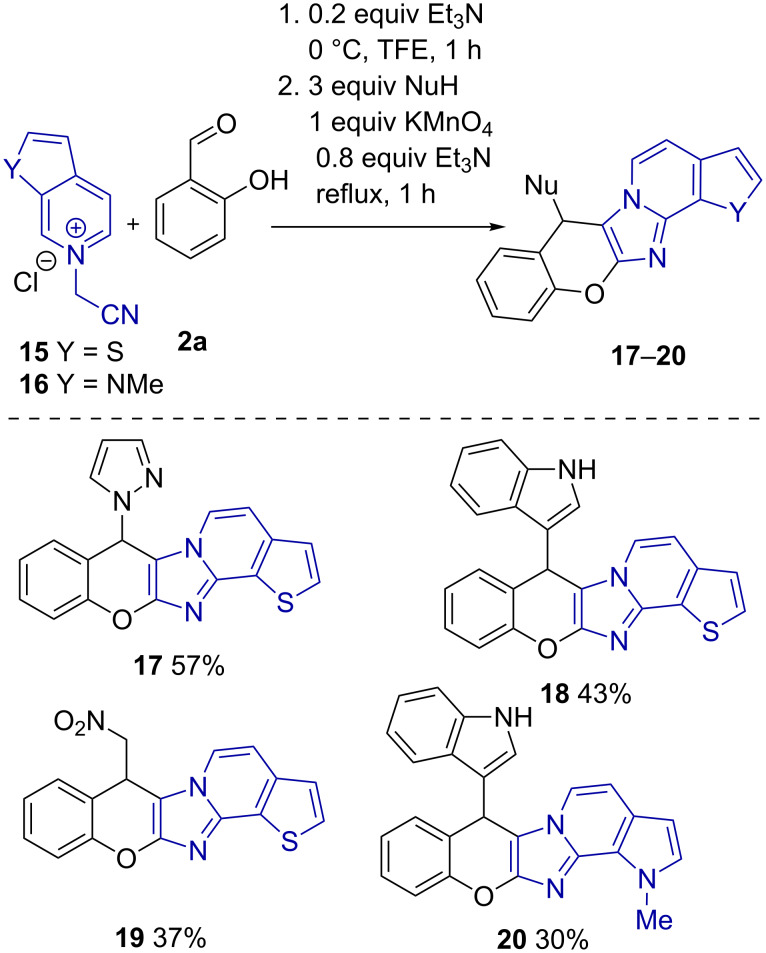
*N*-(Cyanomethyl)thieno[2,3-*c*]pyridinium chloride (**15**) and 6-(cyanomethyl)-1-methyl-1*H*-pyrrolo[2,3-*c*]pyridin-6-ium chloride (**16**) in reactions with salicylaldehyde and different nucleophiles. ^a^Second step was performed at 0 °C, for 7 days; ^b^10 equiv of CH_3_NO_2_ was used.

To conclusively reveal the scope of the reaction, we exploited *N*-cyanomethyl quaternary salts of fused thieno[2,3-*c*]pyridine **15** and 1-methyl-6-azaindole **16** in this transformation. Therefore, annulated chromenoimidazoles **17–20** were effectively produced in a sequential three-component manner (Scheme 5).

**Figure 2:**
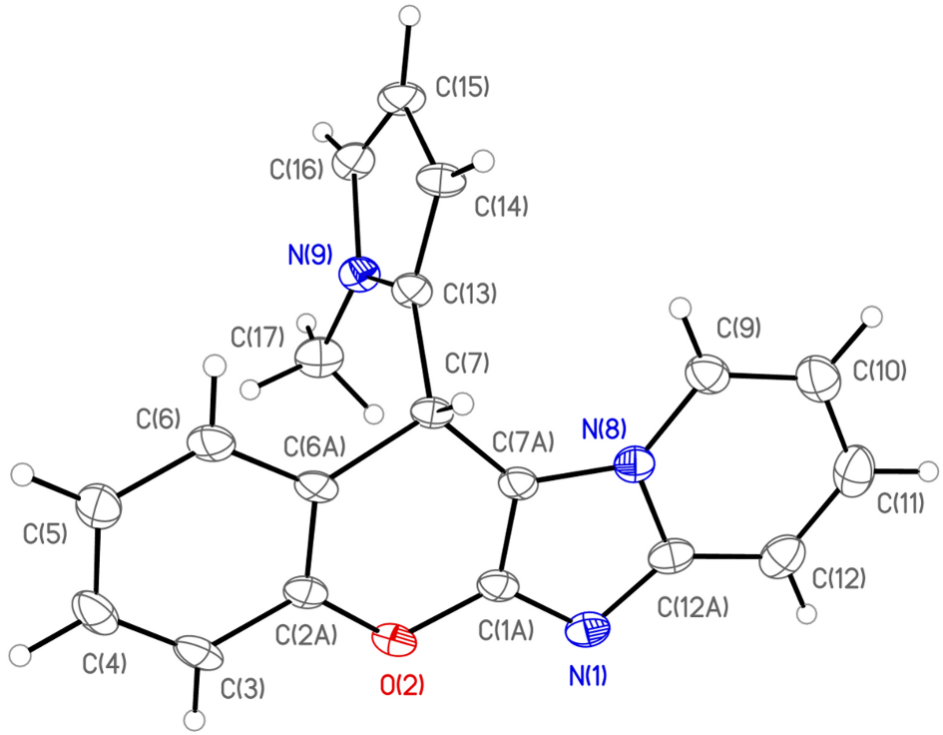
General view of the molecule **7b** in the crystal state (CCDC 1849215). Anisotropic displacement parameters are drawn at 50% probability.

The structures of the synthesized compounds **5a**–**h**, **6a**–**k**, **7**–**14**, **17**–**20** were confirmed by ^1^H, ^13^C NMR, IR spectroscopy and HRMS spectra (see Supporting Information File 1 for details). The structure of the compound **7b** was unambiguously determined by a single crystal X-ray diffraction study (Figure 2).

**Scheme 6:**
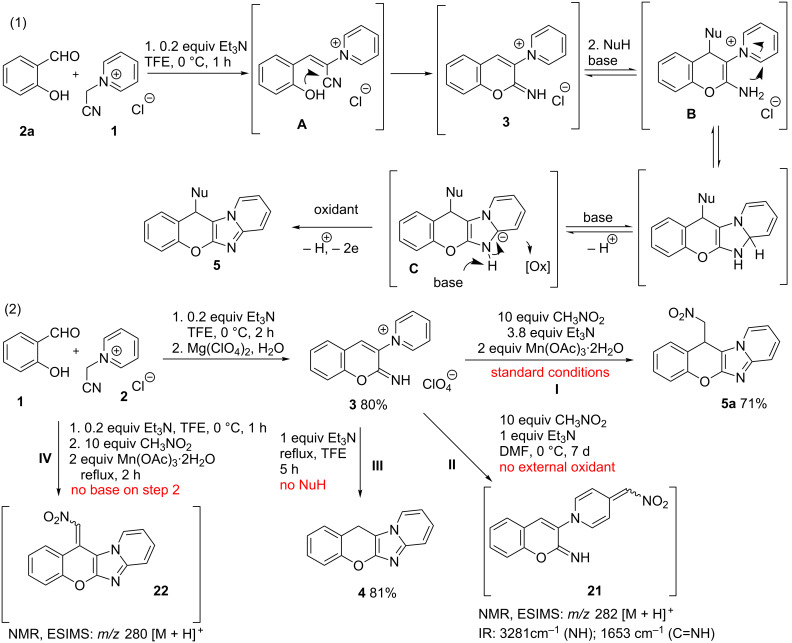
The presumed mechanism for the formation of target chromenoimidazopyridines (reaction 1) and additional experiments for mechanism elucidation (reaction 2).

The sequential domino reaction presumably starts with the Knoevenagel condensation of *o*-hydroxybenzaldehyde and *N*-(cyanomethyl)pyridinium salt forming styryl derivative **A**, which undergoes intramolecular cyclization to give 2-iminochromene salt **3**. Subsequent treatment of the reaction mixture with nucleophile, oxidant and a base leads to the Michael addition on C(4) of the chromene ring to produce 2-aminochromene **B** with incorporated nucleophilic moiety. Further cyclization and deprotonation furnishes anion **C**, which is easily oxidized to final product **5** (Scheme 6, reaction 1). The key 2-iminochromene intermediate **3** may be isolated as a perchlorate salt with 80% yield (Scheme 6, reaction 2). To confirm the reaction pathway, perchlorate **3** was converted into the product **5a** under the standard conditions with 71% yield (Scheme 6, reaction 2, **I**). Without an external oxidant, the reaction of perchlorate **3** with nitromethane fails to give the desired product, while compound **21**, arisen from nucleophilic attack on pyridinium moiety, was the only isolated material (Scheme 6, reaction 2, **II**). Without a nucleophile, the perchlorate **3** was confidently converted into the product **4** with 81% yield (Scheme 6, reaction 2, **III**). The importance of the base on a second step of the transformation and its involvement in the oxidation part was affirmed experimentally. Thus, when the reaction was performed under the standard conditions, but no triethylamine was added at the second step, target product **5a** was not observed, and compound **22** was isolated in trace amounts. Its formation may be explained by an initial nucleophilic addition of water to intermediate chromene **3**, oxidation to keto-derivative and condensation with nitromethane (Scheme 6, reaction 2, **IV**).

## Conclusion

In conclusion, we have developed a practical route towards substituted chromenoimidazopyridines through a sequential three-component domino Knoevenagel/cyclization/Michael addition/oxidative cyclization reaction, employing cheap and abundant oxidants. The discovered process works in a broad substrate scope with special emphasis to the tolerance to a wide range of nucleophiles, despite high proximity of the nucleophilic and reductive properties. We presume the transformation finds its place in the diversity-oriented synthesis toolbox to produce libraries of chromenoimidazoles with complex substitution and annulation patterns.

## Supporting Information

File 1 Experimental part, copies of NMR spectra and X-ray diffraction data.
