# Supplementary material for: Mn-mediated sequential three-component domino Knoevenagel/cyclization/Michael addition/oxidative cyclization reaction towards annulated imidazo[1,2-a]pyridines
Source: Beilstein J Org Chem. 2018 Dec 19;14:3078–87. doi: 10.3762/bjoc.14.287 (PMC6317425; doi:10.3762/bjoc.14.287)
Supplement: File 1 — Experimental part, copies of NMR spectra and X-ray diffraction data. [file Beilstein_J_Org_Chem-14-3078-s001.pdf]

BEILSTEIN JOURNAL OF ORGANIC CHEMISTRY

Supporting Information

for

Mn-mediated sequential three-component domino Knoevenagel/cyclization/Michael addition/oxidative cyclization reaction towards annulated imidazo[1,2-*a*]pyridines

Olga A. Storozhenko, Alexey A. Festa, Delphine R. Bella Ndoutoume, Alexander V. Aksenov, Alexey V. Varlamov and Leonid G. Voskressensky

Beilstein J. Org. Chem. **2018,** *14,* 3078–3087. doi:10.3762/bjoc.14.287

Experimental part, copies of NMR spectra and X-ray diffraction data

License and Terms: This is a supporting information file under the terms of the Creative Commons Attribution License (http://creativecommons.org/licenses/by/4.0). Please note that the reuse, redistribution and reproduction in particular requires that the authors and source are credited. The license is subject to the *Beilstein Journal of Organic Chemistry* terms and conditions: (https://www.beilstein-journals.org/bjoc)

Contents

Experimental S2
Copies of NMR spectra S20
Copies of 2D NMR spectra S63
X-ray diffraction study of compound 7b S82
References S90

Experimental

Starting reagents were purchased from commercial sources and were used without any additional purification. Salt **1** was prepared according to literature procedure [1]. Solvents were distilled and dried according to standard procedures. ^{1}H and ^{13}C NMR spectra were acquired on 400 or 600 MHz spectrometers and referenced to the residual signals of the solvent (for ^{1}H and ^{13}C). Chemical shifts are reported in parts per million (δ/ppm). Coupling constants are reported in Hertz (*J*/Hz). The peak patterns are indicated as follows: s, singlet; d, doublet; t, triplet; q, quadruplet; m, multiplet; dd, doublet of doublets and br s, broad singlet. Infrared spectra were measured on a FT/IR instrument. The wavelengths are reported in reciprocal centimeters (λ_{max} /cm- 1). High resolution mass spectra (HRMS) were obtained by electrospray ionisation using a Bruker MicrOTOF-Q II mass spectrometer and low resolution mass spectra were recorded with LCMS-8040 triple quadrupole liquid chromatograph mass-spectrometer from Shimadzu. The reaction progress was monitored by TLC and the spots were visualized under UV light (254 or 365 nm). Column chromatography was performed using silica gel (230-400 mesh) and mixtures in different proportions of ethyl acetate with hexane and methanol with dichloromethane as mobile phase. Melting points were determined on a SMP-10 apparatus.

1-(2-Imino-2*H*-chromen-3-yl)pyridin-1-ium perchlorate (3)

To a solution of salt **1** [1] (500 mg, 3.23 mmol, 1 equiv.) and salicylaldehyde (1.029 mL, 9.69 mmol, 3 equiv.) in 4 mL of TFE Et_3N (0.09 mL, 0.646 mmol, 0.2 equiv.) was added at 0°C. The mixture was stirred for 2 hours at ice bath, then the solution of $Mg(ClO_4)_2$ (720 mg, 3.23 mmol, 1 equiv.) in 5 mL of water was added at stirring. The precipitate was filtered off, washed with water (3x10mL), acetone (3x5mL) and methanol (5x3mL) then dried in air to give 828 mg (2.57 mmol, 80%) of iminochromene as white solid; mp 179 – 180°C. IR (KBr): 3274 (NH), 3126, 3076, 1666 (C=NH), 1631, 1602, 1474, 1216, 1093 cm^{-1}. Anal. Calcd for $C_{14}H_{11}ClN_2O_5$ (322.70): C, 52.11; H, 3.44; N, 8.68; Found: 52.01; H, 3.42; N, 8.77. ^{1}H NMR (DMSO-d_6, 600 MHz): δ = 9.27 (d, *J* = 5.4 Hz, 2H), 9.01 (s, 1H), 8.82 (t, *J* = 7.5 Hz, 1H), 8.34 (t, *J* = 6.7 Hz, 2H), 8.20 (s, 1H), 7.67 (d, *J* = 7.3 Hz, 1H), 7.64 (t, *J* = 7.8 Hz, 1H), 7.32 – 7.35 (m, 2H). ^{13}C NMR (DMSO-d_6, 150 MHz): δ

= 153.3, 151.4, 148.0, 146.1 (2C), 134.2, 133.2, 131.1, 129.8, 127.8 (2C), 124.6, 117.7, 115.5. HRMS (ESI/QTOF): m/z $[M]^+$ calcd for $C_{14}H_{11}N_2O$ 223.0865; Found 223.0872.

General procedure 1. Synthesis of nitromethylchromenoimidazopyridines 5a-h, 19.

To the solution of 1 mmol of **salt 1** in 2 mL of TFE and 1 mmol (1 equiv.) of aldehyde 0.2 mmol (28μL, 0.2 equiv.) of Et_3N was added at 0°C (ice bath). The reaction was stirred at 0°C for an hour. Then 10 mmol of nitromethane (536 μL, 10 equiv.), 3.8 mmol (529 μL, 3.8 equiv.) Et_3N and 2 mmol (536 mg, 2 equiv.) $Mn(OAc)_3x2H_2O$ were added, and the reaction mixture was rapidly heated to the reflux, and refluxed for 1 hour. White precipitate of $Mn(OAc)_2$ is formed in 10-15 min after beginning of reflux. Upon the completion, the reaction mixture was cooled to rt, extracted with DCM (3x20 mL), dried with Na_2SO_4 and concentrated in vacuo. Products were isolated by column chromatography on silica gel, eluent ethyl acetate-hexane 1-1.

12-(Nitromethyl)-12*H*-chromeno[2',3':4,5]imidazo[1,2-*a*]pyridine (5a)

Prepared according to the *general procedure 1* using **salt 1** (155 mg, 1 mmol, 1 equiv.), salicylaldehyde (106 μL, 1 mmol, 1 equiv.); the product was obtained as yellow solid (180 mg, 0.641 mmol, 64%); mp 176°C. IR (KBr): 3109 – 2877, 1648, 1603, 1569, 1542, 1503, 1484, 1468, 1431, 1377, 1275, 1215 – 1184, 754, 733 cm^{-1}. ^1H NMR (DMSO-d_6, 400 MHz): δ = 8.58 (d, *J* = 6.4 Hz, 1H, H-10), 7.64 (d, *J* = 7.5 Hz, 1H, H-1), 7.56 (d, *J* = 9.1 Hz 1H, H- 7), 7.35 – 7.41 (m, 2H, H-8, H-3), 7.23 – 7.27 (m, 2H, H-2, H-4), 7.07 (t, *J* = 6.7 Hz, 1H, H-9), 5.48 (s, 1H, H-12), 5.31 (dd, *J* = 4.0, 12.5 Hz, 1H $CH_{A\underline{B}}$), 5.15 (dd, *J* = 3.1, 12.5 Hz, 1H, $CH_{\underline{A}B}$). ^{13}C NMR (DMSO-d_6, 100 MHz): δ = 152.5 (C-5a), 151.5 (C-4a), 140.6 (C-6a), 129.6 (C-1), 129.1 (C-3), 124.9 (C-10), 124.8 (C-8), 123.9 (C-2), 118.9 (C-12a), 117.5 (C-7), 115.8 (C-4), 112.3 (C-9), 96.7 (C-11a), 77.9 (CH_2), 33.8 (CH). HRMS (ESI/QTOF): m/z $[M+H]^+$ calcd for $C_{15}H_{12}N_3O_3$: 282.0873; found: 282.0869.

2-Methoxy-12-(nitromethyl)-12*H*-chromeno[2',3':4,5]imidazo[1,2-*a*]pyridine (5b)

Prepared according to the *general procedure 1* using **salt 1** (155 mg, 1 mmol, 1 equiv.), 5-methoxy-2-hydroxybenzaldehyde (125 μL, 1 mmol, 1 equiv.); the product was obtained as yellow

solid (166 mg, 0.533 mmol, 53%); mp185°C. IR (KBr): 3096 – 2832, 1643, 1604, 1575, 1551, 1491, 1466, 1432, 1374, 1274, 1210 – 1196, 1147, 1046, 759, 728 cm^{-1}. ^1H NMR (DMSO-d_6, 600 MHz): δ = 8.57 (d, *J* = 6.7 Hz, 1H), 7.55 (d, *J* = 9.0 Hz, 1H), 7.35 (t, *J* = 7.9 Hz, 1H), 7.19 – 7.21 (m, 2H), 7.04 – 7.06 (m, 1H), 6.97 (dd, *J* = 2.9, 9.0 Hz, 1H), 5.42 (s, 1H), 5.32 (dd, *J* = 4.0, 12.5 Hz, 1H), 5.23 (dd, *J* = 3.3, 12.5 Hz, 1H), 3.80 (s, 3H). ^{13}C NMR (DMSO-d_6, 150 MHz): δ = 155.3, 152.9, 145.5, 140.7, 125.0, 124.8, 119.6, 118.3, 115.7, 115.1, 113.8, 112.3, 96.3, 77.6, 55.6, 34.1. HRMS (ESI/QTOF): m/z $[M+H]^+$ calcd for $C_{16}H_{14}N_3O_4$: 312.0978; found: 312.0982.

4-Ethoxy-12-(nitromethyl)-12*H*-chromeno[2',3':4,5]imidazo[1,2-*a*]pyridine (5c)

Prepared according to the *general procedure 1* using **salt 1** (155 mg, 1 mmol, 1 equiv.), 3-ethoxy-2-hydroxybenzaldehyde (166 mg, 1 mmol, 1 equiv.); the product was obtained as yellow solid (184 mg, 0.566 mmol, 57%); mp 157°C. IR (KBr): 3069 – 2842, 1644, 1607, 1572, 1549, 1467, 1434, 1378, 1272, 1207 – 1187, 1080, 762 cm^{-1}. ^1H NMR (DMSO-d_6, 600 MHz): δ = 8.57 (d, *J* = 6.5 Hz, 1H), 7.56 (d, *J* = 8.8 Hz, 1H), 7.35 – 7.37 (m, 1H), 7.12 – 7.17 (m, 2H), 7.05 – 7.08 (m, 2H), 5.46 (s, 1H), 5.28 (dd, *J* = 4.0, 12.5 Hz, 1H), 5.11 (dd, *J* = 3.1, 12.5 Hz, 1H), 4.11 (q, *J* = 6.7 Hz, 2H),1.41 (t, *J* = 6.7 Hz, 3H). ^{13}C NMR (DMSO-d_6, 150 MHz): δ = 152.6, 147.4, 141.4, 140.7, 125.0, 124.9, 123.7, 120.5, 119.6, 115.9, 112.5, 112.4, 96.8, 77.9, 64.1, 34.0, 14.7. HRMS (ESI/QTOF): m/z $[M+H]^+$ calcd for $C_{17}H_{16}N_3O_4$: 326.1135; found: 326.1140.

2-Chloro-12-(nitromethyl)-12*H*-chromeno[2',3':4,5]imidazo[1,2-*a*]pyridine (5d).

Prepared according to the *general procedure 1* using **salt 1** (155 mg, 1 mmol, 1 equiv.), 5-chloro-2-hydroxybenzaldehyde (157 mg, 1 mmol, 1 equiv.); the product was obtained as yellow solid (184 mg, 0.583 mmol, 58%); mp 211°C. IR (KBr): 3111 – 2838, 1645, 1605, 1566, 1552, 1466, 1431, 1375, 1342, 1221, 1116, 757 cm^{-1}. ^1H NMR (DMSO-d_6, 600 MHz): δ = 8.57 (d, *J* = 6.7 Hz, 1H), 7.78 (d, *J* = 2,3 Hz, 1H), 7.53 (d, *J* = 9.1 Hz, 1H), 7.40 (dd, *J* = 2.4, 8.7 Hz, 1H), 7.33 – 7.35 (m, 1H), 7.26 (d, *J* = 8.8 Hz, 1H), 7.04 (t, *J* = 6.7 Hz, 1H), 5.42 – 5.43 (m, 1H), 5.33 (dd, *J* = 4.0, 12.5 Hz, 1H), 5.21 (dd, *J* = 3.3, 12.5 Hz, 1H). ^{13}C NMR (DMSO-d_6, 150 MHz): δ = 152.4, 150.5,

140.8, 129.3, 129.1, 127.6, 125.2, 125.1, 121.2, 119.4, 115.9, 112.5, 96.3, 77.6, 33.8. HRMS (ESI/QTOF): m/z $[M+H]^+$ calcd for $C_{15}H_{11}ClN_3O_3$: 316.0483; found: 316.0491.

2,4-Dichloro-12-(nitromethyl)-12*H*-chromeno[2',3':4,5]imidazo[1,2-*a*]pyridine (5e)

Prepared according to the *general procedure 1* using **salt 1** (155 mg, 1 mmol, 1 equiv.), 3,5-dichloro-2-hydroxybenzaldehyde (191 mg, 1 mmol, 1 equiv.); the product was obtained as brown solid (153 mg, 0.437 mmol, 44%); mp 207°C. IR (KBr): 3115 – 2900, 1650, 1609, 1552, 1503, 1456 – 1435, 1380, 1239, 1174, 965, 860, 752, 732 cm^{-1}. ^1H NMR (DMSO-d_6, 600 MHz): δ = 8.63 (d, *J* = 6.7 Hz, 1H), 7.84 (d, *J* = 2.2 Hz, 1H), 7.74 (d, *J* = 2.2 Hz, 1H), 7.58 (d, *J* = 8.9 Hz, 1H), 7.38 – 7.41 (m, 1H), 7.10 (t, *J* = 6.7 Hz, 1H),), 5.50 – 5.51 (m, 1H), 5.40 (dd, *J* = 3.7, 13.1 Hz, 1H),5.27 (dd, *J* = 3.1, 13.1 Hz, 1H). ^{13}C NMR (DMSO-d_6, 150 MHz): δ = 151.9, 146.6, 140.8, 129.2, 128.4, 127.5, 125.5, 125.4, 123.0, 122.4, 118.1, 112.8, 96.4, 77.5, 34.1. HRMS (ESI/QTOF): m/z $[M+H]^+$ calcd for $C_{15}H_{10}Cl_2N_3O_3$ 350.0093; found: 350.0102.

2-Bromo-12-(nitromethyl)-12*H*-chromeno[2',3':4,5]imidazo[1,2-*a*]pyridine (5f)

Prepared according to the *general procedure 1* using **salt 1** (155 mg, 1 mmol, 1 equiv.), 5-bromo-2-hydroxybenzaldehyde (201 mg, 1 mmol, 1 equiv.); the product was obtained as yellow solid (161 mg, 0.447 mmol, 45%); mp 217°C. IR (KBr): 3075 – 2851, 1645, 1604, 1552, 1503, 1475, 1431, 1374, 1341, 1221, 757 cm^{-1}. ^1H NMR (DMSO-d_6, 600 MHz): δ = 8.61 (d, *J* = 6.6 Hz, 1H), 7.94 (d, *J* = 1.6 Hz, 1H), 7.55 – 7.59 (m, 2H), 7.36 – 7.39 (m, 1H), 7.24 (d, *J* = 8.7 Hz, 1H), 7.07 (t, *J* = 6.7 Hz, 1H), 5.46 – 5.48 (m, 1H), 5.36 (dd, *J* = 3.7, 12.8 Hz, 1H),5.25 (dd, *J* = 2.9, 12.8 Hz, 1H).

^{13}C NMR (DMSO-d_6, 150 MHz): δ = 152.3, 151.0, 140.8, 132.1, 132.0, 125.2, 125.1, 121.6, 119.7, 115.9, 115.4, 112.5, 96.3, 77.6, 33.7. HRMS (ESI/QTOF): m/z $[M+H]^+$ calcd for $C_{15}H_{11}BrN_3O_3$: 359.9978; found: 359.9980.

3-Methoxy-12-(nitromethyl)-12*H*-chromeno[2',3':4,5]imidazo[1,2-*a*]pyridine (5g)

Prepared according to the *general procedure 1* using **salt 1** (155 mg, 1 mmol, 1 equiv.), 4-methoxy-2-hydroxybenzaldehyde (152 mg, 1 mmol, 1 equiv.); the product was obtained as yellow

solid (114 mg, 0.367 mmol, 37%); mp 201°C**.** IR (KBr): 3095 – 2842, 1645, 1622, 1601, 1563, 1537, 1504, 1466, 1429, 1374, 1341, 1273, 1229, 1159, 1105, 1031, 979, 840, 817, 751, 736, 631 cm^{-1}.^{1}H NMR (DMSO-d_6, 600 MHz): δ = 8.57 (d, *J* = 6.7 Hz, 1H), 7.53 -7.57 (m, 2H), 7.35 – 7.38 (m, 1H), 7.06 (t, *J* = 6.7 Hz, 1H), 6.83 – 6.85 (m, 2H), 5.37 – 5.39 (m, 1H), 5.26 (dd, *J* = 4.1, 12.4 Hz, 1H), 5.23 (dd, *J* = 3.5, 12.4 Hz, 1H), 3.80 (s, 3H). ^{13}C NMR (DMSO-d_6, 150 MHz): δ = 159.7, 152.6, 152.5, 140.6, 130.3, 125.0, 124.8, 115.9, 112.4, 111.0, 110.7, 102.2, 97.1, 77.9, 55.5, 33.3. HRMS (ESI/QTOF): m/z $[M+H]^+$ calcd for $C_{16}H_{14}N_3O_4$: 312.0978; found: 312.0979.

14-(Nitromethyl)-14*H*-benzo[5',6']chromeno[2',3':4,5]imidazo[1,2-*a*]pyridine (5h)

Prepared according to the *general procedure 1* using **salt 1** (155 mg, 1 mmol, 1 equiv.), 2-hydroxy-1-naphthaldehyde (152 mg, 1 mmol, 1 equiv.); the product was obtained as brown solid (226 mg, 0.683 mmol, 68%); mp 200°C. IR (KBr): 3061 – 2851, 1648, 1575, 1545, 1501, 1469, 1435, 1382, 1229, 806, 753 cm^{-1}. ^{1}H NMR (DMSO-d_6, 600 MHz): δ = 8.67 (d, *J* = 6.6 Hz, 1H), 8.16 (t, *J* = 8.3 Hz, 1H), 8.04 (d, *J* = 8.1 Hz, 1H), 8.02 (d, *J* = 8.9 Hz, 1H), 7.75 (t, *J* = 7.6 Hz, 1H), 7.61 (d, *J* = 8.9 Hz, 1H), 7.58 (t, *J* = 7.4 Hz, 1H), 7.49 (d, *J* = 8.9 Hz, 1H), 7.39 – 7.42 (m, 1H), 7.16 (t, *J* = 6.7 Hz, 1H), 5.25 – 6.30 (m, 1H), 5.26 (dd, *J* = 3.4, 12.0 Hz, 1H), 5.06 (dd *J* = 3.2, 12.0 Hz, 1H). ^{13}C NMR (DMSO-d_6, 150 MHz): δ = 152.5, 150.4, 140.8, 130.7, 130.6, 130.2, 129.1, 127.6, 125.03, 124.99, 124.8, 122.4, 118.4, 116.0, 112.6, 111.2, 97.5, 76.9, 31.3. HRMS (ESI/QTOF): m/z $[M+H]^+$ calcd for $C_{19}H_{14}N_3O_3$: 332.1029; found: 332.1028.

General procedure 2. Synthesis of chromenoimidazopyridines 6b,7a,b, 11c,d, 12, 13, 17.

To the solution of 1 mmol of **salt** in 2 mL of TFE and 1 mmol (1 equiv.) of the aldehyde 0.2 mmol (28μL, 0.2 equiv.) of Et_3N was added at 0°C (ice bath). The reaction was stirred at 0°C for an hour. Then 3 mmol (3equiv.) of the **nucleophile**, 0.8 mmol (111μL, 0.8 equiv.) Et_3N and 1 mmol (158 mg, 1 equiv.) $KMnO_4$ were added, and the reaction mixture was stirred at 0°C for 1 hour, after that it was kept at 0°C for 5-8 days (control by TLC, alumina oxide, DCM-MeOH 1-10). Upon the

completion reaction mixture was concentrated in vacuo. Products were isolated by column chromatography on silica gel, eluent ethyl acetate-hexane in different proportions (1-5, 1-3, 1-1).

General procedure 3. Synthesis of chromenoimidazopyridines 6a, 6c-k, 8-10, 18, 20.

To the solution of 1 mmol of **salt** in 2 mL of TFE and 1 mmol (1 equiv.) of the aldehyde 0.2 mmol (28μL, 0.2 equiv.) of Et_3N was added at 0°C (ice bath). The reaction was stirred at 0°C for an hour. Then 3 mmol (3 equiv.) of the **nucleophile**, 0.8 mmol (111μL, 0.8 equiv.) Et_3N and 1 mmol (158 mg, 1 equiv.) $KMnO_4$ were added, and the reaction mixture was refluxed for 1 hour. Upon the completion reaction mixture was concentrated in vacuo. Products were isolated by column chromatography on silica gel, eluent DCM-MeOH (1-100) for compounds **6a, c-k,** DCM-MeOH (1-20) for **9**, (1-15) for **10**, ethyl acetate-hexane (1-1), ethyl acetate for compound **8**.

12-(1*H*-Indol-3-yl)-12*H*-chromeno[2',3':4,5]imidazo[1,2-*a*]pyridine (6a)

Prepared according to the *general procedure 3* using **salt 1** (155 mg, 1 mmol, 1 equiv.), salicylaldehyde (106 μL, 1 mmol, 1 equiv.), indole (351 mg, 3 mmol, 3 equiv.); the product was obtained as beige solid (234 mg, 0.694 mmol, 69%); mp 239°C. IR (KBr): 3413, 3202 - 2874, 1650, 1606, 1567, 1504, 1453, 1427, 1209, 761 – 730 cm^{-1}. ^1H NMR (DMSO-d_6, 600 MHz): δ = 11.08 (br s, 1H, NH, H-1'), 7.89 (d, *J* = 6.7 Hz, 1H, H-10), 7.70 (d, *J* = 2.3 Hz, 1H, H-2'), 7.50 (d, *J* = 8.9 Hz, 1H, H-7), 7.26 – 7.31 (m, 4H, H-1, H-3, H-4, H-7'), 7.19 (t, *J* = 8.1 Hz, 1H, H-8), 7.04 (t, *J* = 7.4 Hz, 1H, H-2), 6.93 (t, *J* = 7.5 Hz, 1H, H-6'), 6.83 (d, *J* = 8.1 Hz, 1H, H-4'), 6.79 (t, *J* = 6.8 Hz, 1H, H-9), 6.68 (t, *J* = 7.5 Hz, 1H, H-5'), 6.07 (s, 1H, CH, H-12). ^{13}C NMR (DMSO-d_6, 150 MHz): δ = 150.9 (C-5a), 150.5 (C-4a), 140.1 (C-6a), 136.9 (C-7a), 130.8 (C-1), 128.1 (C-3 or C-4), 125.2 (C-3a), 124.2 (C-2'), 124.0 (C-8), 123.9 (C-10), 123.8 (C-2), 123.6 (C-12a), 121.2 (C-6'), 118.7 (C-5'), 117.8 (C-4'), 117.3 (C-3 or C-4), 115.7 (C-7), 114.6 (C-3'), 112.0 (C-9), 111.8 (C-7'), 100.7 (C-11a), 33.7 (CH, C-12).

HRMS (ESI/QTOF): m/z $[M+Na]^+$ calcd for $C_{22}H_{15}N_3ONa$: 360.1107; found: 360.1118.

12-(1-Methyl-1*H*-indol-3-yl)-12*H*-chromeno[2',3':4,5]imidazo[1,2-*a*]pyridine (6b)

Prepared according to the *general procedure 2* using **salt 1** (155 mg, 1 mmol, 1 equiv.), salicylaldehyde (106 µL, 1 mmol, 1 equiv.), *N*-methylindole (374 µL, 3 mmol, 3 equiv.); the product was obtained as beige solid (190 mg, 0.541 mmol, 54%); mp 190 – 191°C. IR (KBr): 3106, 3056, 2959 - 2824, 1643, 1598, 1566, 1500, 1482, 1463, 1428, 761 – 742 cm^{-1}. ^{1}H NMR (DMSO-d_6, 600 MHz): δ = 7.88 (d, *J* = 6.5 Hz, 1H), 7.62 (c, 1H), 7.52 (d, *J* = 8.8 Hz, 1H), 7.25 – 7.34 (m, 4H), 7.19 (t, *J* = 7.7 Hz, 1H), 7.00 – 7.04 (m, 2H), 6.94 (d, *J* = 7.8 Hz, 1H), 6.80 (t, *J* = 6.6 Hz, 1H), 6.76 (t, *J* = 7.5 Hz, 1H), 6.08 (s, 1H), 3.76 (s, 3H). ^{13}C NMR (DMSO-d_6, 150 MHz): δ = 150.9, 150.3, 140.1, 137.1, 130.7, 128.3, 128.1, 125.7, 124.0, 123.9, 123.7, 123.6, 121.3, 118.9, 117.9, 117.3, 115.7, 113.9, 112.0, 110.0, 100.6, 32.4, 31.3. HRMS (ESI/QTOF): m/z $[M+H]^+$ calcd for $C_{23}H_{18}N_3O$: 352.1444; found: 352.1444.

4-Ethoxy-12-(1*H*-indol-3-yl)-12*H*-chromeno[2',3':4,5]imidazo[1,2-*a*]pyridine (6c)

Prepared according to the **general procedure 3** using **salt 1** (155 mg, 1 mmol, 1 equiv.), 3-ethoxy-2-hydroxybenzaldehyde (166 mg, 1 mmol, 1 equiv.) indole (351 mg, 3 mmol, 3 equiv.); the product was obtained as beige solid (166 mg, 0.436 mmol, 44%); mp 246°C. IR (KBr): 3412, 3140 – 2880, 1650, 1604, 1541, 1459, 1428, 1183, 1063, 742 cm^{-1}. ^{1}H NMR (DMSO-d_6, 600 MHz): δ = 11.07(br s, 1H), 7.87 (d, *J* = 6.5 Hz, 1H), 7.67(s, 1H), 7.50 (d, *J* = 9.0 Hz, 1H), 7.28 (d, *J* = 8.1 Hz, 1H), 7.18 (t, *J* = 7.8 Hz, 1H), 6.92 – 6.94 (m, 3H), 6.78 – 6.85 (m, 3H), 6.69 (t, *J* = 7.4 Hz, 1H), 6.05 (s, 1H), 4.14 (q, *J* = 2.9 Hz, 2H), 1.45 (t, *J* = 6.7 Hz, 3H). ^{13}C NMR (DMSO-d_6, 150 MHz): δ = 150.8, 147.3, 140.3, 140.0, 136.8, 125.2, 124.3, 124.1, 123.9, 123.86, 123.2, 121.7, 121.1, 118.6, 117.7, 115.6, 114.5, 111.9, 111.7, 111.4, 100.6, 64.1, 31.7, 14.8. HRMS (ESI/QTOF): m/z $[M+H]^+$ calcd for $C_{24}H_{20}N_3O_2$: 382.1550; found: 382.1554.

2-Chloro-12-(1*H*-indol-3-yl)-12*H*-chromeno[2',3':4,5]imidazo[1,2-*a*]pyridine (6d)

Prepared according to the **general procedure 3** using **salt 1** (155 mg, 1 mmol, 1 equiv.), 5-chloro-2-hydroxybenzaldehyde (157 mg, 1 mmol, 1 equiv.), indole (351 mg, 3 mmol, 3 equiv.); the product was obtained as beige solid (224 mg, 0.602 mmol, 60%); mp 246°C. IR (KBr): 3391 – 2931, 1648, 1605, 1566, 1474, 1424, 806, 729 cm^{-1}. ^{1}H NMR (DMSO-d_6, 600 MHz): δ = 11.16

(br s, 1H), 7.87 (d, *J* = 6.7 Hz, 1H), 7.74 (d, *J* = 2.2 Hz, 1H), 7.51 (d, *J* = 9.0 Hz, 1H), 7.37 (d, *J* = 8.8 Hz, 1H), 7.31 – 7.34 (m, 2H), 7.28 (d, *J* = 2.2 Hz, 1H), 7.20 (t, *J* = 7.9 Hz, 1H), 6.95 (t, *J* = 7.5 Hz, 1H), 6.79 – 6.82 (m, 2H), 6.71 (t, *J* = 7.5 Hz, 1H), 6.09 (s, 1H). ^{13}C NMR (DMSO-d$_6$, 150 MHz): δ = 150.7, 149.3, 140.1, 136.8, 129.9, 128.1, 127.2, 125.8, 125.0, 124.5, 124.2, 123.9, 121.3, 119.3, 118.9, 117.5, 115.7, 113.9, 112.1, 111.9, 100.1, 31.7. HRMS (ESI/QTOF): m/z [M+H]$^+$ calcd for $C_{22}H_{15}ClN_3O$: 372.0898; found: 372.0912.

2-Fluoro-12-(1*H*-indol-3-yl)-12*H*-chromeno[2',3':4,5]imidazo[1,2-*a*]pyridine (6e)

Prepared according to the **general procedure 3** using **salt 1** (155 mg, 1 mmol, 1 equiv.), 5-fluoro-2-hydroxybenzaldehyde (140 mg, 1 mmol, 1 equiv.), indole (351 mg, 3 mmol, 3 equiv.); the product was obtained as beige solid (273 mg, 0.769 mmol, 77%); mp 236°C. IR (KBr): 3411, 3142 – 2887, 1650, 1608, 1580, 1482 – 1427, 1357, 1258, 1183, 1132, 747 cm^{-1}. ^{1}H NMR (600 MHz, DMSO-d$_6$) δ 11.15 (br s, 1H), 7.88 (d, *J* = 6.7 Hz, 1H), 7.73 (d, *J* = 2.2 Hz, 1H), 7.51 (d, *J* = 8.9 Hz, 1H), 7.37 (dd, *J* = 4.8, 9.0 Hz, 1H), 7.31 (d, *J* = 8.1 Hz, 1H), 7.19 (t, *J* = 7.9 Hz, 1H), 7.14 (m, 1H), 7.05 (dd, *J* = 2.9, 9.2 Hz, 1H), 6.95 (t, *J* = 7.5 Hz, 1H), 6.79 – 6.82 (m, 2H), 6.70 (t, *J* = 7.5 Hz, 1H), 6.08 (s, 1H). ^{13}C NMR (150 MHz, DMSO-d$_6$) δ 157.1 (d, J_{CF} = 239.9 Hz), 150.9, 146.7, 140.1, 136.8, 125.47 (d, J_{CF} = 7.2 Hz), 125.0, 124.4, 124.1, 123.9, 121.22, 118.90 (d, J_{CF} = 7.2 Hz), 118.8, 117.5, 116.1 (d, J_{CF} = 23.1 Hz), 115.7, 115.2 (d, J_{CF} = 23.1 Hz), 113.7, 112.0, 111.9, 99.9, 32.0. HRMS (ESI/QTOF): m/z [M+H]$^+$ calcd for $C_{22}H_{15}FN_3O$: 356.1193; found: 356.1206**.**

2-Bromo-12-(1*H*-indol-3-yl)-12*H*-chromeno[2',3':4,5]imidazo[1,2-*a*]pyridine (6f)

Prepared according to the **general procedure 3** using **salt 1** (155 mg, 1 mmol, 1 equiv.), 5-bromo-2-hydroxybenzaldehyde (201 mg, 1 mmol, 1 equiv.), indole (351 mg, 3 mmol, 3 equiv.); the product was obtained as beige solid (230 mg, 0.553 mmol, 55%); mp 245°C. IR (KBr): 3217 – 2930, 1647, 1604, 1562, 1472, 1424, 1216, 1109, 1083, 773 – 728 cm^{-1}. ^{1}H NMR (DMSO-d$_6$, 600 MHz): δ = 11.15 (br s, 1H), 7.86 (d, *J* = 6.7 Hz, 1H), 7.74 (d, *J* = 2.2 Hz, 1H), 7.51 (d, *J* = 9.0 Hz, 1H), 7.44 (dd, *J* = 2.3, 8.8 Hz, 1H), 7.40 (d, *J* = 2.2 Hz, 1H), 7.30 – 7.32 (m, 2H), 7.20 (t, *J* = 7.9 Hz, 1H), 6.95 (t, *J* = 7.5 Hz, 1H), 6.79 – 6.82 (m, 2H), 6.71 (t, *J* = 7.5 Hz, 1H), 6.10 (s, 1H). ^{13}C

NMR (DMSO-d_6, 150 MHz): δ = 150.6, 149.8, 140.1, 136.8, 132.9, 130.9, 126.3, 125.0, 124.5, 124.2, 123.9, 121.3, 119.6, 118.9, 117.4, 115.7, 115.1, 113.9, 112.2, 111.9, 100.2, 31.6. HRMS (ESI/QTOF): m/z $[M+H]^+$ calcd for $C_{22}H_{15}BrN_3O$: 416.0393; found: 416.0396.

14-(1*H*-Indol-3-yl)-14*H*-benzo[5',6']chromeno[2',3':4,5]imidazo[1,2-*a*]pyridine (6g)

Prepared according to the **general procedure 3** using **salt 1** (155 mg, 1 mmol, 1 equiv.), 2-hydroxy-1-naphthaldehyde (152 mg, 1 mmol, 1 equiv.), indole (351 mg, 3 mmol, 3 equiv.); the product was obtained as beige solid (256 mg, 0.661 mmol, 66%); mp 251°C. IR (KBr): 3390 – 2924, 1649, 1591, 1573, 1502, 1425, 1339, 1227, 745 cm^{-1}. ^1H NMR (DMSO-d_6, 600 MHz): δ = 11.01 (br s, 1H), 8.40 (d, *J* = 8.4 Hz, 1H), 8.35 (d, *J* = 6.3 Hz, 1H), 7.94 – 7.97 (m, 2H), 7.89 (d, *J* = 7.8 Hz, 1H), 7.61 (d, *J* = 8.7 Hz, 1H), 7.47 – 7.52 (m, 2H), 7.39 (t, *J* = 7.1 Hz, 1H), 7.19 – 7.23 (m, 2H), 7.05 (d, *J* = 7.9 Hz, 1H), 6.93 (t, *J* = 6.5 Hz, 1H), 6.86 (t, *J* = 7.4 Hz, 1H), 6.81 (s, 1H), 6.69 (t, *J* = 7.4 Hz, 1H). ^{13}C NMR (DMSO-d_6, 150 MHz): δ = 150.2, 148.7, 140.0, 136.5, 131.8, 130.7, 129.4, 128.3, 126.5, 125.2, 124.4, 124.37, 124.0, 123.9, 123.8, 120.8, 118.6, 118.3, 117.6, 115.7, 115.1, 113.0, 112.0, 111.7, 102.1, 29.3. HRMS (ESI/QTOF): m/z $[M+H]^+$ calcd for $C_{26}H_{18}N_3O$: 388.1444; found: 388.1446.

12-(1*H*-Indol-3-yl)-2-methoxy-12*H*-chromeno[2',3':4,5]imidazo[1,2-*a*]pyridine (6h)

Prepared according to the **general procedure 3** using **salt 1** (155 mg, 1 mmol, 1 equiv.), 5-methoxy-2-hydroxybenzaldehyde (124 μL, 1 mmol, 1 equiv.), indole (351 mg, 3 mmol, 3 equiv.); the product was obtained as beige solid (255 mg, 0.695 mmol, 70%); mp 237°C. IR (KBr): 3404, 3139 – 2565, 1650, 1577, 1460, 1429, 1371 – 1346, 1277, 1196, 1143, 1043, 745 cm^{-1}. ^1H NMR (DMSO-d_6, 600 MHz): δ = 11.08 (br s, 1H), 7.88 (d, *J* = 6.7 Hz, 1H), 7.71 (d, *J* = 2.3 Hz, 1H), 7.49 (d, *J* = 9.0 Hz, 1H), 7.29 (d, *J* = 8.1 Hz, 1H), 7.26 (d, *J* = 9.0 Hz, 1H), 7.17 (t, *J* = 7.9 Hz, 1H), 6.93 (t, *J* = 7.6 Hz, 1H), 6.88 (dd, *J* = 3.0, 9.0 Hz, 1H), 6.83 (d, *J* = 8.0 Hz, 1H), 6.77 – 6.80 (m, 2H), 6.69 (t, *J* = 7.5 Hz, 1H), 6.02 (s, 1H), 3.62 (s, 3H). ^{13}C NMR (DMSO-d_6, 150 MHz): δ = 155.1, 151.2, 144.4, 140.0, 136.8, 125.2, 124.4, 124.2, 123.9, 123.8, 121.2, 118.7, 118.0, 117.7,

115.5, 115.0, 114.3, 113.6, 111.9, 111.8, 100.2, 55.3, 31.9. HRMS (ESI/QTOF): m/z $[M+H]^+$ calcd for $C_{23}H_{18}N_3O_2$: 368.1393; found: 368.1397.

12-(5-Methoxy-1*H*-indol-3-yl)-12*H*-chromeno[2',3':4,5]imidazo[1,2-*a*]pyridine (6i)

Prepared according to the **general procedure 3** using **salt 1** (155 mg, 1 mmol, 1 equiv.), salicylaldehyde (106 μL, 1 mmol, 1 equiv.), 5-methoxyindole (441 mg, 3 mmol, 3 equiv.); the product was obtained as beige solid (206 mg, 0.561 mmol, 56%); mp 205 – 207°C. IR (KBr): 3433, 3174 - 2832, 1649, 1606, 1570, 1502 - 1427, 1207, 747 – 732 cm^{-1}. ^1H NMR (DMSO-d_6, 600 MHz): δ = 10.93 (br s, 1H), 7.89 (d, *J* = 6.6 Hz, 1H), 7.62 (d, *J* = 2.2 Hz 1H), 7.52 (d, *J* = 9.0 Hz, 1H), 7.26 – 7.32 (m, 3H), 7.18 – 7.21 (m, 2H), 7.05 (t, *J* = 7.4 Hz, 1H), 6.80 (t, *J* = 6.8 Hz, 1H),3.48 (s, 3H), 6.61 (dd, *J* = 2.2, 8.7 Hz, 1H), 6.35 (d, *J* = 2.0 Hz, 1H), 6.04 (s, 1H). ^{13}C NMR (DMSO-d_6, 150 MHz): δ = 152.8, 151.0, 150.5, 140.1, 131.9, 130.8, 128.1, 125.7, 124.7, 124.0, 123.9, 123.7, 123.65, 117.1, 115.6, 114.3, 112.4, 112.0, 110.6, 100.6, 100.1, 54.9, 31.5. HRMS (ESI/QTOF): m/z $[M+H]^+$ calcd for $C_{23}H_{18}N_3O_2$ $(M + H)^+$: 368.1393; found: 368.1401.

12-(1*H*-Indol-3-yl)-4-methyl-12*H*-chromeno[2',3':4,5]imidazo[1,2-*a*]pyridine (6j)

Prepared according to the **general procedure 3** using **salt 1** (155 mg, 1 mmol, 1 equiv.), 3-methyl-2-hydroxybenzaldehyde (121 μL, 1 mmol, 1 equiv.), indole (351 mg, 3 mmol, 3 equiv.); the product was obtained as beige solid (144 mg, 0.410 mmol, 41%); mp 234°C. 3429, 3163 – 2859, 1648, 1606, 1576, 1421, 1177, 743 cm^{-1}.

^1H NMR (DMSO-d_6, 600 MHz): δ = 11.06 (br s, 1H), 7.87 (d, *J* = 6.7 Hz, 1H), 7.66 (d, *J* = 2.2 Hz, 1H), 7.49 (d, *J* = 8.9 Hz 1H), 7.28 (d, *J* = 8.1 Hz, 1H), 7.18 (t, *J* = 7.9 Hz, 1H), 7.10 – 7.14 (m, 2H), 6.91 – 6.94 (m, 2H), 6.84 (d, *J* = 8.1 Hz, 1H), 6.79 (t, *J* = 6.8 Hz, 1H), 6.69 (t, *J* = 7.6 Hz, 1H), 6.05 (s, 1H), 2.44 (s, 3H). ^{13}C NMR (DMSO-d_6, 150 MHz): δ = 150.9, 148.7, 140.0, 136.8, 129.2, 128.3, 125.7, 125.2, 124.1, 123.88, 123.86, 123.2, 123.0, 121.1, 118.7, 117.8, 115.6, 114.7, 111.9, 111.7, 100.7, 31.7, 16.2. HRMS (ESI/QTOF): m/z $[M+H]^+$ calcd for $C_{23}H_{18}N_3O$ $(M + H)^+$: 352.1444; found: 352.1440.

12-(1*H*-Indol-3-yl)-12*H*-pyrido[2”,1”:2’,3’]imidazo[4’,5’:5,6]pyrano[3,2-*b*]pyridine (6k)

Prepared according to the *general procedure 3* using **salt 1** (155 mg, 1 mmol, 1 equiv.), 3-hydroxypicolinaldehyde (123 mg, 1 mmol, 1 equiv.), indole (351 mg, 3 mmol, 3 equiv.); the product was obtained as gray solid (162 mg, 0.479 mmol, 48%); mp 242 – 245°C. IR (KBr): 3417, 3157 - 2875, 1649, 1605, 1560, 1455, 1429, 731 cm^{-1}. ^1H NMR (DMSO-d_6, 600 MHz): δ = 12.06 (br s, 1H), 8.27 (d, *J* = 4.1 Hz, 1H), 8.00 (d, *J* = 6.7 Hz, 1H), 7.77 (d, *J* = 8.2 Hz, 1H), 7.58 (d, *J* = 2.1 Hz 1H), 7.53 (d, *J* = 9.0 Hz, 1H), 7.33 (dd, *J* = 4.4, 8.2 Hz, 1H), 7.29 (d, *J* = 8.1, Hz, 1H), 7.21 (t, *J* = 7.8 Hz, 1H), 6.91 – 6.95 (m, 2H), 6.83 (t, *J* = 6.7, Hz, 1H), 6.72 (t, *J* = 7.5 Hz, 1H), 6.16 (s, 1H). ^{13}C NMR (DMSO-d_6, 150 MHz): δ = 150.2, 147.2, 144.9, 143.2, 140.4, 136.6, 125.5, 125.1, 124.5, 124.3, 124.2, 123.4, 121.0, 118.7, 117.7, 115.8, 113.5, 112.1, 111.7, 101.4, 35.1. HRMS (ESI/QTOF): m/z $[M+H]^+$ calcd for $C_{21}H_{15}N_4O$ $(M + H)^+$: 339.1240; found: 339.1241.

12-(1*H*-pyrrol-2-yl)-12*H*-chromeno[2',3':4,5]imidazo[1,2-*a*]pyridine (7a)

Prepared according to the **general procedure 2** using **salt 1** (155 mg, 1 mmol, 1 equiv.), salicylaldehyde (106 µL, 1 mmol, 1 equiv.), pyrrole (208 µL, 3 mmol, 3 equiv.); the product was obtained as white solid (123 mg, 0.428 mmol, 43%); mp 224°C. IR (KBr): 3405, 3174, 3116, 3080, 2990, 3851, 1649, 1606, 1569, 1501,1482, 1455, 1430, 1212, 828, 746 – 721 cm^{-1}. ^1H NMR (DMSO-d_6, 600 MHz): δ = 11.69 (br s, 1H), 7.74 (d, *J* = 6.6 Hz, 1H), 7.53 (d, *J* = 8.9 Hz, 1H), 7.29 – 7.32 (m, 2H), 7.24 - 7.25 (m, 2H), 7.11 (t, *J* = 7.4 Hz, 1H), 6.88 (t, *J* = 6.9 Hz, 1H), 6.58 (dd, *J* = 2.5, 4.5 Hz, 1H), 6.09 (s, 1H), 5.95 (dd, *J* = 2.5, 5.6 Hz, 1H), 5.86 (s, 1H). ^{13}C NMR (DMSO-d_6, 150 MHz): δ = 151.1, 150.2, 140.2, 131.2, 130.6, 128.2, 124.2, 123.9, 123.7, 122.6, 118.4, 117.5, 115.7, 112.1, 107.3, 106.8, 99.7, 33.1.HRMS (ESI/QTOF): m/z $[M+H]^+$ calcd for $C_{18}H_{14}N_3O$: 288.1131; found: 288.1118.

12-(1-Methyl-1*H*-pyrrol-2-yl)-12*H*-chromeno[2',3':4,5]imidazo[1,2-*a*]pyridine (7b)

Prepared according to the **general procedure 2** using **salt 1** (155 mg, 1 mmol, 1 equiv.), salicylaldehyde (106 µL, 1 mmol, 1 equiv.), (266 µL, 3 mmol, 3 equiv.); the product was obtained as white crystals (70 mg, 0.233 mmol, 23%); mp 195°C. IR (KBr): 3128 - 2805, 1645, 1602, 1566, 1502 - 1421, 1210, 751 - 721 cm^{-1}. ^1H NMR (DMSO-d_6, 600 MHz): δ = 7.77 (d, *J* = 6.7 Hz, 1H),

7.54 (d, *J* = 9.0 Hz, 1H), 7.34 (m, 1H), 7.26 – 7.30 (m, 2H), 7.13 – 7.18 (m, 2H), 6.90 (t, *J* = 6.7 Hz, 1H), 6.57 (m, 1H), 6.26 (c, 1H), 6.06 (s, 1H), 5.96 (t, *J* = 3.0 Hz, 1H), 3.01 (s, 3H). ^{13}C NMR (DMSO-d$_6$, 150 MHz): δ = 150.9, 150.4, 140.2, 130.2, 130.0, 128.6, 124.4, 124.0, 123.9, 123.7, 121.7, 117.4, 115.8, 112.3, 109.8, 106.4, 100.0, 33.4, 32.6. HRMS (ESI/QTOF): m/z $[M+H]^+$ calcd for $C_{19}H_{16}N_3O$: 302.1287; found: 302.1289.

12-(1*H*-Pyrrolo[2,3-*b*]pyridin-3-yl)-12*H*-chromeno[2',3':4,5]imidazo[1,2-*a*]pyridine (8)

Prepared according to the **general procedure 3** using **salt 1** (155 mg, 1 mmol, 1 equiv.), salicylaldehyde (106 µL, 1 mmol, 1 equiv.), 7-azaindole (354 mg, 3 mmol, 3 equiv.); the product was obtained as white solid (167 mg, 0.494 mmol, 49%); mp 251 – 254°C. IR (KBr): 3211 - 2573, 1643, 1599, 1566, 1420, 1210, 766 - 728 cm^{-1}. ^{1}H NMR (DMSO-d$_6$, 600 MHz): δ = 11.68 (br s, 1H), 8.05 (d, *J* = 4.5 Hz, 1H), 7.93 (d, *J* = 6.7 Hz, 1H), 7.87 (d, *J* = 2.2 Hz 1H), 7.51 (d, *J* = 8.9 Hz, 1H), 7.27 – 7.32 (m, 3H), 7.19 (t, *J* = 7.9, Hz, 1H), 7.12 (d, *J* = 7.9 Hz, 1H), 7.05 (t, *J* = 7.4, Hz, 1H), 6.81 (t, *J* = 6.7, Hz, 1H), 6.76 (dd, *J* = 4.6, 7.9 Hz, 1H), 6.07 (s, 1H). ^{13}C NMR (DMSO-d$_6$, 150 MHz): δ = 150.9, 150.3, 148.9, 142.7, 140.2, 130.7, 128.3, 125.8, 124.6, 124.2, 123.9, 123.8, 123.2, 117.44, 117.35, 115.7, 115.3, 113.6, 112.1, 100.3, 31.8. HRMS (ESI/QTOF): m/z $[M+H]^+$ calcd for $C_{21}H_{15}N_4O$: 339.1240; found: 339.1252.

12-(1*H*-Pyrrolo[2,3-*c*]pyridin-3-yl)-12*H*-chromeno[2',3':4,5]imidazo[1,2-*a*]pyridine (9)

Prepared according to the **general procedure 3** using **salt 1** (155 mg, 1 mmol, 1 equiv.), salicylaldehyde (106 µL, 1 mmol, 1 equiv.), 6-azaindole (354 mg, 3 mmol, 3 equiv.); the product was obtained as white solid (180 mg, 0.533 mmol, 53%); mp 251 – 253°C. IR (KBr): 3664, 3395, 3180 – 2619, 1649, 1608, 1569, 1502 – 1429, 747 – 731 cm^{-1}. ^{1}H NMR (DMSO-d$_6$, 600 MHz): δ = 11.61 (br s, 1H), 8.65 (s, 1H), 7.94 (d, *J* = 2.0 Hz, 1H), 7.89 (d, *J* = 6.7 Hz, 1H), 7.80 (d, *J* = 5.4 Hz 1H), 7.52 (d, *J* = 8.8 Hz, 1H), 7.27 – 7.33 (m, 3H), 7.20 (t, *J* = 7.9 Hz, 1H), 7.05 (t, *J* = 7.3, Hz, 1H), 6.81 (t, *J* = 6.8, Hz, 1H), 6.75 (d, *J* = 5.3 Hz, 1H), 6.13 (s, 1H). ^{13}C NMR (DMSO-d$_6$, 150 MHz): δ = 150.9, 150.3, 140.1, 137.6, 134.8, 133.9, 130.8, 129.4, 128.3, 128.0, 124.2, 123.88,

123.85, 123.2, 117.4, 115.7, 114.6, 112.3, 112.1, 100.3, 31.2. HRMS (ESI/QTOF): m/z $[M+H]^+$ calcd for $C_{21}H_{15}N_4O$: 339.1240; found: 339.1245.

12-(1*H*-Pyrrolo[3,2-*c*]pyridin-3-yl)-12*H*-chromeno[2',3':4,5]imidazo[1,2-*a*]pyridine (10)

Prepared according to the **general procedure 3** using **salt 1** (155 mg, 1 mmol, 1 equiv.), salicylaldehyde (106 μL, 1 mmol, 1 equiv.), 5-azaindole (354 mg, 3 mmol, 3 equiv.); the product was obtained as white solid (204 mg, 0.604 mmol, 60%). MP = 258 - 261°C. IR (KBr): 3200 - 2510, 1645, 1602, 1567, 1480 - 1427, 748 cm^{-1}. ^1H NMR (DMSO-d_6, 600 MHz): δ = 11.54 (br s, 1H), 8.08 (s, 1H), 8.00 (d, *J* = 5.5 Hz 1H), 7.94 (d, *J* = 8.9 Hz, 1H), 7.84 (s, 1H), 7.53 (d, *J* = 8.9 Hz, 1H), 7.28 – 7.35 (m, 4H), 7.20 (t, *J* = 7.9 Hz, 1H), 7.05 (t, *J* = 6.5, Hz, 1H), 6.81 (t, *J* = 6.7, Hz, 1H), 6.15 (s, 1H). ^{13}C NMR (DMSO-d_6, 150 MHz): δ = 150.8, 150.3, 140.8, 140.22 (2C), 140.17, 130.8, 128.3, 125.2, 124.2, 123.91, 123.87, 123.2, 122.3, 117.4, 115.7, 115.0, 112.1, 107.2, 100.5, 31.4. HRMS (ESI/QTOF): m/z $[M+H]^+$ calcd for $C_{21}H_{15}N_4O$: 339.1240; found: 339.1251.

4-(*tert*-Butyl)-2-(12*H*-chromeno[2',3':4,5]imidazo[1,2-*a*]pyridin-12-yl)phenol (11c)

Prepared according to the **general procedure 2** using **salt 1** (155 mg, 1 mmol, 1 equiv.), salicylaldehyde (106 μL, 1 mmol, 1 equiv.), 4-*tert*-butylphenol (450 mg, 3 mmol, 3 equiv.); the product was obtained as white solid (51 mg, 0.138 mmol, 14%); mp 218 – 220°C. IR (KBr): 3406 – 3000, 2959- 2813, 1648, 1607, 1569, 1502, 1462, 1431, 1374, 1271, 750 cm^{-1}. ^1H NMR (DMSO-d_6, 600 MHz): δ = 9.59 (s, 1H), 7.75 (d, *J* = 6.5 Hz, 1H), 7.52 (d, *J* = 8.7 Hz, 1H), 7.21 – 7.30 (m, 4H), 7.08 (t, *J* = 7.1 Hz, 1H), 7.05 (dd, *J* = 2.0, 8.4 Hz, 1H), 6.82 – 6.88 (m, 2H), 6.76 (d, *J* = 8.4 Hz, 1H), 6.76 (s, 1H), 1.04 (s, 9H). ^{13}C NMR (DMSO-d_6, 150 MHz): δ = 152.3, 151.5, 150.7, 141.5, 139.9, 130.4, 128.0, 127.1, 125.9, 124.9, 123.89, 123.86, 123.7, 123.6, 117.2, 115.7, 115.4, 112.1, 101.0, 34.1, 33.5, 31.2 (3C). HRMS (ESI/QTOF): m/z $[M+H]^+$ calcd for $C_{24}H_{23}N_2O_2$: 371.1754; found: 371.1761.

2-(12*H*-Chromeno[2',3':4,5]imidazo[1,2-*a*]pyridin-12-yl)-4-isopropylphenol (11d)

Prepared according to the **general procedure 2** using **salt 1** (155 mg, 1 mmol, 1 equiv.), salicylaldehyde (106 μL, 1 mmol, 1 equiv.), 4-isopropylphenol (408 mg, 3 mmol, 3 equiv.); the

product was obtained as white solid (65 mg, 0.183 mmol, 18%); mp 218 – 220°C. IR (KBr): 3455 – 2467, 1648, 1606, 1568, 1499, 1461, 1430, 1371, 1277, 747 cm^{-1}. ^{1}H NMR (DMSO-d_6, 600 MHz): δ = 9.62 (s, 1H), 7.75 (d, *J* = 6.7 Hz, 1H), 7.52 (d, *J* = 8.9 Hz, 1H), 7.22 – 7.30 (m, 4H), 7.08 (t, *J* = 7.3 Hz, 1H), 7.91 (dd, *J* = 2.1, 8.3 Hz, 1H), 6.87 (t, *J* = 6.8 Hz, 1H), 6.79 (d, *J* = 8.3 Hz, 1H), 6.07 (s, 1H), 6.65 (s, 1H), 2.56 – 2.61 (m, 1H), 0.94 (dd, *J* = 2.3, 6.7 Hz, 6H). ^{13}C NMR (DMSO-d_6, 150 MHz): δ = 152.7, 151.4, 150.7, 139.9, 139.2, 130.5, 128.0, 127.6, 127.1, 125.6, 123.9 (2C), 123.8, 123.6, 117.2, 115.8, 115.7, 112.1, 101.1, 33.4, 32.3, 24.0, 23.9. HRMS (ESI/QTOF): m/z $[M+H]^+$ calcd for $C_{23}H_{21}N_2O_2$: 357.1597; found: 357.1609.

12-(1*H*-Pyrazol-1-yl)-12*H*-chromeno[2',3':4,5]imidazo[1,2-*a*]pyridine (12)

Prepared according to the **general procedure 2** using **salt 1** (155 mg, 1 mmol, 1 equiv.), salicylaldehyde (106 µL, 1 mmol, 1 equiv.), pyrazole (204 mg, 3 mmol, 3 equiv.); the product was obtained as white solid (162 mg, 0.563 mmol, 56%), mp 209°C. IR (KBr): 3131 – 3034, 2946, 1644, 1609, 1570, 1503 – 1427, 772 – 736 cm^{-1}.

^{1}H NMR (DMSO-d_6, 600 MHz): δ = 7.91 – 7.93 (m, 2H), 7.62 (d, *J* = 8.9 Hz, 1H), 7.45 – 7.47 (m, 2H), 7.41 – 7.42 (m, 3H), 7.31 (t, *J* = 7.9, Hz, 1H), 6.98 (t, *J* = 6.8, Hz, 1H), 6.27 (t, *J* = 1.9 Hz, 1H). ^{13}C NMR (DMSO-d_6, 150 MHz): δ = 152.4, 150.4, 141.1, 139.6, 129.9, 129.8, 129.3, 125.9, 124.5, 124.1, 119.5, 117.8, 116.0, 112.7, 105.9, 98.0, 55.1. HRMS (ESI/QTOF): m/z $[M+H]^+$ calcd for $C_{17}H_{13}N_4O$: 289.1084; found: 289.1089.

12-(1*H*-Indazol-1-yl)-12*H*-chromeno[2',3':4,5]imidazo[1,2-*a*]pyridine (13)

Prepared according to the **general procedure 2** using **salt 1** (155 mg, 1 mmol, 1 equiv.), salicylaldehyde (106 µL, 1 mmol, 1 equiv.), indazole (354 mg, 3 mmol, 3 equiv.); the product was obtained as beige solid (125 mg, 0.370 mmol, 37%); mp 180°C. IR (KBr): 3100 – 3032, 2958 – 2833, 1640, 1603, 1567, 1483 – 1431, 750 cm^{-1}. ^{1}H NMR (DMSO-d_6, 600 MHz): δ = 8.08 (s, 1H), 7.90 (s, 1H), 7.72 (d, *J* = 8.1 Hz, 1H), 7.66 (d, *J* = 6.7 Hz, 1H), 7.57 (d, *J* = 9.1 Hz, 1H), 7.37 – 7.44 (m, 3H), 7.24 – 7.29 (m, 3H), 7.07 – 7.10 (m, 2H), 6.85 (t, *J* = 6.8 Hz, 1H). ^{13}C NMR (DMSO-d_6, 150 MHz): δ = 152.6, 150.6, 141.1, 138.7, 134.1, 130.0, 129.4, 126.8, 125.8, 124.4, 124.3,

124.2, 121.4, 121.1, 119.4, 117.8, 116.1, 112.9, 108.9, 97.8, 51.8. HRMS (ESI/QTOF): m/z $[M+H]^+$ calcd for $C_{21}H_{15}N_4O$: 339.1240; found: 339.1254.

General procedure 4. Synthesis of chromenoimidazopyridines 14a-c.

To the solution of 1 mmol of **salt 1** (155 mg, 1 equiv.) in 2 mL of TFE and 1 mmol (1 equiv.) of the aldehyde 0.2 mmol (28μL, 0.2 equiv.) of Et_3N was added at 0°C (ice bath). The reaction was stirred at 0°C for an hour. Then 1 mmol of the diethylmalonate (152 μL, 1 equiv.), 0.8 mmol (111μL, 0.8 equiv.) Et_3N were added and reaction mixture was cooled to 0°C for 2 days, after that 1 mmol (158 mg, 1 equiv.) $KMnO_4$ was added, and the reaction was refluxed for 1 hour. Upon the completion reaction mixture was cooled to rt, concentrated in vacuo. Products were isolated by column chromatography on silica gel (eluent ethyl acetate-hexane in different proportions: 1-5, 1-3, 1-1).

Diethyl 2-(12*H*-chromeno[2',3':4,5]imidazo[1,2-*a*]pyridin-12-yl)malonate (14*a*)

Prepared according to the *general procedure 4* using **salt 1** (155 mg, 1 mmol, 1 equiv.), salicylaldehyde (106 μL, 1 mmol, 1 equiv.), diethylmaonate (152 μL, 1 mmol, 1 equiv.); the product was obtained as colorless oil (222 mg, 0.584 mmol, 58%). R_f 0.24 (hexane / EtOAc = 1:1). IR (KBr): 2980 – 2932, 1730, 1645, 1601, 1505, 1462, 1429, 1376, 1344, 1270 – 1175, 1038, 756 cm^{-1}. ^1H NMR ($CDCl_3$, 600 MHz): δ = 8.30 (d, *J* = 6.8 Hz, 1H), 7.47 (d, *J* = 9.0 Hz, 1H), 7.35 (d, *J* = 7.7 Hz, 1H), 7.24 – 7.27 (m, 1H), 7.21 (d, *J* = 8.0 Hz, 1H), 7.16 (t, *J* = 7.9 Hz, 1H), 7.08 (t, *J* = 7.5 Hz, 1H), 6.82 (t, *J* = 6.7 Hz, 1H), 5.40 (d, *J* = 4.0 Hz, 1H), 4.02 – 4.11 (m, 2H), 3.80 – 3.89 (m, 2H), 3.77 (d, *J* = 4.0 Hz, 1H), 1.10 (t, *J* = 7.1 Hz, 3H), 0.91 (t, *J* = 7.1 Hz, 3H). ^{13}C NMR (DMSO-d_6, 150 MHz): δ = 168.3, 167.6, 153.7, 152.6, 141.6, 129.2, 128.7, 124.5, 124.2, 123.9, 121.6, 118.0, 116.5, 112.0, 99.1, 61.9, 61.8, 59.5, 35.0, 13.8, 13.5. HRMS (ESI/QTOF): m/z $[M+Na]^+$ calcd for $C_{21}H_{20}N_2O_5Na$: 403.1264; found: 403.1277.

Diethyl 2-(2-methoxy-12*H*-chromeno[2',3':4,5]imidazo[1,2-*a*]pyridin-12-yl)malonate (14b)

Prepared according to the **general procedure 4** using **salt 1** (155 mg, 1 mmol, 1 equiv.), 5-methoxy-2-hydroxybenzaldehyde (124 μL, 1 mmol, 1 equiv.), diethylmaonate (152 μL, 1 mmol,

1 equiv.); the product was obtained as light-yellow oil (212 mg, 0.517 mmol, 52%). R_f 0.18 (hexane / EtOAc = 1:1). IR (KBr): 3112 – 2831, 1727, 1640, 1593, 1569, 1487, 1427, 1371, 1345, 1265, 1229, 1200, 1167, 1148, 1032, 763 cm^{-1}. ^1H NMR (DMSO-d_6, 600 MHz): δ = 8.53 (d, *J* = 6.8 Hz, 1H), 7.51 (d, *J* = 8.9 Hz, 1H), 7.30 (t, *J* = 7.9 Hz, 1H), 7.20 (d, *J* = 2.2 Hz, 1H), 7.16 (d, *J* = 8.9 Hz, 1H), 7.00 (t, *J* = 6.8 Hz, 1H), 6.92 (dd, *J* = 2.4, 8.9 Hz, 1H), 5.47 (d, *J* = 2.4 Hz, 1H), 4.08 (d, *J* = 2.4 Hz, 1H), 3.92 – 3.98 (m, 2H), 3.86 – 3.90 (m, 2H), 3.75 (s, 3H), 0.97 (t, *J* = 7.1 Hz, 3H), 0.91 (t, *J* = 7.1 Hz, 3H). ^{13}C NMR (DMSO-d_6, 150 MHz): δ = 167.7, 167.3, 155.2, 153.2, 146.1, 140.7, 125.2, 124.5, 121.9, 117.9, 115.6, 114.7, 114.1, 111.9, 98.3, 61.2, 61.1, 57.3, 55.5, 34.2, 13.5, 13.4. HRMS (ESI/QTOF): m/z $[M+H]^+$ calcd for $C_{22}H_{23}N_2O_6$: 411.1550; found: 411.1564.

Diethyl 2-(14*H*-benzo[5',6']chromeno[2',3':4,5]imidazo[1,2-*a*]pyridin-14-yl)malonate (14c)

Prepared according to the **general procedure 4** using **salt 1** (155 mg, 1 mmol, 1 equiv.), 2-hydroxy-1-naphthaldehyde (152 mg, 1 mmol, 1 equiv.), diethylmaonate (152 μL, 1 mmol, 1 equiv.); the product was obtained as light-yellow solid (173 mg, 0.402 mmol, 40%); mp 139°C. IR (KBr): 3091 – 2933, 1738, 1647, 1590, 1464 – 1156, 1032, 817, 755 – 737 cm^{-1}. ^1H NMR (DMSO-d_6, 600 MHz): δ = 8.64 (d, *J* = 6.7 Hz, 1H), 8.03 (d, *J* = 8.0 Hz, 1H), 7.99 (t, *J* = 9.0 Hz, 1H), 7.85 (d, *J* = 8.4 Hz, 1H), 7.71 – 7.72 (m, 1H), 7.54 – 7.57 (m, 2H), 6.49 (d, *J* = 9.0 Hz, 1H), 7.35 (t, *J* = 7.9 Hz, 1H), 7.08 (t, *J* = 6.8 Hz, 1H), 6.23 (s, 1H), 4.20 – 4.30 (m, 2H), 3.80 (d, *J* = 1.5 Hz, 1H), 3.49 – 3.62 (m, 2H), 1.17 (t, *J* = 7.1 Hz, 3H), 0.67 (t, *J* = 7.1 Hz, 3H). ^{13}C NMR (DMSO-d_6, 150 MHz): δ = 168.7, 166.6, 152.6, 150.1, 140.9, 130.7, 130.2, 129.8, 129.2, 127.8, 125.7, 124.9, 124.8, 121.8, 118.2, 115.7, 114.3, 111.9, 99.0, 61.9, 61.0, 58.0, 31.2, 13.8, 13.0. HRMS (ESI/QTOF): m/z $[M+H]^+$ calcd for $C_{25}H_{23}N_2O_5$: 431.1599; found: 431.1601.

6-(Cyanomethyl)thieno[2,3-*c*]pyridin-6-ium chloride (16)

A solution of 1.0 g (5.7 mmol) thieno[2,3-*c*]pyridine [2] and 0.71 mL (11.2 mmol) of chloroacetonitrile in 4 mL of acetonitrile was refluxed during 48 h. The precipitate was filtered off and washed with acetonitrile for 3 times, then dried on air to give 1.20 g of salt (77%), beige

solid; mp 195 °C. IR (KBr): 3115 - 3091, 2993 - 2588, 2253, 1647, 1545, 1495, 1426, 1220, 1195, 1170 – 939, 855, 839, 796, 778, 710, 592 cm^{-1}.

^{1}H NMR (DMSO-d$_6$, 400 MHz): δ = 10.25 (s, 1H), 9.0 (d, *J* = 6.8 Hz, 1H), 8.98 (d, *J* = 5.5 Hz, 1H), 8.61 (d, *J* = 6.8 Hz, 1H), 7.98 (d, *J* = 5.5 Hz, 1H), 6.27 (s, 2H, CH$_2$). ^{13}C NMR (DMSO-d$_6$, 100 MHz): δ = 149.7, 147.7, 142.7, 137.0, 136.8, 124.2, 121.3, 114.6, 46.9. ESI-MS: m/z = 175 [M-Cl]$^+$. Anal. Calcd for $C_9H_7ClN_2S$ (210.68): C, 51.31; H, 3.35; N, 13.30; Found: C 51.29; H 3.31; N 13.28.

7-(1H-Pyrazol-1-yl)-7*H*-chromeno[2',3':4,5]imidazo[1,2-*a*]thieno[2,3-*c*]pyridine (17)

Prepared according to the **general procedure 2** using **salt 16** (210 mg, 1 mmol, 1 equiv.), salicylaldehyde (106 µL, 1 mmol, 1 equiv.) and pyrazole (204 mg, 3 mmol, 3 equiv.) the product was obtained as light brown solid (197 mg, 0.573 mmol, 57%); mp 198°C. IR (KBr): 3097, 2929 – 2852, 1638, 1611, 1571, 1460 – 1434, 1371, 1271, 1214, 1177, 751,647,630 cm^{-1}. ^{1}H NMR (DMSO-d$_6$, 600 MHz): δ = 7.91 (d, *J*=2.0 Hz, 1H), 7.89 (d, *J*=5.2 Hz, 1H), 7.77 (d, *J*=7.2 Hz, 1H), 7.5 (d, *J*=5.2 Hz, 1H), 7.44 (s, 1H), 7.37 – 7.43 (m, 5H), 7.18 (t, *J*=7.4 Hz, 1H), 6.24 (t, *J*=2.0 Hz, 1H). ^{13}C NMR (DMSO-d$_6$, 150 MHz): δ = 151.5, 150.3, 139.6, 137.1, 136.7, 129.8, 127.7, 129.3, 128.9, 125.3, 125.0, 124.2, 121.2, 119.5, 117.8, 109.1, 105.9, 98.7, 53.3. HRMS (ESI/QTOF): m/z [M+H]$^+$ calcd for $C_{19}H_{13}N_4OS$: 345.0804; found: 345.0807**.**

7-(1*H*-Indol-3-yl)-7*H*-chromeno[2',3':4,5]imidazo[1,2-*a*]thieno[2,3-*c*]pyridine (18)

Prepared according to the **general procedure 3** using **salt 16** (210 mg, 1 mmol, 1 equiv.), salicylaldehyde (106 µL, 1 mmol, 1 equiv.) and indole (351 mg, 3 mmol, 3 equiv.); the product was obtained as light brown solid (170 mg, 0.433 mmol, 43%); mp 263 - 265°C. IR (KBr): 3418, 3375 – 2857, 1639 – 1567, 1423, 1369, 1215, 771 – 726, 638 cm^{-1}. ^{1}H NMR (DMSO-d$_6$, 600 MHz): δ = 11.12 (br s 1H), 7.80 – 7.81 (m, 2H), 7.72 (d, *J* = 2.3 Hz, 1H), 7.42 (d, *J* = 5.2 Hz, 1H), 7.25 – 7.32 (m, 5H), 7.04 (t, *J* =.5 Hz, 1H), 6.90 – 6.94(m, 2H), 6.99 (t, *J* = 7.5 Hz, 1H), 6.10 (s, 1H). ^{13}C NMR (DMSO-d$_6$, 150 MHz): δ = 150.3, 149.8, 136.8, 135.9, 135.5, 130.7, 128.1, 127.7,

125.3, 125.2, 124.9, 124.1, 123.7, 123.6, 121.2, 121.0, 118.7, 117.7, 117.2, 114.9, 111.8, 108.3, 101.5, 31.8. HRMS (ESI/QTOF): m/z $[M+H]^+$ calcd for $C_{24}H_{16}N_3OS$: 394.1008; found: 394.0996.

7-(Nitromethyl)-7*H*-chromeno[2',3':4,5]imidazo[1,2-*a*]thieno[2,3-*c*]pyridine (19)

Prepared according to the **general procedure 1** using **salt 16** (210 mg, 1 mmol, 1 equiv.), salicylaldehyde (106 µL, 1 mmol, 1 equiv.) and nitromethane (536 µL, 10 mmol, 10 equiv.); the product was obtained as orange solid (126 mg, 0.374 mmol, 37%); mp 188°C. IR (KBr): 3109, 3043, 2897, 1634 – 1566, 1533, 1434, 1368, 1216 779 – 756 cm^{-1}. ^1H NMR (DMSO-d_6, 600 MHz): δ = 8.49 (d, *J* = 7.0 Hz, 1H), 7.9 (d, *J* = 5.1 Hz, 1H), 7.65 (d, *J* = 7.5 z, 1H), 7.56 (d, *J* = 5.1 Hz, 1H), 7.54 (d, *J* = 7.0 Hz, 1H), 7.40 (t, *J* = 7.5 Hz, 1H), 7.24 – 7.27 (m, 2H), 5.51 (m, 1H), 5.32 (dd, *J* = 4.2, 12.5 Hz, 1H), 5.17 (dd, *J* = 3.3, 12.5 Hz, 1H). ^{13}C NMR (DMSO-d_6, 150 MHz): δ = 151.54, 151.50, 136.6, 136.2, 129.7, 129.2, 128.4, 125.37, 125.0, 124.1, 122.0, 118.9, 117.5, 108.7, 97.6, 78.4, 34.0. HRMS (ESI/QTOF): m/z $[M+H]^+$ calcd for $C_{17}H_{12}N_3O_3S$: 338.0593; found: 338.0605.

7-(1*H*-Indol-3-yl)-1-methyl-1,7-dihydrochromeno[2',3':4,5]imidazo[1,2-*a*]pyrrolo[2,3-*c*]pyridine (20)

Prepared according to the **general procedure 3** using **salt 15** [3] (208 mg, 1 mmol, 1 equiv.), salicylaldehyde (106 µL, 1 mmol, 1 equiv.) and indole (351 mg, 3 mmol, 3 equiv.); the product was obtained as beige solid (118 mg, 0.303 mmol, 30%); mp 225°C. IR (KBr): 3469 – 3125, 3125 – 2907, 1649, 1628, 1572, 1425, 1379, 1201, 758, 737 cm^{-1}. ^1H NMR (DMSO-d_6, 600 MHz): δ = 11.05 (s, 1H), 7.68 (d, *J* = 2.2 Hz, 1H), 7.47 (d, *J* = 7.1 Hz, 1H), 7.25 – 7.29 (m, 5H), 7.03 (t, *J* = 6.8 Hz, 1H), 6.96 (d, *J* = 7.0 Hz, 1H), 6.91 – 6.94 (m, 2H), 6.69 (t, *J* = 7.5 Hz, 1H), 6.41 (d, *J* = 2.7 Hz, 1H), 6.04 (s, 1H), 4.21 (s, 3H). ^{13}C NMR (DMSO-d_6, 150 MHz): δ = 150.5, 149.0, 136.8, 132.3, 130.7, 128.7, 127.9, 125.3, 123.9, 123.8, 123.4, 121.9, 121.5, 121.1, 118.6, 117.9, 117.1, 116.5, 115.5, 111.7, 107.1, 102.5, 99.8, 35.2, 32.0. HRMS (ESI/QTOF): m/z $[M+H]^+$ calcd for $C_{25}H_{19}N_4O$: 391.1553; found: 391.1552.

Copies of NMR spectra

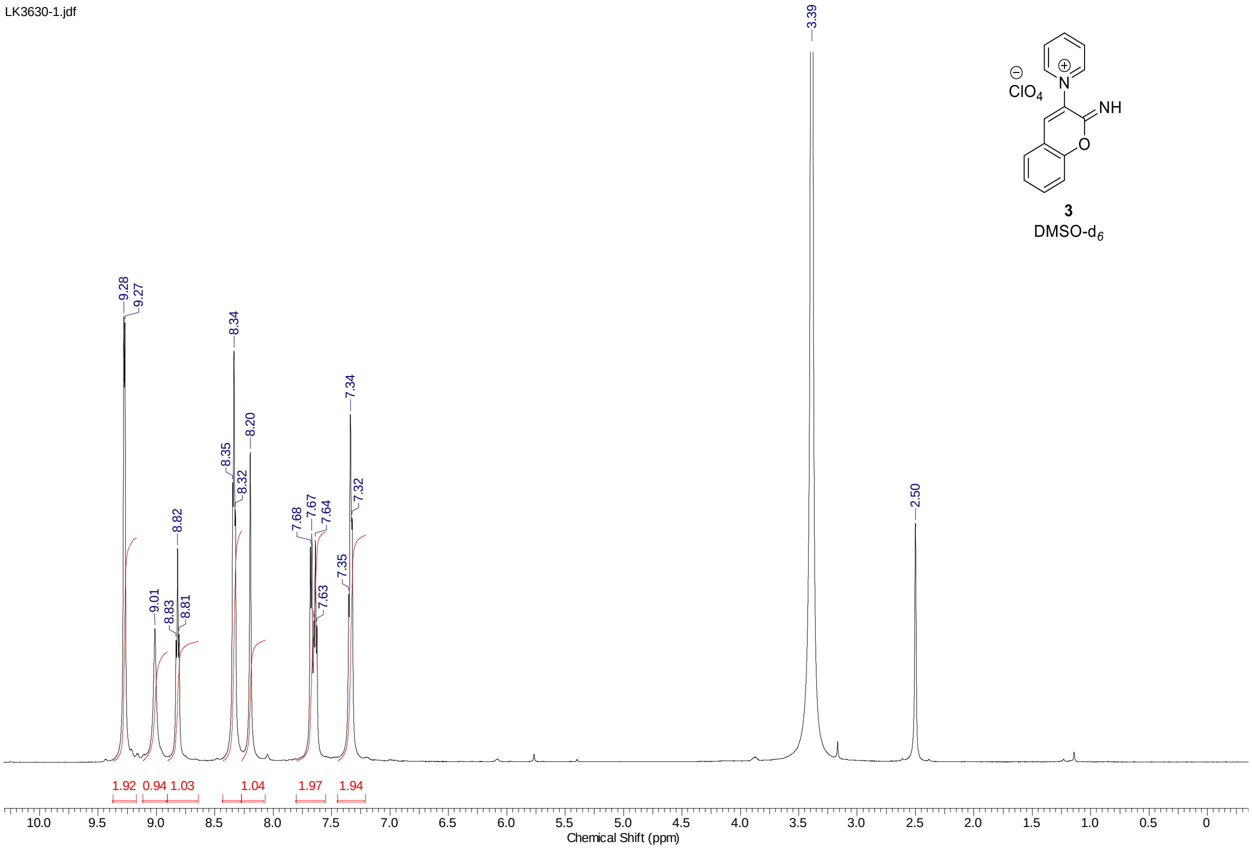

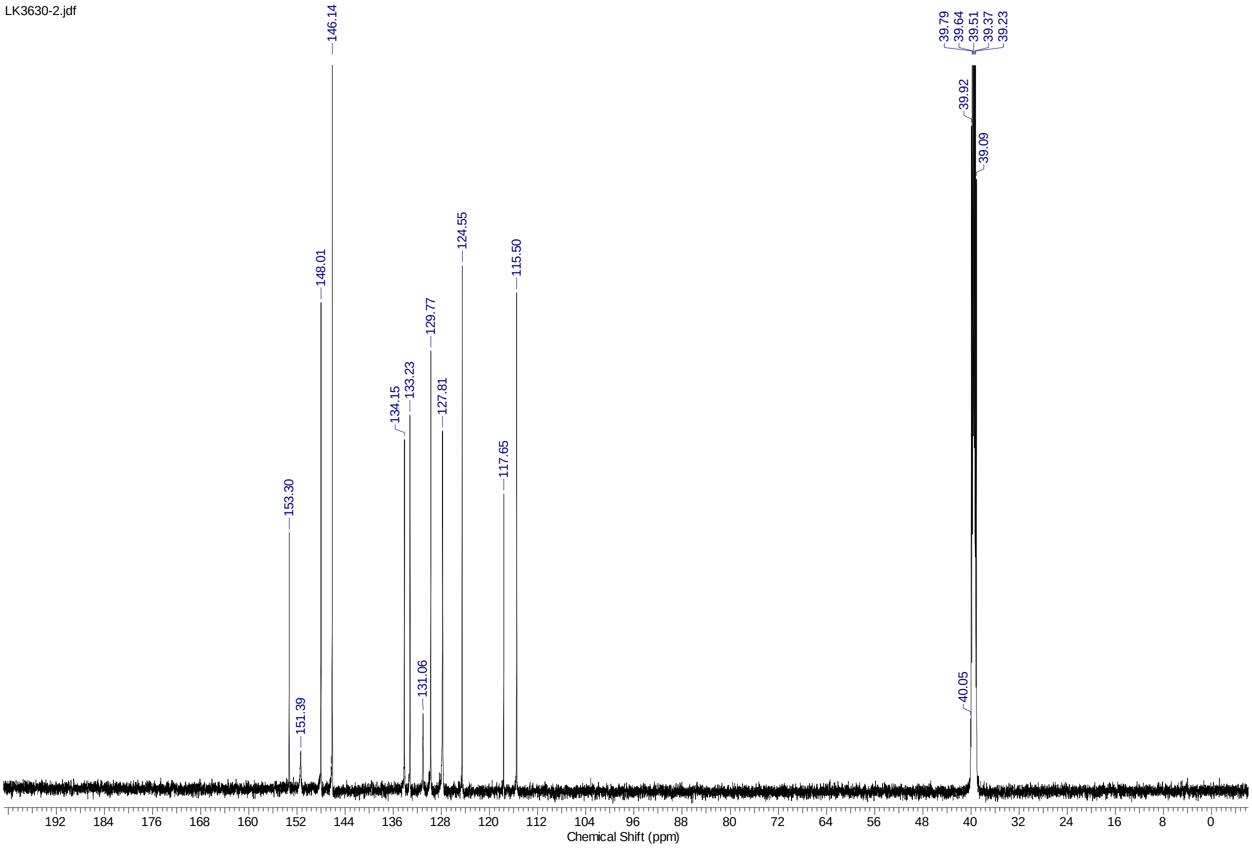

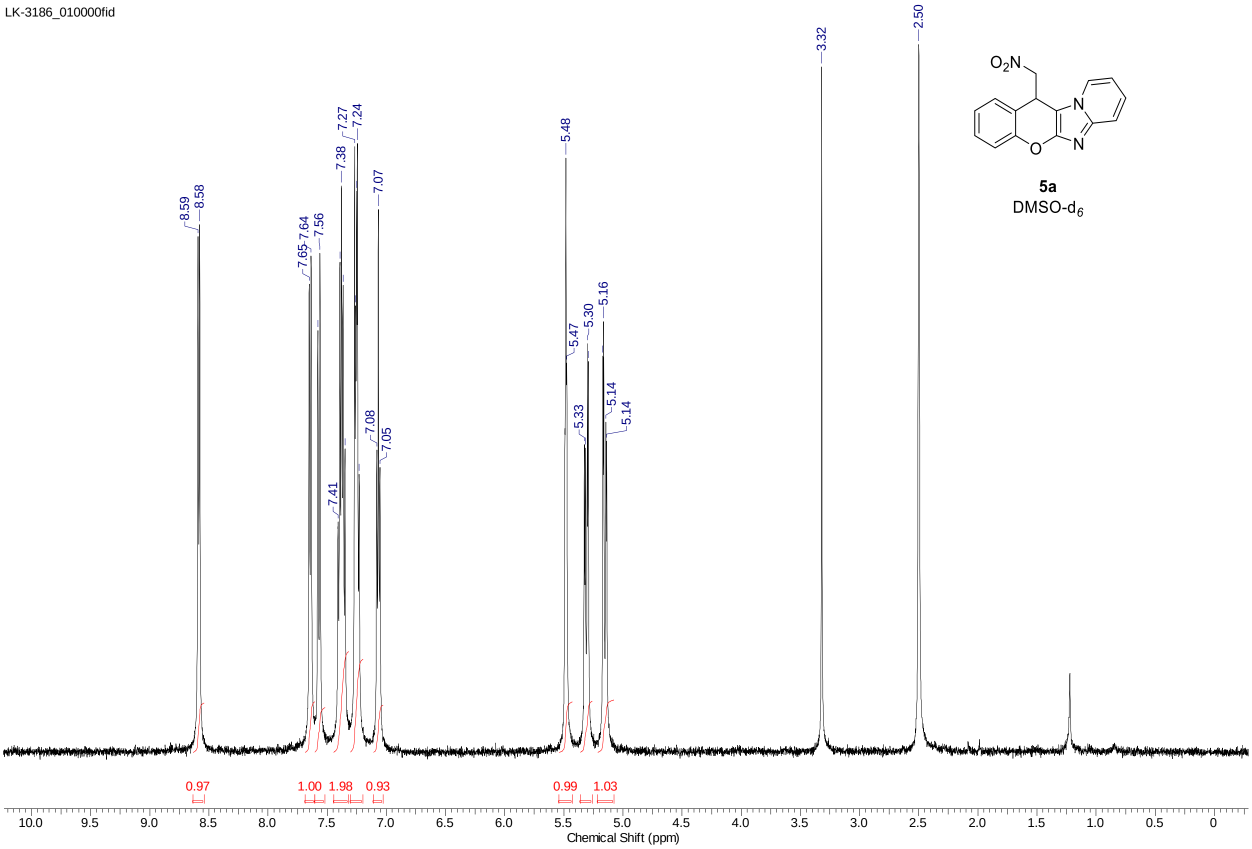
LK-3186_010000fid
8.59
8.58
7.65
7.64
7.56
7.41
7.38
7.27
7.24
7.08
7.07
7.05
5.48
5.47
5.33
5.30
5.16
5.14
5.14
3.32
2.50
0.97
1.00
1.98
0.93
0.99
1.03
10.0 9.5 9.0 8.5 8.0 7.5 7.0 6.5 6.0 5.5 5.0 4.5 4.0 3.5 3.0 2.5 2.0 1.5 1.0 0.5 0
Chemical Shift (ppm)
O2N
N
N
O

5a
DMSO-d$_6$

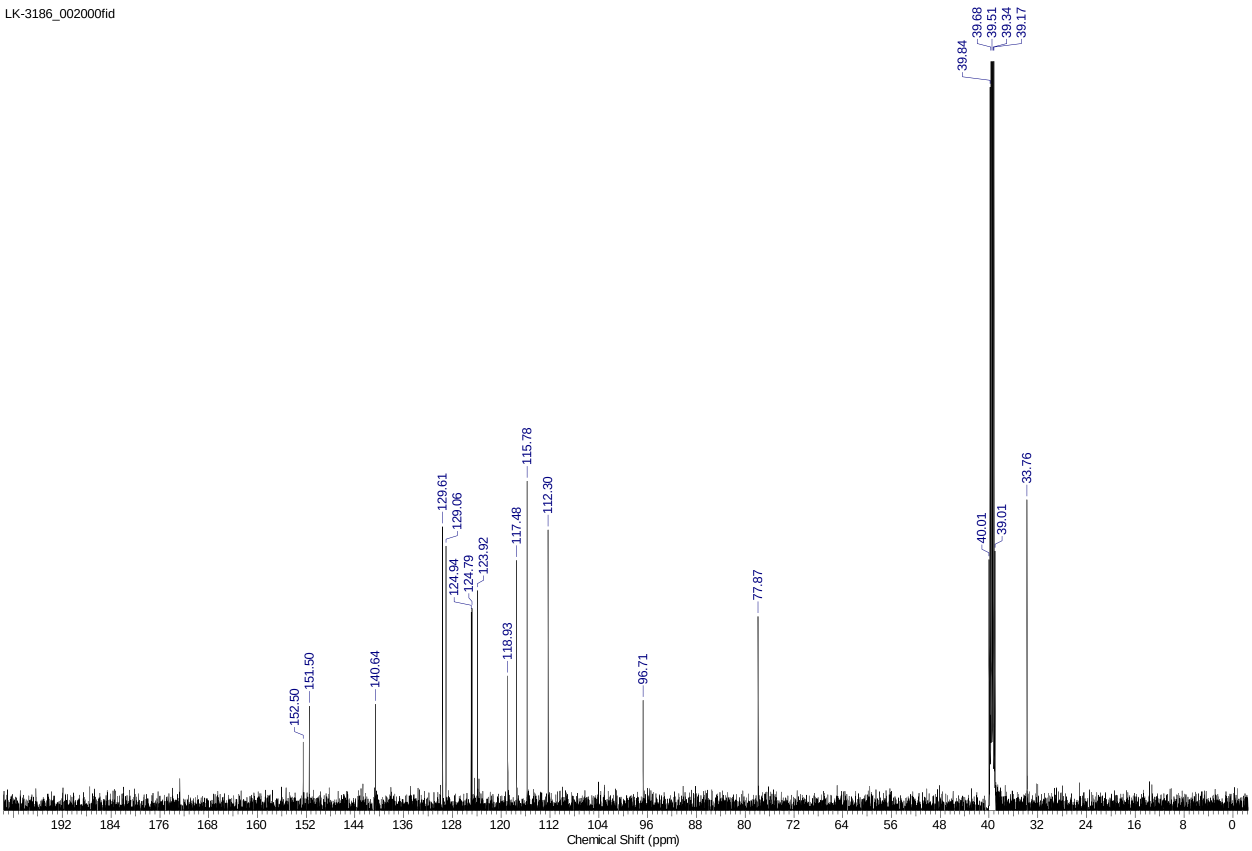
LK-3186_002000fid
39.84
39.68
39.51
39.34
39.17
152.50
151.50
140.64
129.61
129.06
124.94
124.79
123.92
118.93
117.48
115.78
112.30
96.71
77.87
40.01
39.01
33.76
192 184 176 168 160 152 144 136 128 120 112 104 96 88 80 72 64 56 48 40 32 24 16 8 0
Chemical Shift (ppm)

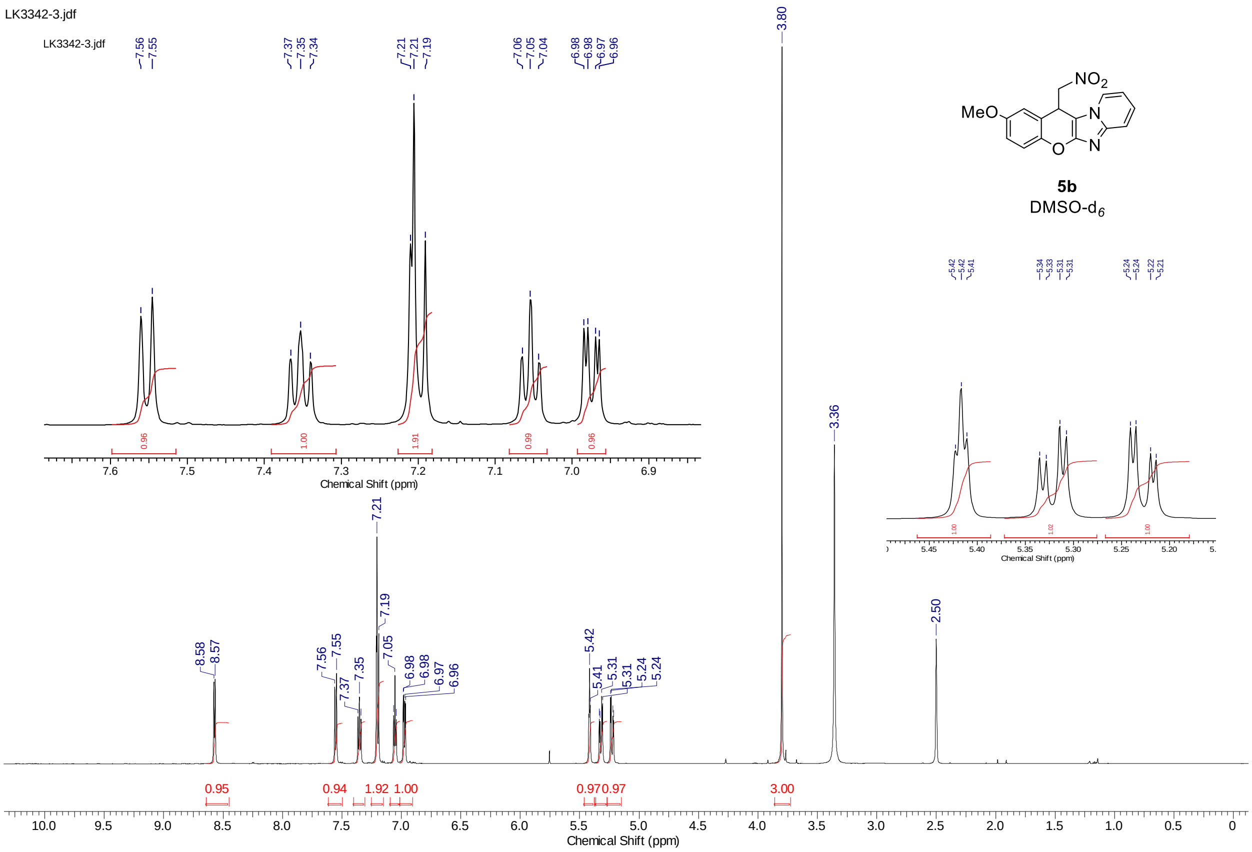
LK3342-3.jdf
MeO
NO_2
N
O
5b
DMSO-d6
Chemical Shift (ppm)

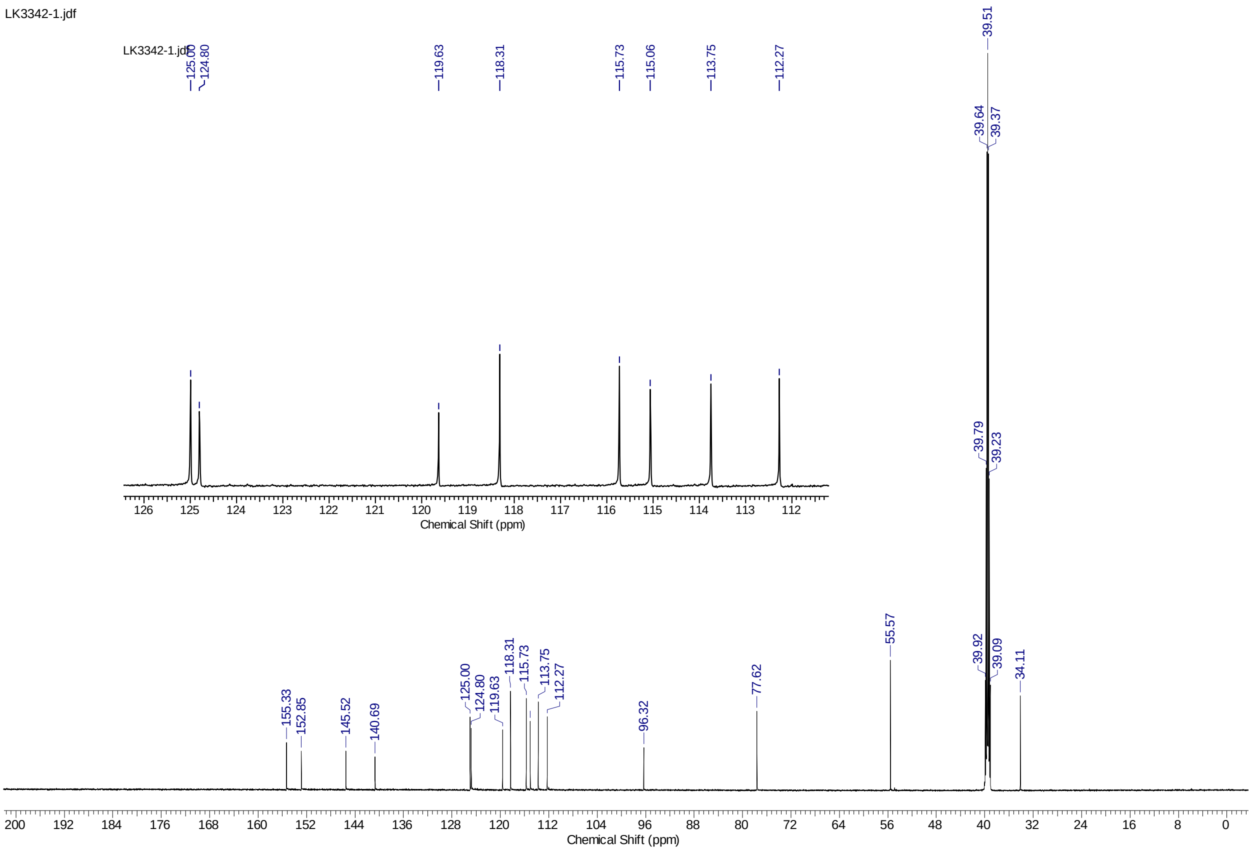
LK3342-1.jdf
Chemical Shift (ppm)

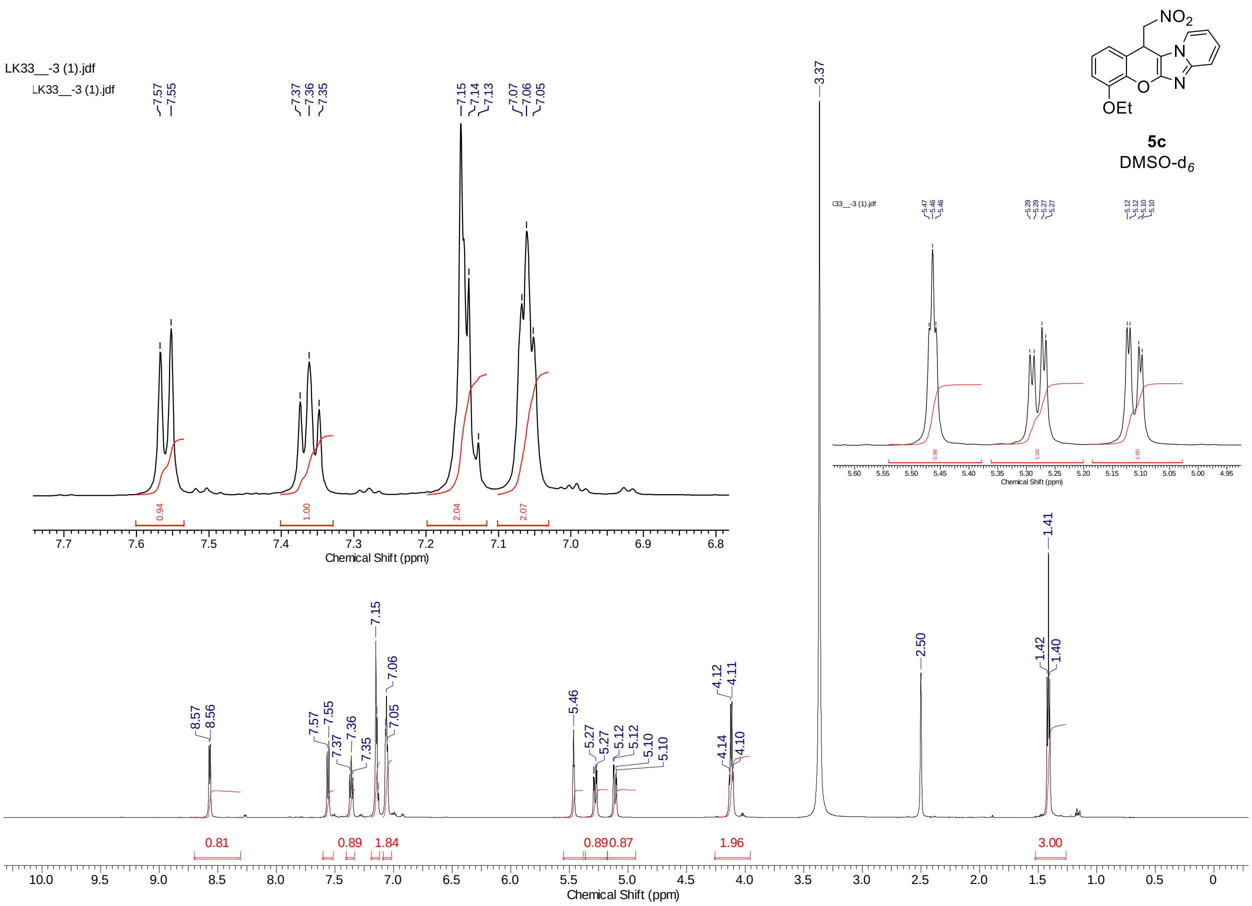
LK33__-3 (1).jdf
5c
DMSO-d6
Chemical Shift (ppm)
8.57
8.56
7.57
7.55
7.37
7.36
7.35
7.15
7.06
7.05
5.46
5.27
5.27
5.12
5.12
5.10
5.10
4.14
4.12
4.11
4.10
3.37
2.50
1.42
1.41
1.40
0.81
0.89
1.84
0.89
0.87
1.96
3.00

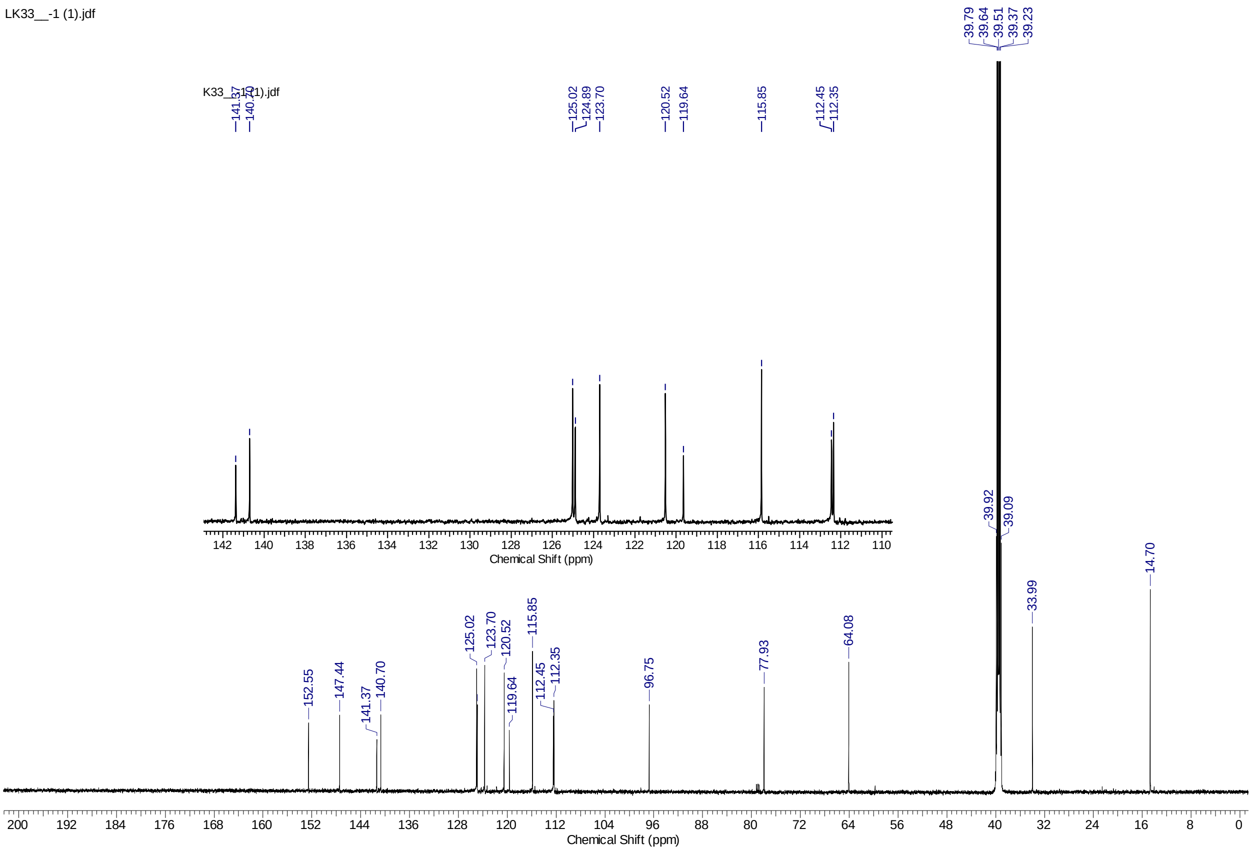
LK33__-1 (1).jdf
Chemical Shift (ppm)
152.55
147.44
141.37
140.70
125.02
124.89
123.70
120.52
119.64
115.85
112.45
112.35
96.75
77.93
64.08
39.92
39.79
39.64
39.51
39.37
39.23
39.09
33.99
14.70

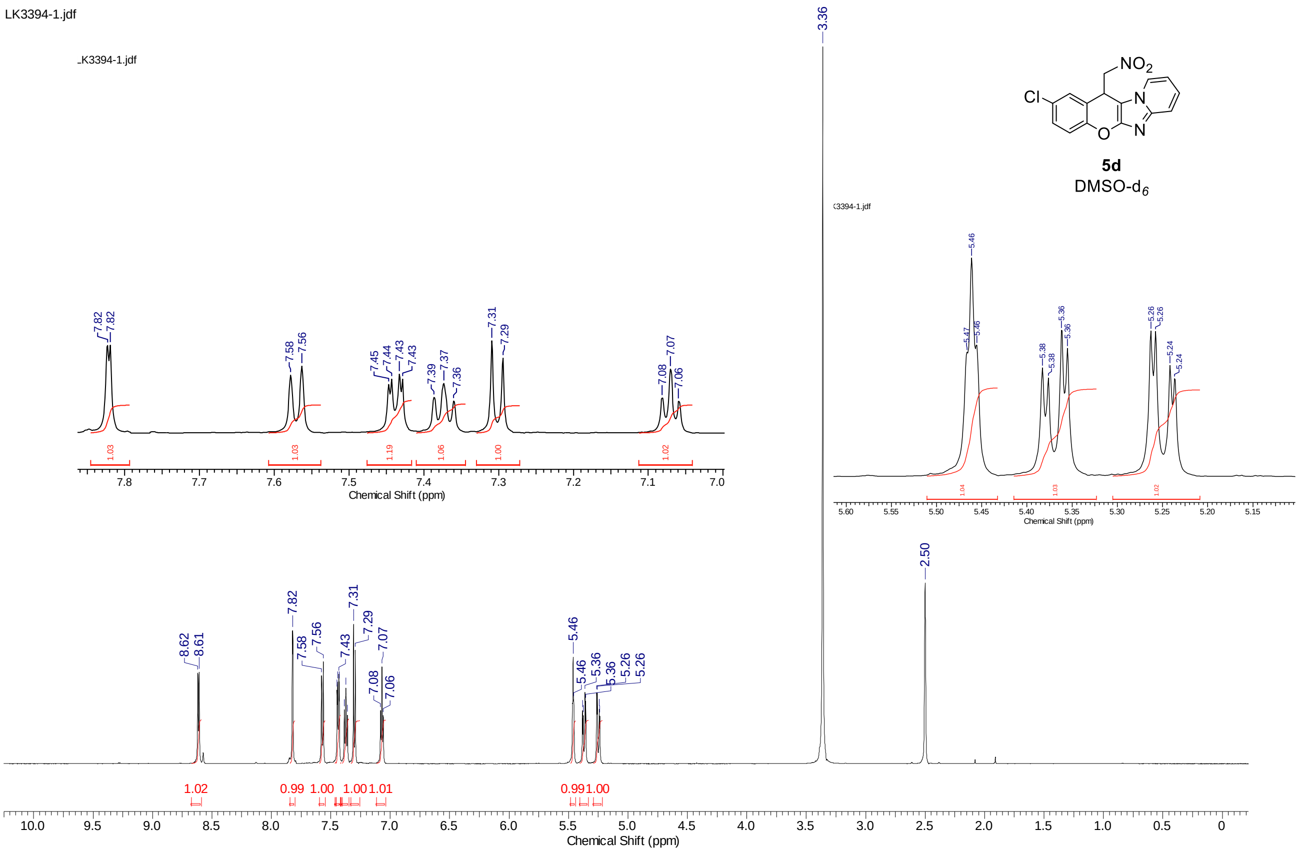
LK3394-1.jdf
3.36
2.50
8.62
8.61
7.82
7.58
7.56
7.43
7.31
7.29
7.07
7.08
7.06
5.46
5.36
5.26
1.02
0.99
1.00
1.00
1.01
0.99
1.00
Chemical Shift (ppm)
NO2
Cl
N
O
N

5d
DMSO-d_6

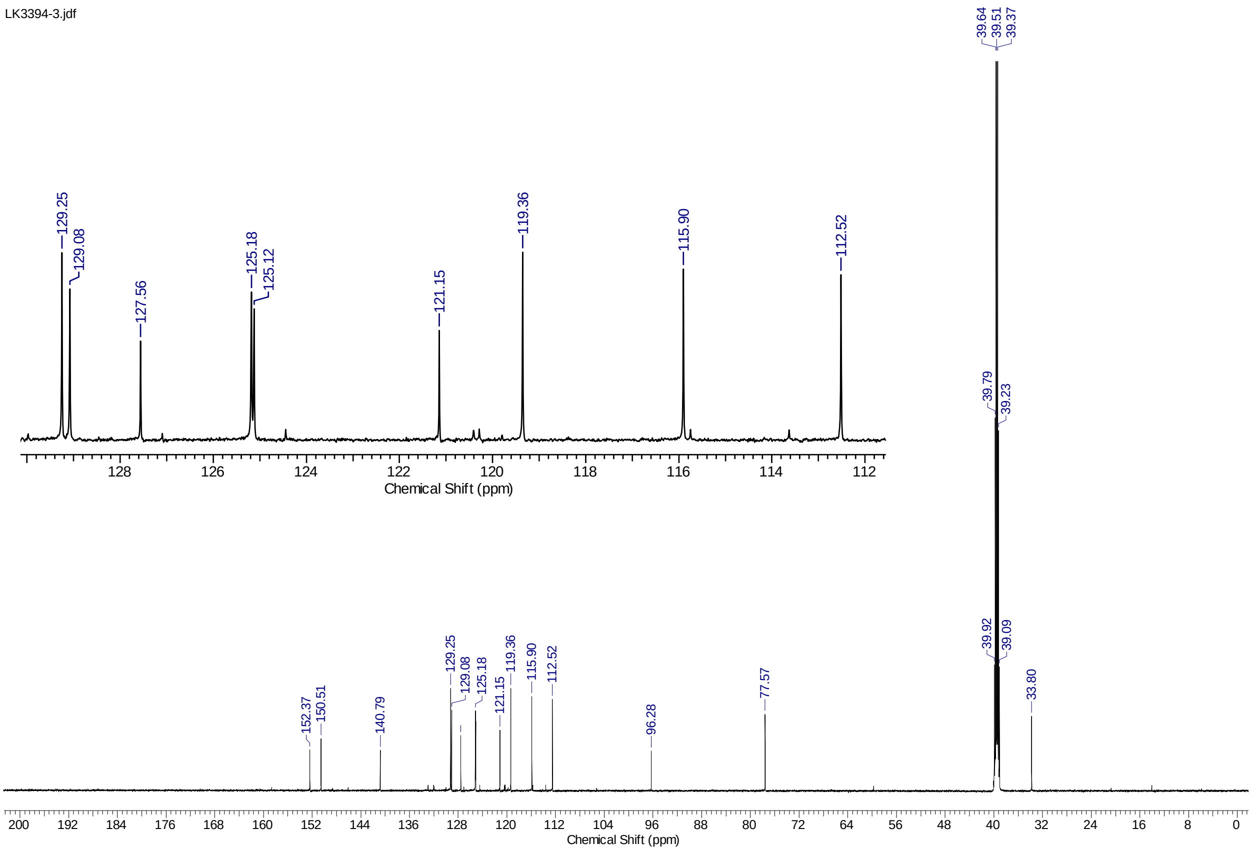
LK3394-3.jdf
39.64
39.51
39.37
129.25
129.08
127.56
125.18
125.12
121.15
119.36
115.90
112.52
39.79
39.23
39.92
39.09
152.37
150.51
140.79
96.28
77.57
33.80
Chemical Shift (ppm)

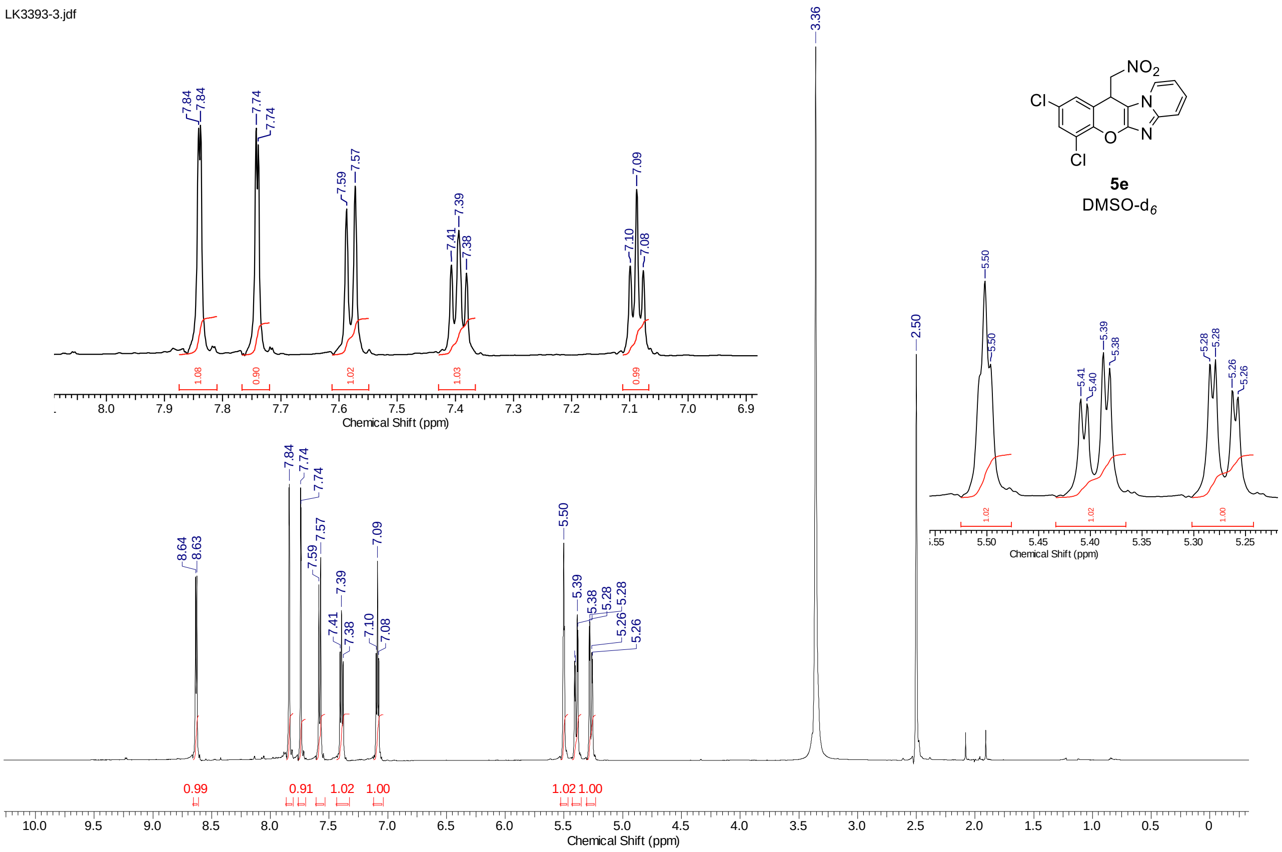
LK3393-3.jdf
5e
DMSO-d6
8.64
8.63
7.84
7.74
7.74
7.59
7.57
7.41
7.39
7.38
7.10
7.09
7.08
5.50
5.39
5.38
5.28
5.28
5.26
5.26
3.36
2.50
0.99
0.91
1.02
1.00
1.02
1.00
Chemical Shift (ppm)

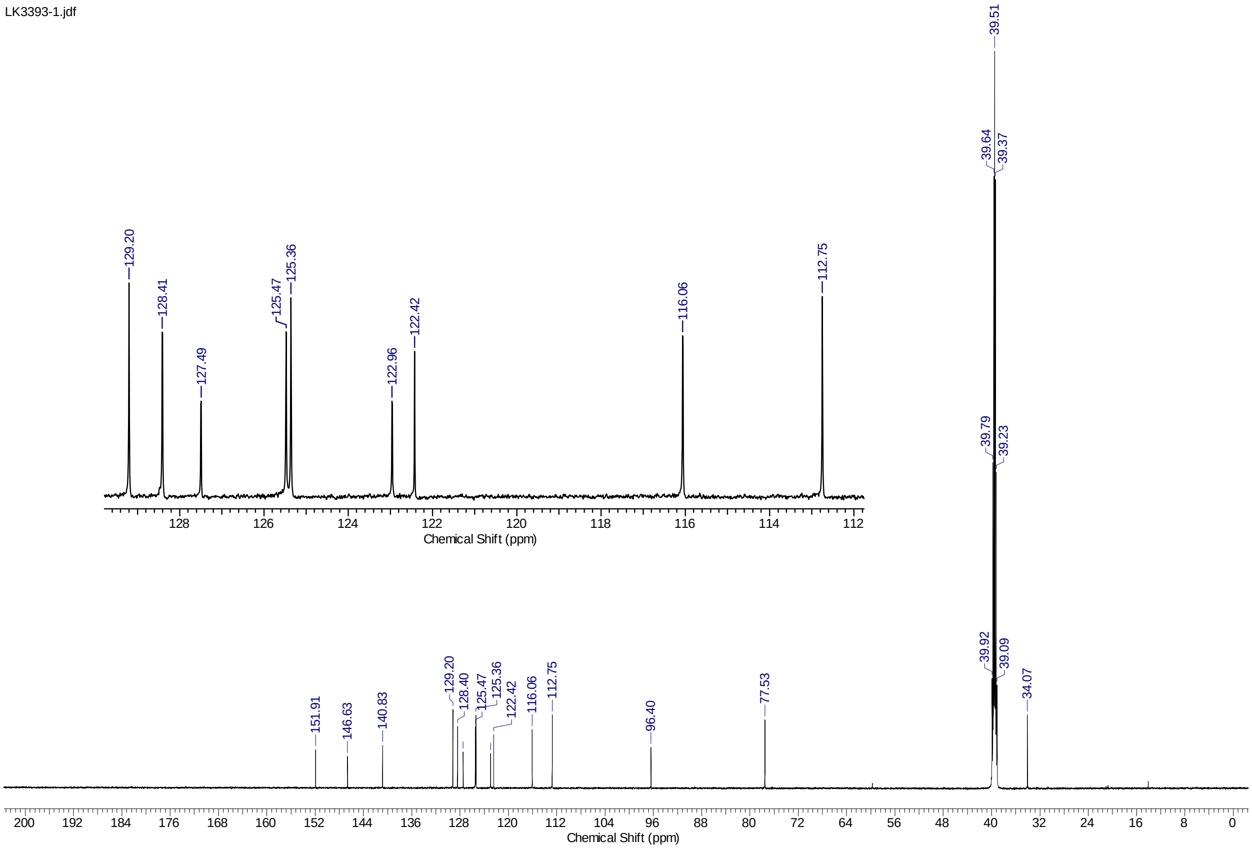
LK3393-1.jdf
151.91
146.63
140.83
129.20
128.40
125.47
125.36
122.42
116.06
112.75
96.40
77.53
39.92
39.79
39.64
39.51
39.37
39.23
39.09
34.07
129.20
128.41
127.49
125.47
125.36
122.96
122.42
116.06
112.75
Chemical Shift (ppm)

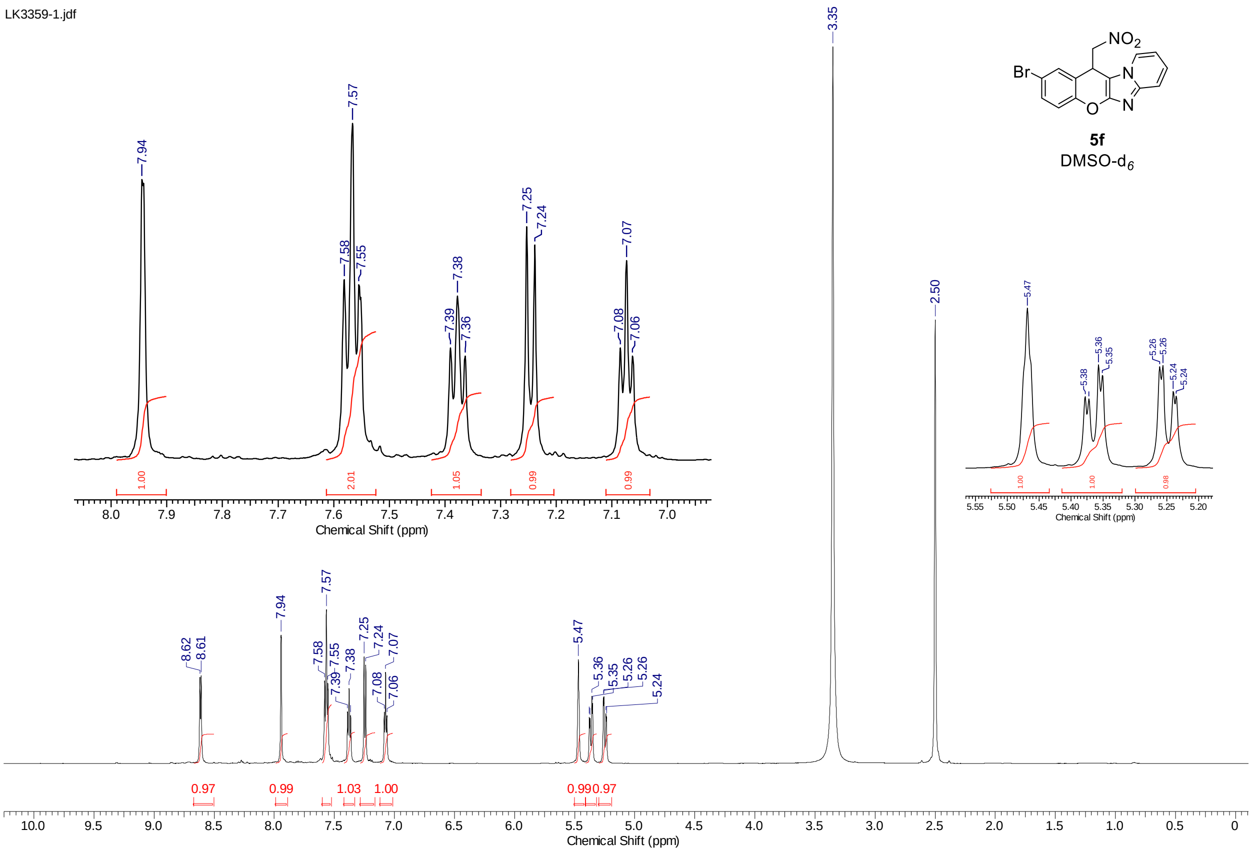
LK3359-1.jdf
5f
DMSO-d6
Chemical Shift (ppm)

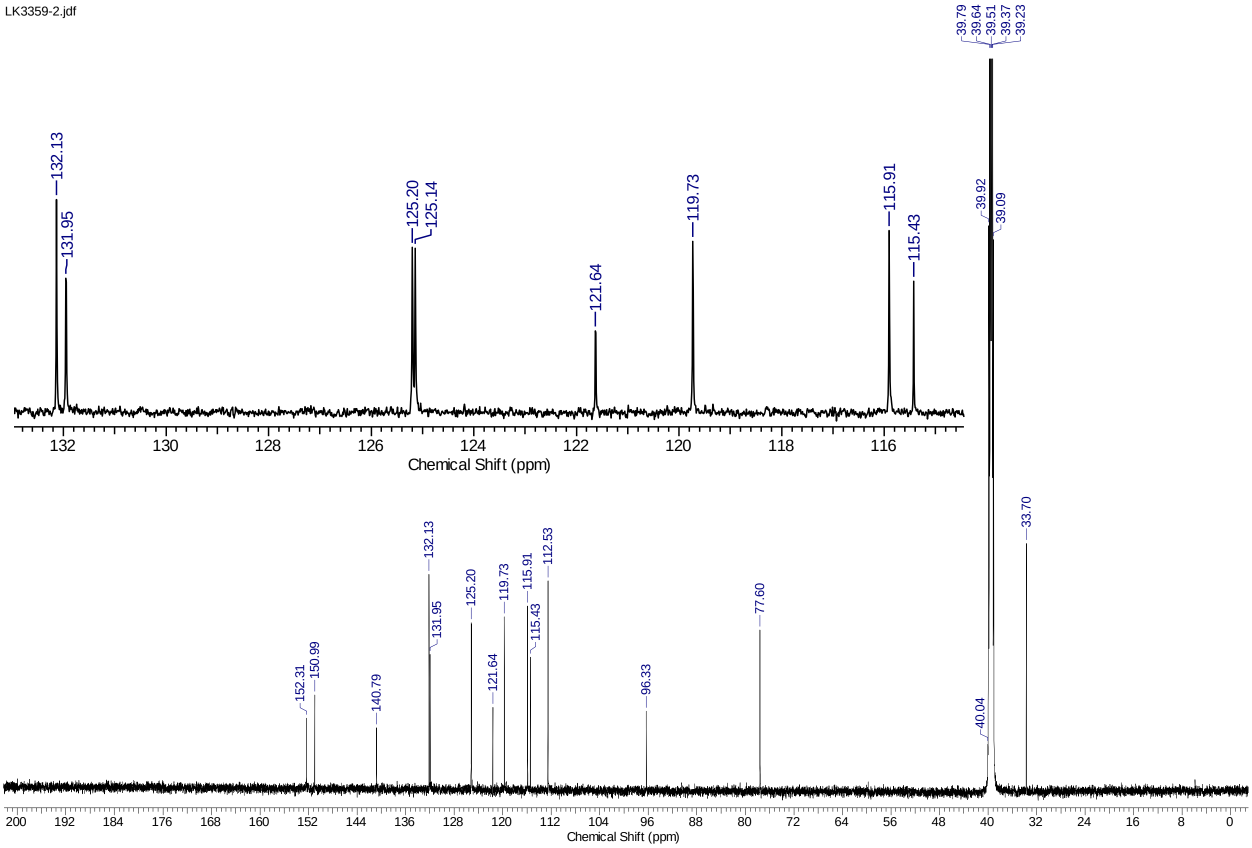
LK3359-2.jdf
Chemical Shift (ppm)

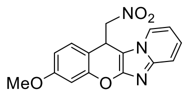
NO2
N
N
O
MeO

5g
DMSO-d6

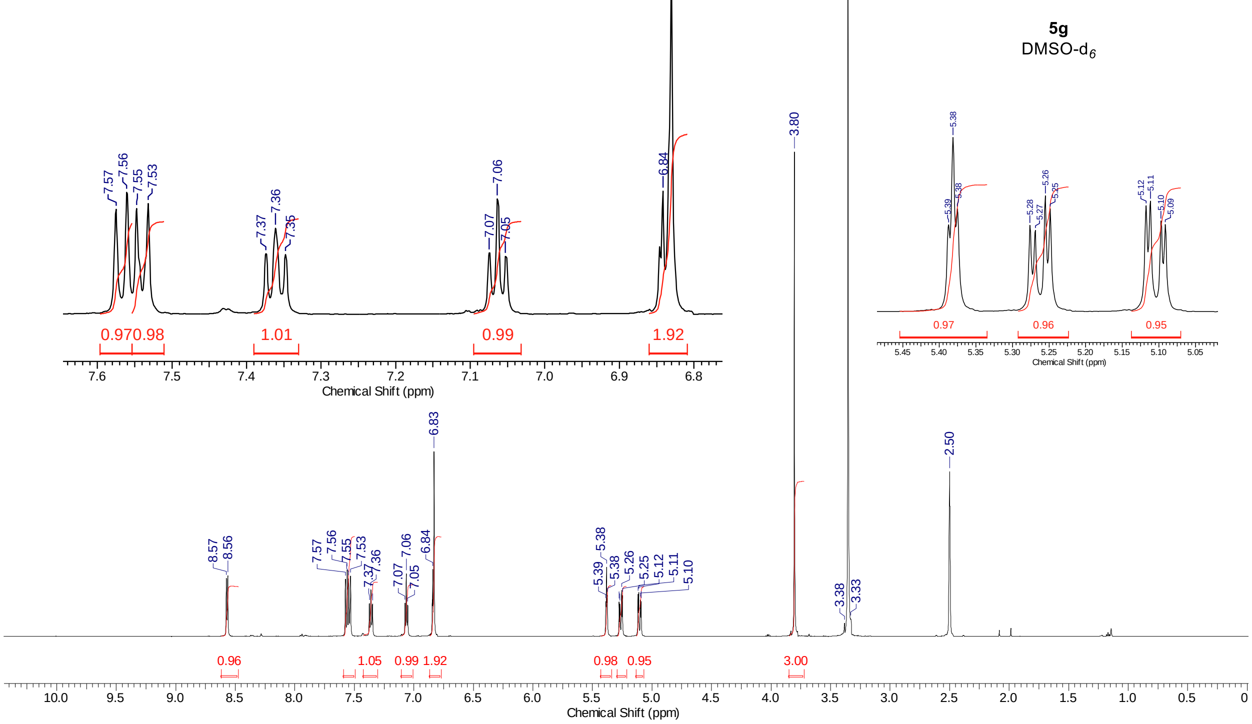
LK3392-1 (1).jdf
3.35
6.83
3.80
7.57 7.56 7.55 7.53
7.37 7.36 7.35
7.07 7.06 7.05
6.84
0.97 0.98 1.01 0.99 1.92
Chemical Shift (ppm)
5.39 5.38 5.28 5.27 5.26 5.25 5.12 5.11 5.10 5.09
0.97 0.96 0.95
8.57 8.56
5.38 5.26 5.25 5.12 5.11 5.10
3.38 3.33
2.50
0.96 1.05 0.99 1.92 0.98 0.95 3.00

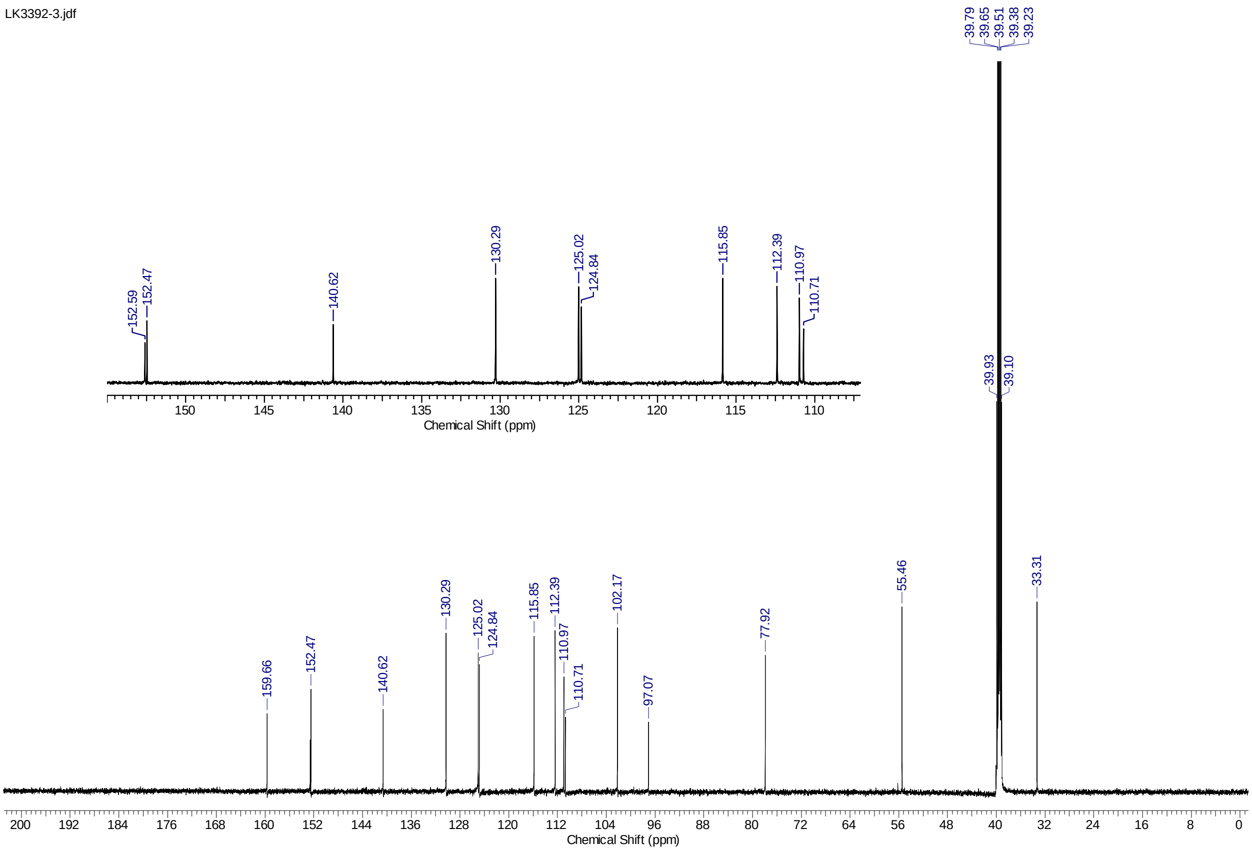
LK3392-3.jdf
39.79 39.65 39.51 39.38 39.23
39.93 39.10
152.59 152.47 140.62 130.29 125.02 124.84 115.85 112.39 110.97 110.71
159.66 102.17 97.07 77.92 55.46 33.31
Chemical Shift (ppm)

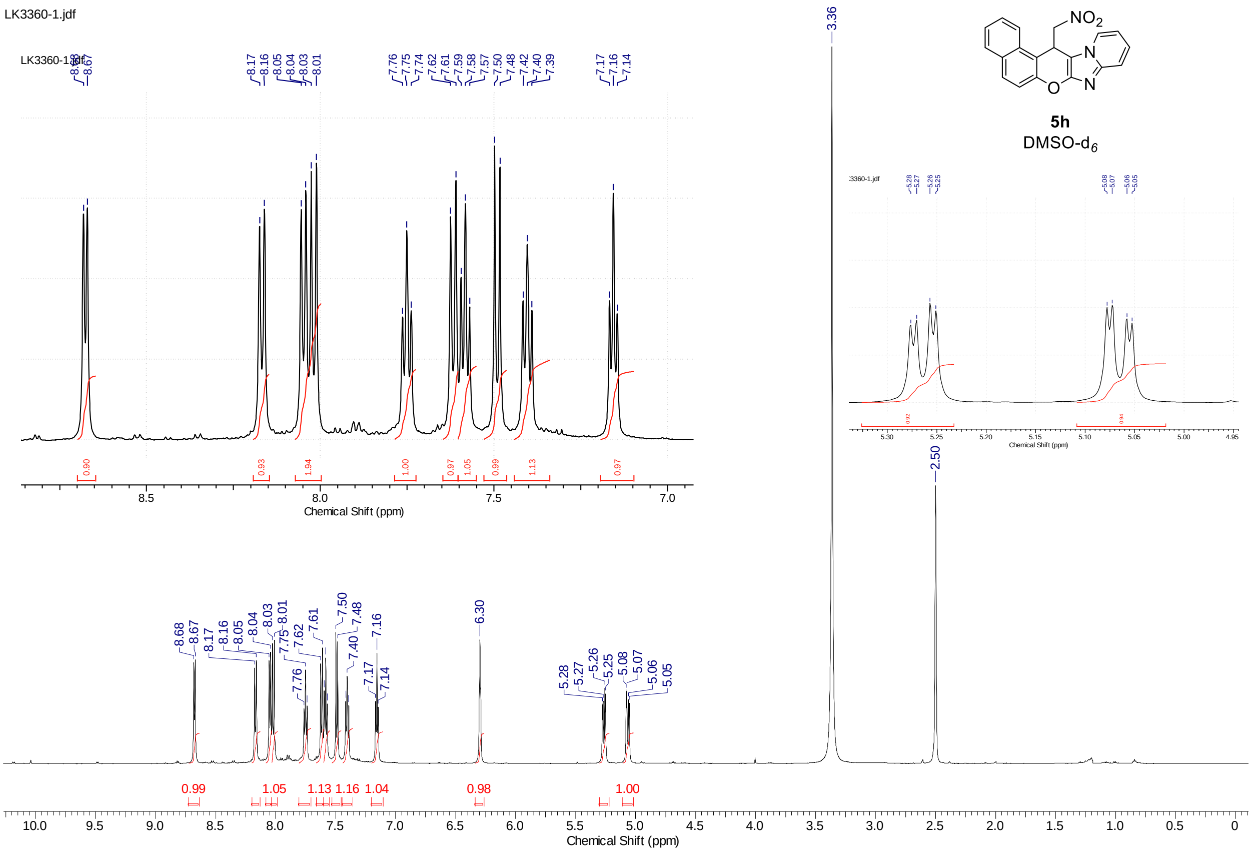
LK3360-1.jdf
Chemical Shift (ppm)

5h
DMSO-d_6

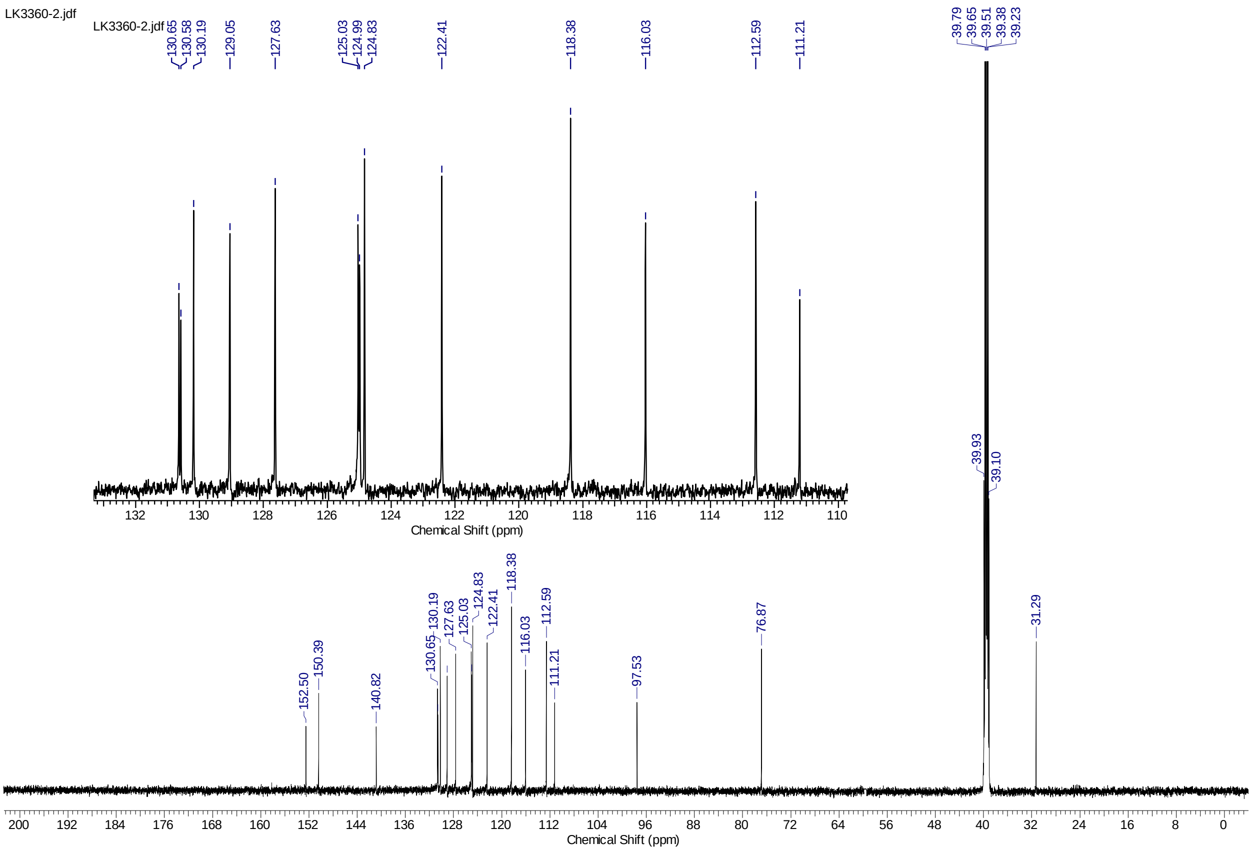
LK3360-2.jdf
Chemical Shift (ppm)

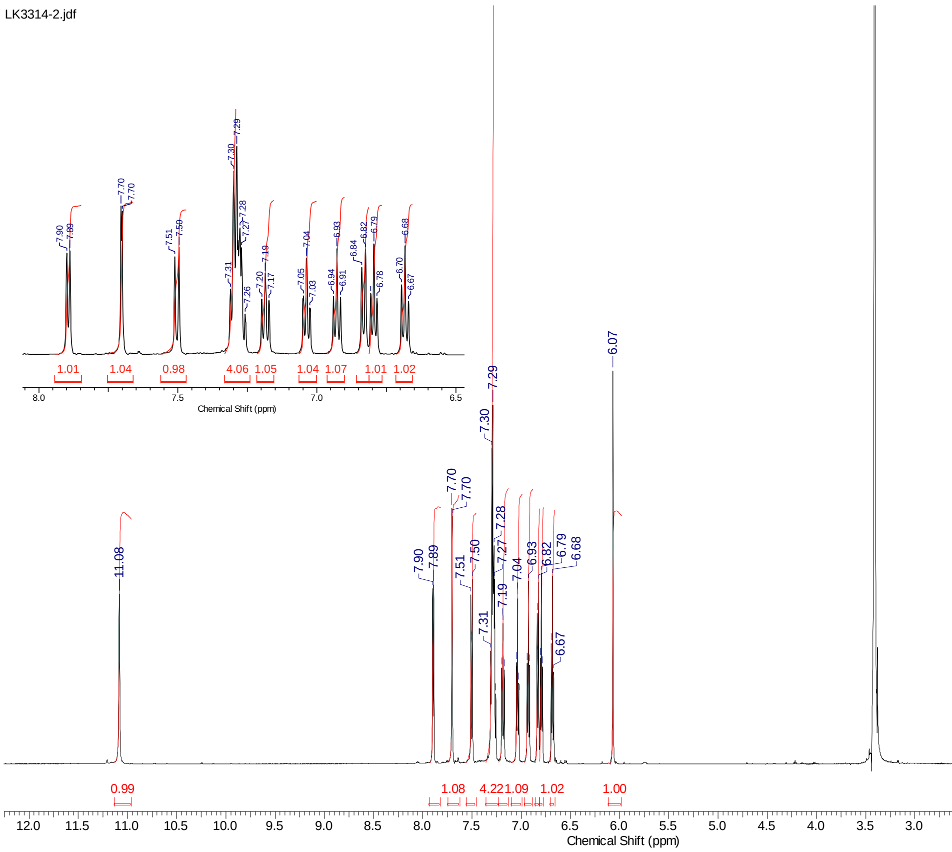
LK3314-2.jdf
11.08
7.90
7.89
7.70
7.70
7.51
7.50
7.31
7.30
7.29
7.28
7.27
7.19
7.04
6.93
6.82
6.79
6.68
6.67
6.07
0.99
1.08
4.22
1.09
1.02
1.00
1.01
1.04
0.98
4.06
1.05
1.04
1.07
1.01
1.02
Chemical Shift (ppm)

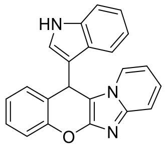

6a
DMSO-d$_6$

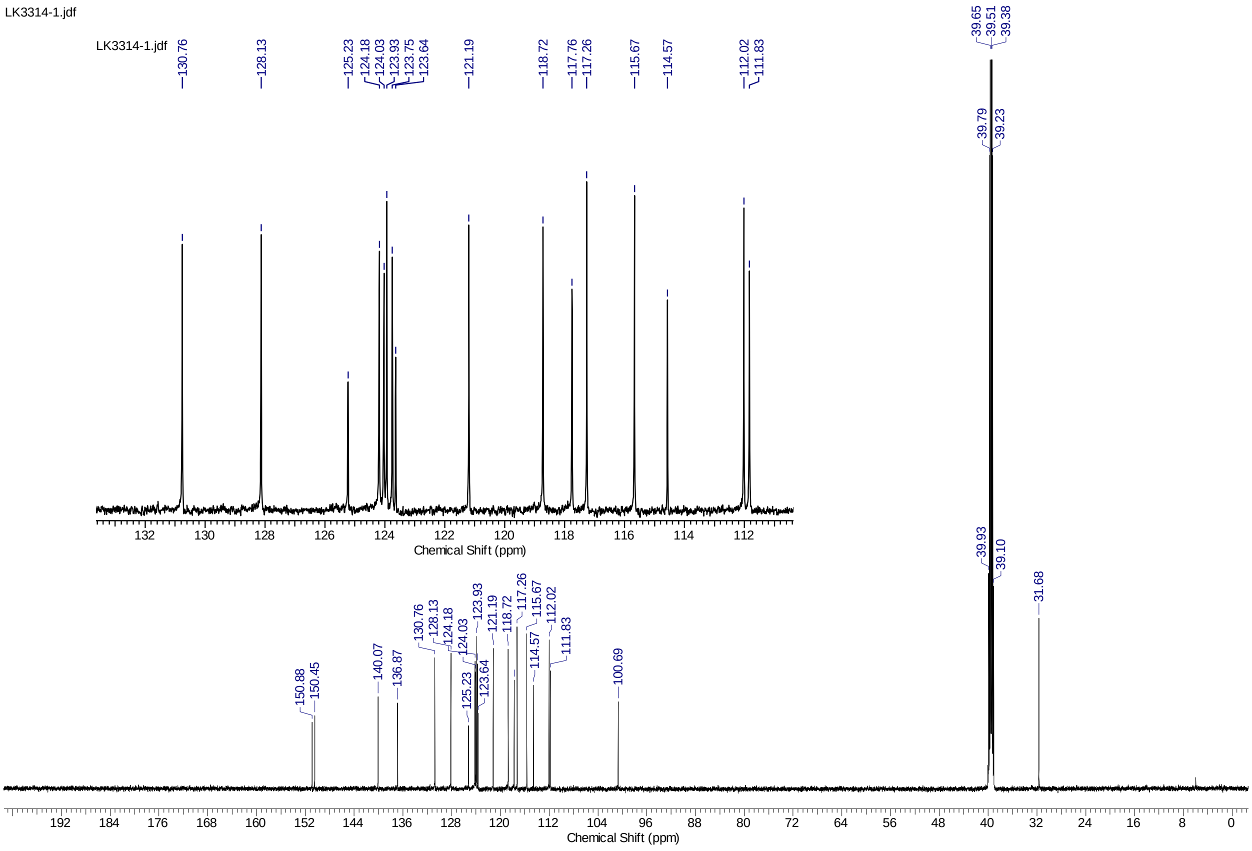
LK3314-1.jdf
39.65
39.51
39.38
39.79
39.23
130.76
128.13
125.23
124.18
124.03
123.93
123.75
123.64
121.19
118.72
117.76
117.26
115.67
114.57
112.02
111.83
150.88
150.45
140.07
136.87
100.69
39.93
39.10
31.68
Chemical Shift (ppm)

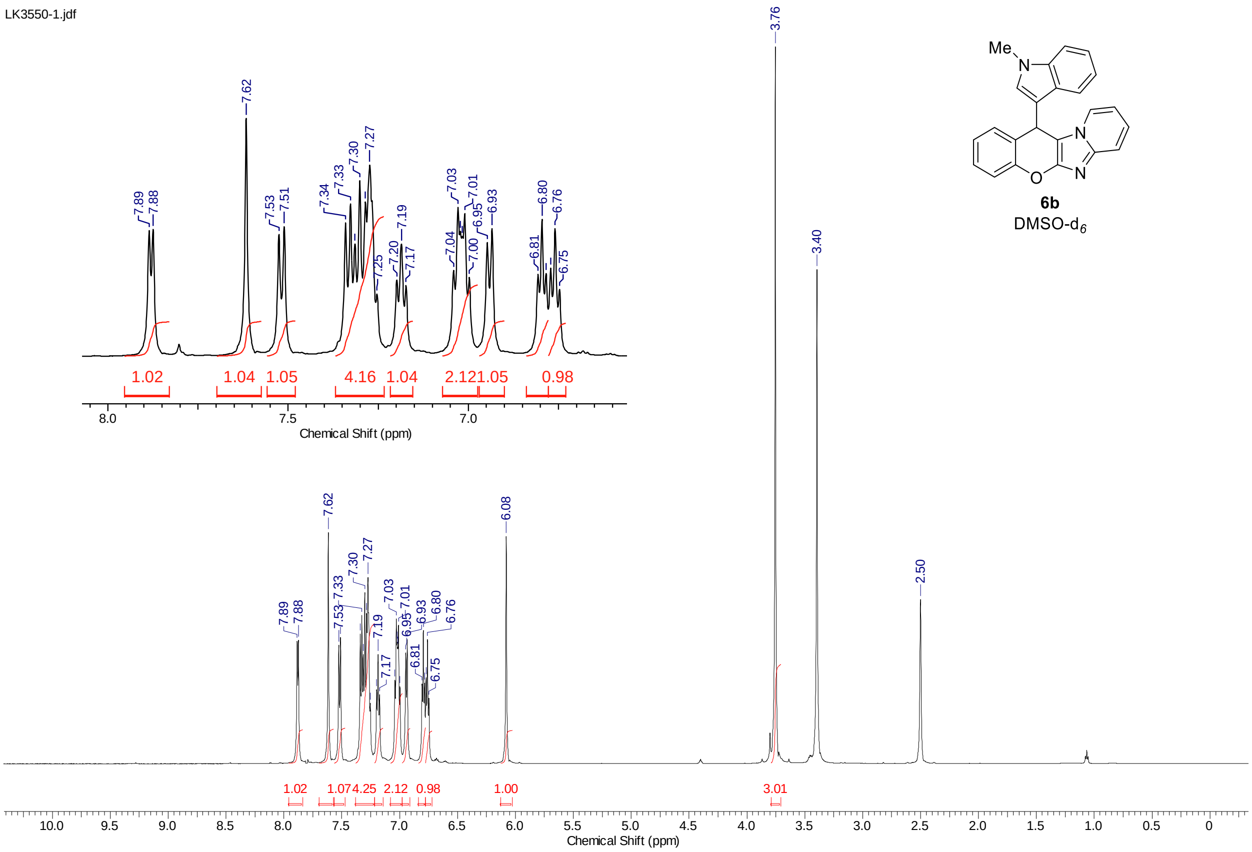
LK3550-1.jdf
Chemical Shift (ppm)
Me
N
N
N
O

6b
DMSO-d_6

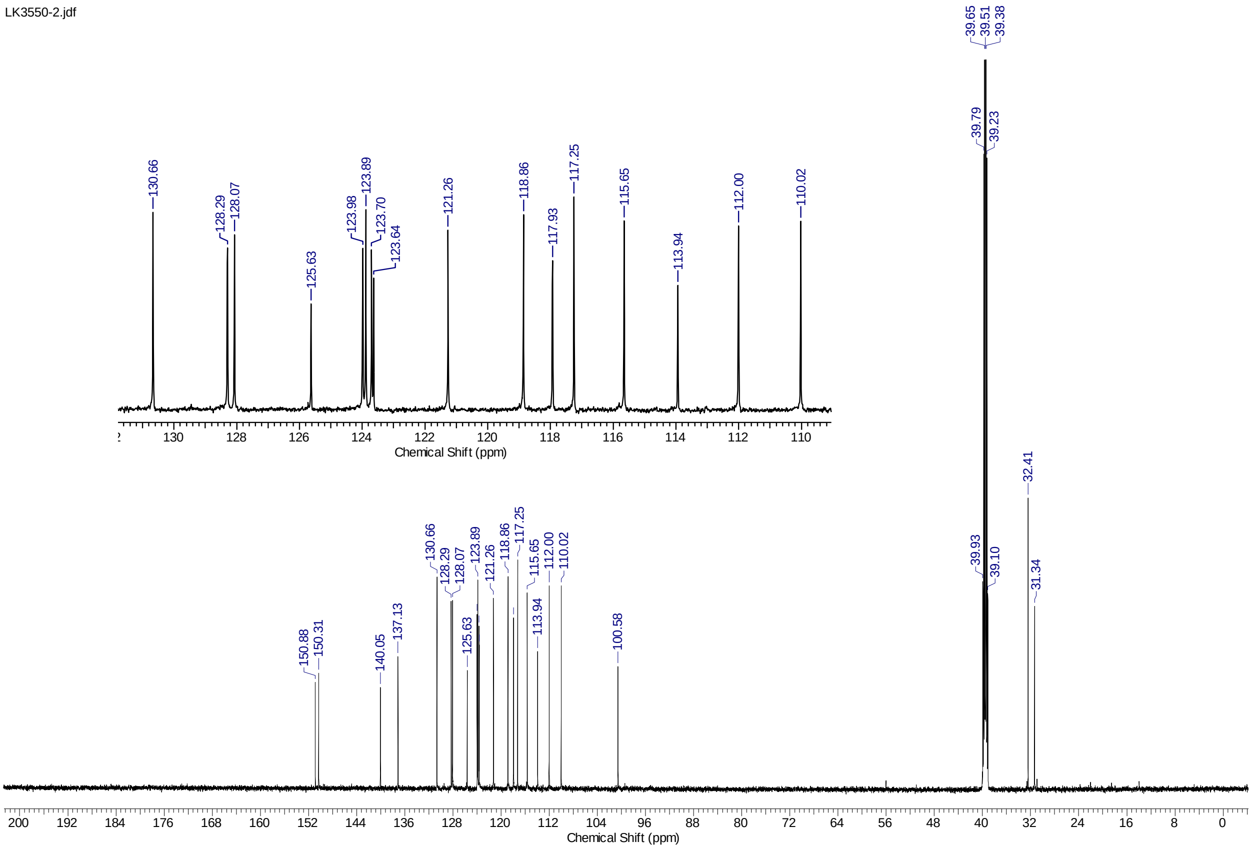
LK3550-2.jdf
Chemical Shift (ppm)

LK3693-1 (1).jdf

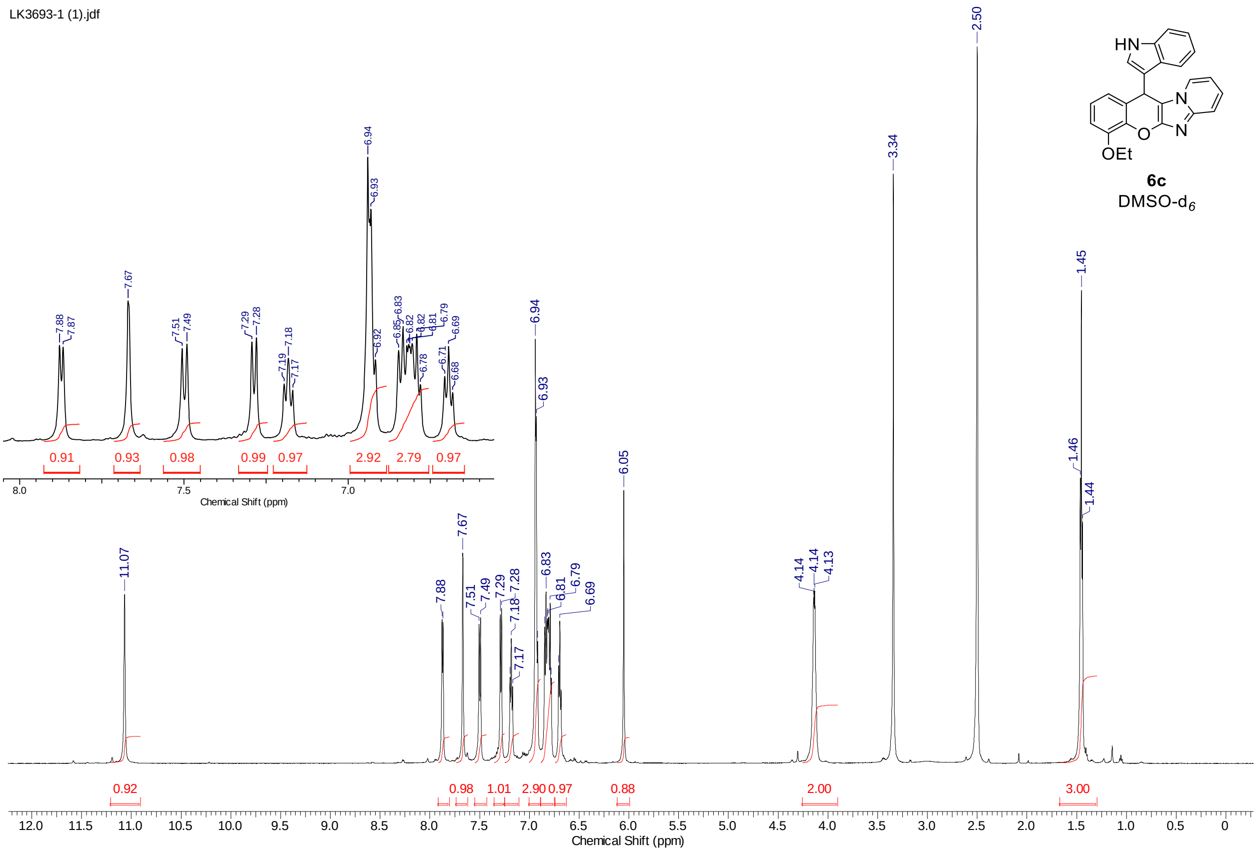

LK3693-2.jdf

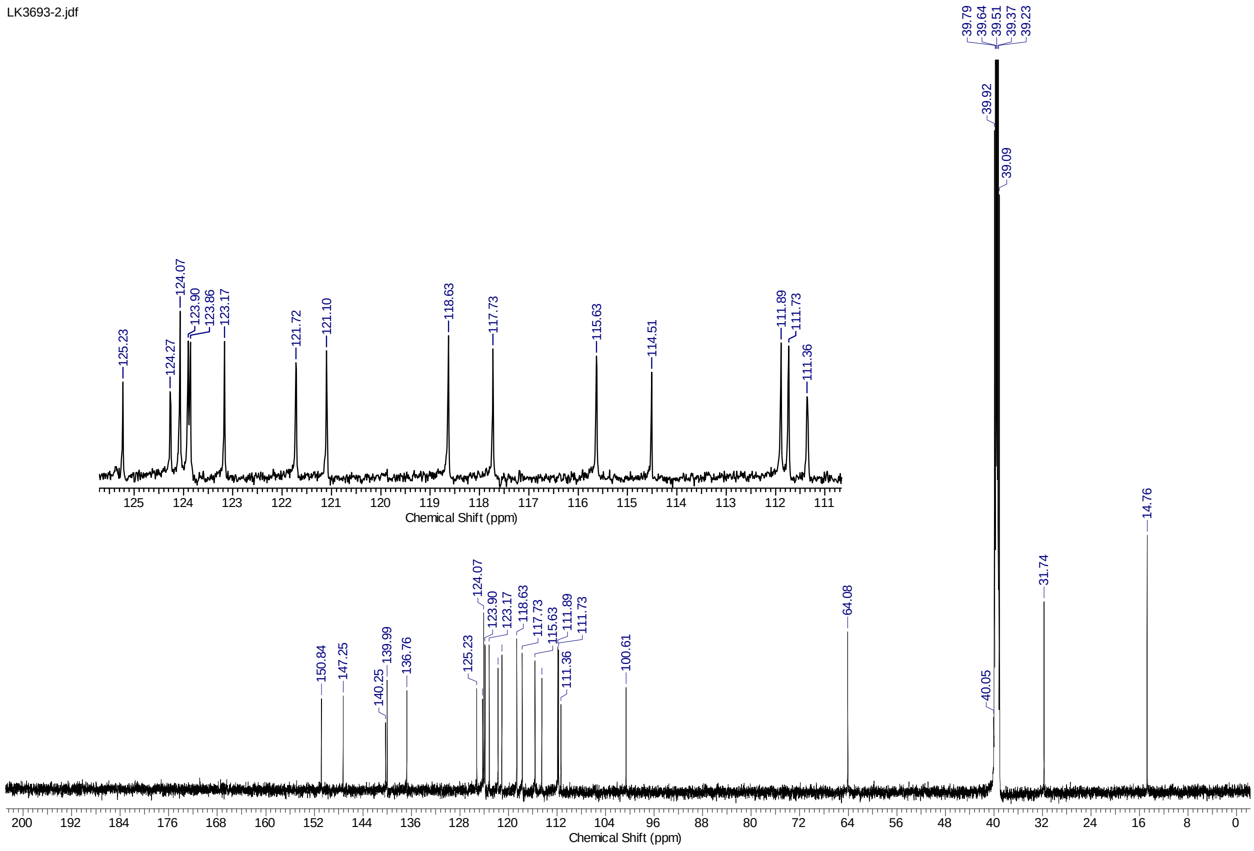

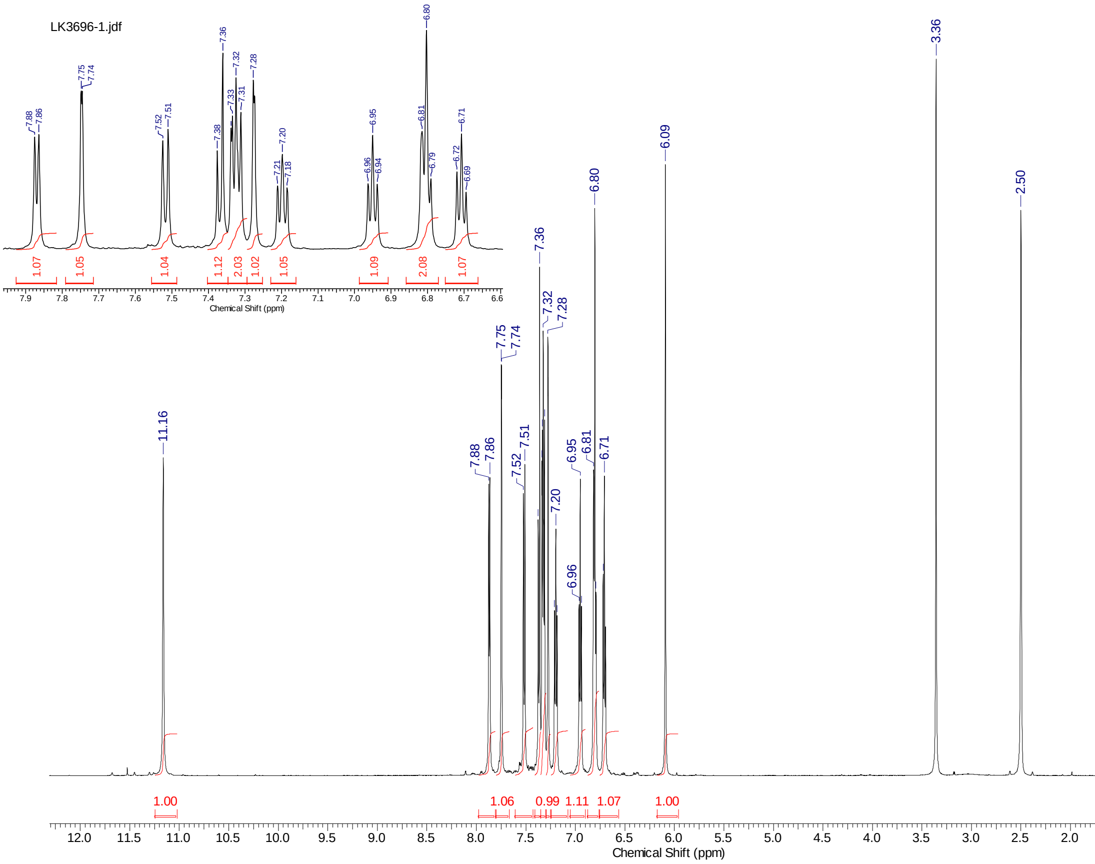
LK3696-1.jdf
11.16
7.88
7.86
7.75
7.74
7.52
7.51
7.36
7.32
7.28
7.20
6.96
6.95
6.81
6.80
6.71
6.09
3.36
2.50
Chemical Shift (ppm)

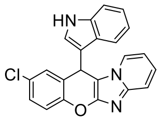
HN
Cl
O
N
N

6d

DMSO-d$_6$

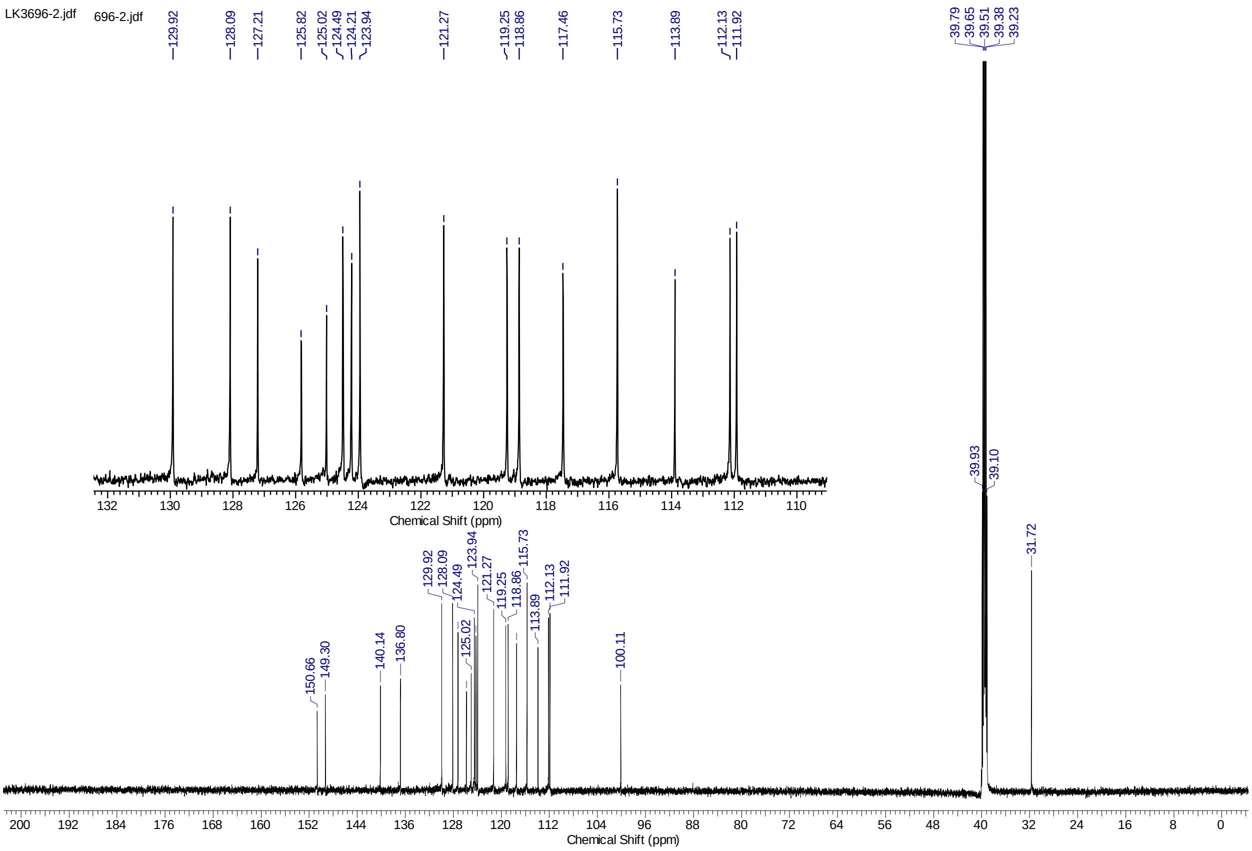
LK3696-2.jdf
696-2.jdf
150.66
149.30
140.14
136.80
129.92
128.09
125.02
124.49
123.94
121.27
119.25
118.86
115.73
113.89
112.13
111.92
100.11
39.93
39.10
31.72
Chemical Shift (ppm)

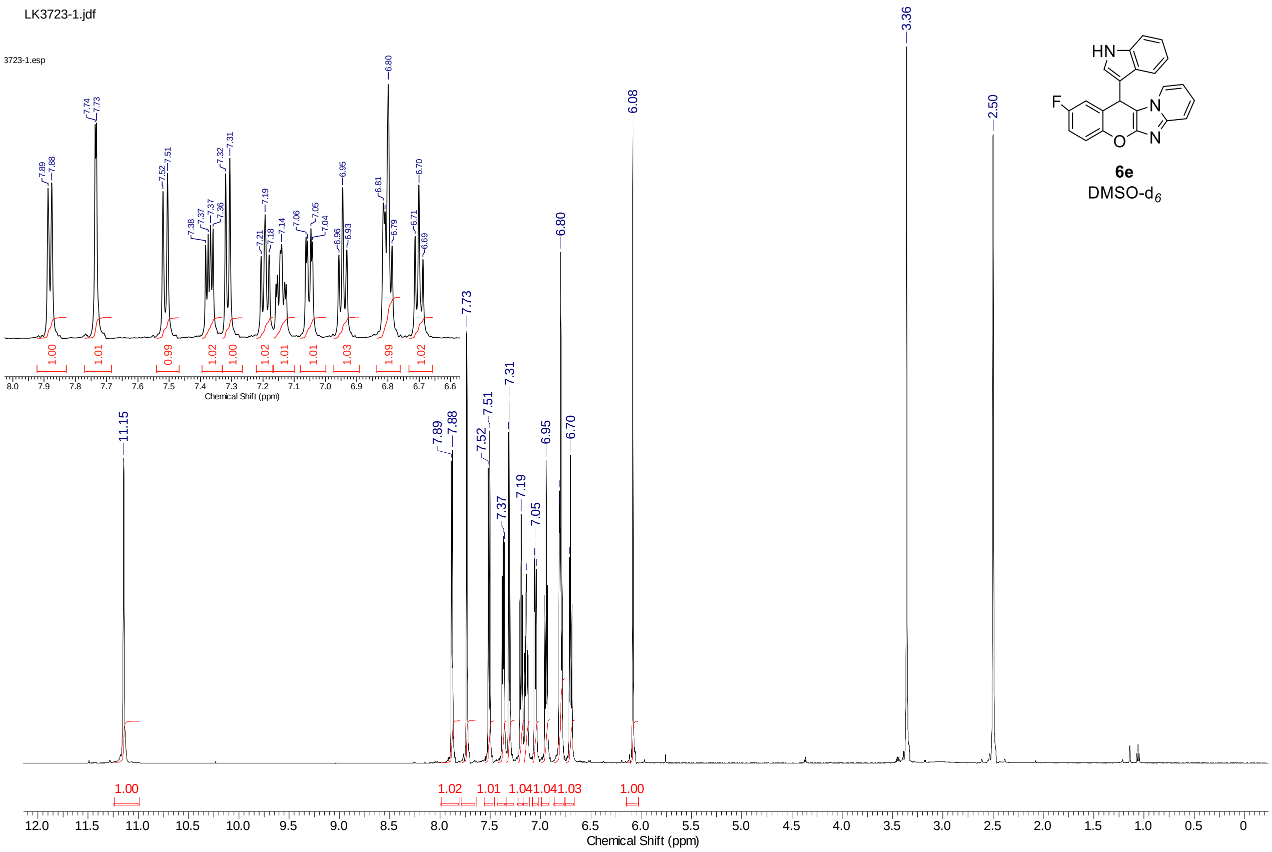
LK3723-1.jdf
3723-1.esp
HN
F
N
O
N
11.15
7.89
7.88
7.73
7.52
7.51
7.37
7.31
7.19
7.05
6.95
6.80
6.70
6.08
3.36
2.50
1.00
1.02
1.01
1.04
1.04
1.03
1.00
0.99
1.02
1.00
1.02
1.01
1.01
1.03
1.99
1.02
Chemical Shift (ppm)

6e
DMSO-d_6

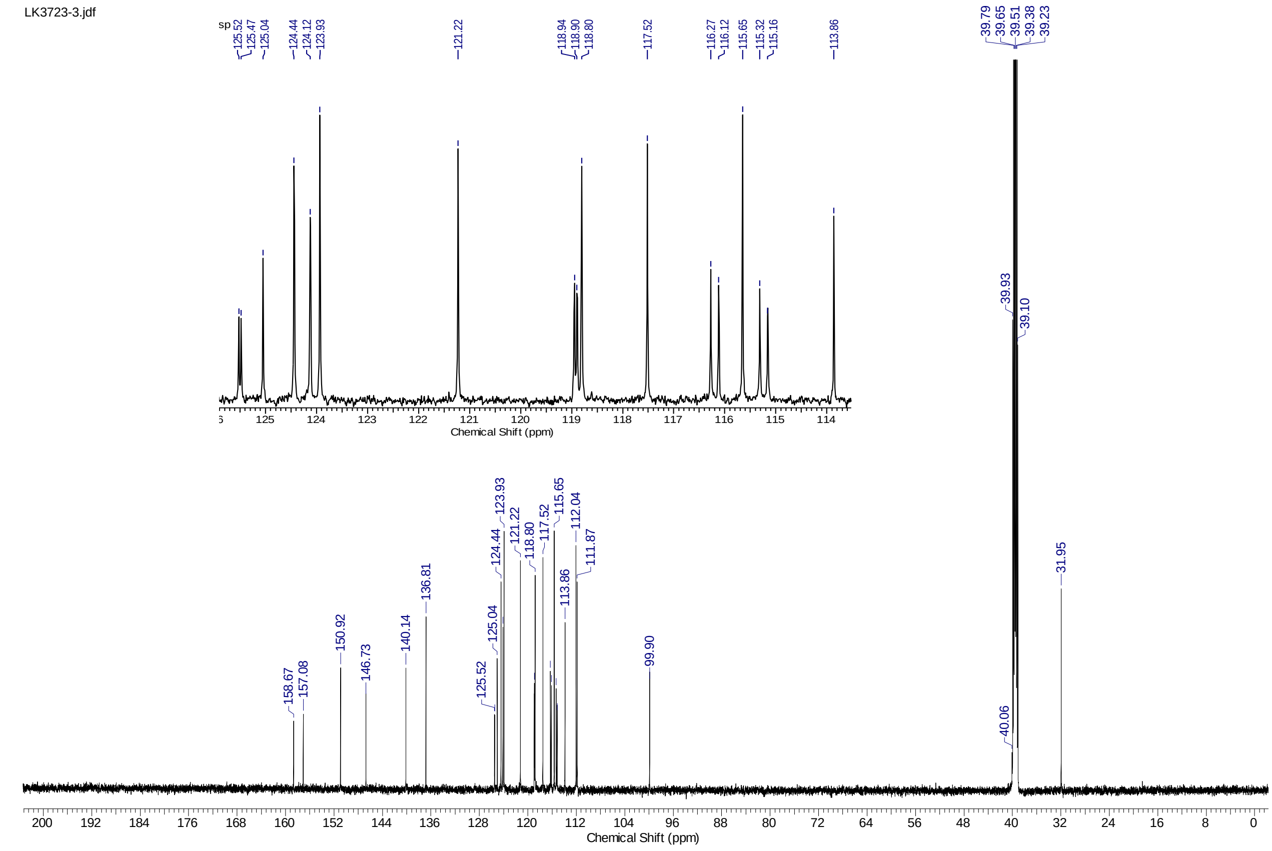
LK3723-3.jdf
158.67
157.08
150.92
146.73
140.14
136.81
125.52
125.04
124.44
123.93
121.22
118.80
117.52
115.65
113.86
112.04
111.87
99.90
40.06
39.93
39.10
31.95
125.52
125.47
125.04
124.44
124.12
123.93
121.22
118.94
118.90
118.80
117.52
116.27
116.12
115.65
115.32
115.16
113.86
39.79
39.65
39.51
39.38
39.23
Chemical Shift (ppm)

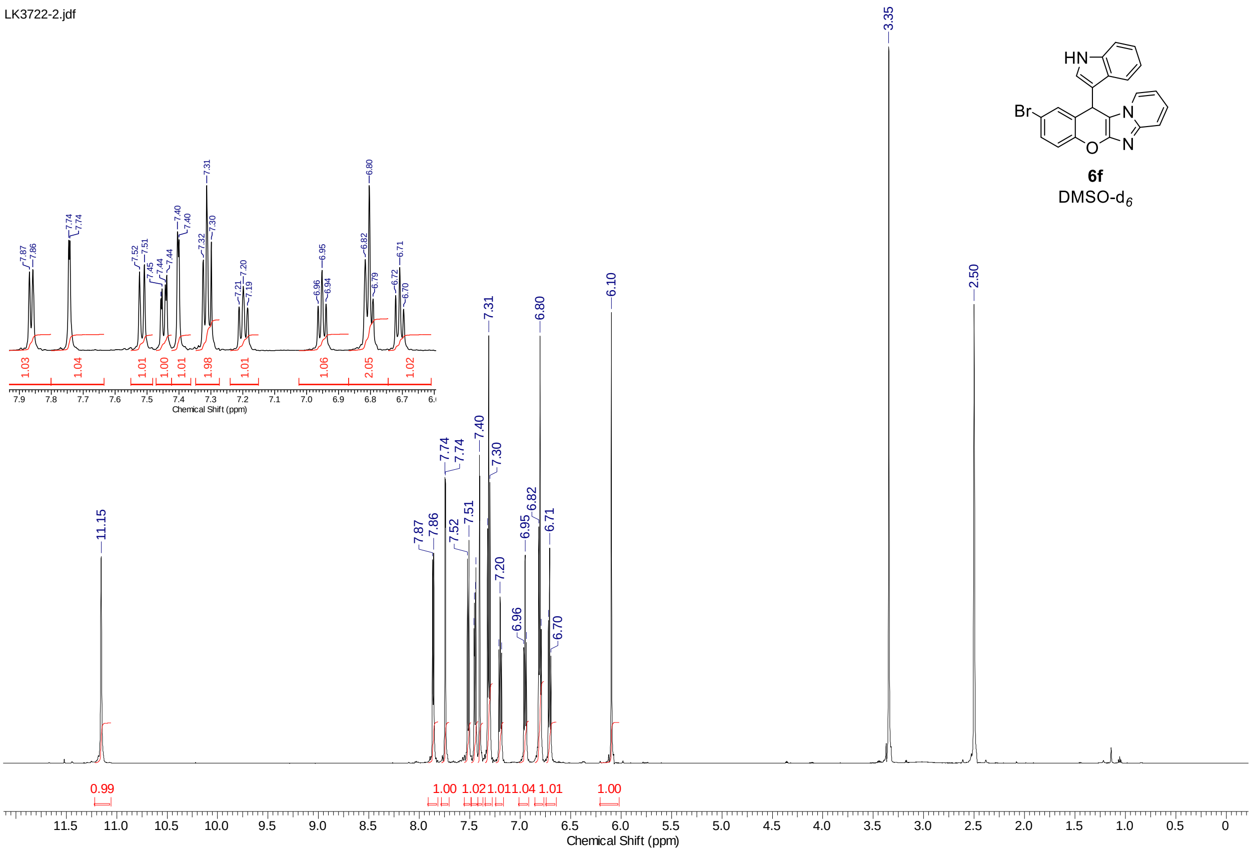
LK3722-2.jdf
HN
Br
N
O
N
6f
DMSO-d6
3.35
2.50
6.10
6.80
7.31
7.74
7.74
7.40
7.30
11.15
7.87
7.86
7.52
7.51
6.95
6.82
6.71
7.20
6.96
6.70
0.99
1.00
1.02
1.01
1.01
1.04
1.01
1.00
1.03
1.04
1.01
1.00
1.01
1.98
1.01
1.06
2.05
1.02
Chemical Shift (ppm)

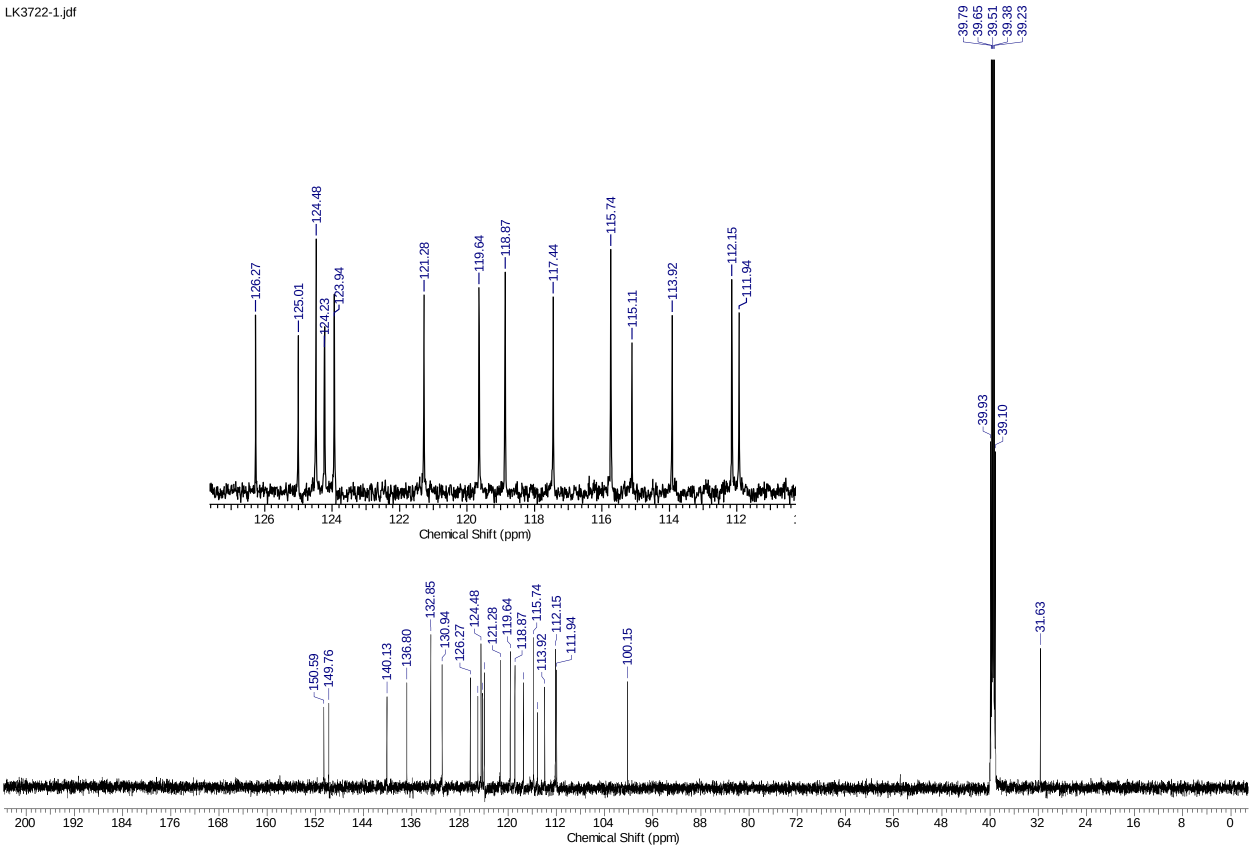
LK3722-1.jdf
39.79
39.65
39.51
39.38
39.23
39.93
39.10
126.27
125.01
124.48
124.23
123.94
121.28
119.64
118.87
117.44
115.74
115.11
113.92
112.15
111.94
150.59
149.76
140.13
136.80
132.85
130.94
100.15
31.63
Chemical Shift (ppm)

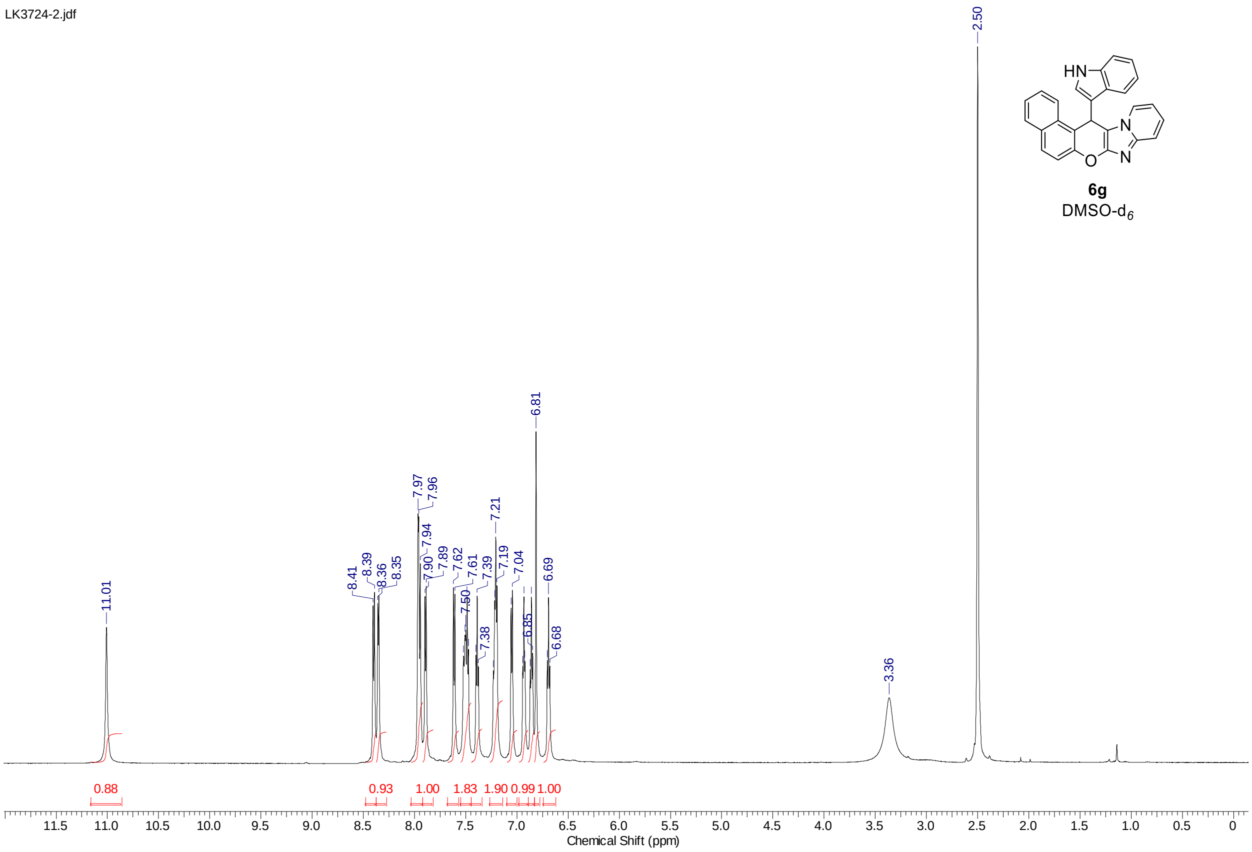
LK3724-2.jdf
2.50
11.01
8.41
8.39
8.36
8.35
7.97
7.96
7.94
7.90
7.89
7.62
7.61
7.50
7.39
7.38
7.21
7.19
7.04
6.85
6.81
6.69
6.68
3.36
0.88
0.93
1.00
1.83
1.90
0.99
1.00
Chemical Shift (ppm)

6g
DMSO-d_6

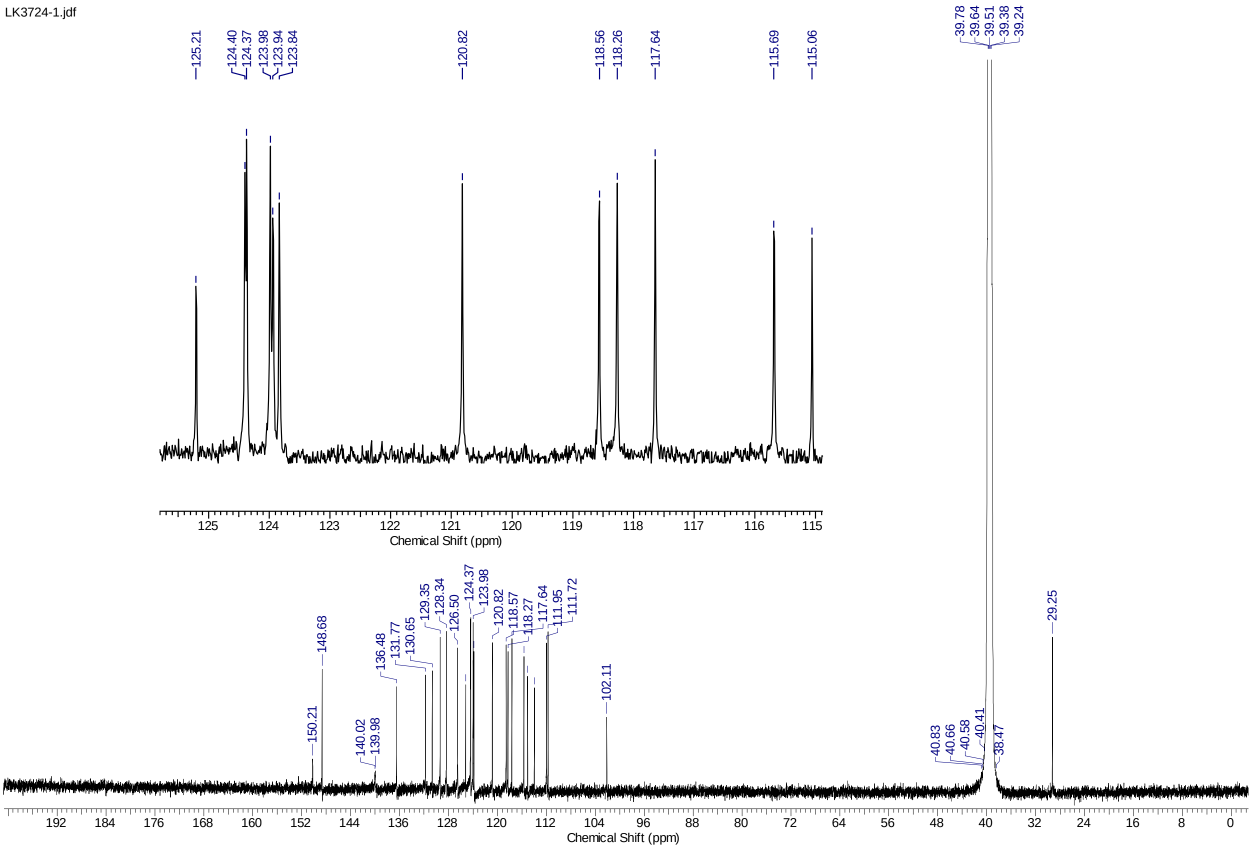
LK3724-1.jdf
125.21
124.40
124.37
123.98
123.94
123.84
120.82
118.56
118.26
117.64
115.69
115.06
39.78
39.64
39.51
39.38
39.24
Chemical Shift (ppm)
150.21
148.68
140.02
139.98
136.48
131.77
130.65
129.35
128.34
126.50
124.37
123.98
120.82
118.57
118.27
117.64
111.95
111.72
102.11
40.83
40.66
40.58
40.41
38.47
29.25
Chemical Shift (ppm)

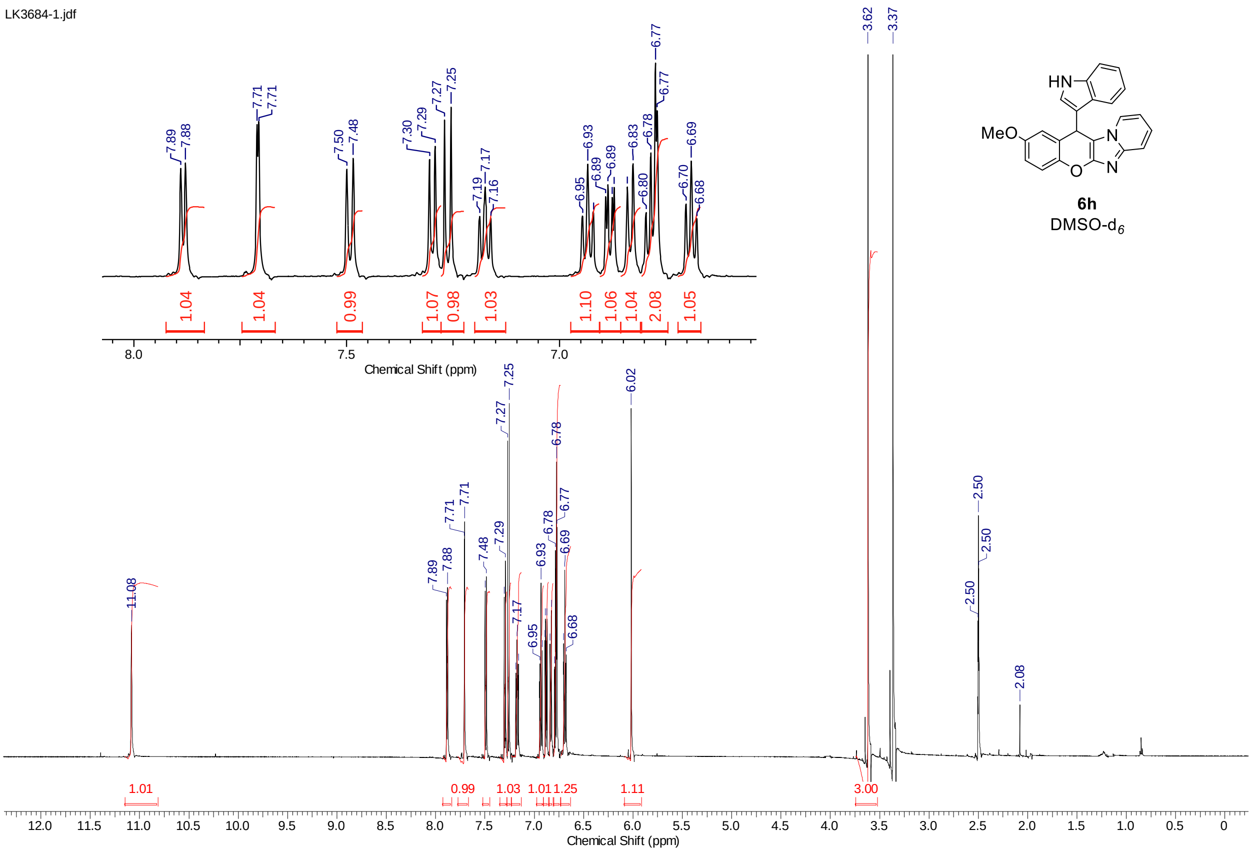
LK3684-1.jdf
7.89 7.88 7.71 7.71 7.50 7.48 7.30 7.29 7.27 7.25 7.19 7.17 7.16 6.95 6.93 6.89 6.89 6.83 6.80 6.78 6.77 6.77 6.70 6.69 6.68
1.04 1.04 0.99 1.07 0.98 1.03 1.10 1.06 1.04 2.08 1.05
Chemical Shift (ppm)
11.08 7.89 7.88 7.71 7.71 7.48 7.29 7.27 7.25 7.17 6.95 6.93 6.78 6.78 6.77 6.69 6.68 6.02 3.62 3.37 2.50 2.50 2.50 2.08
1.01 0.99 1.03 1.01 1.25 1.11 3.00
Chemical Shift (ppm)
HN
MeO
N
O
N

6h
DMSO-d_6

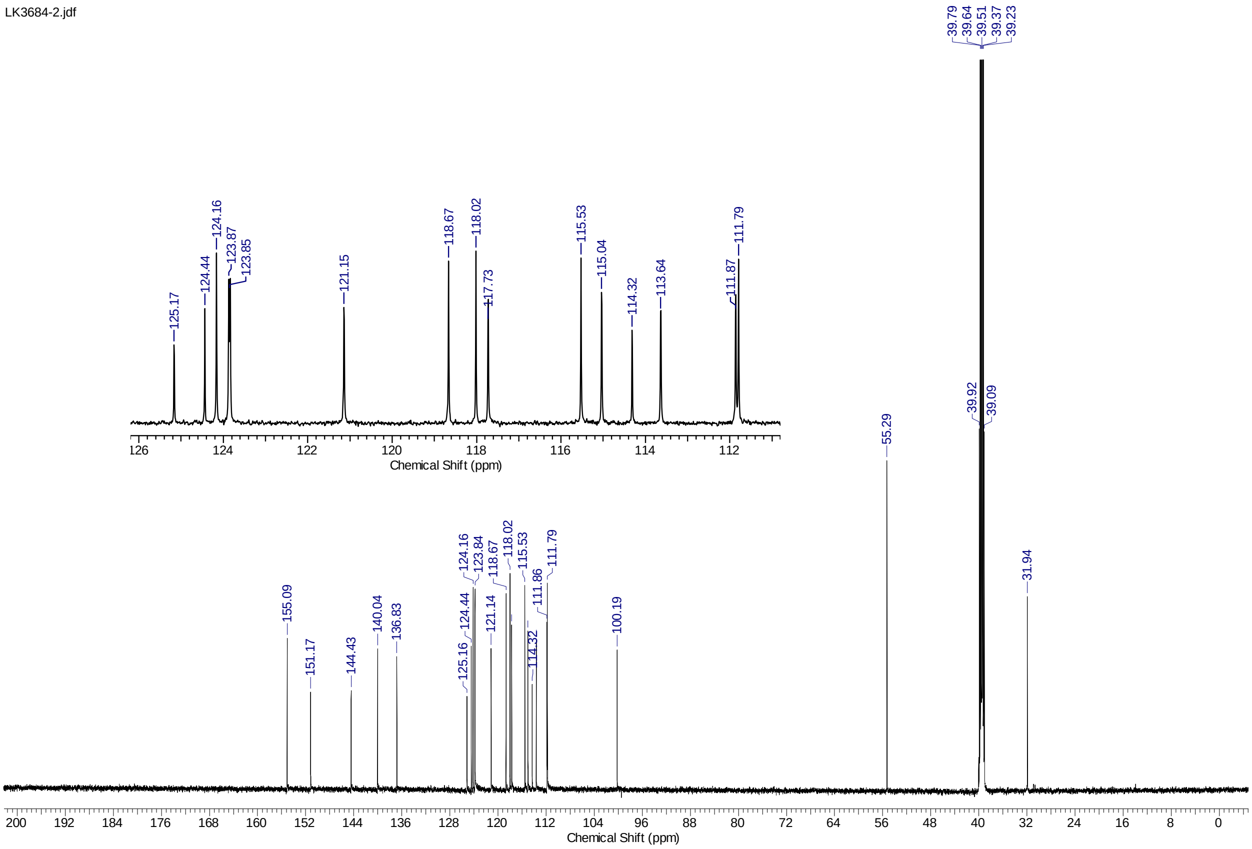
LK3684-2.jdf
125.17 124.44 124.16 123.87 123.85 121.15 118.67 118.02 117.73 115.53 115.04 114.32 113.64 111.87 111.79
Chemical Shift (ppm)
155.09 151.17 144.43 140.04 136.83 125.16 124.44 124.16 123.84 121.14 118.67 118.02 115.53 114.32 111.86 111.79 100.19 55.29 39.92 39.79 39.64 39.51 39.37 39.23 39.09 31.94
Chemical Shift (ppm)

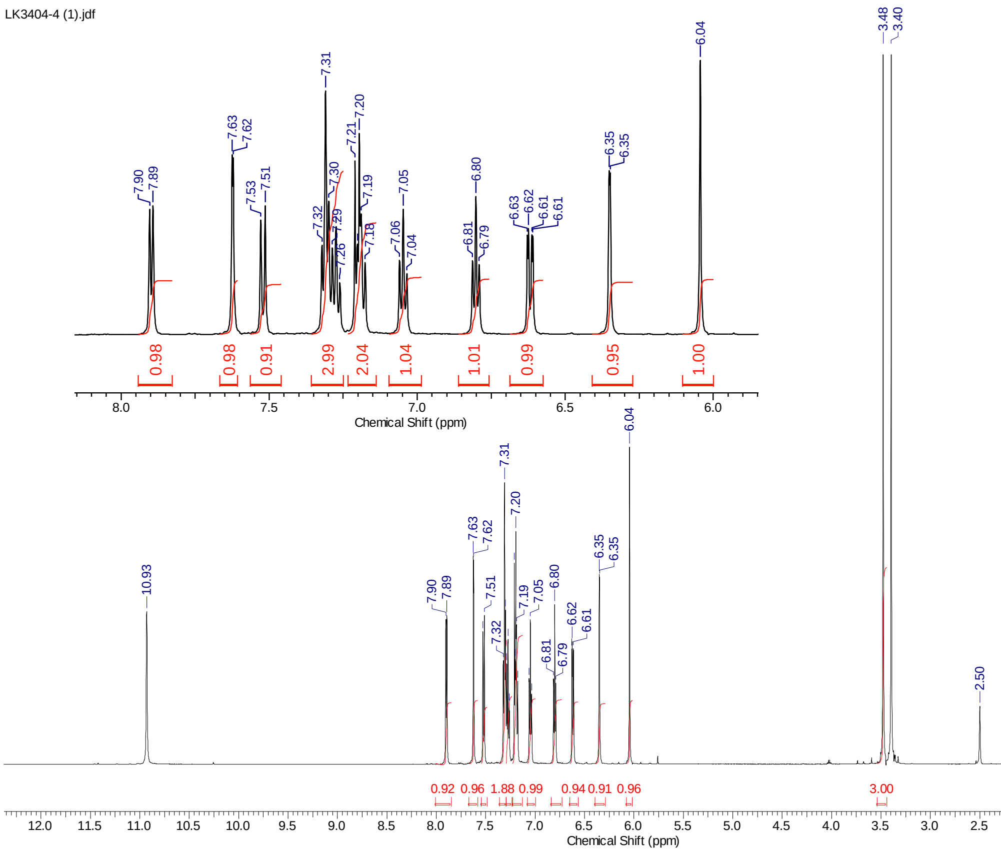
LK3404-4 (1).jdf
Chemical Shift (ppm)

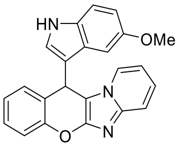
HN
OMe
N
N
O

6i
DMSO-d_6

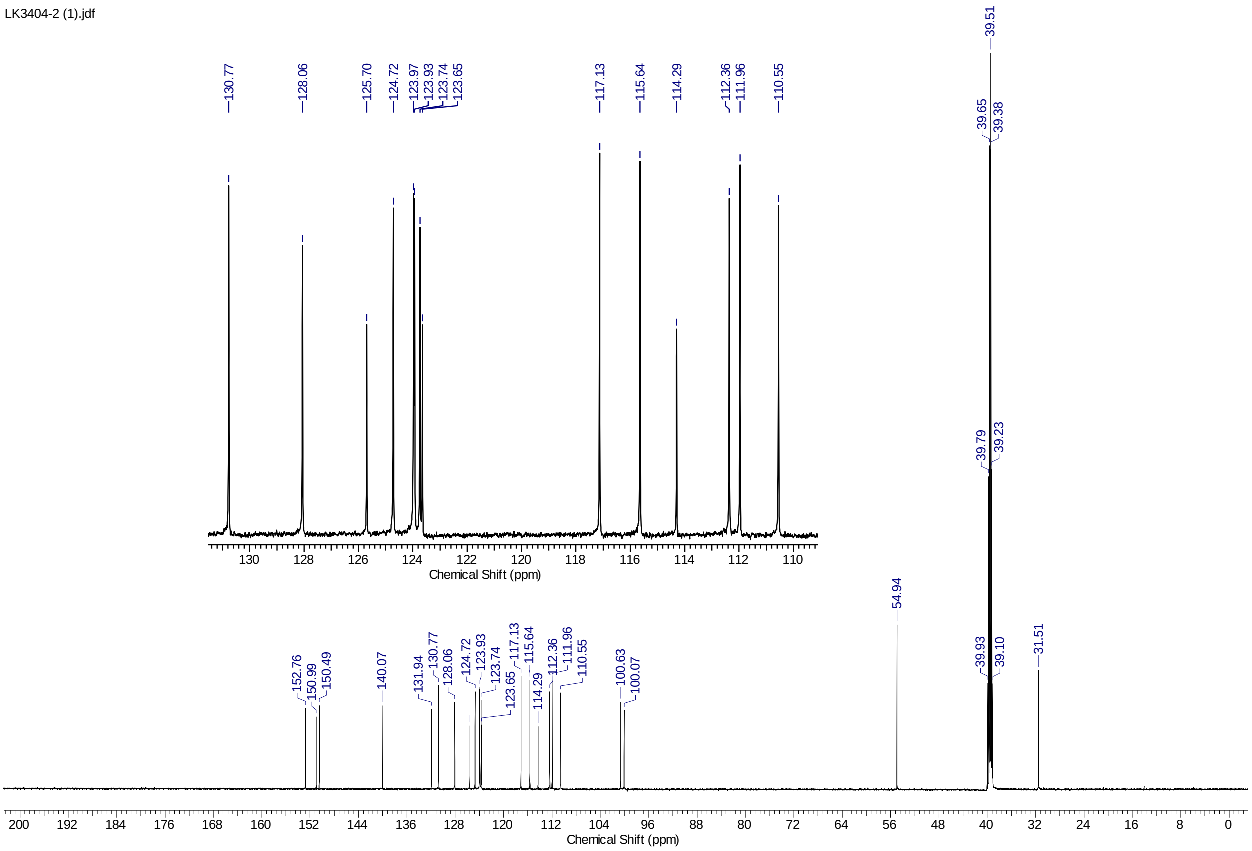
LK3404-2 (1).jdf
Chemical Shift (ppm)

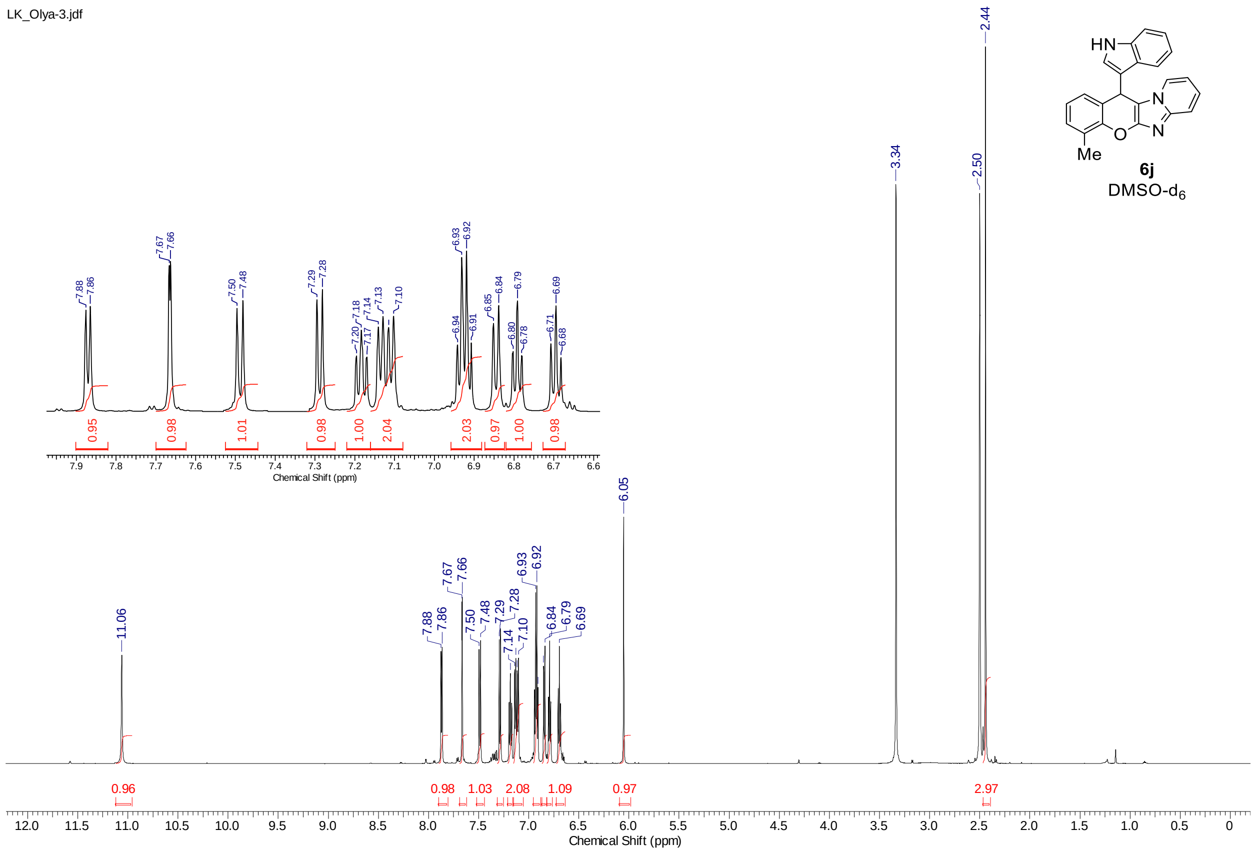
LK_Olya-3.jdf
HN
N
N
O
Me
6j
DMSO-d_6
11.06
7.88
7.86
7.67
7.66
7.50
7.48
7.29
7.28
7.14
7.10
6.93
6.92
6.84
6.79
6.69
6.05
3.34
2.50
2.44
0.96
0.98
1.03
2.08
1.09
0.97
2.97
0.95
1.01
1.00
2.04
2.03
0.97
Chemical Shift (ppm)

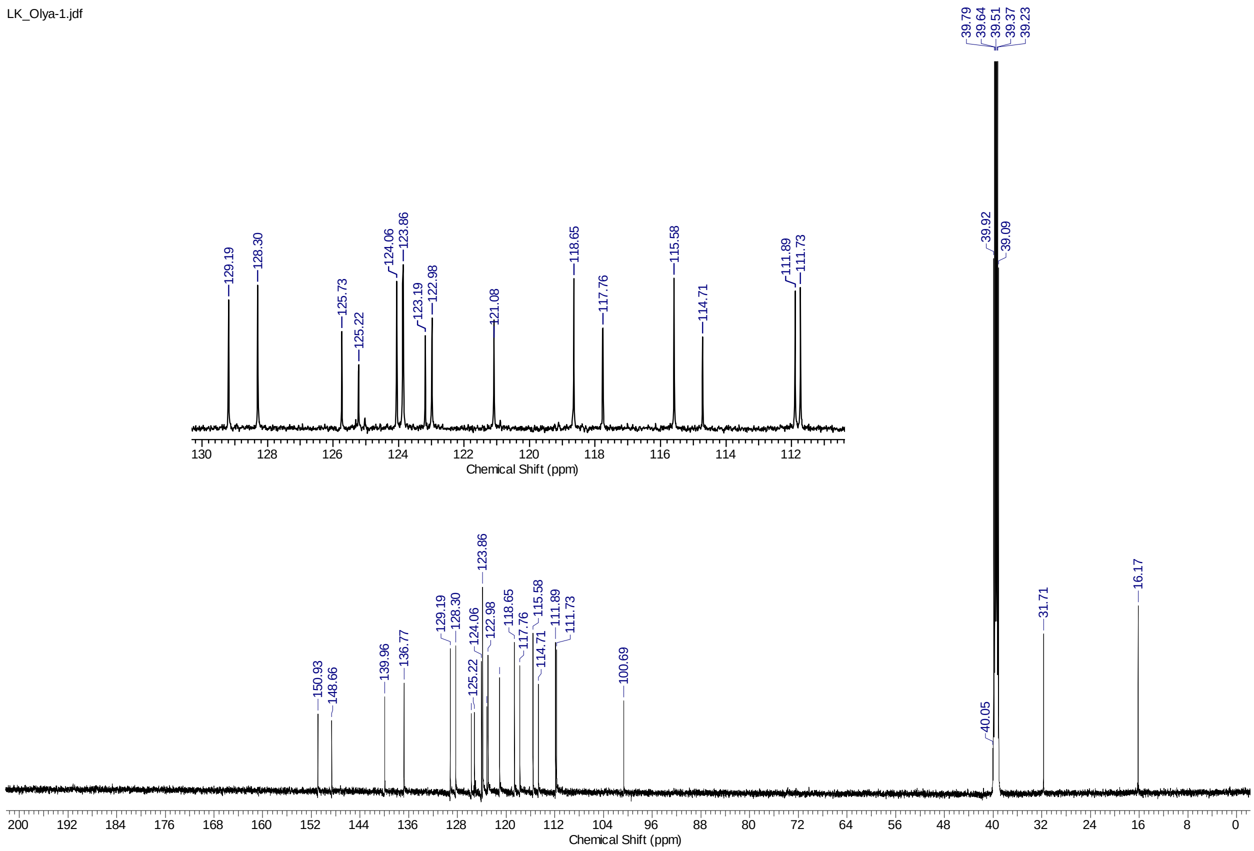
LK_Olya-1.jdf
150.93
148.66
139.96
136.77
129.19
128.30
125.73
125.22
124.06
123.86
123.19
122.98
121.08
118.65
117.76
115.58
114.71
111.89
111.73
100.69
40.05
39.92
39.79
39.64
39.51
39.37
39.23
39.09
31.71
16.17
Chemical Shift (ppm)

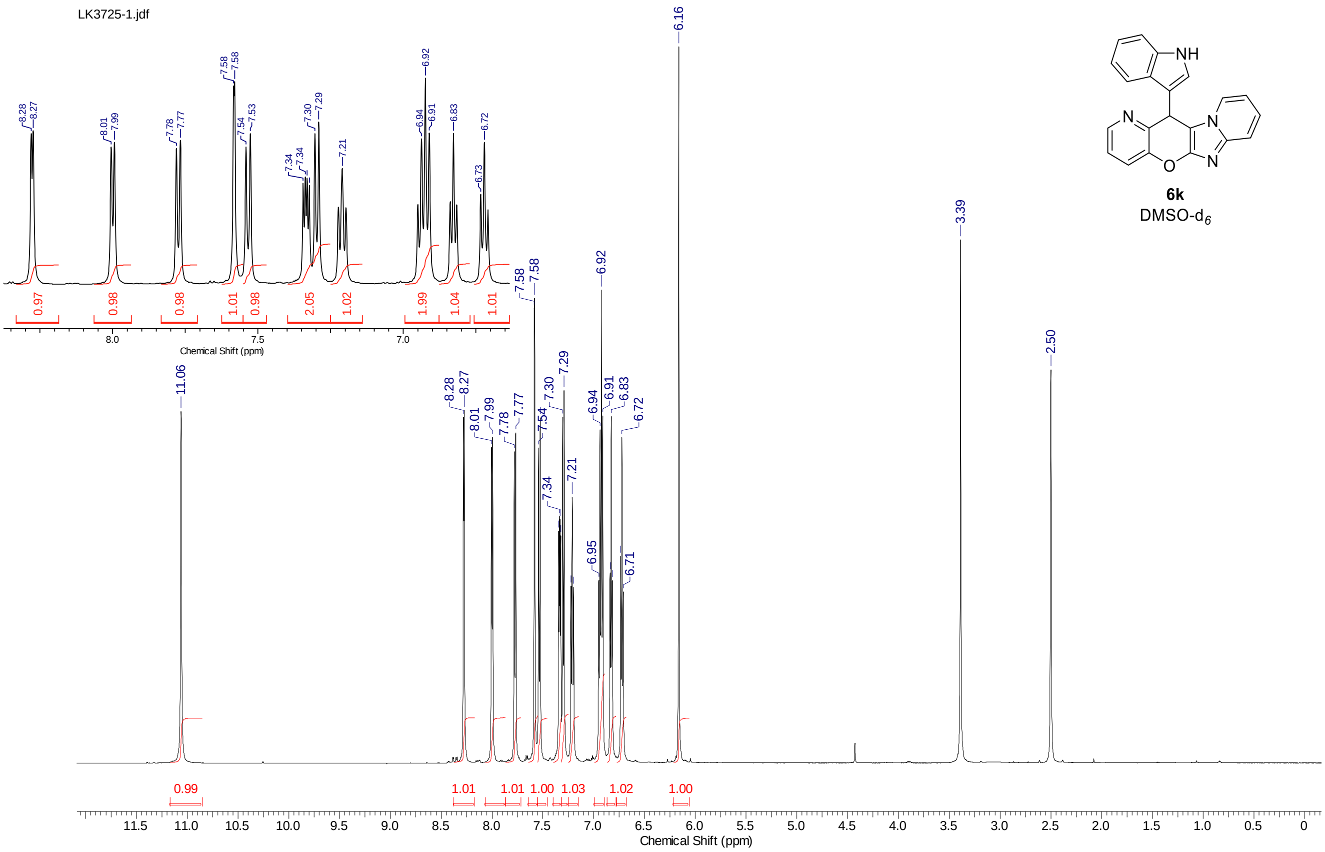
LK3725-1.jdf
11.06
8.28
8.27
8.01
7.99
7.78
7.77
7.58
7.58
7.54
7.34
7.30
7.29
7.21
6.95
6.94
6.92
6.91
6.83
6.72
6.71
6.16
3.39
2.50
0.99
1.01
1.01
1.00
1.03
1.02
1.00
0.97
0.98
0.98
1.01
0.98
2.05
1.02
1.99
1.04
1.01
Chemical Shift (ppm)
NH
N
N
O
N

6k
DMSO-d_6

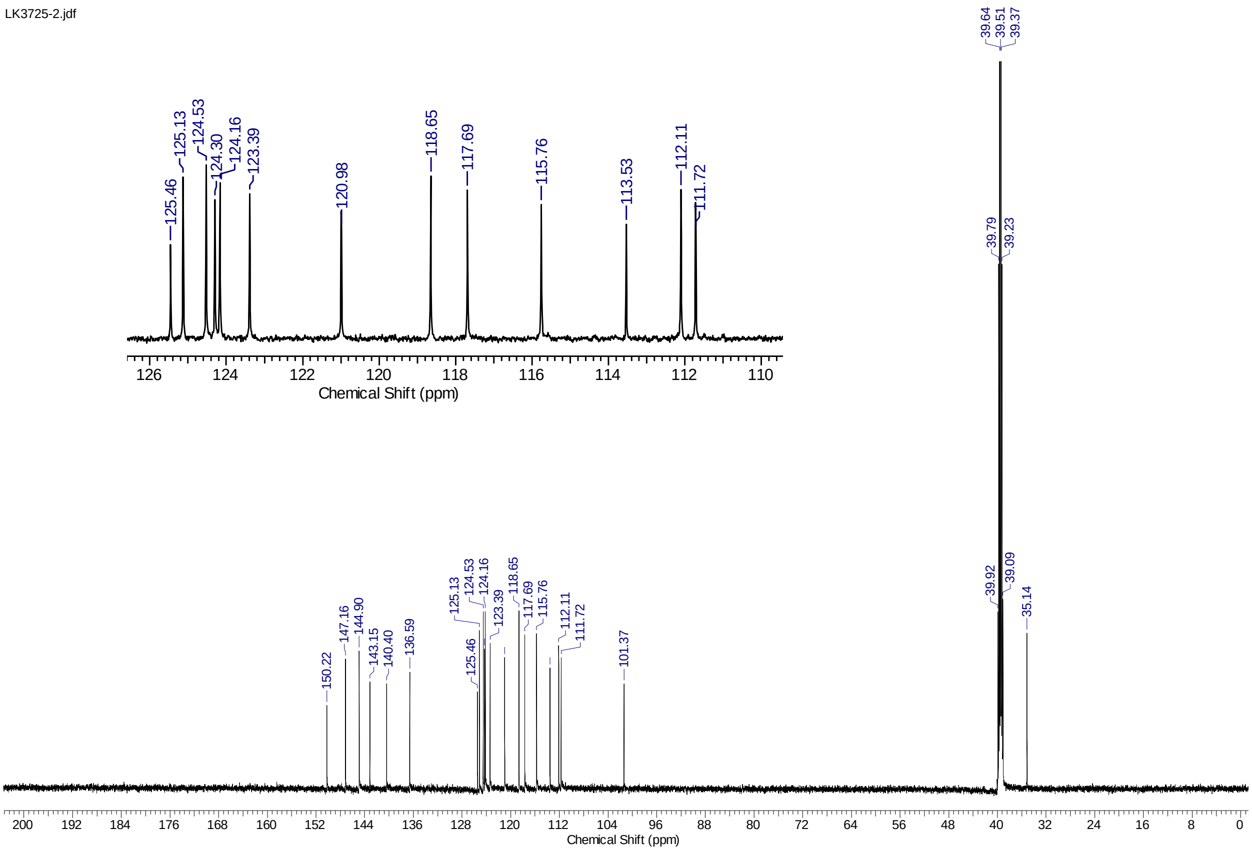
LK3725-2.jdf
150.22
147.16
144.90
143.15
140.40
136.59
125.46
125.13
124.53
124.30
124.16
123.39
120.98
118.65
117.69
115.76
113.53
112.11
111.72
101.37
39.92
39.79
39.64
39.51
39.37
39.23
39.09
35.14
Chemical Shift (ppm)

LK3316-1.jdf

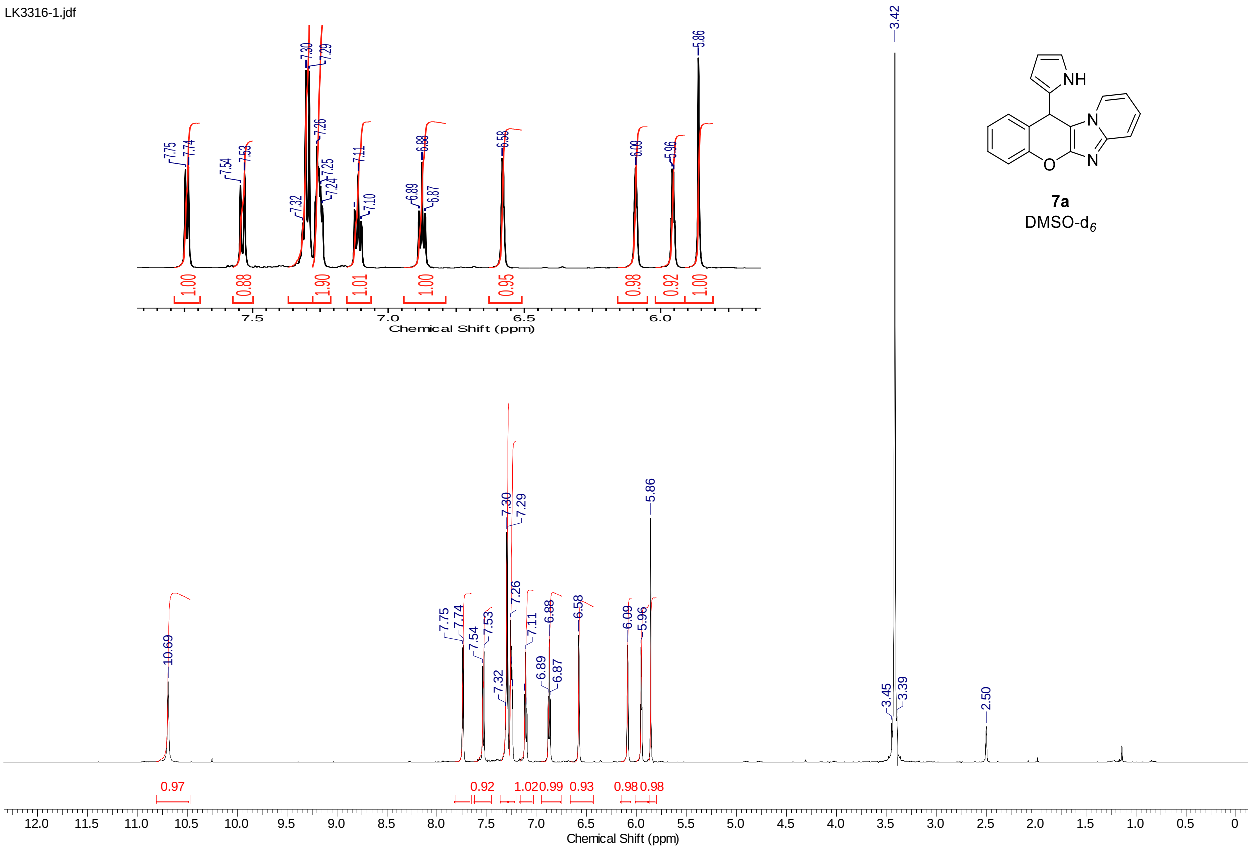

LK3333-2.jdf

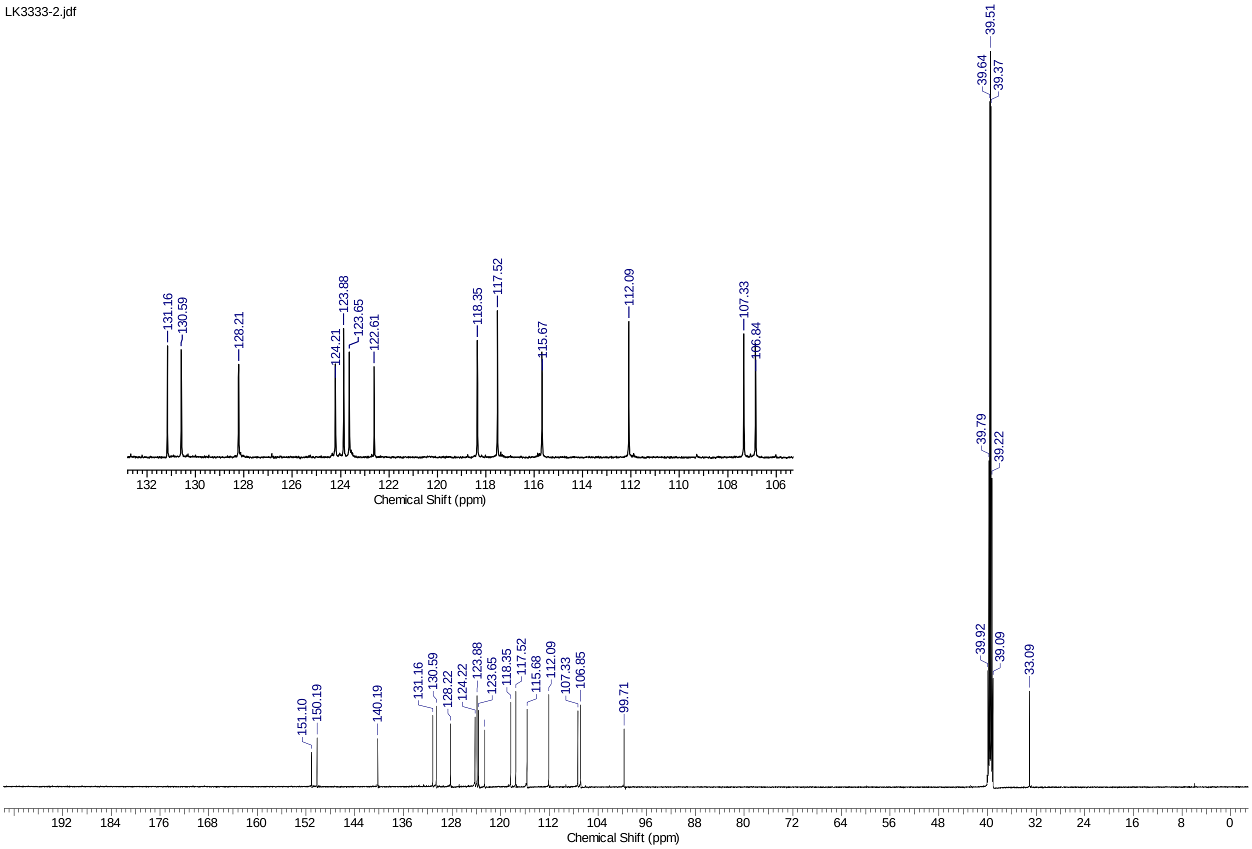

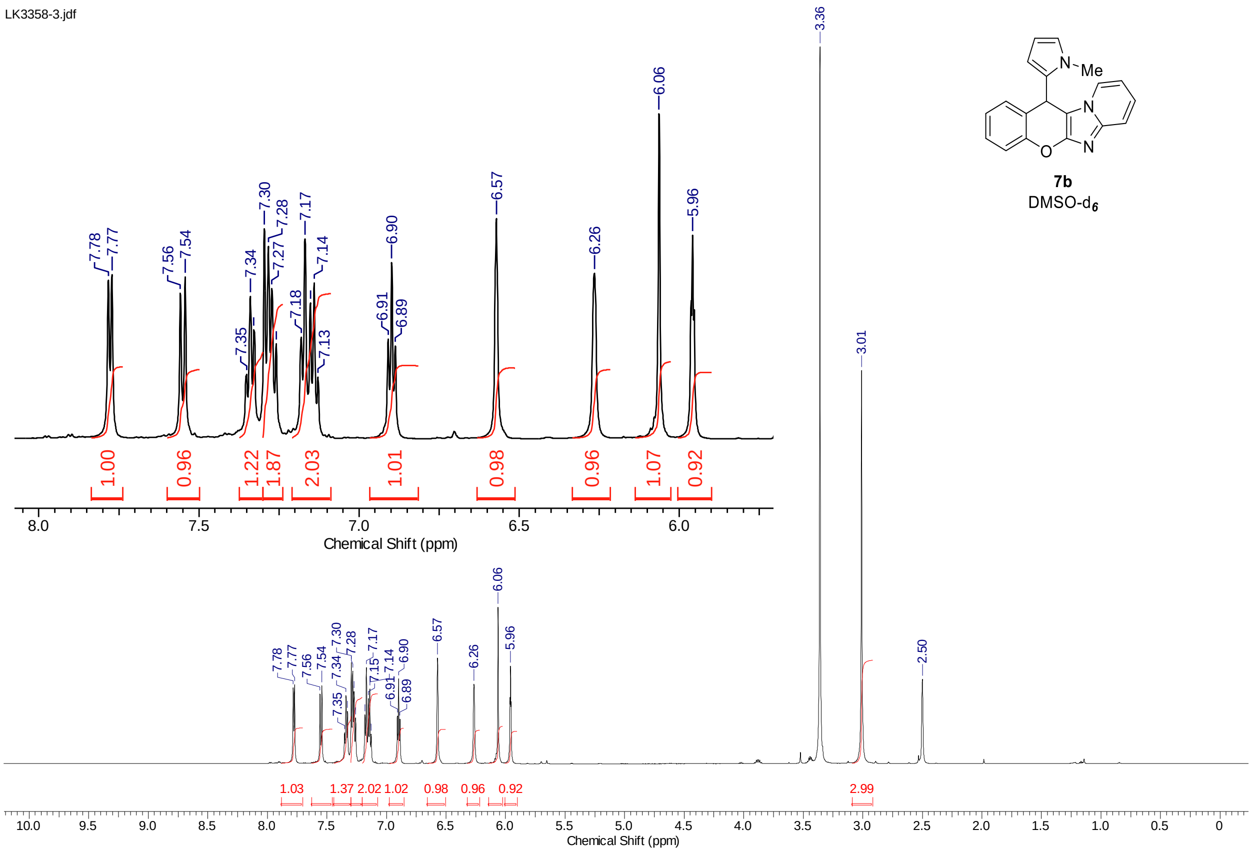
LK3358-3.jdf
Chemical Shift (ppm)
Me

7b
DMSO-d$_6$

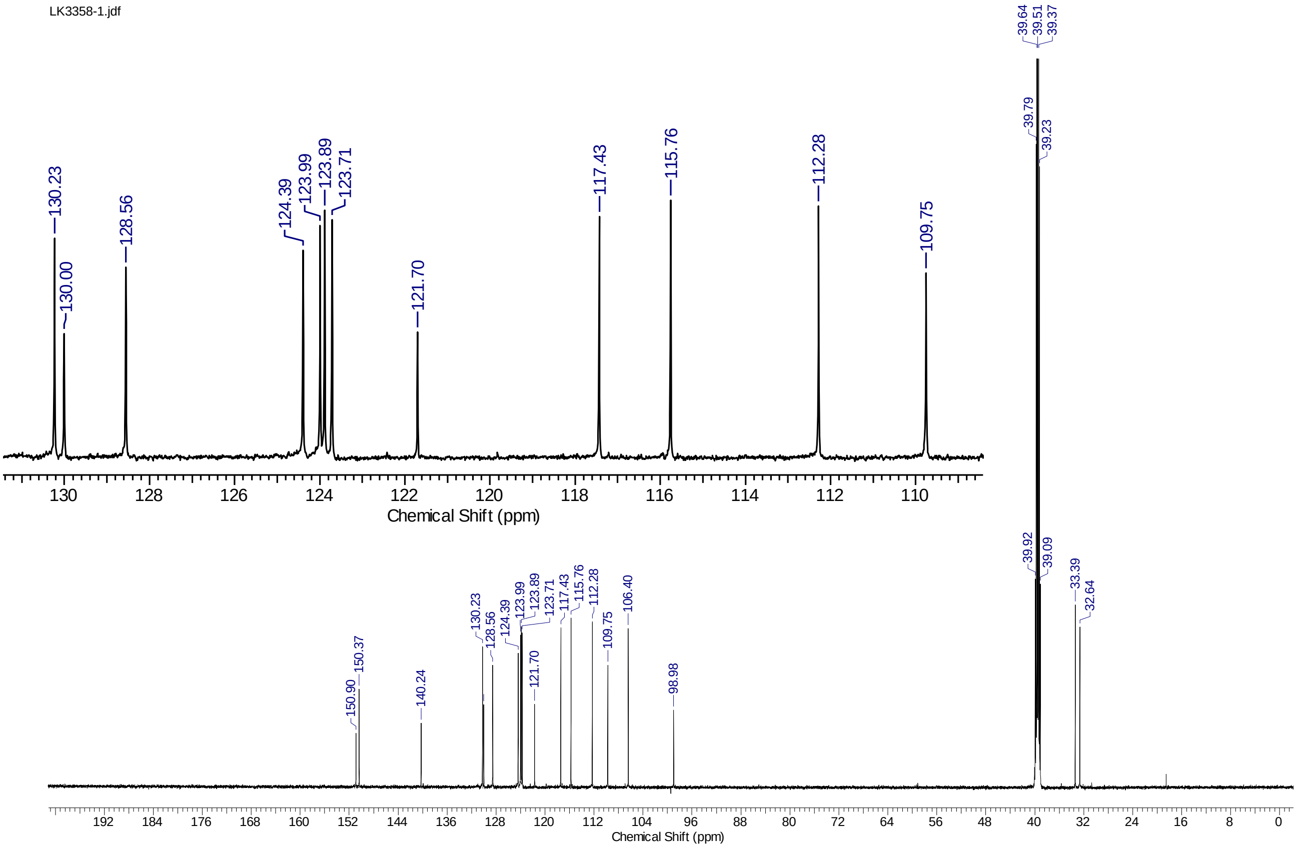
LK3358-1.jdf
Chemical Shift (ppm)

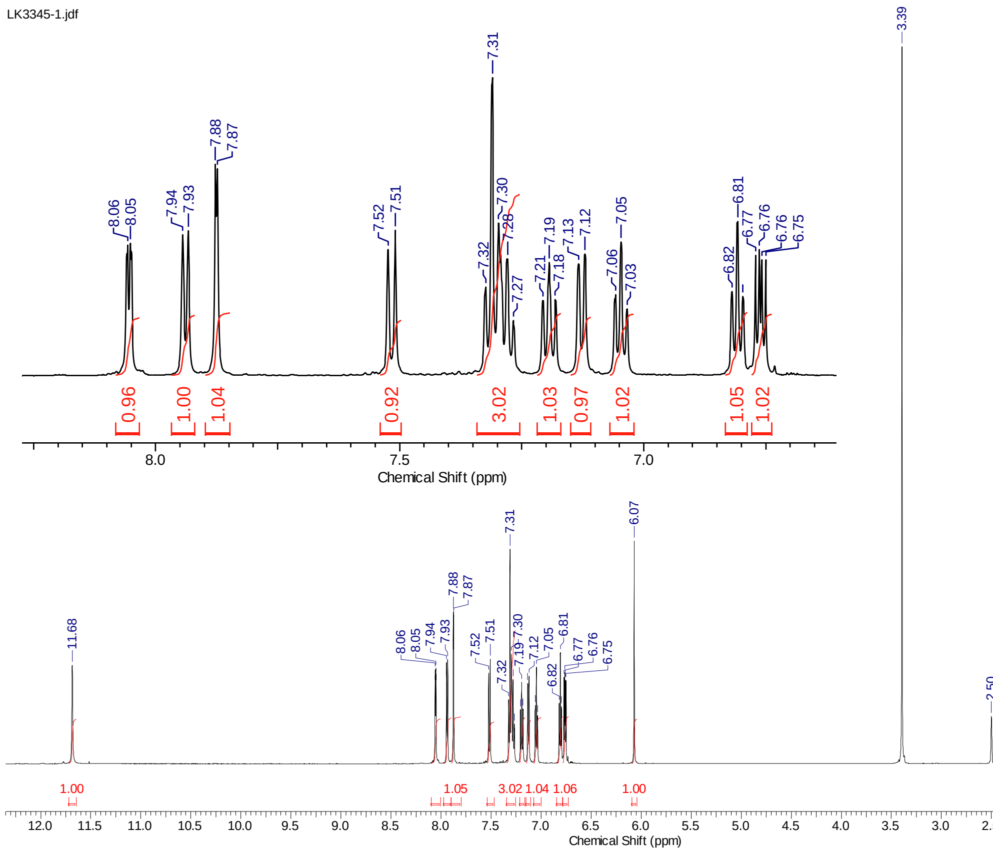
LK3345-1.jdf
Chemical Shift (ppm)

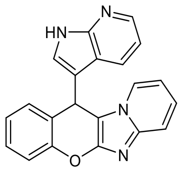

8
DMSO-d_6

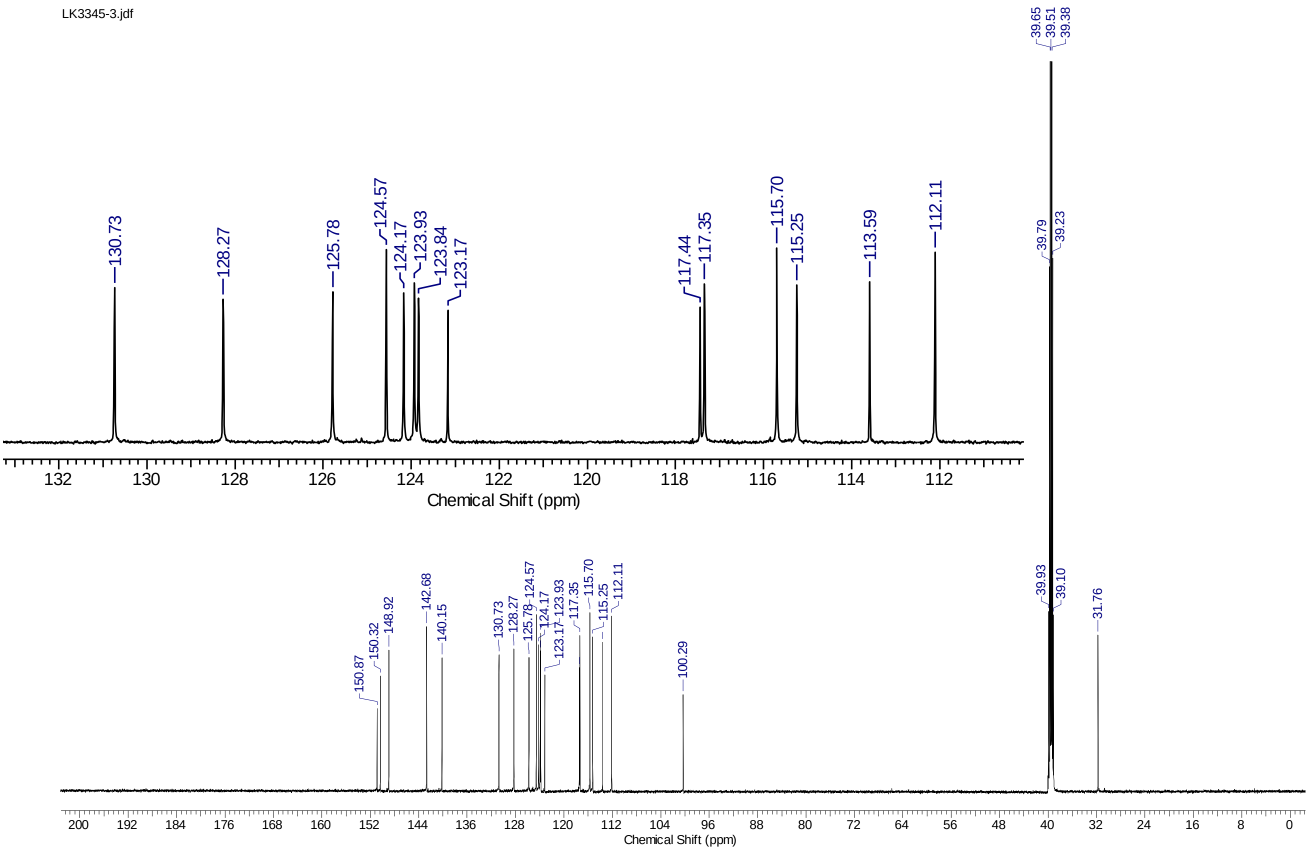
LK3345-3.jdf
Chemical Shift (ppm)

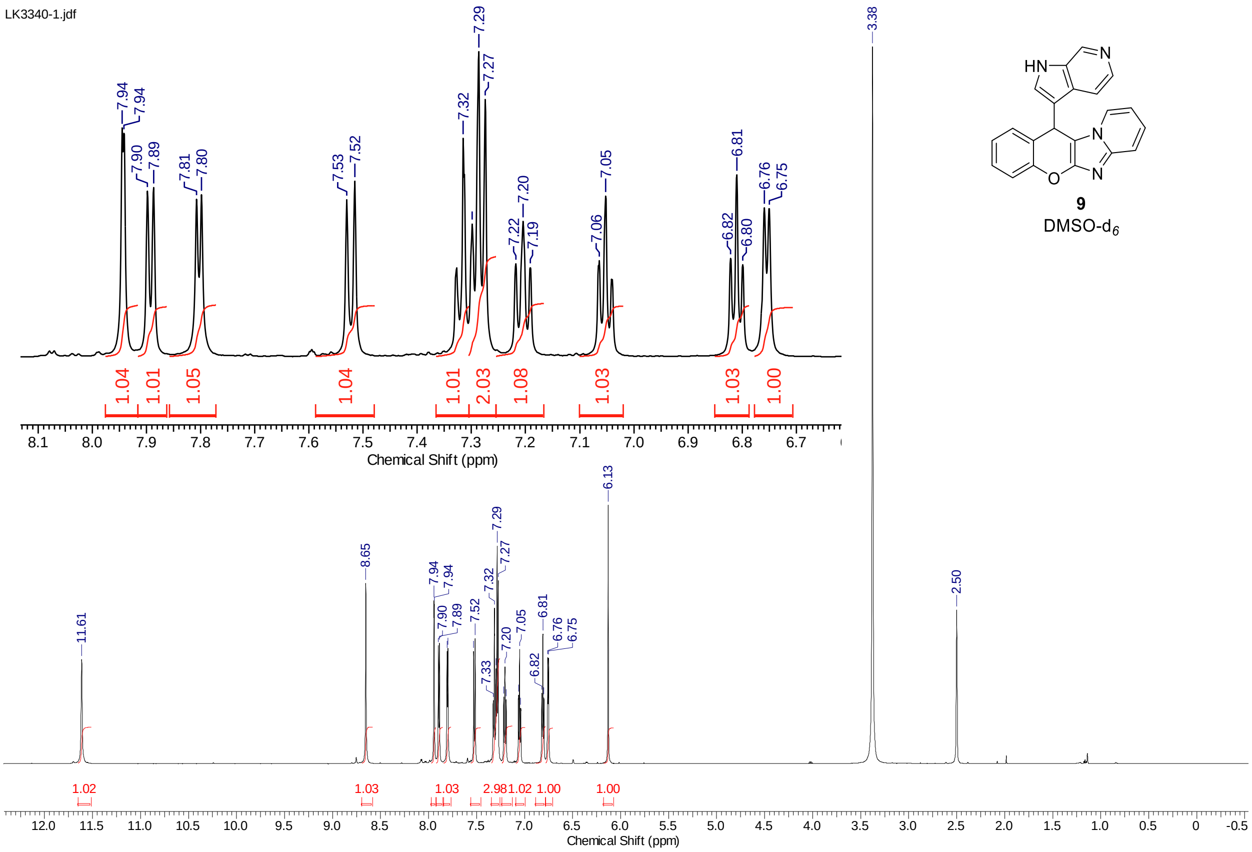
LK3340-1.jdf
7.94 7.94 7.90 7.89 7.81 7.80 7.53 7.52 7.32 7.29 7.27 7.22 7.20 7.19 7.06 7.05 6.82 6.81 6.80 6.76 6.75
1.04 1.01 1.05 1.04 1.01 2.03 1.08 1.03 1.03 1.00
Chemical Shift (ppm)
11.61 8.65 7.94 7.94 7.90 7.89 7.52 7.33 7.32 7.29 7.27 7.20 7.05 6.82 6.81 6.76 6.75 6.13 3.38 2.50
1.02 1.03 1.03 2.98 1.02 1.00 1.00
Chemical Shift (ppm)
HN
N
N
N
O
9
DMSO-d6

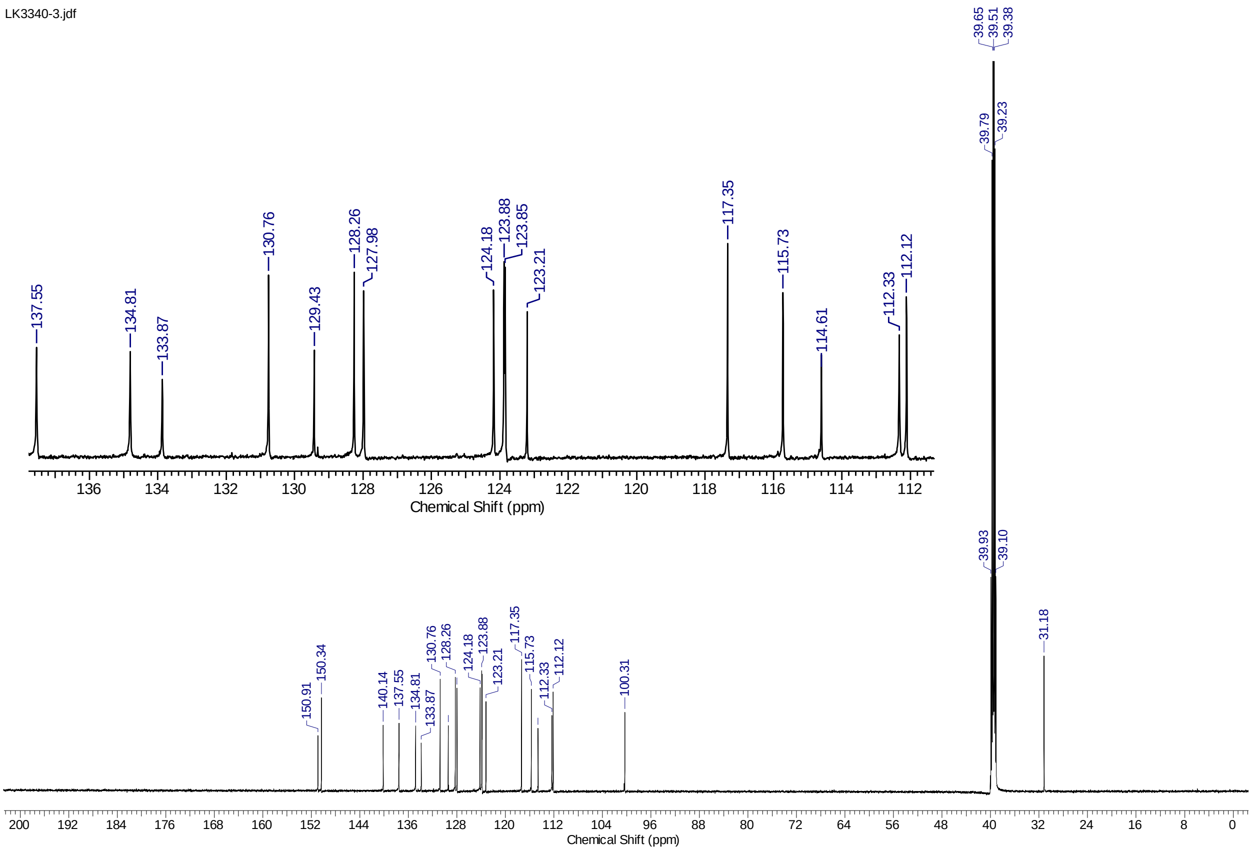
LK3340-3.jdf
39.65 39.51 39.38 39.79 39.23
137.55 134.81 133.87 130.76 129.43 128.26 127.98 124.18 123.88 123.85 123.21 117.35 115.73 114.61 112.33 112.12
Chemical Shift (ppm)
39.93 39.10
150.91 150.34 140.14 137.55 134.81 133.87 130.76 128.26 124.18 123.88 123.21 117.35 115.73 112.33 112.12 100.31 31.18
Chemical Shift (ppm)

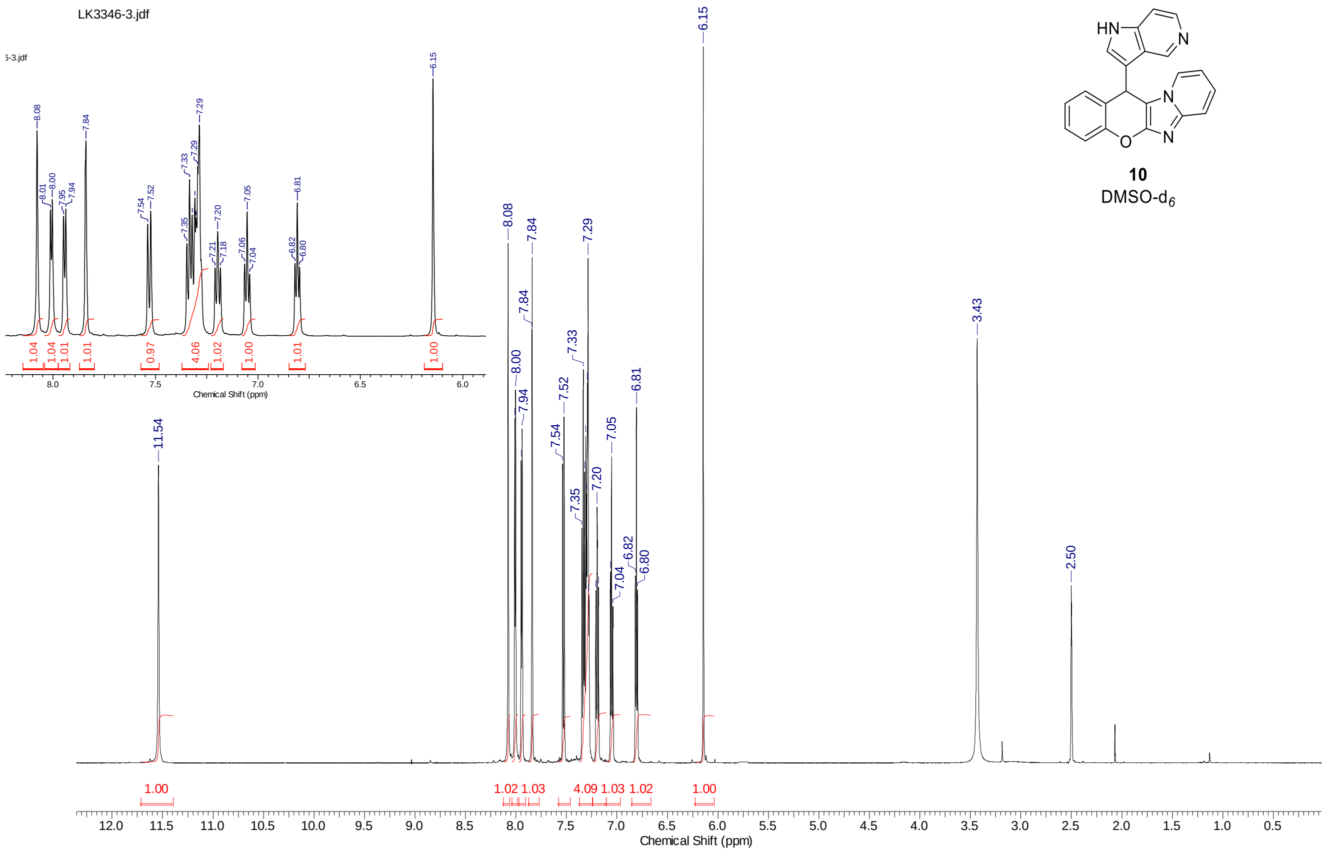

10
DMSO-d6
LK3346-3.jdf
Chemical Shift (ppm)

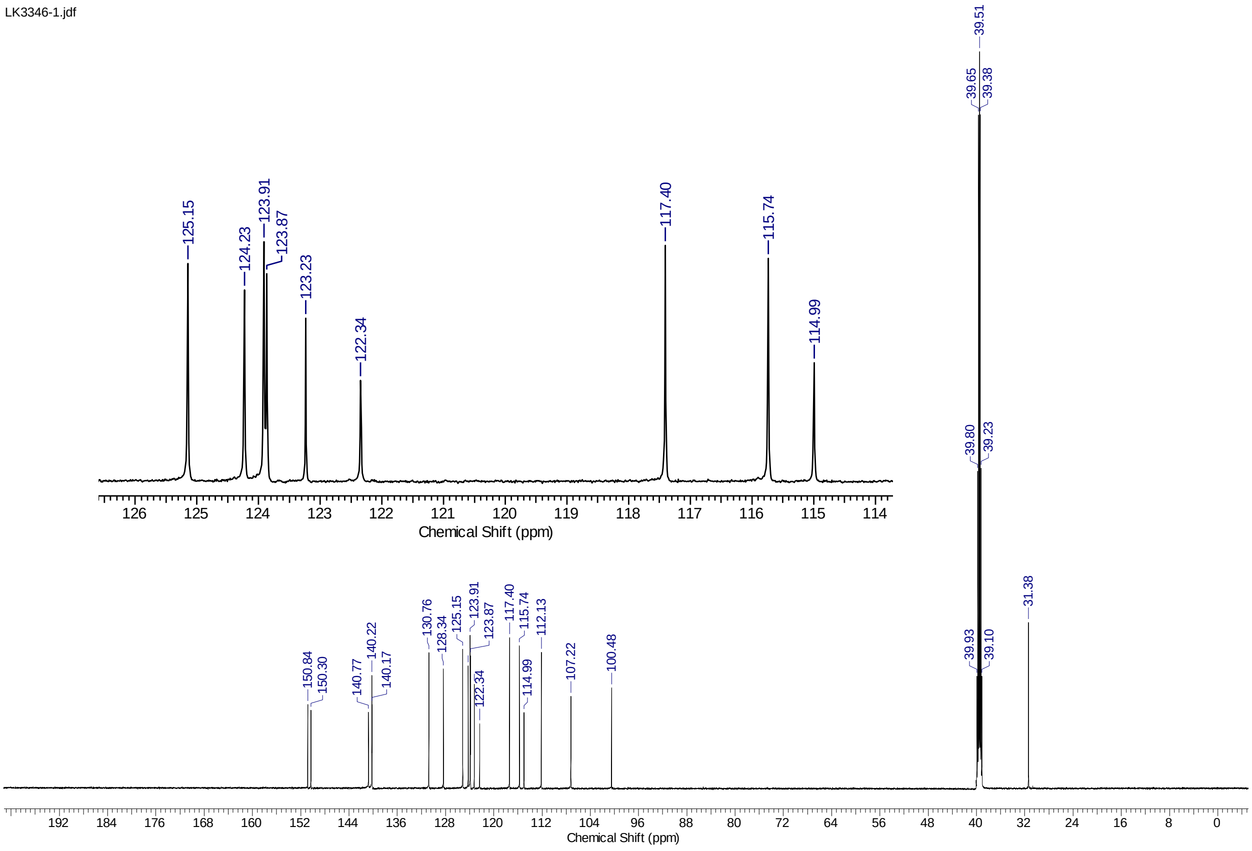

LK3346-1.jdf
Chemical Shift (ppm)

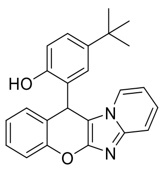
HO
N
N
O

11c
DMSO-d_6

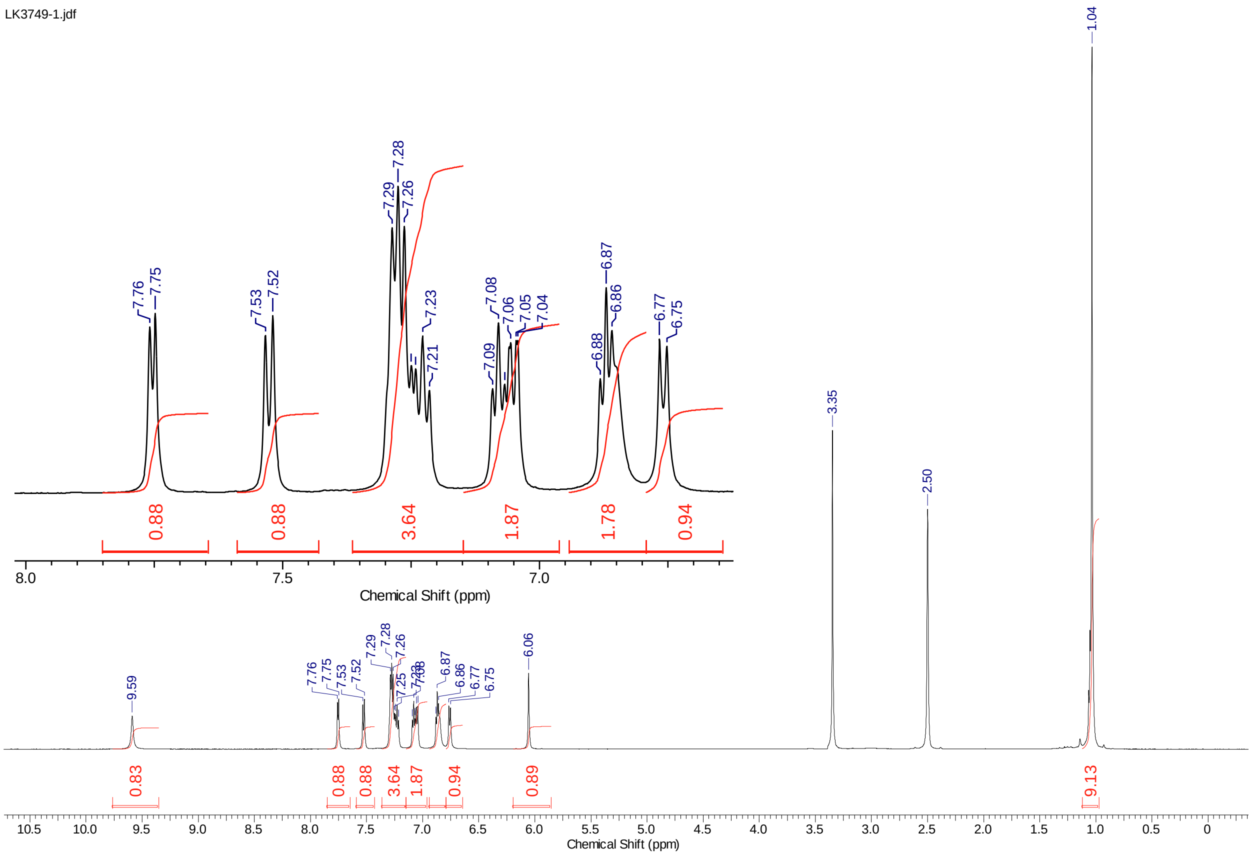
LK3749-1.jdf
Chemical Shift (ppm)
9.59
6.06
3.35
2.50
1.04
0.83
0.88
0.88
3.64
1.87
1.78
0.94
0.89
9.13

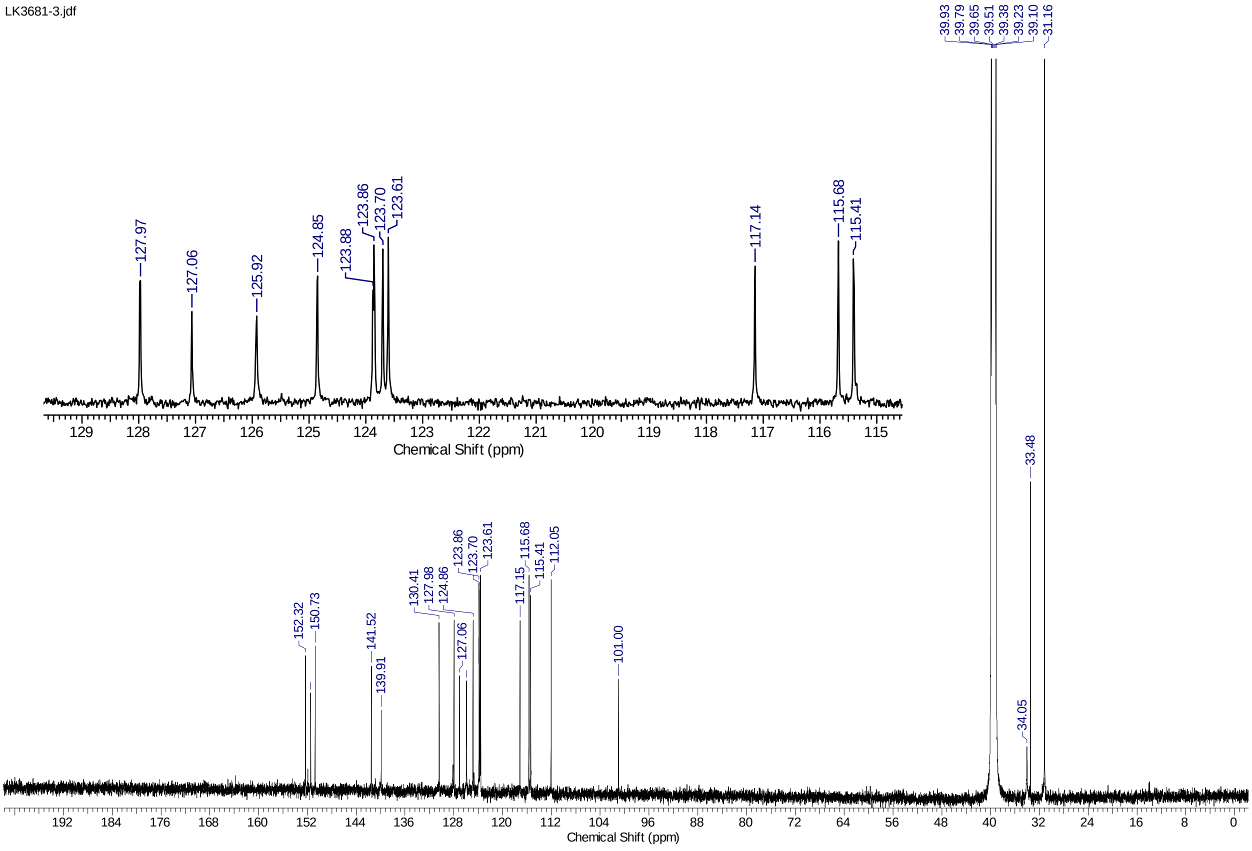
LK3681-3.jdf
Chemical Shift (ppm)
152.32
150.73
141.52
139.91
130.41
127.98
124.86
127.06
123.86
123.70
123.61
117.15
115.68
115.41
112.05
101.00
34.05
33.48
31.16

LK3675-1.jdf

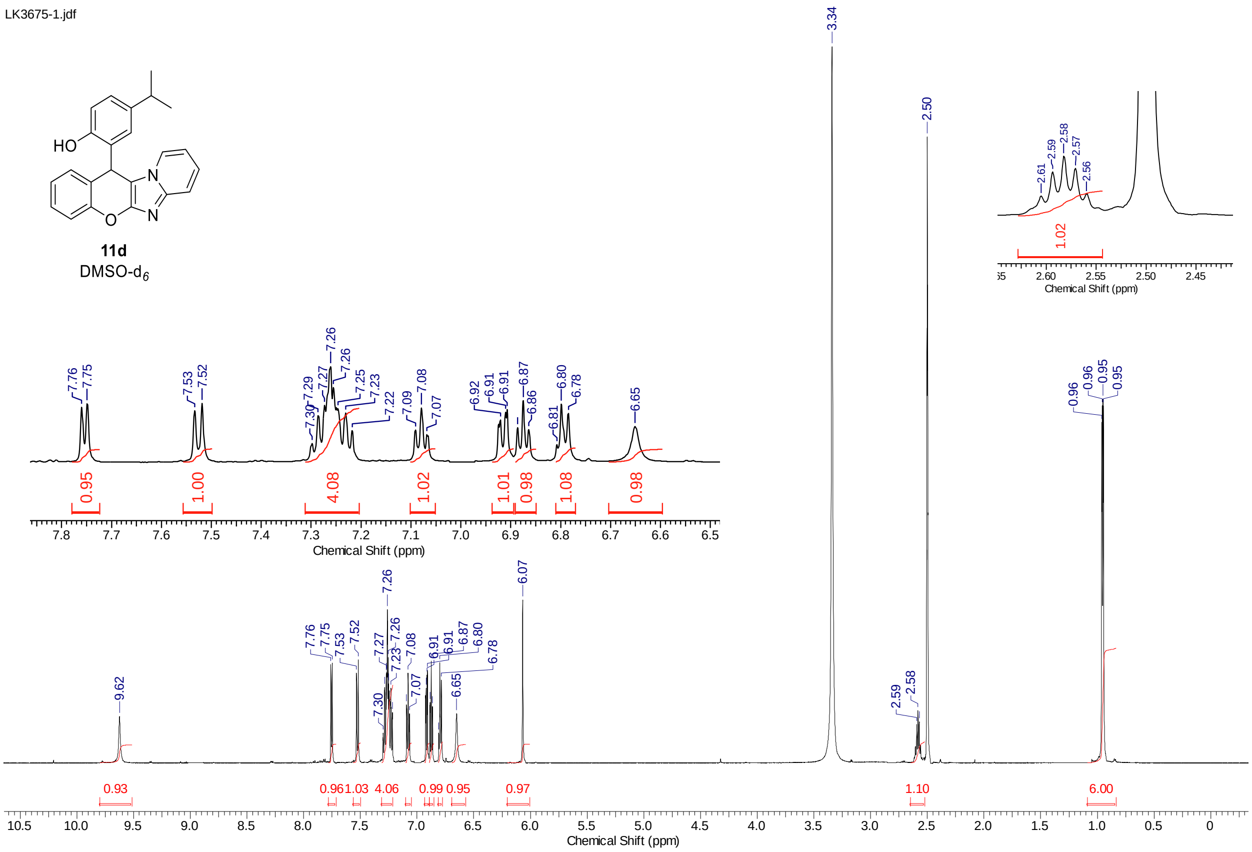

11d
DMSO-d$_6$

LK3680-1.jdf

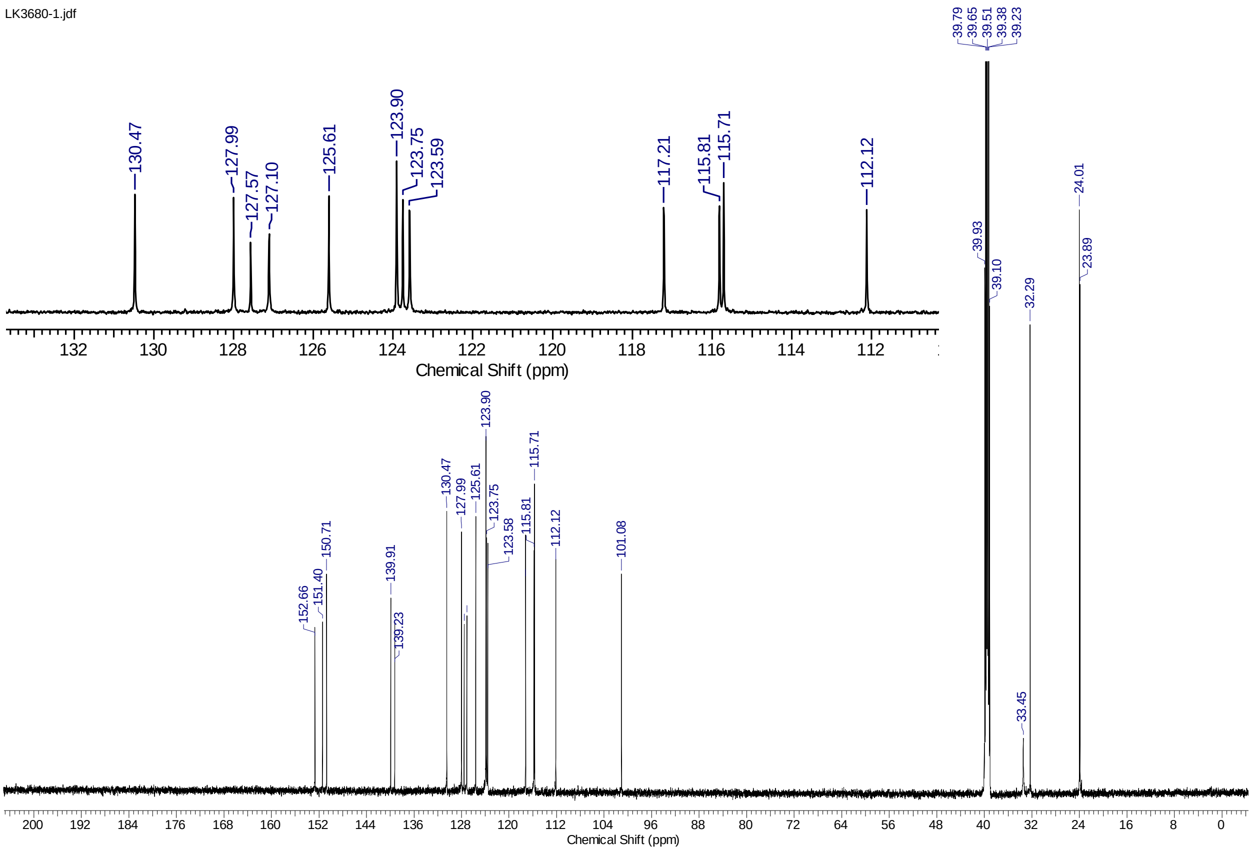

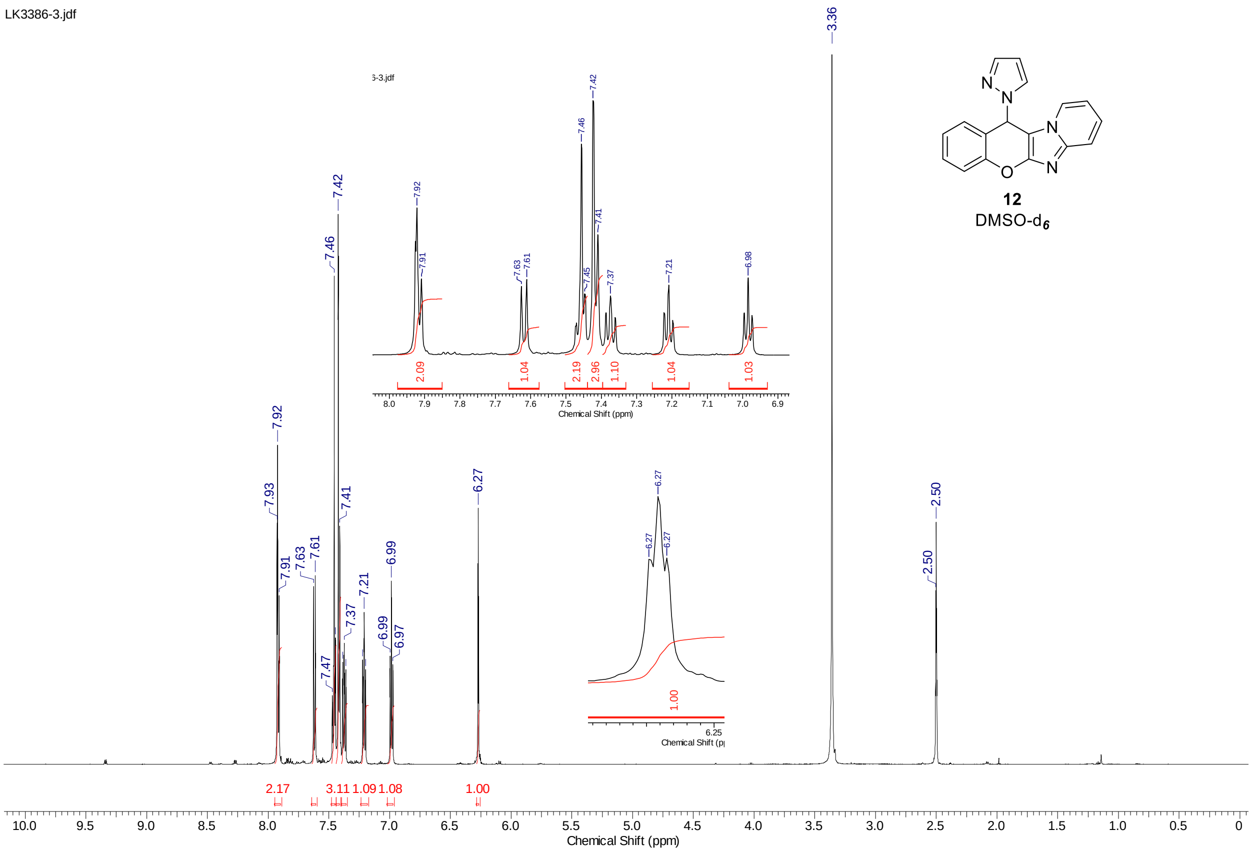
LK3386-3.jdf
12
DMSO-d6
Chemical Shift (ppm)

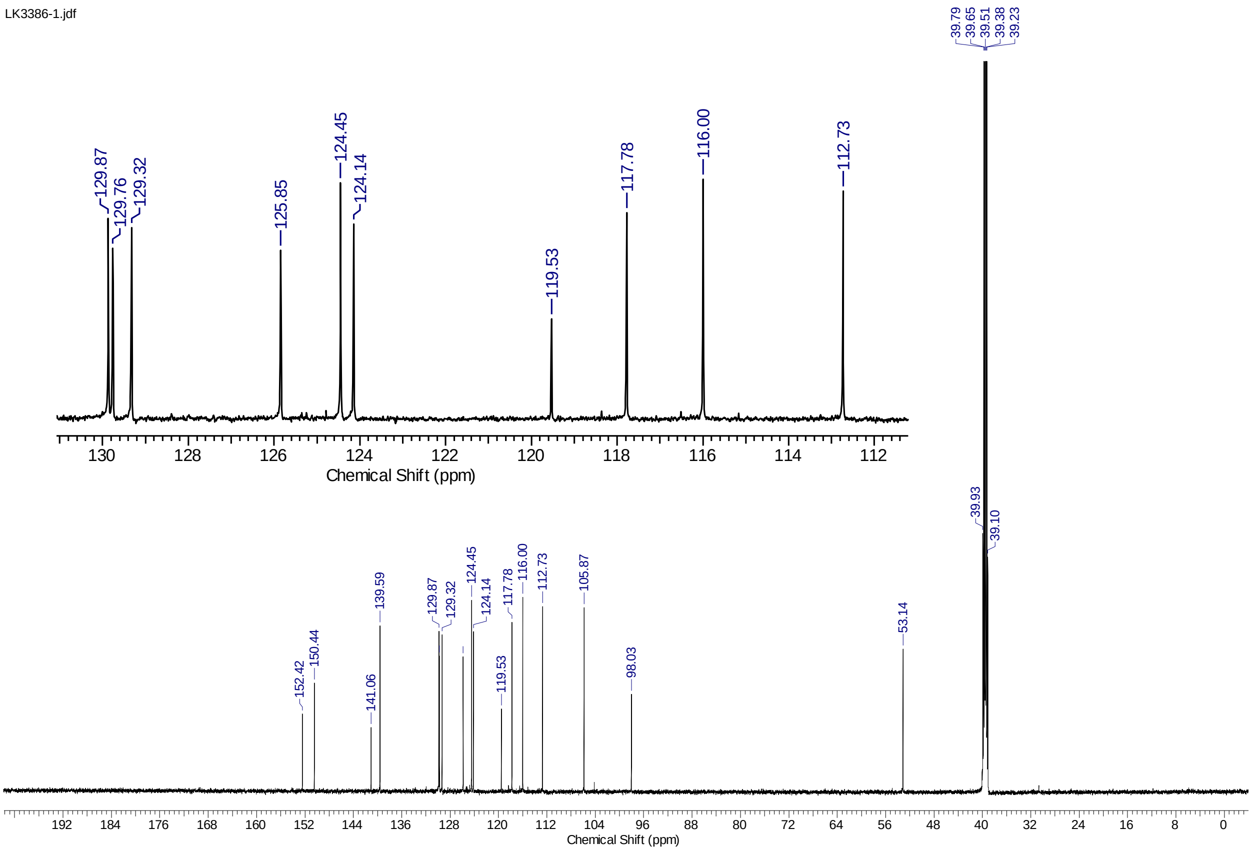
LK3386-1.jdf
Chemical Shift (ppm)

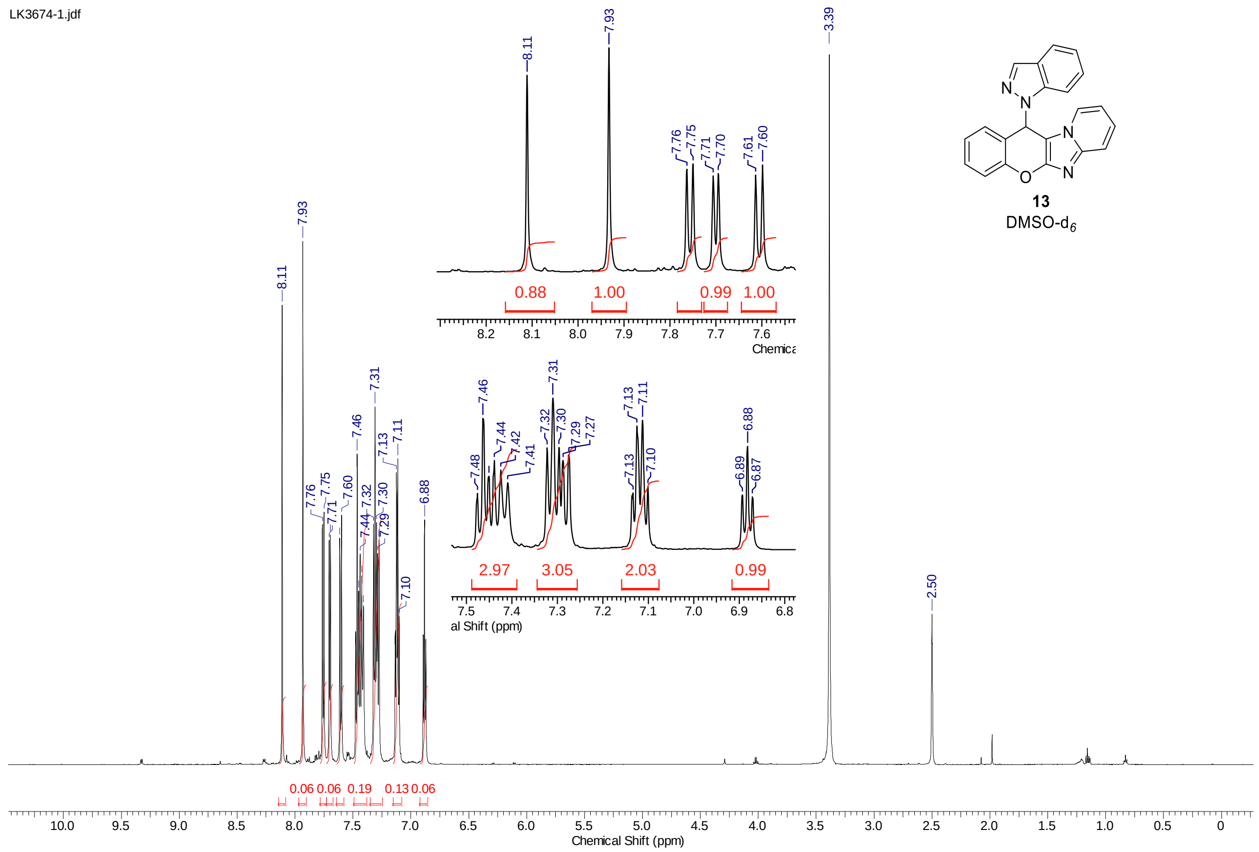
LK3674-1.jdf
13
DMSO-d6
8.11
7.93
7.76
7.75
7.71
7.70
7.61
7.60
7.48
7.46
7.44
7.42
7.41
7.32
7.31
7.30
7.29
7.27
7.13
7.11
7.10
6.89
6.88
6.87
3.39
2.50
0.88
1.00
0.99
1.00
2.97
3.05
2.03
0.99
0.06
0.06
0.19
0.13
0.06
Chemical Shift (ppm)

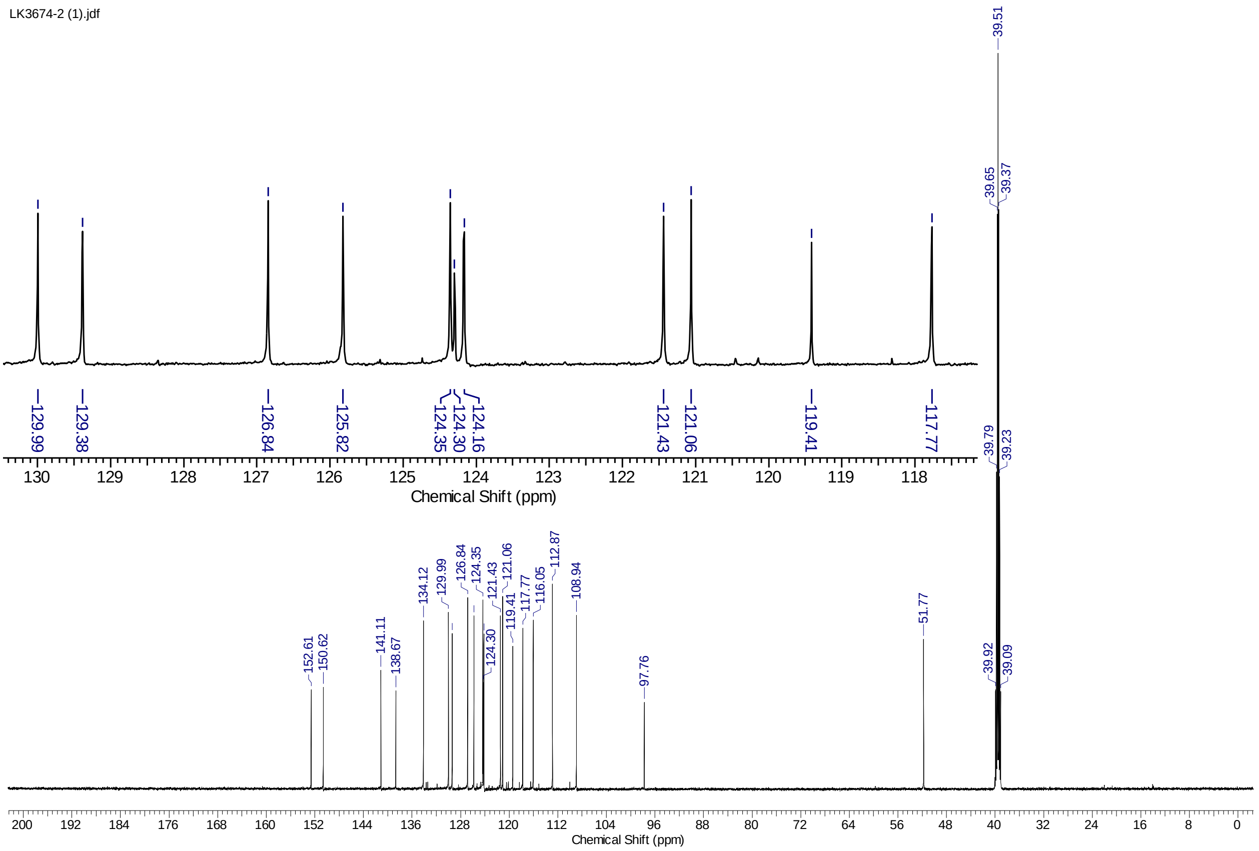
LK3674-2 (1).jdf
129.99
129.38
126.84
125.82
124.35
124.30
124.16
121.43
121.06
119.41
117.77
39.51
39.65
39.37
39.79
39.23
39.92
39.09
152.61
150.62
141.11
138.67
134.12
116.05
112.87
108.94
97.76
51.77
Chemical Shift (ppm)

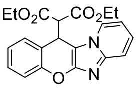

14a

$CDCl_3$

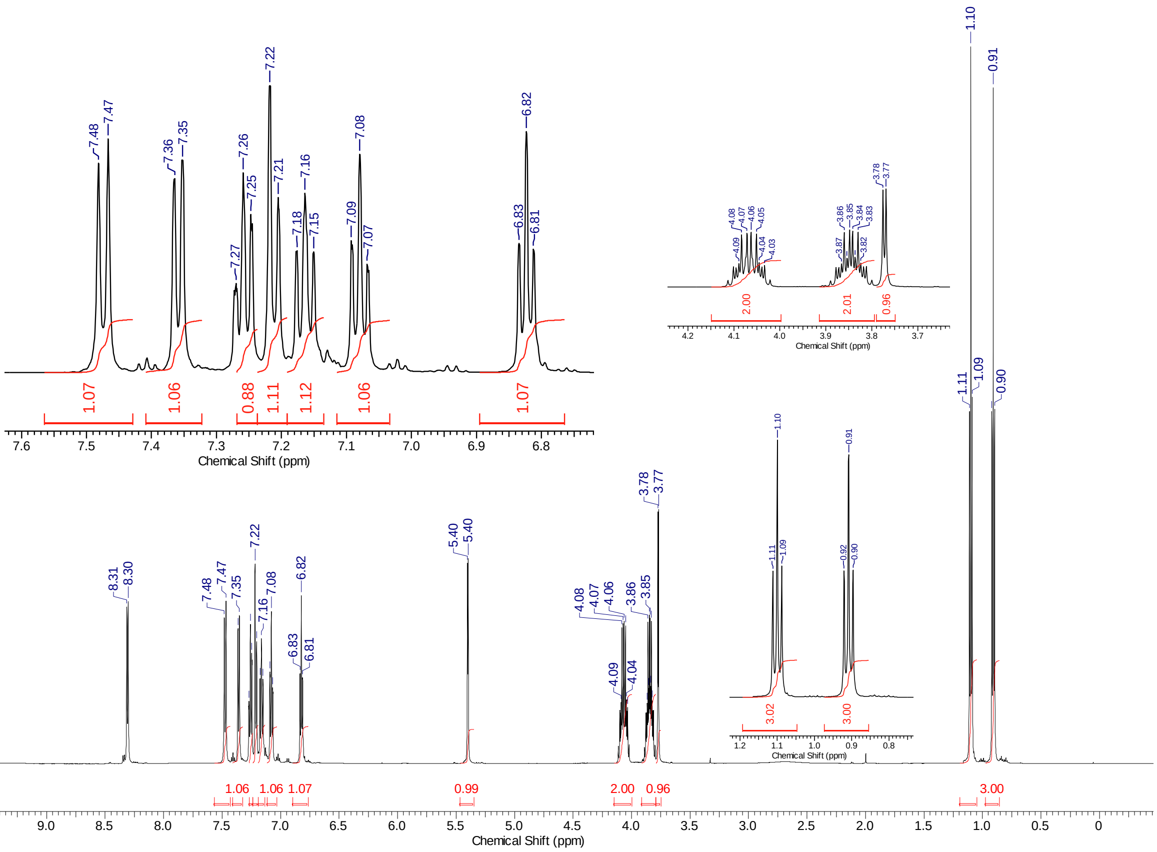
LK3512-1.esp
8.31 8.30 7.48 7.47 7.35 7.22 7.16 7.08 6.83 6.82 6.81 5.40 5.40 4.09 4.08 4.07 4.06 4.04 3.86 3.85 3.78 3.77 1.11 1.10 1.09 0.91 0.90
1.06 1.06 1.07 0.99 2.00 0.96 3.00
1.07 1.06 0.88 1.11 1.12 1.06 1.07
2.00 2.01 0.96
3.02 3.00
Chemical Shift (ppm)

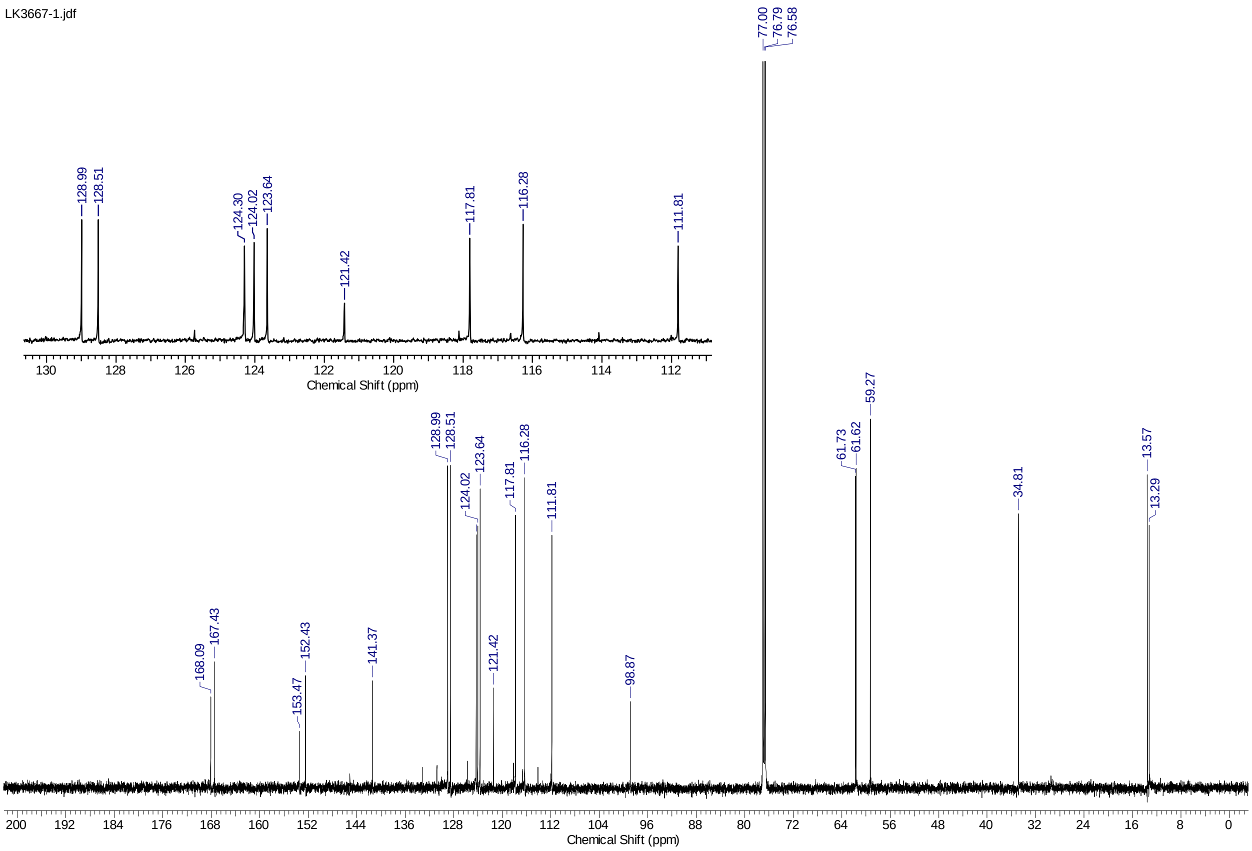
LK3667-1.jdf
128.99 128.51 124.30 124.02 123.64 121.42 117.81 116.28 111.81
168.09 167.43 153.47 152.43 141.37 98.87 77.00 76.79 76.58 61.73 61.62 59.27 34.81 13.57 13.29
Chemical Shift (ppm)

LK3560-1.jdf

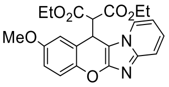

14b
DMSO-d_6

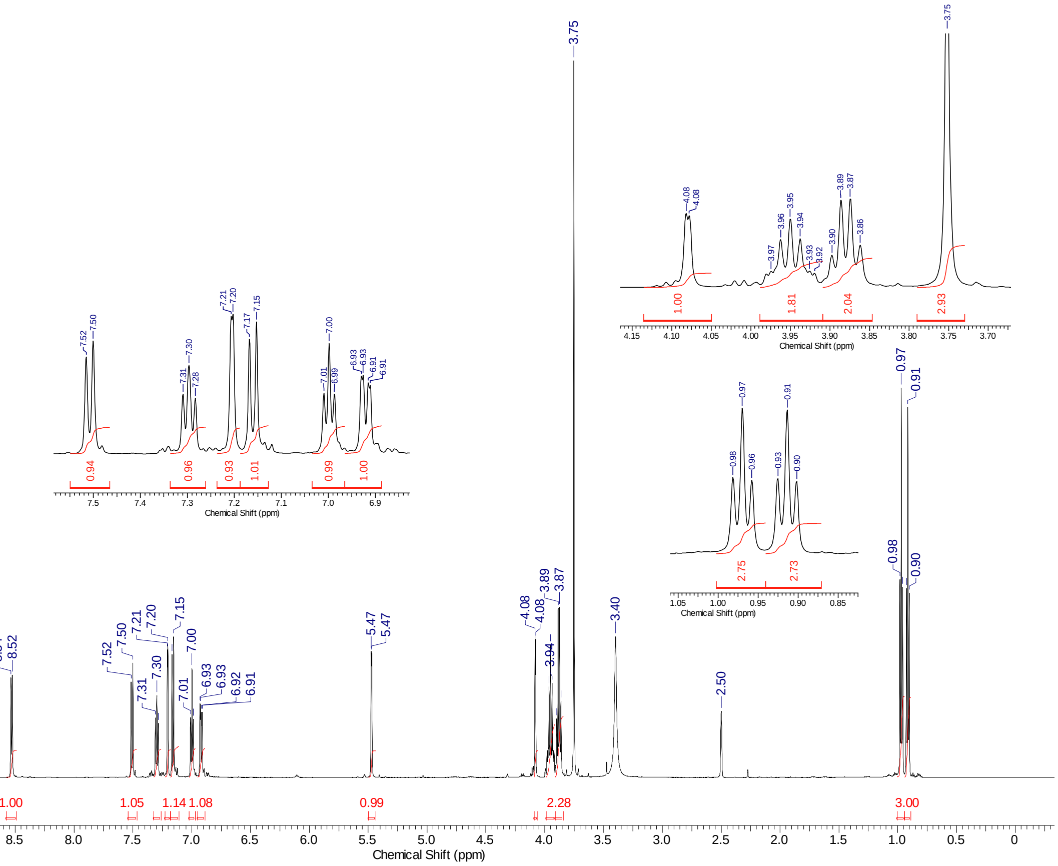

LK3560-4 (1).jdf

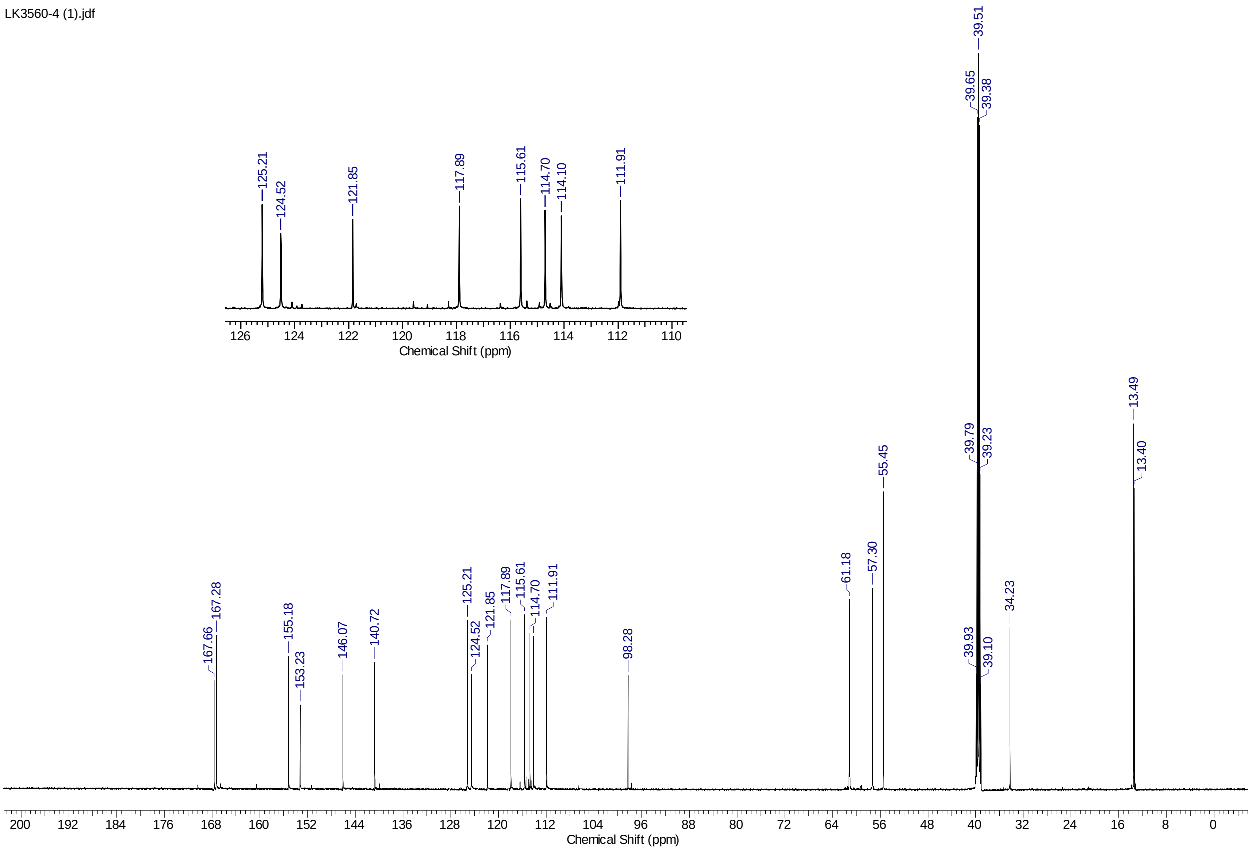

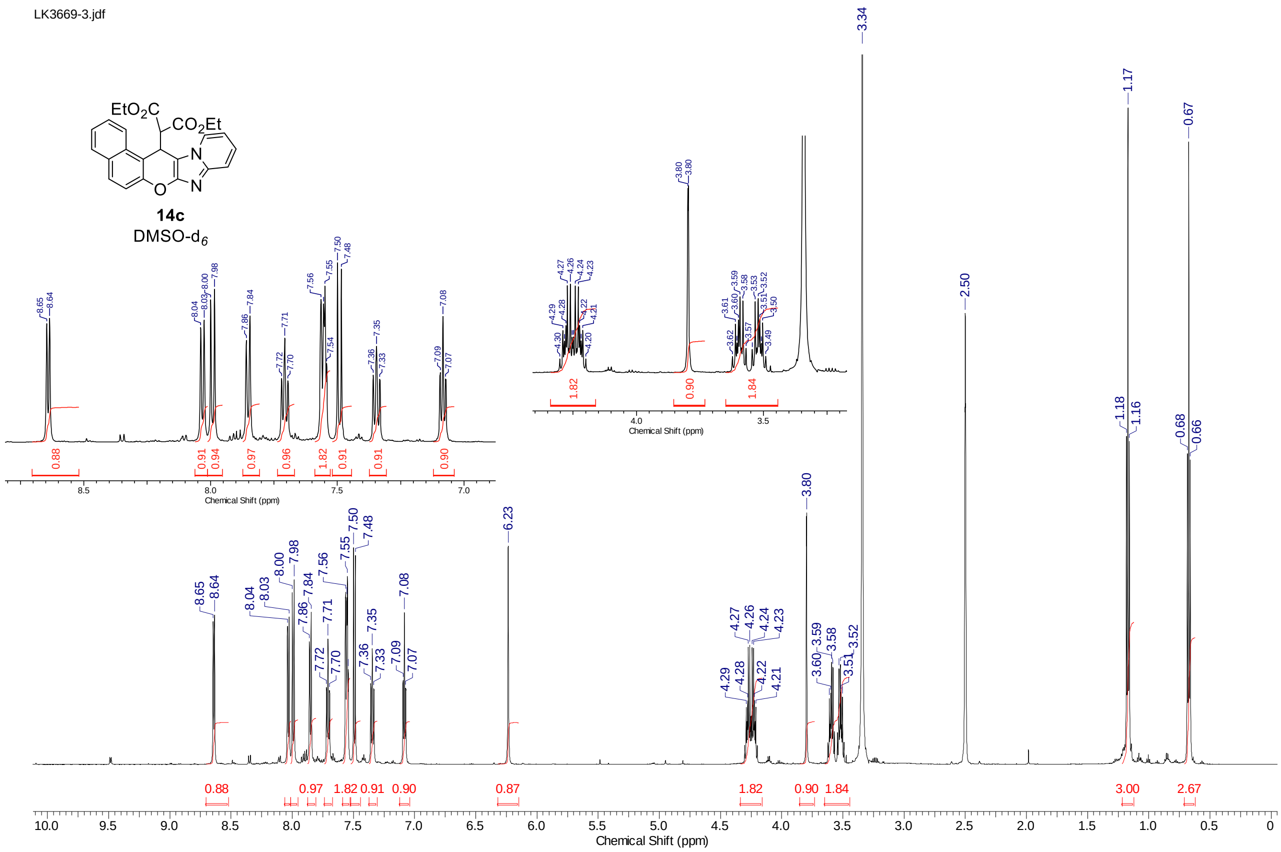
LK3669-3.jdf
14c
DMSO-d6
Chemical Shift (ppm)

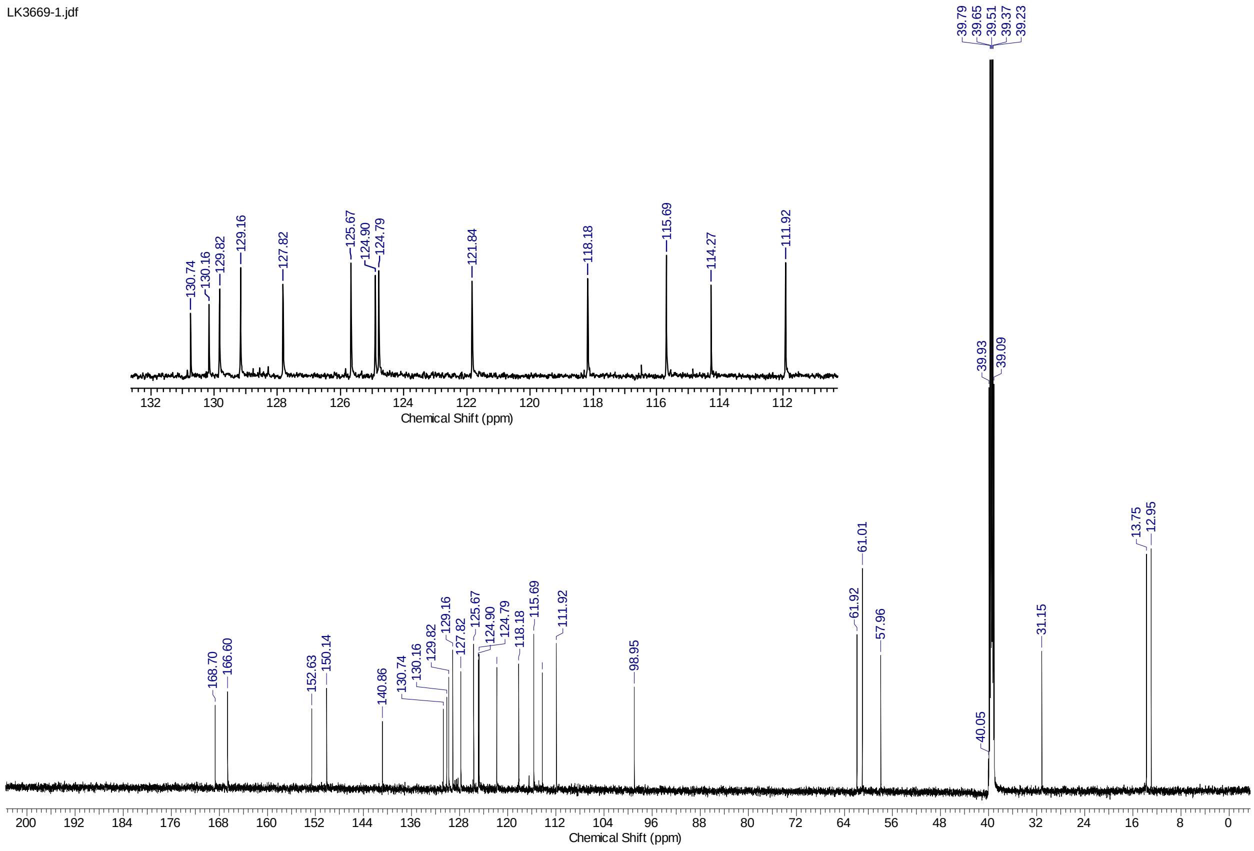
LK3669-1.jdf
Chemical Shift (ppm)

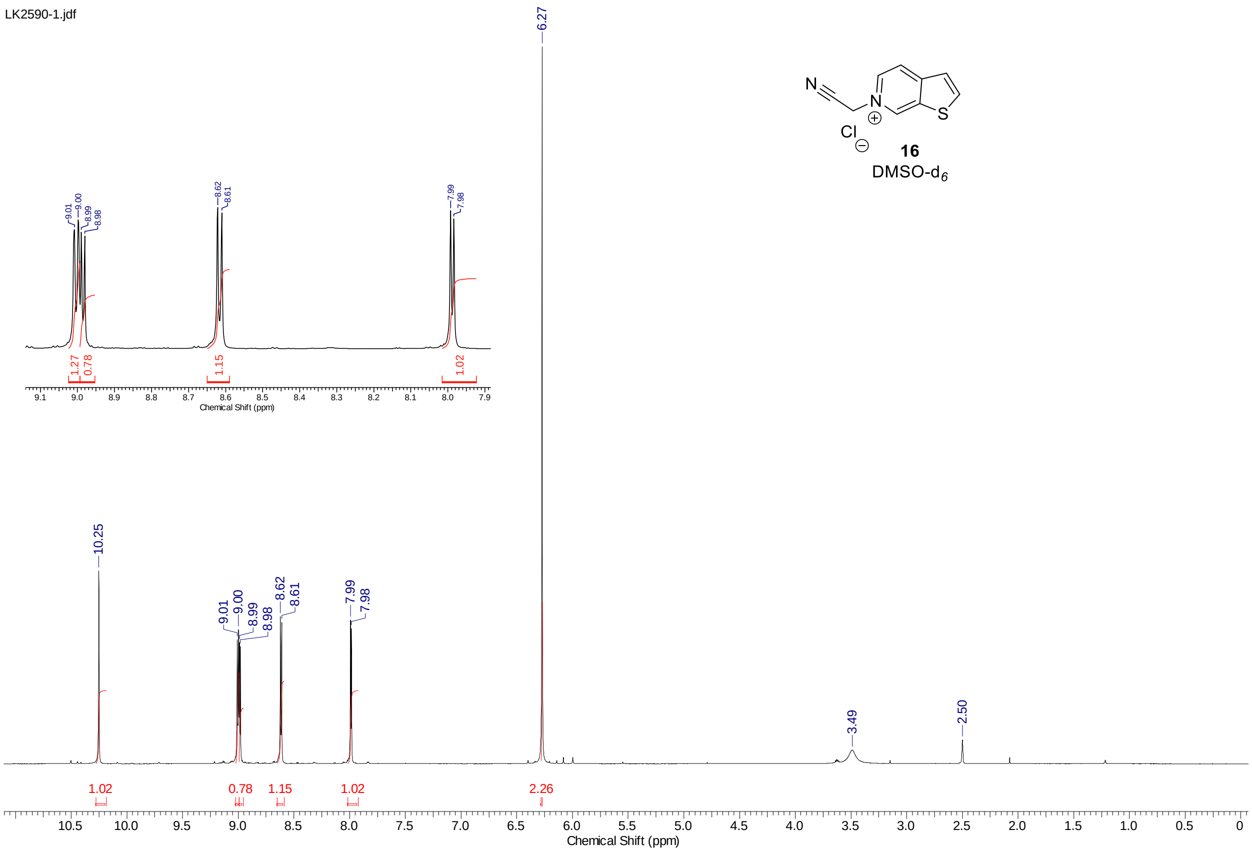
LK2590-1.jdf
16
DMSO-d6
N
N
Cl
S
10.25
9.01
9.00
8.99
8.98
8.62
8.61
7.99
7.98
6.27
3.49
2.50
1.02
0.78
1.15
1.02
2.26
1.27
0.78
1.15
1.02
Chemical Shift (ppm)

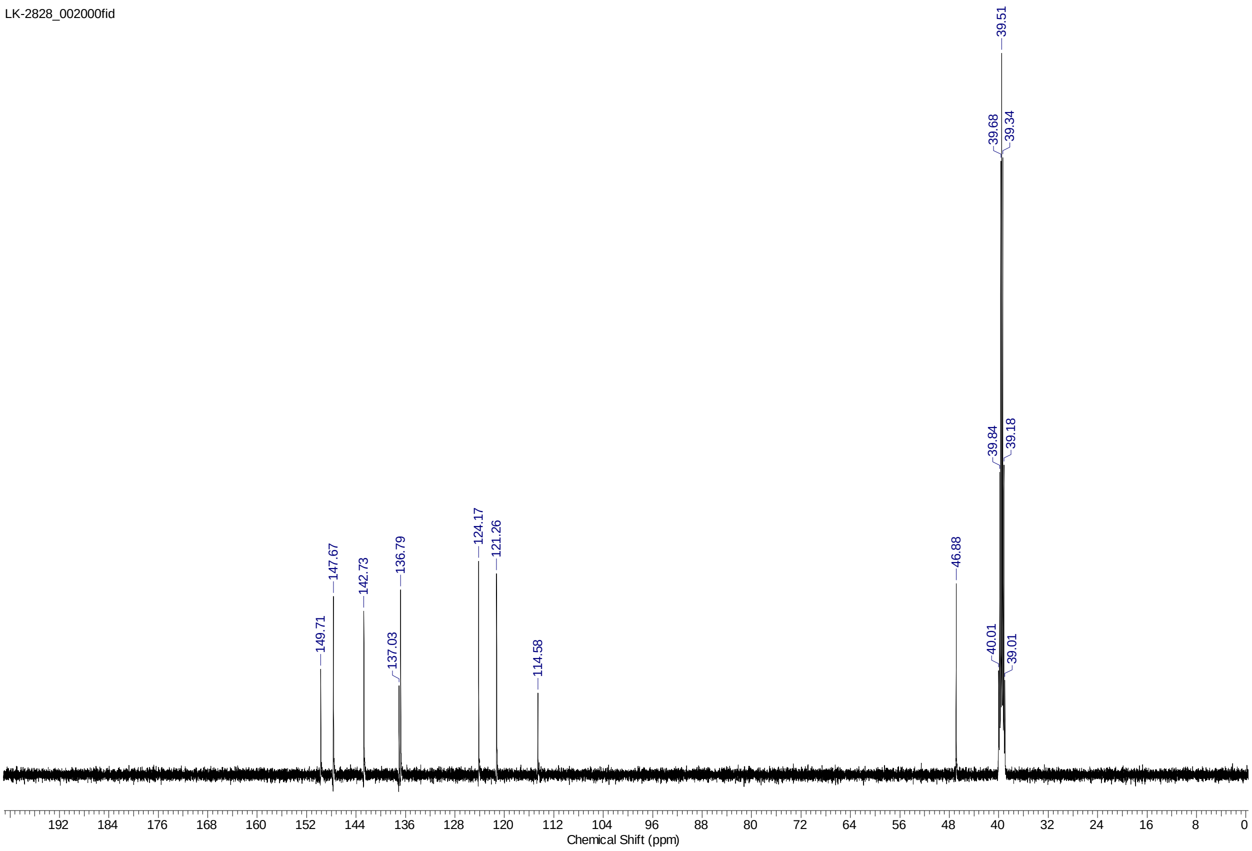
LK-2828_002000fid
149.71
147.67
142.73
137.03
136.79
124.17
121.26
114.58
46.88
40.01
39.84
39.68
39.51
39.34
39.18
39.01
Chemical Shift (ppm)

LK3608-1.jdf

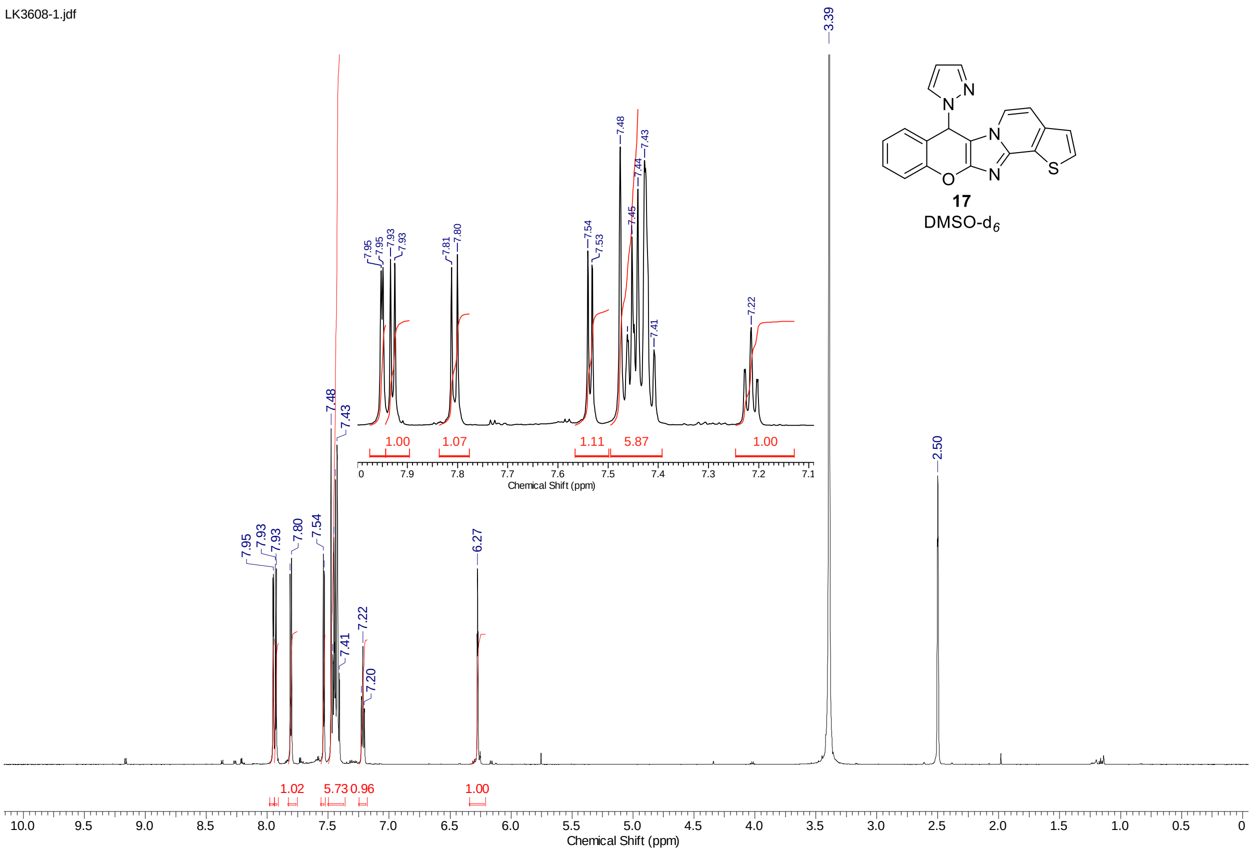

LK3628-1.jdf

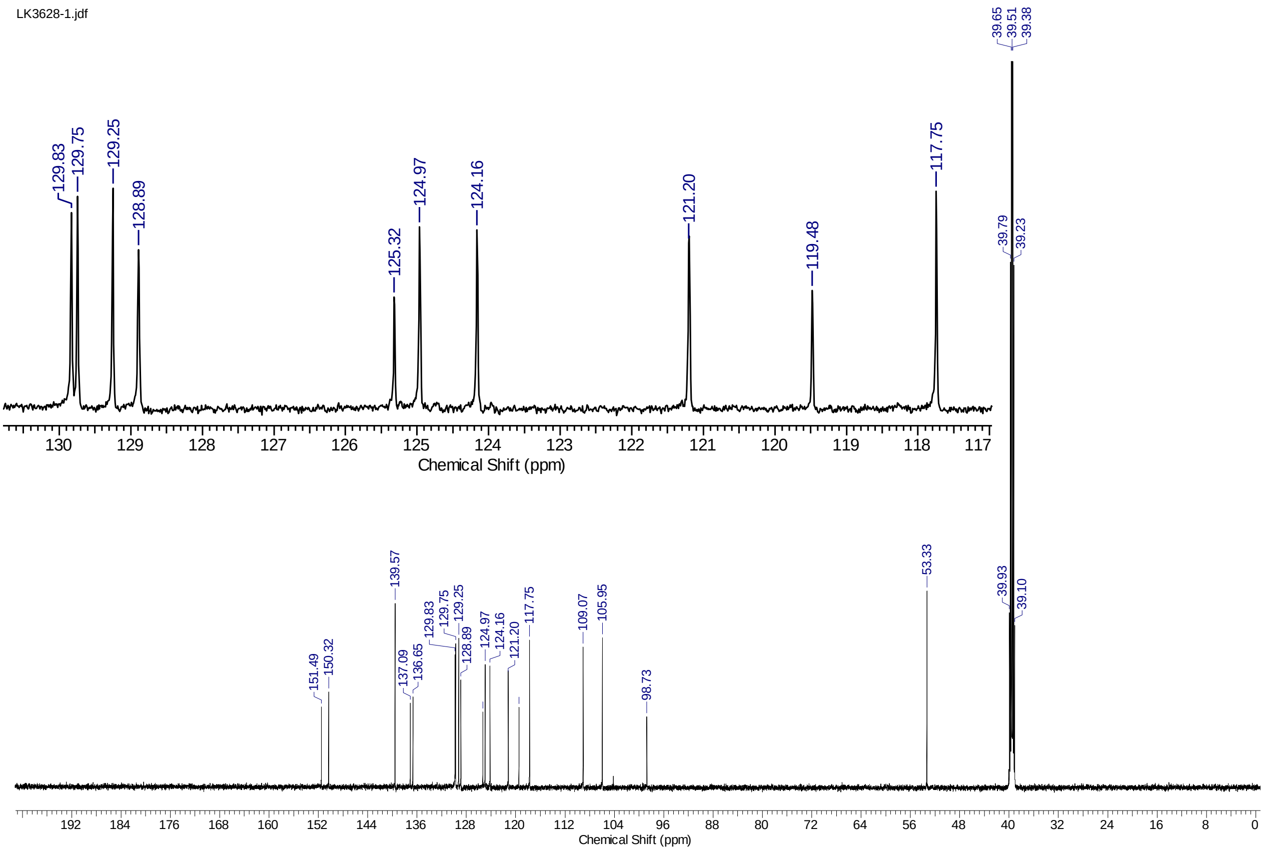

LK3574-1.jdf

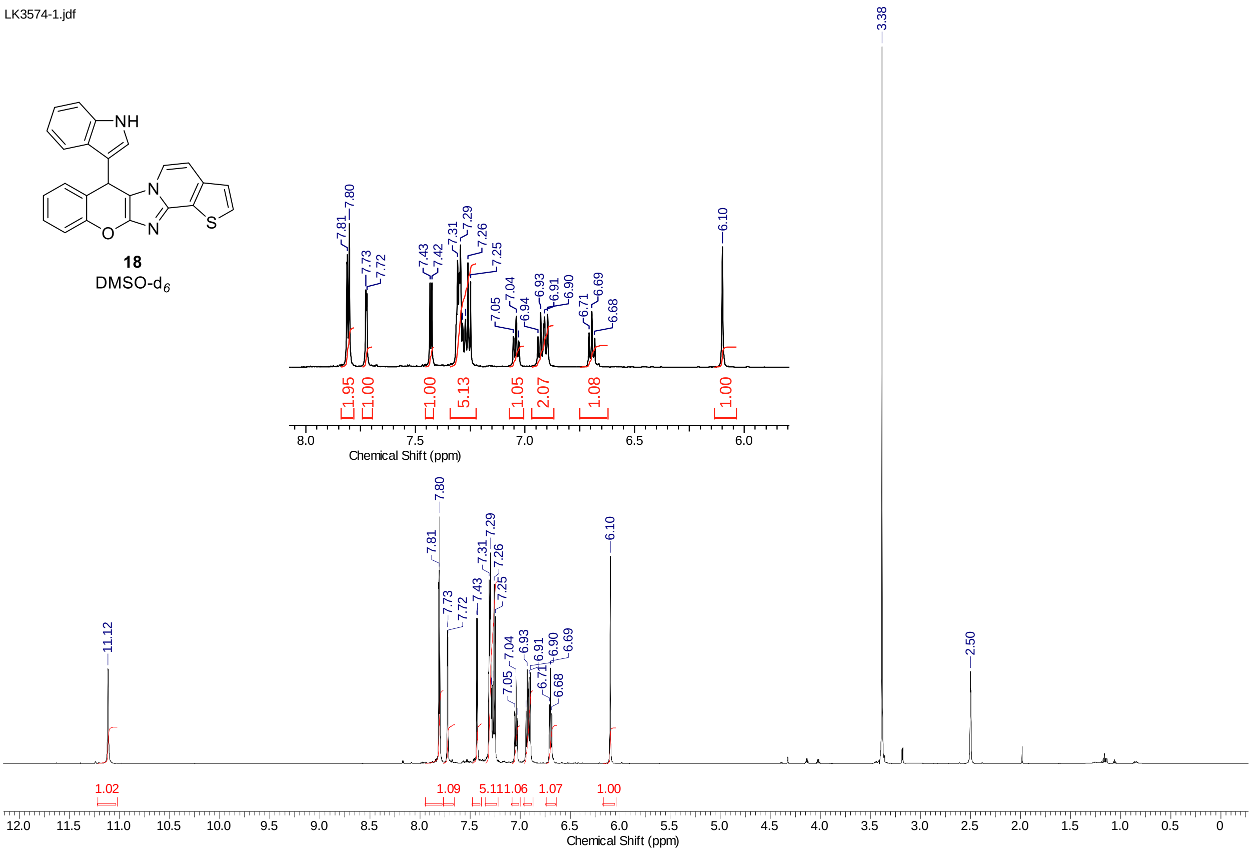

LK3629-1.jdf

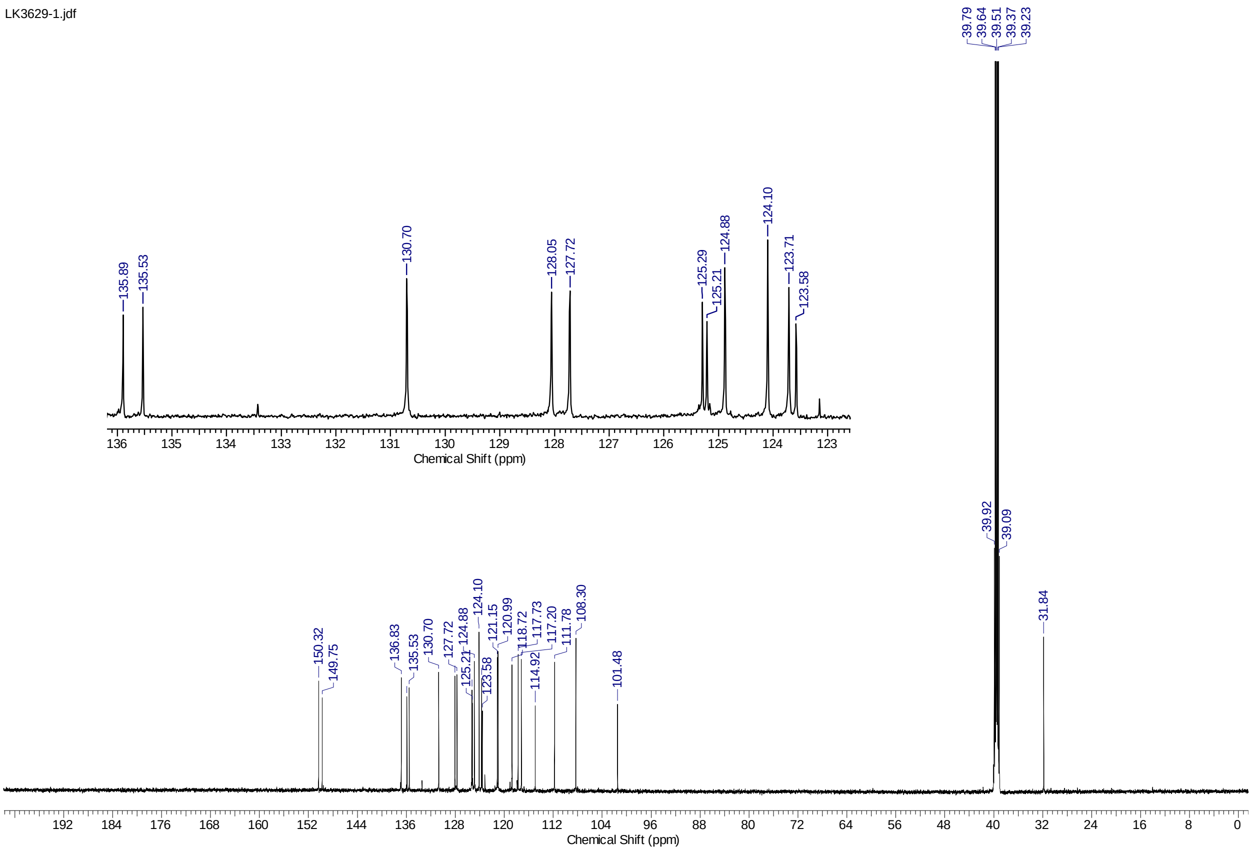

LK3591-1.jdf

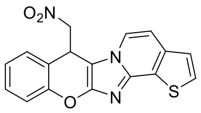

19
DMSO-d$_6$

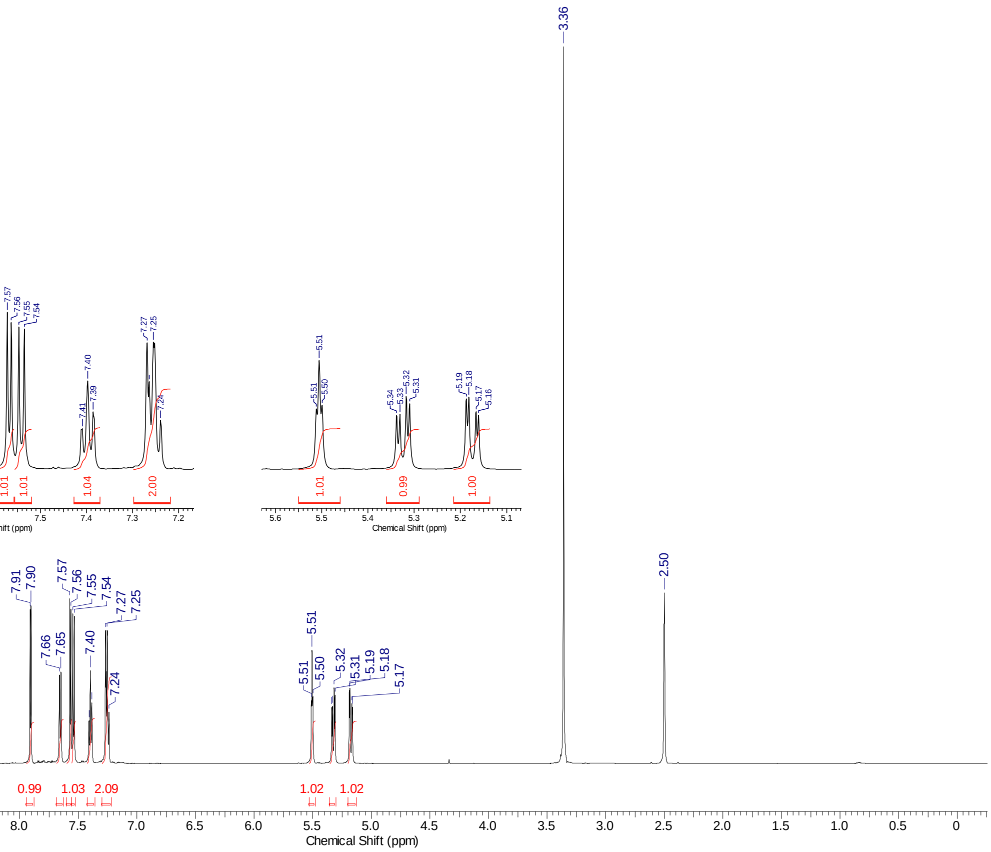

LK3627-1.jdf

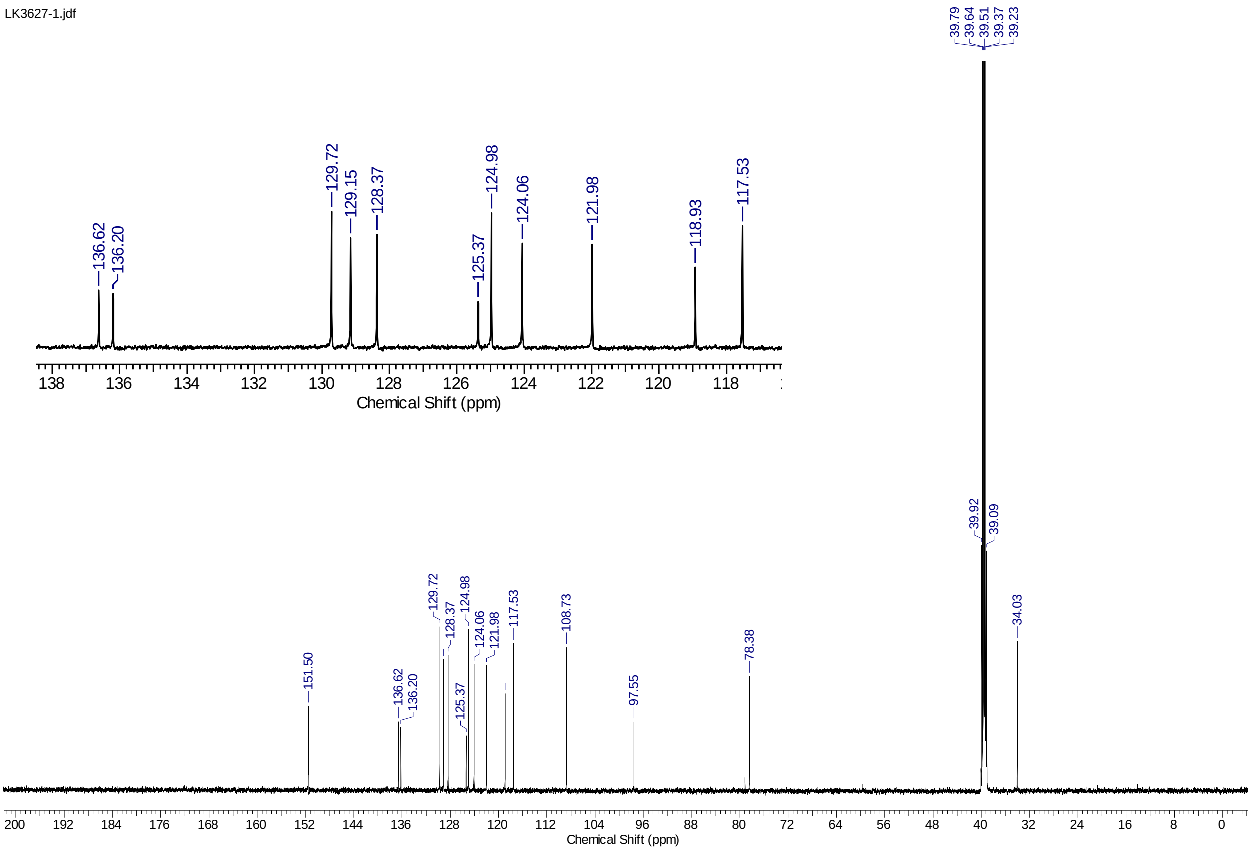

LK3691-1.jdf

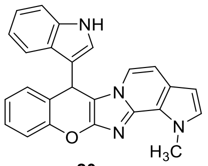

20

DMSO-d_6

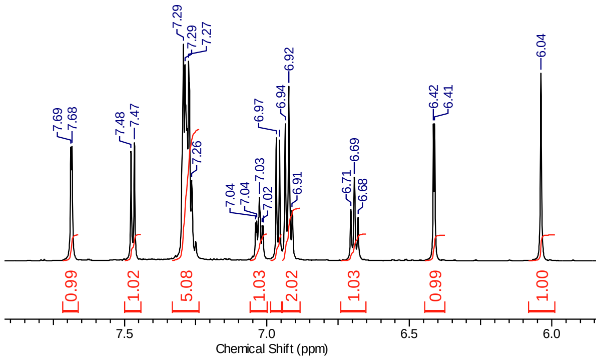

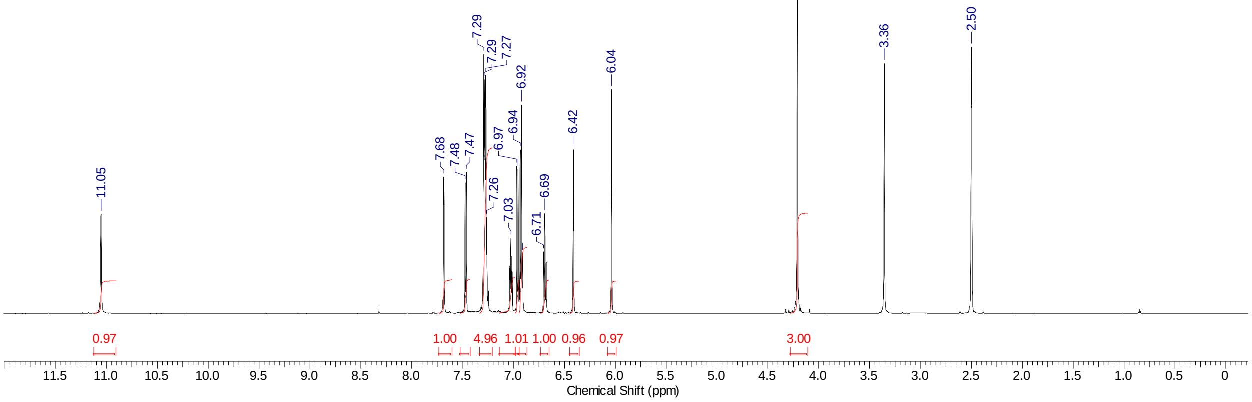

LK3726-2.esp

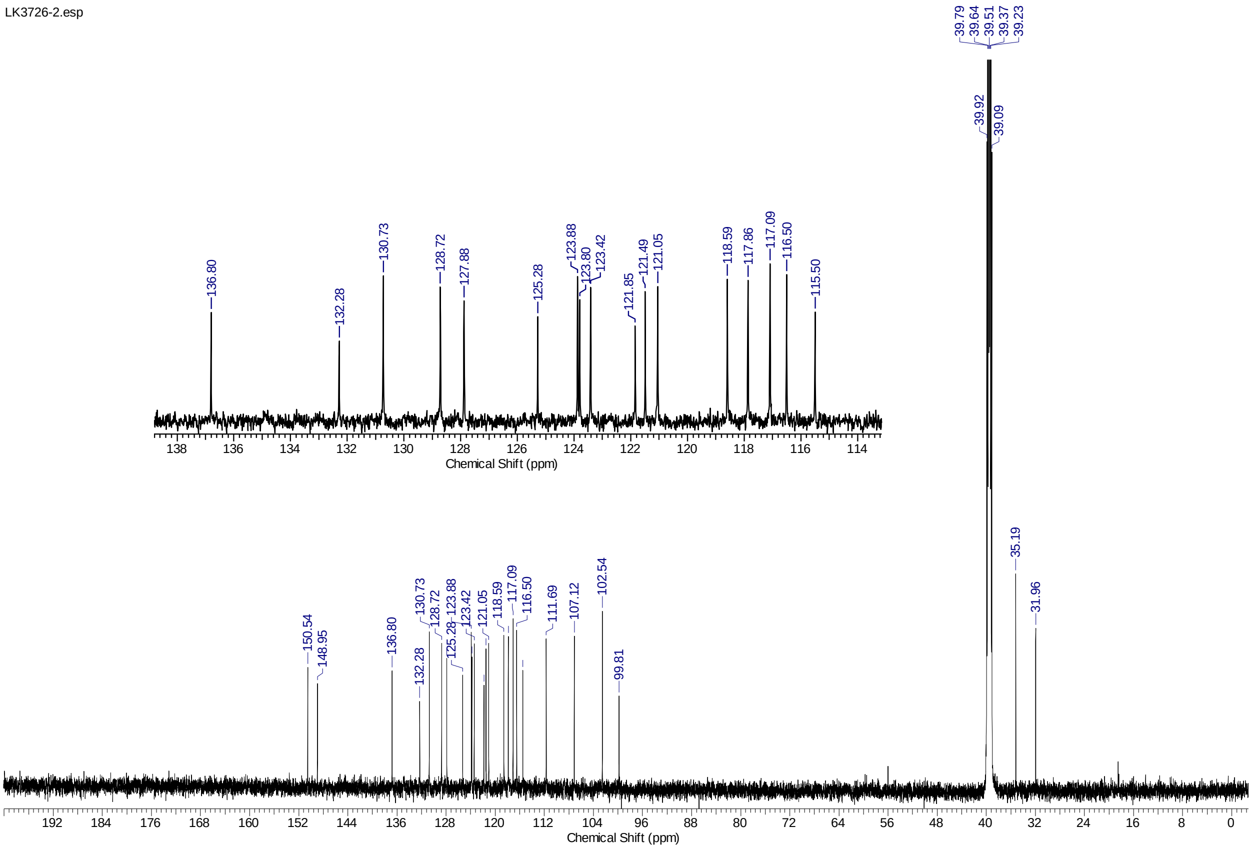

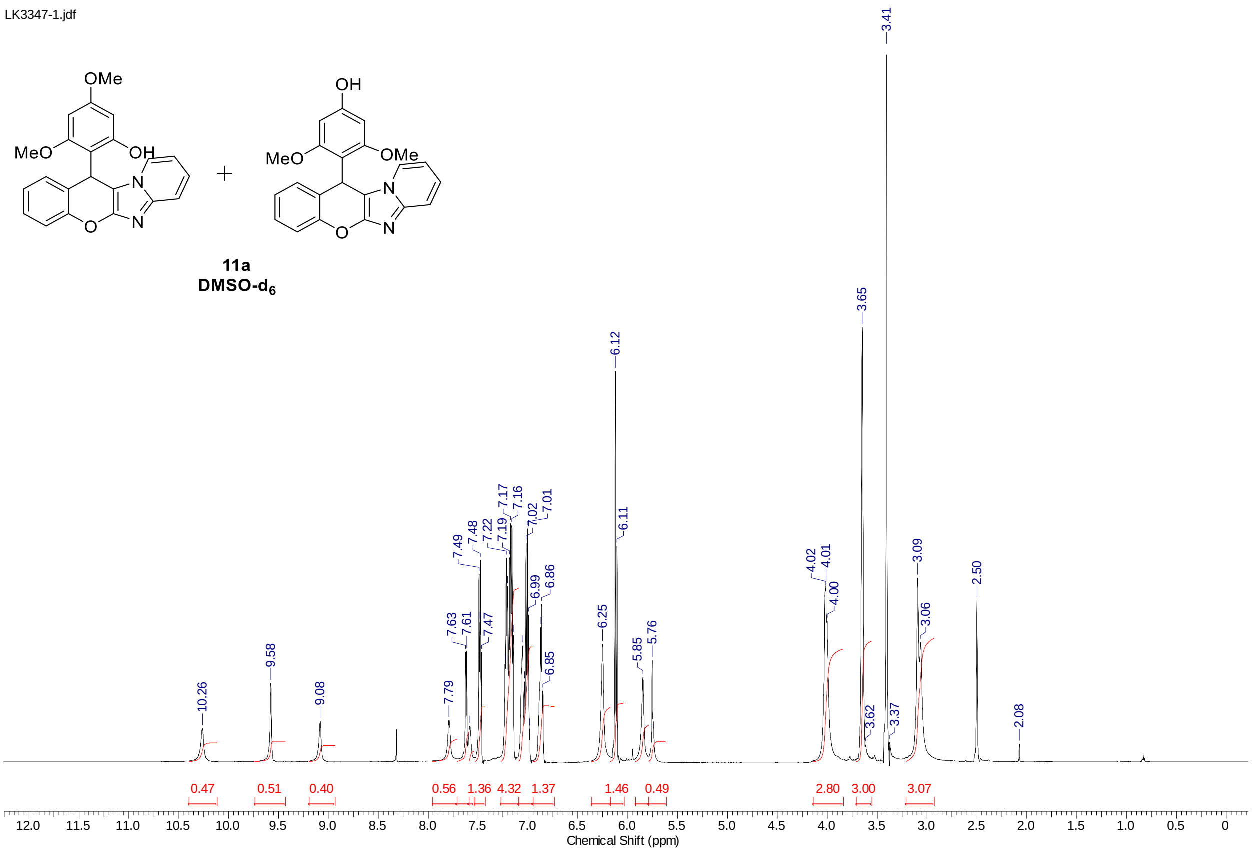

LK3347-1.jdf
OMe
MeO
OH
N
O
+
11a
DMSO-d6
10.26
9.58
9.08
7.79
7.63
7.61
7.49
7.48
7.47
7.22
7.19
7.17
7.16
7.02
7.01
6.99
6.86
6.85
6.25
6.12
6.11
5.85
5.76
4.02
4.01
4.00
3.65
3.62
3.41
3.37
3.09
3.06
2.50
2.08
0.47
0.51
0.40
0.56
1.36
4.32
1.37
1.46
0.49
2.80
3.00
3.07
Chemical Shift (ppm)

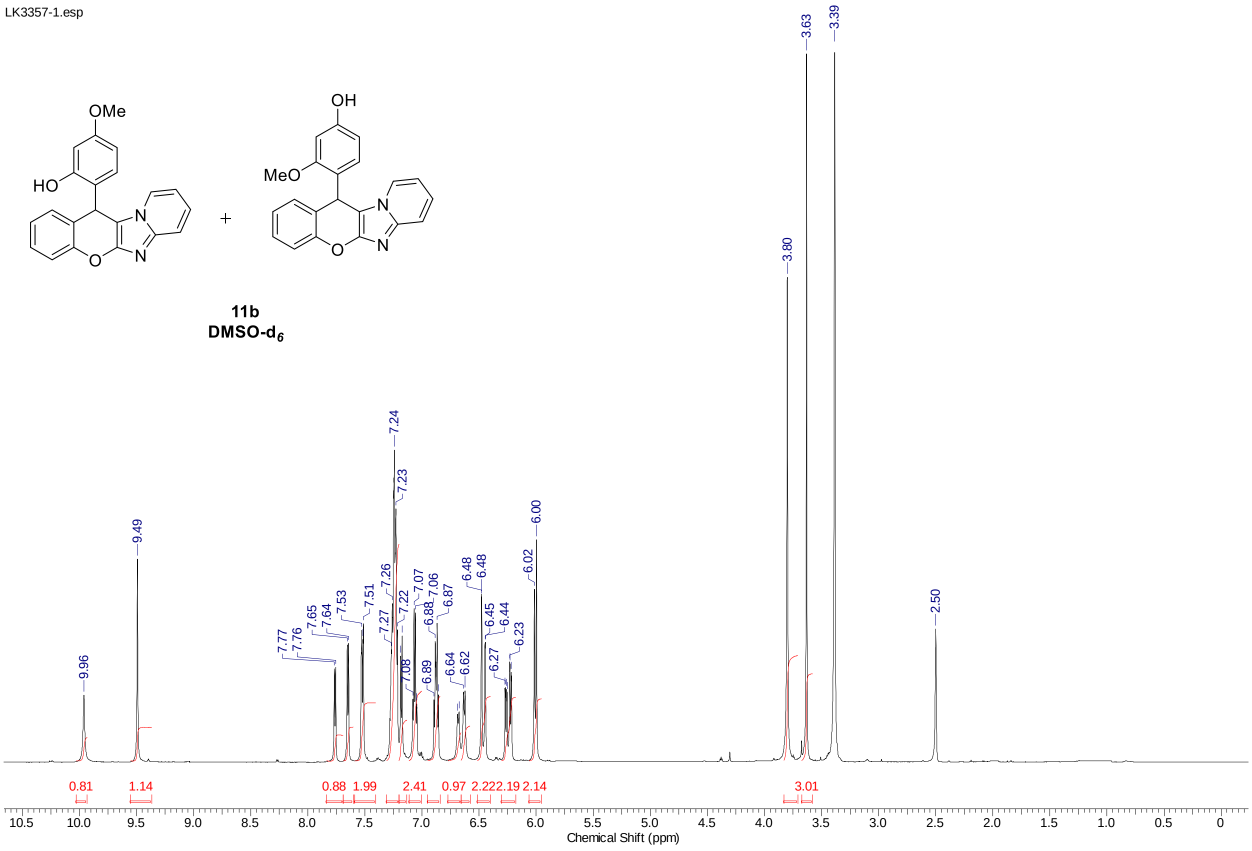

LK3357-1.esp
OMe
HO
OH
MeO
N
O
+
11b
DMSO-d6
9.96
9.49
7.77
7.76
7.65
7.64
7.53
7.51
7.27
7.26
7.24
7.23
7.22
7.08
7.07
7.06
6.89
6.88
6.87
6.64
6.62
6.48
6.48
6.45
6.44
6.27
6.23
6.02
6.00
3.80
3.63
3.39
2.50
0.81
1.14
0.88
1.99
2.41
0.97
2.22
2.19
2.14
3.01
Chemical Shift (ppm)

==== Shimadzu LabSolutions Data Report ====

Sample ID : Acquired 23.05.2017 18:12:30
Data Filename : lk-395 08.lcd

<Chromatogram>

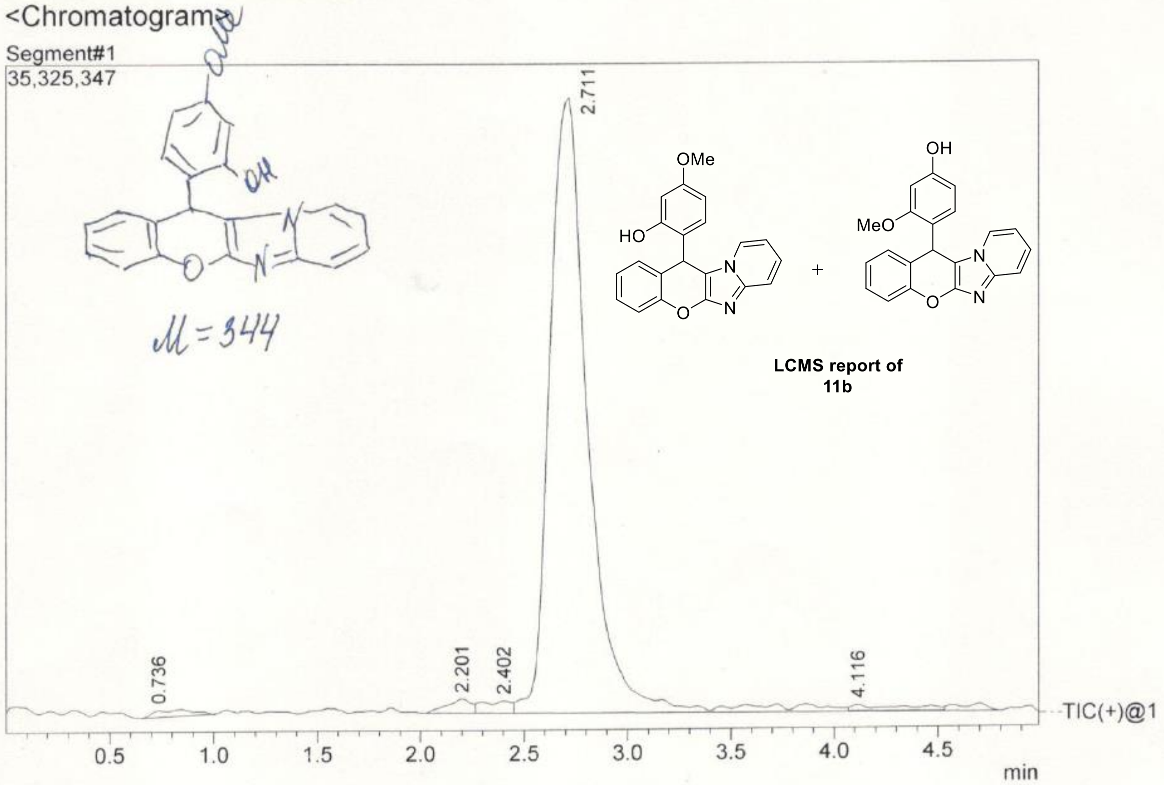

LCMS report of 11b

<Spectrum>

R.Time:----(Scan#:----)
MassPeaks:2 BasePeak:345(18747413)
Spectrum Mode:Averaged 2.683-2.717(162-164)
BG Mode:Calc Polarity:Positive Segment 1 - Event 1

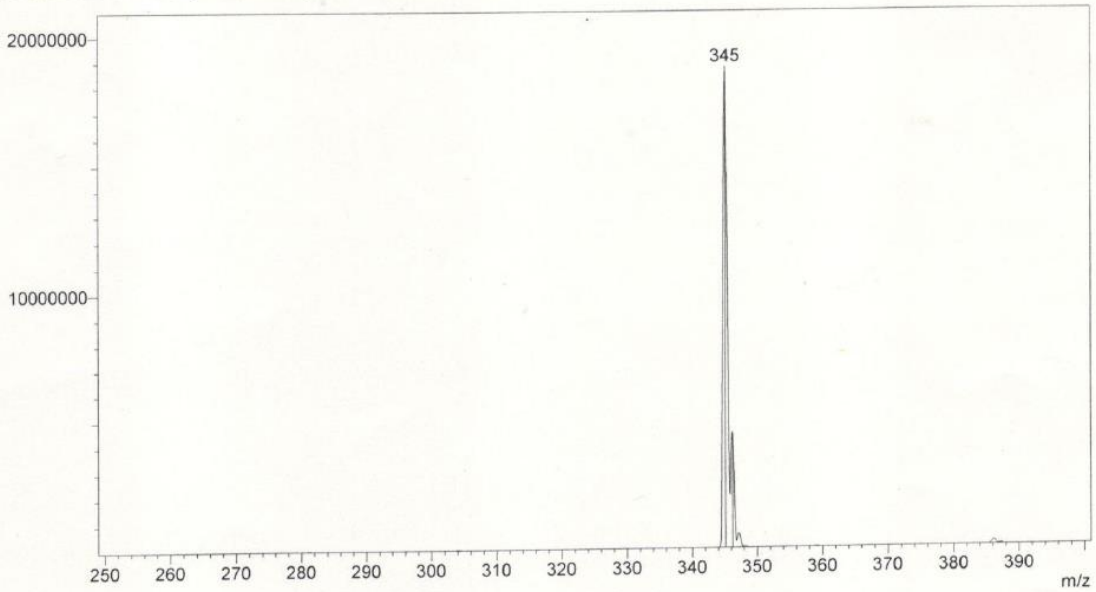

C:¥LabSolutions¥Sample¥new ms¥081216¥lk-395_08.lcd

==== Shimadzu LabSolutions Data Report ====

Sample ID :
Data Filename : lk-377 20.lcd
Acquired 27.04.2017 21:05:17

<Chromatogram>

Segment#1

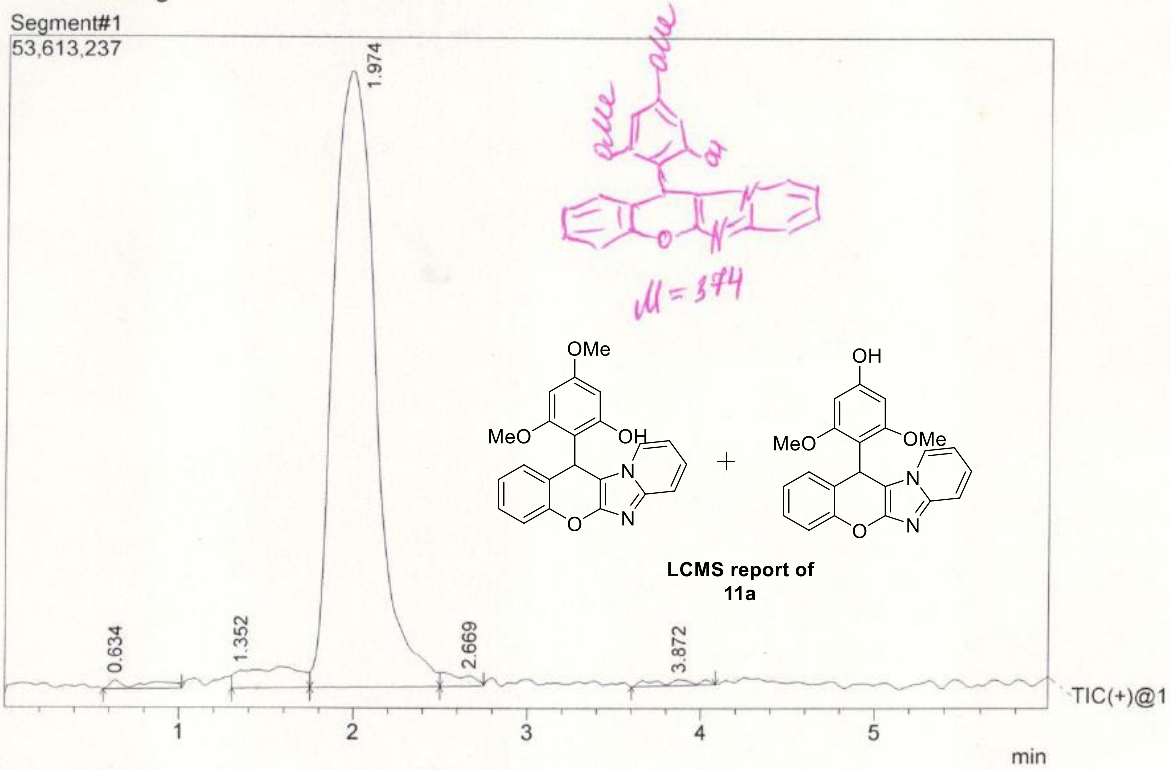

LCMS report of 11a

<Spectrum>

R.Time:1.917(Scan#:116)
MassPeaks:3 BasePeak:375(21153438)
Spectrum Mode:Single 1.917(116)
BG Mode:None Polarity:Positive Segment 1 - Event 1

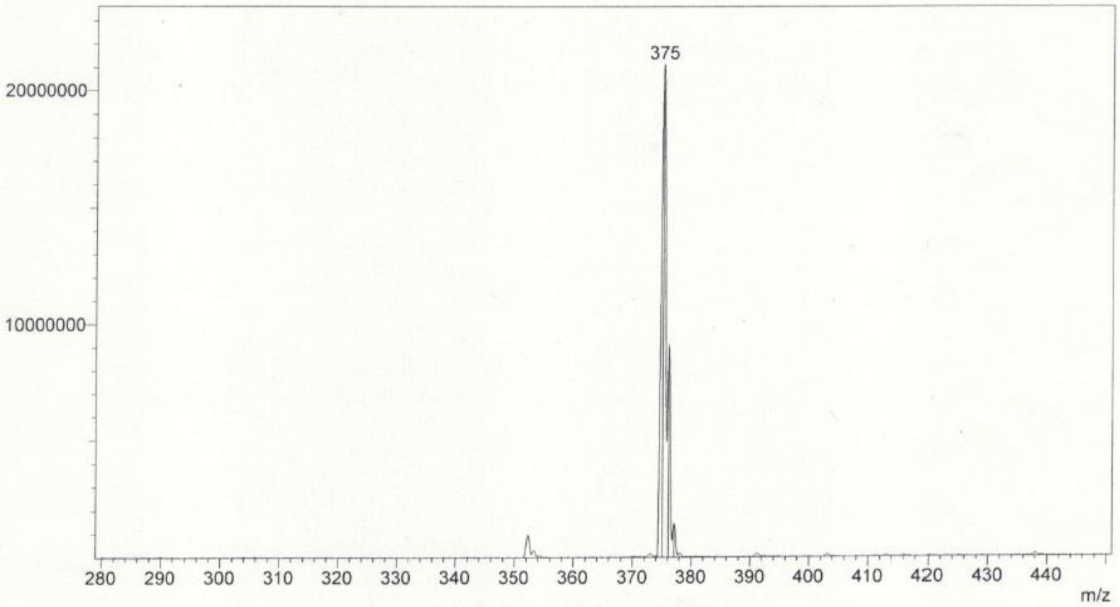

C:¥LabSolutions¥Sample¥new ms¥081216¥lk-377_20.lcd

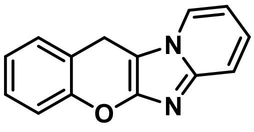

4

DMSO-d_6

.

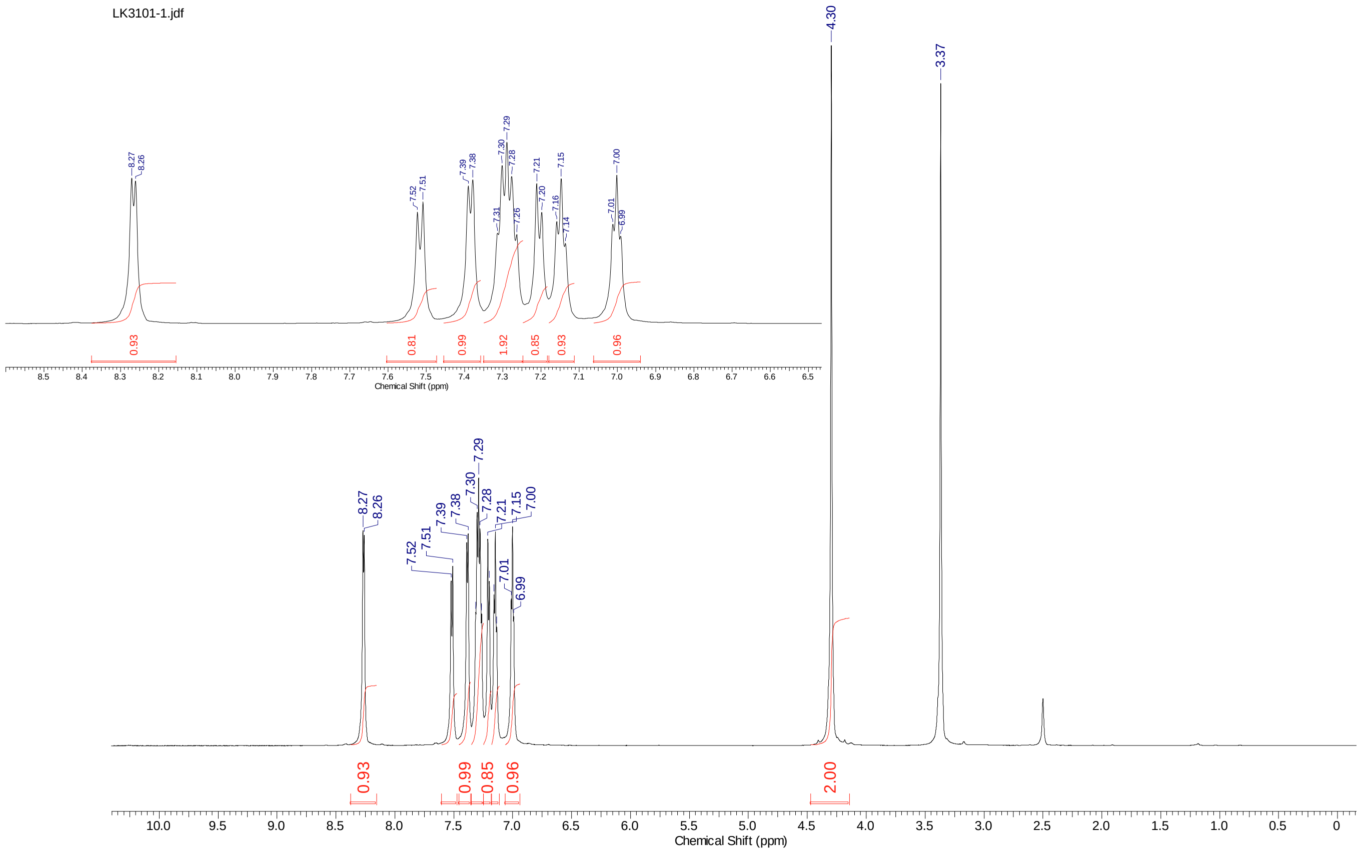
LK3101-1.jdf
4.30
3.37
8.27
8.26
7.52
7.51
7.39
7.38
7.31
7.30
7.29
7.28
7.26
7.21
7.20
7.16
7.15
7.14
7.01
7.00
6.99
0.93
0.81
0.99
1.92
0.85
0.93
0.96
2.00
Chemical Shift (ppm)

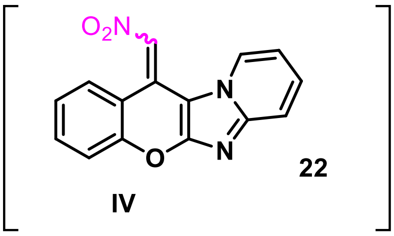

1 H NMR, DMSO-d_6
ESI MS: m/z 280 $[M+H]^{+\cdot}$

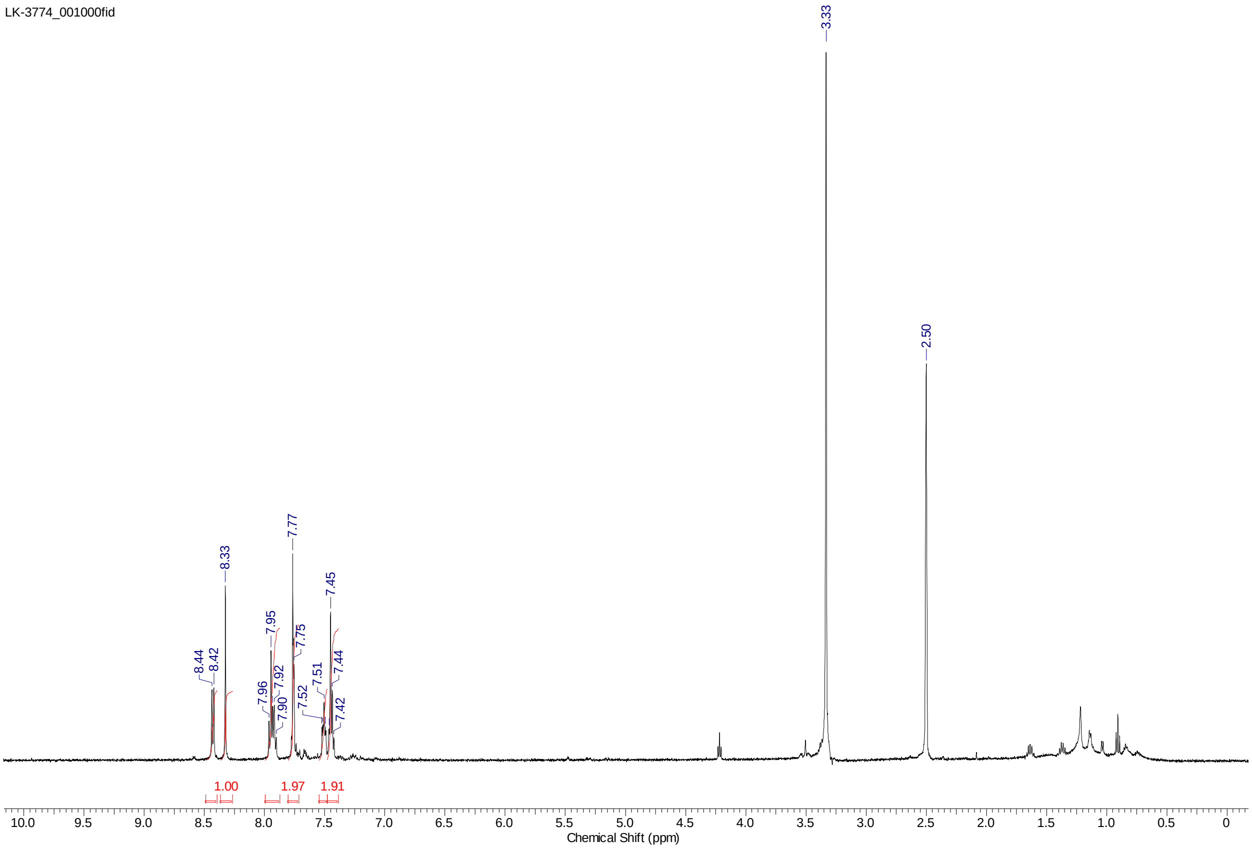

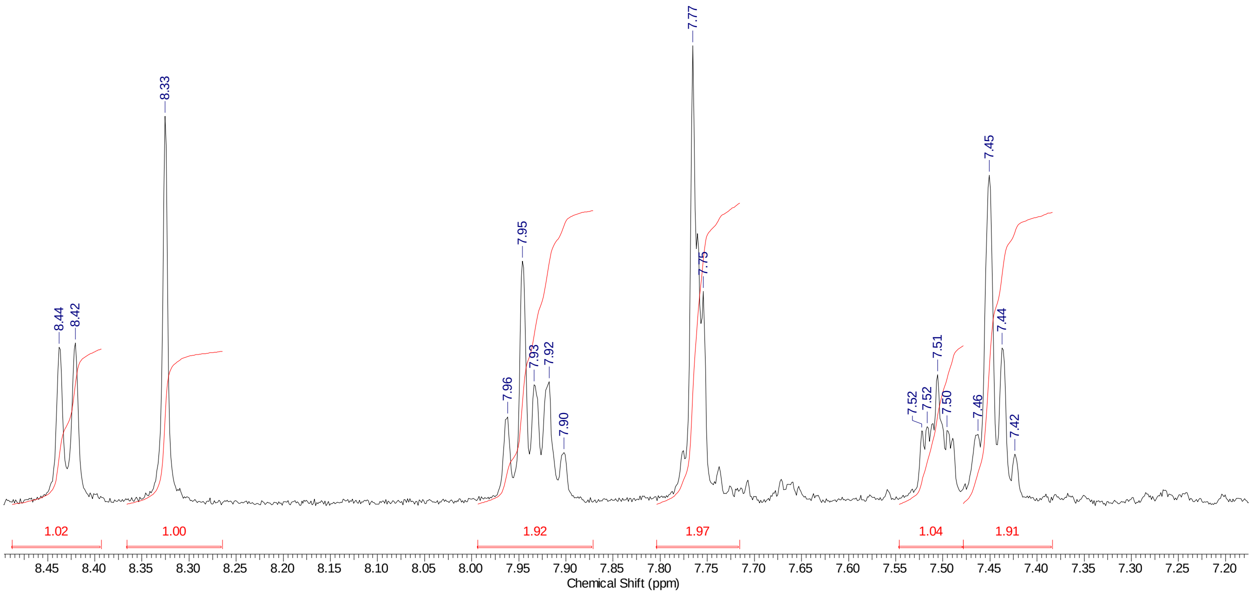

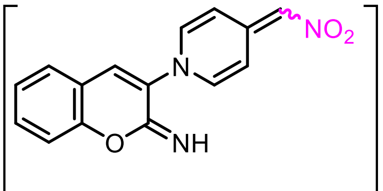

II 21

^{1}H NMR, DMSO-d_6

ESI MS: m/z 282 $[M+H]^+$.

IR: 3281cm^{-1} (NH); 1653 cm^{-1} (C=NH)

LK3748-1.jdf

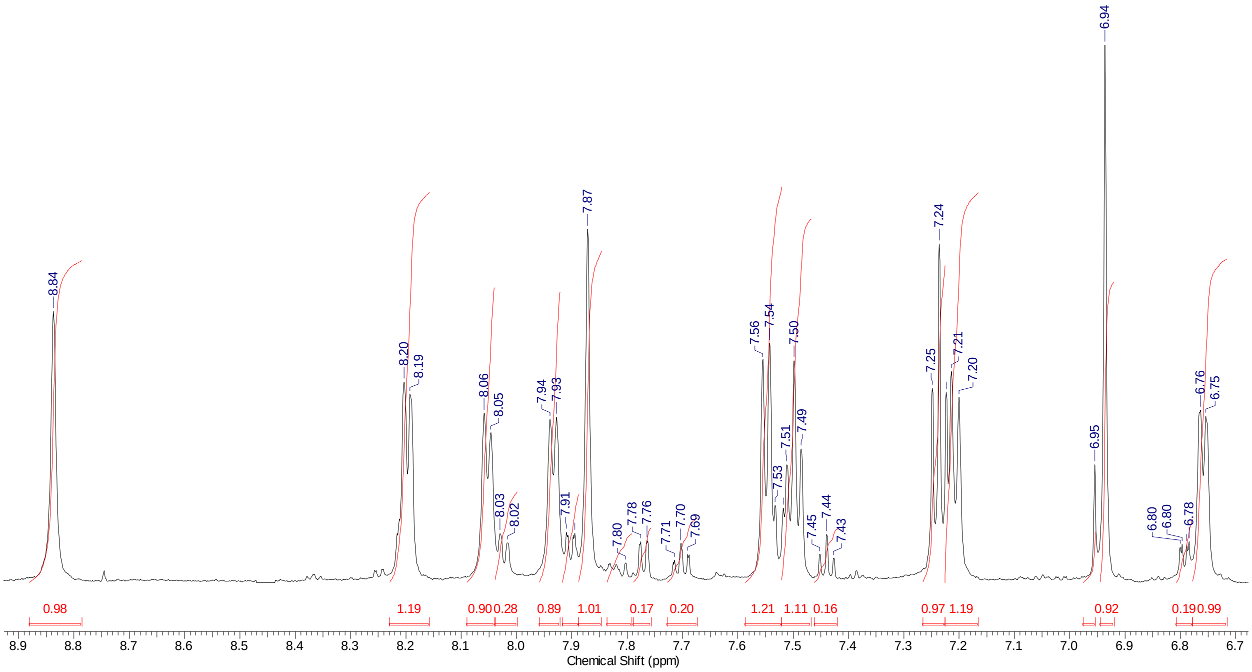

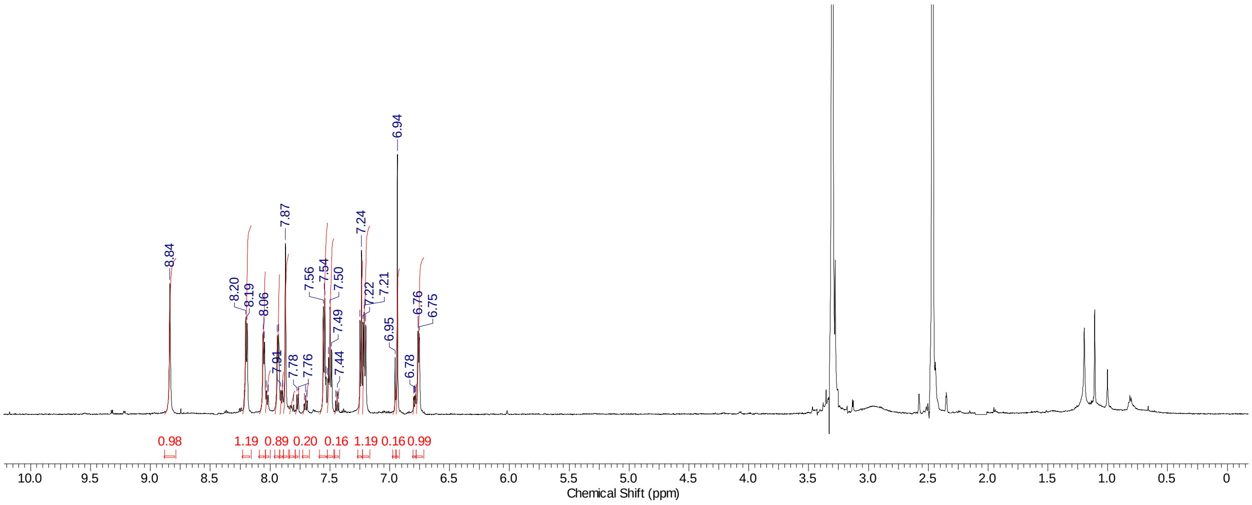

Copies of 2D NMR spectra

LK3756_dqf_cosy_pfg-1 (1).jdf

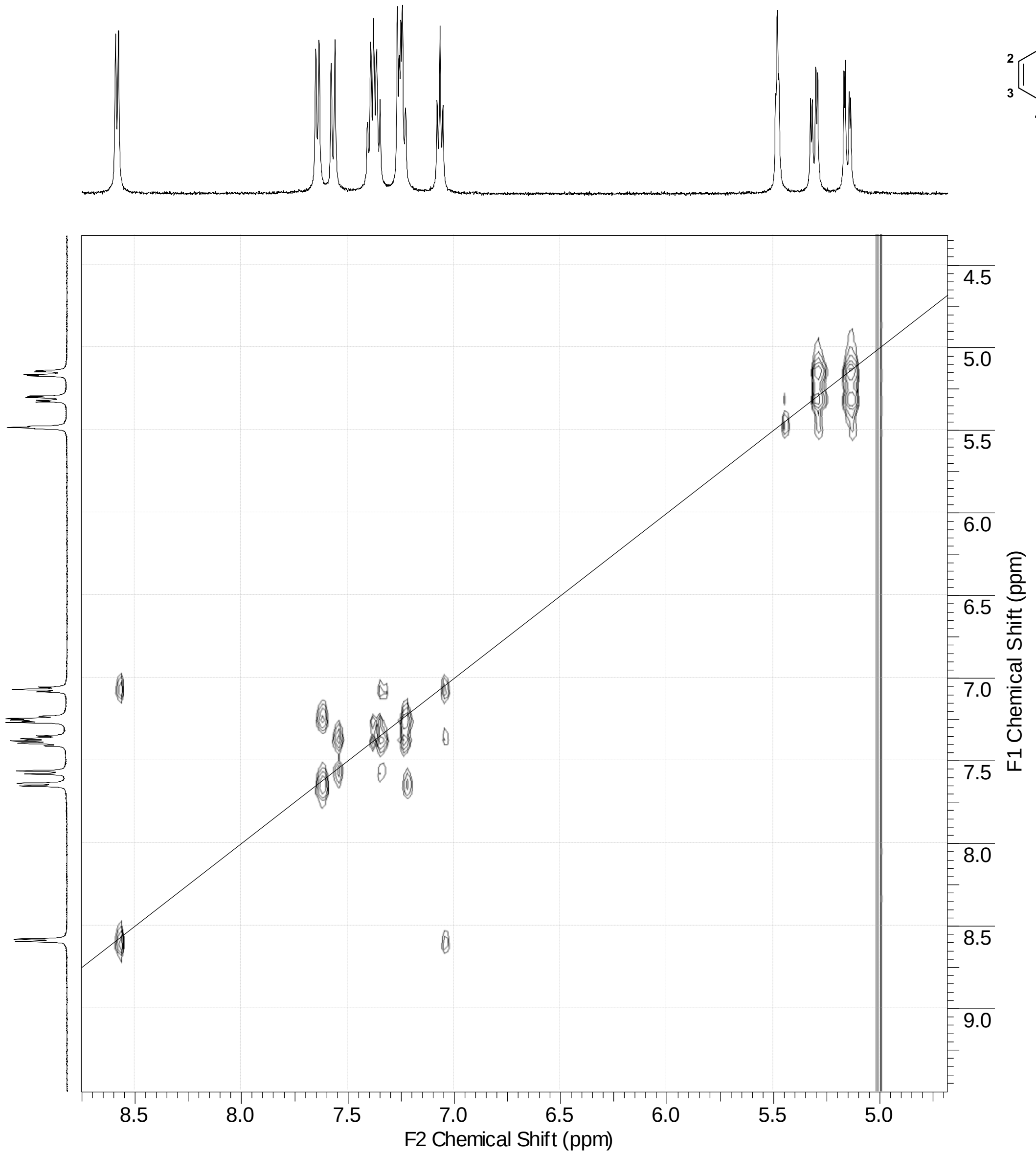

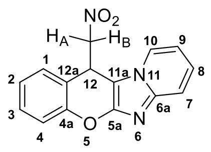

5a COSY

LK3756_hmqc_pfg-1.jdf

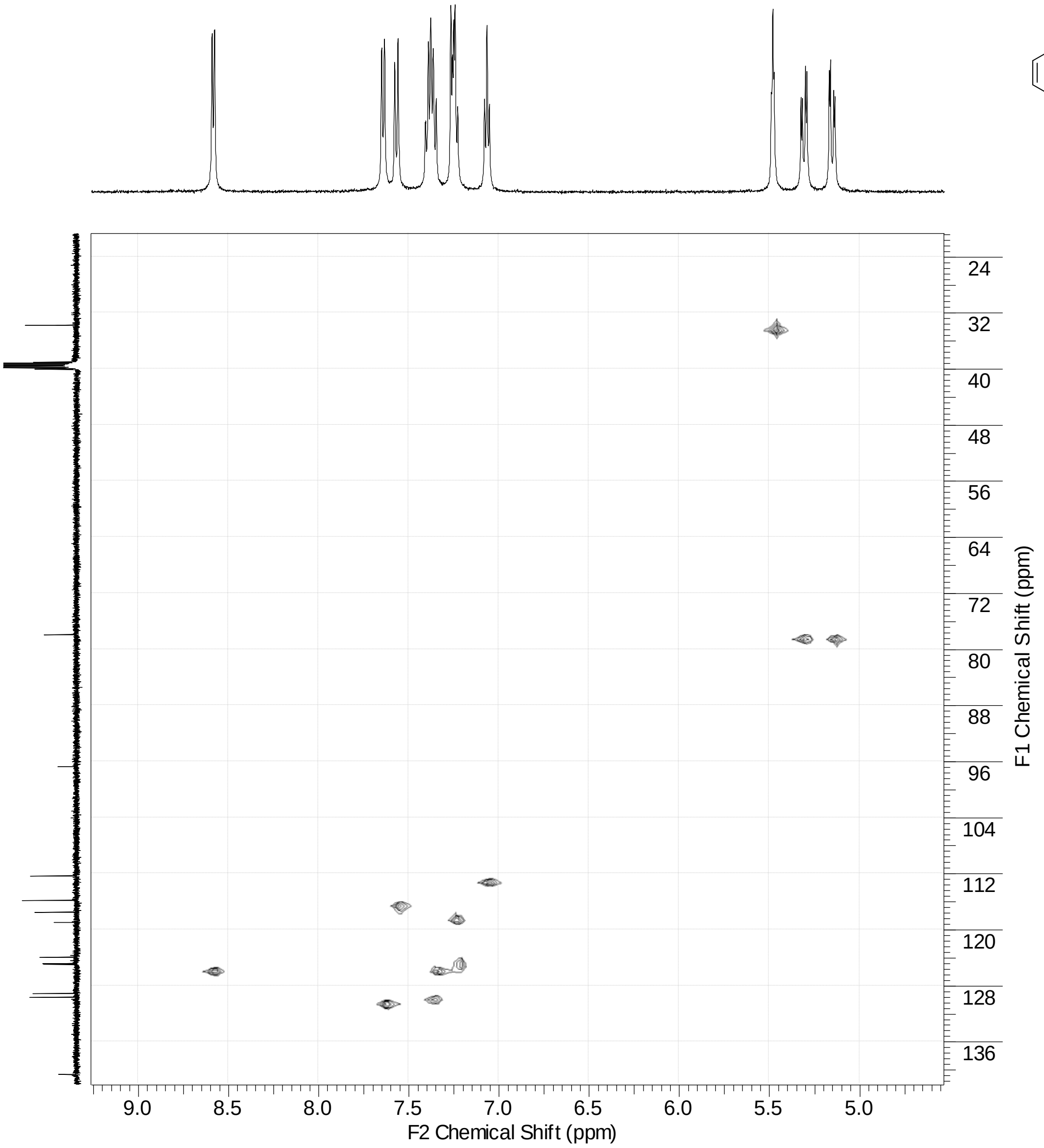

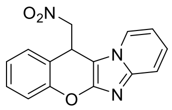

5a HMQC

LK3756_hmqc_pfg-1.jdf

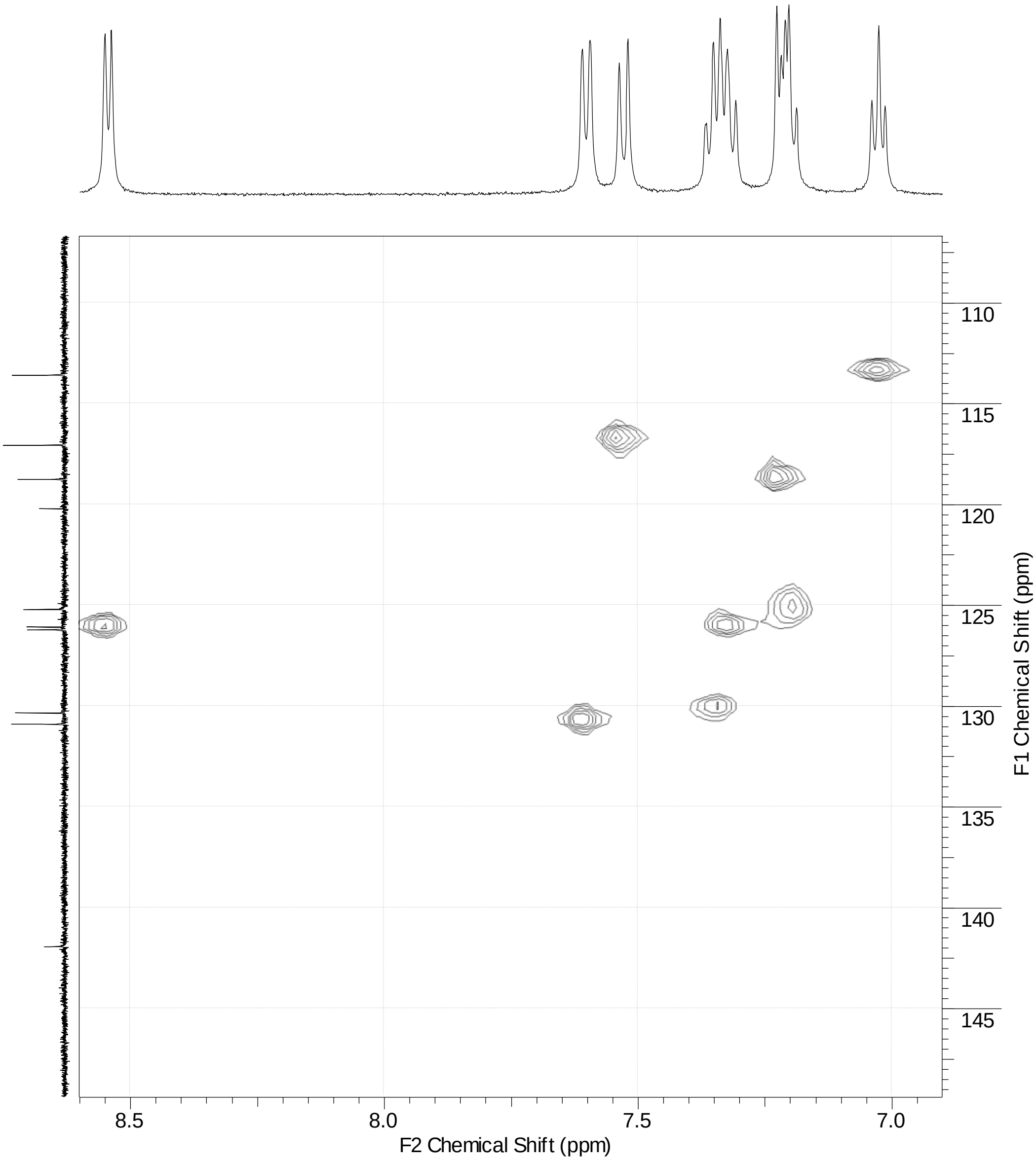

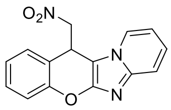

5a HMQC

LK3756_hmbc_pfg-1.jdf

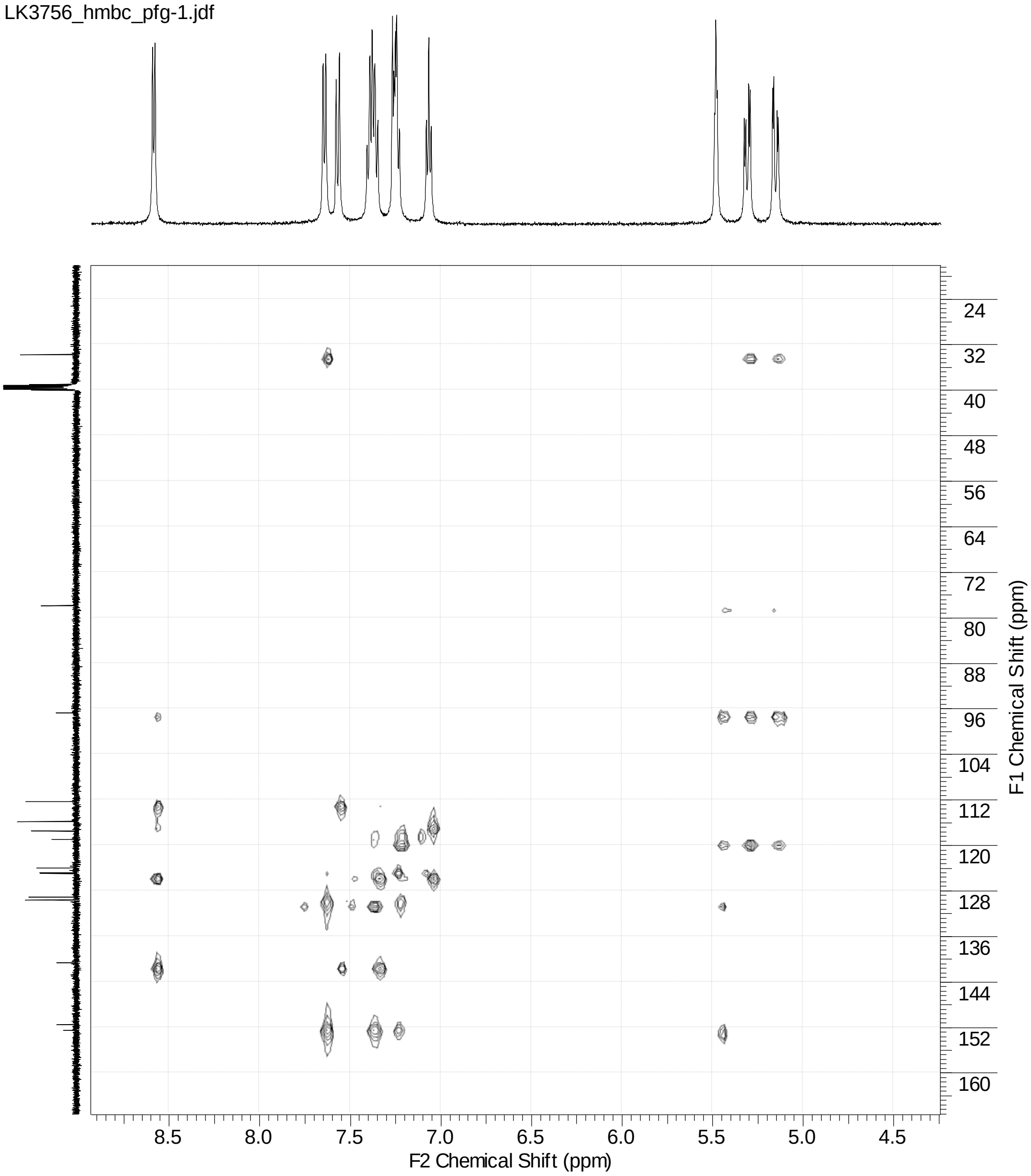

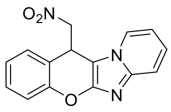

5a HMBC

LK3756_hmbc_pfg-1.jdf

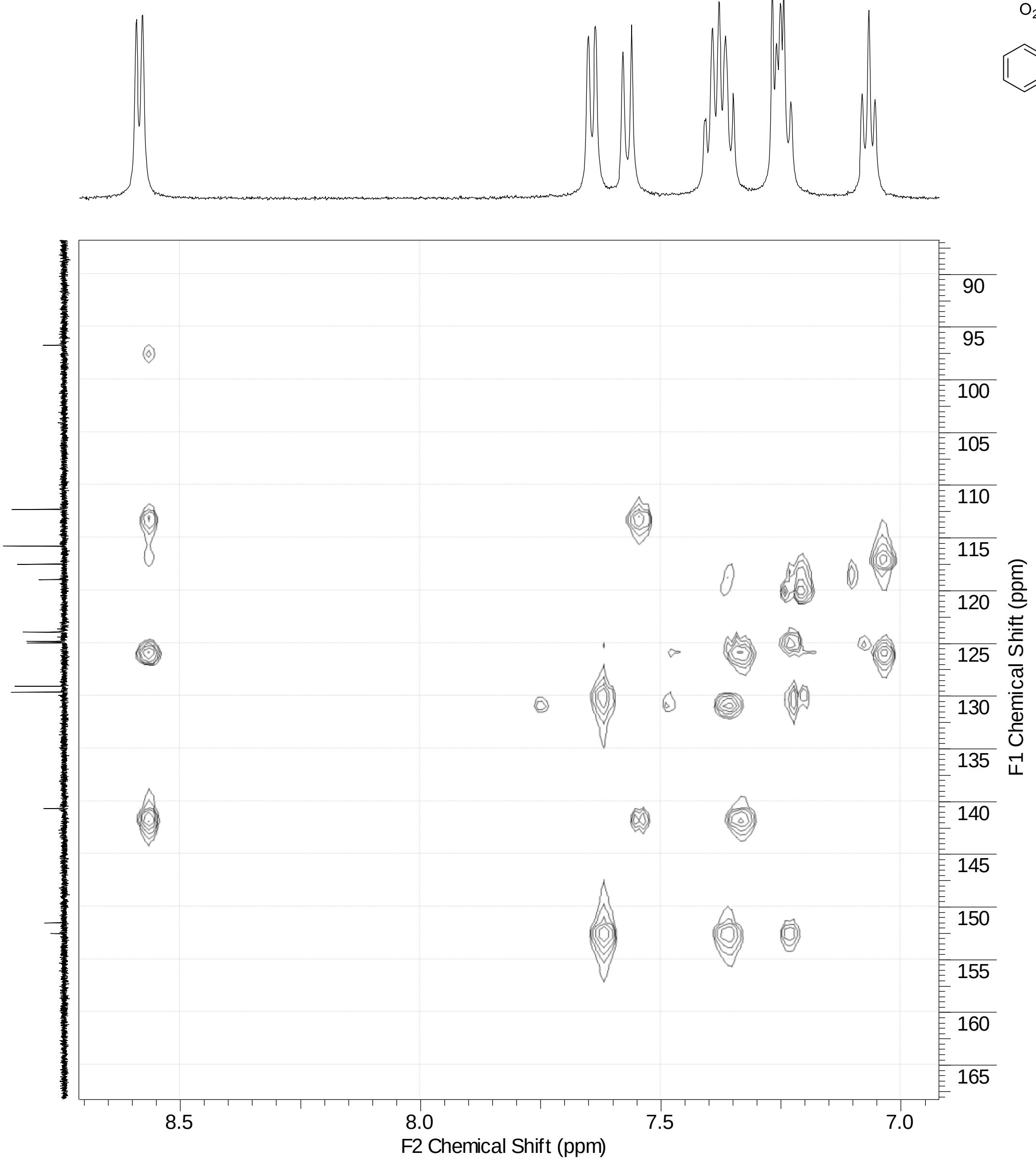

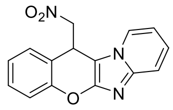

5a HMBC

LK-3772.002.001.2rr.esp

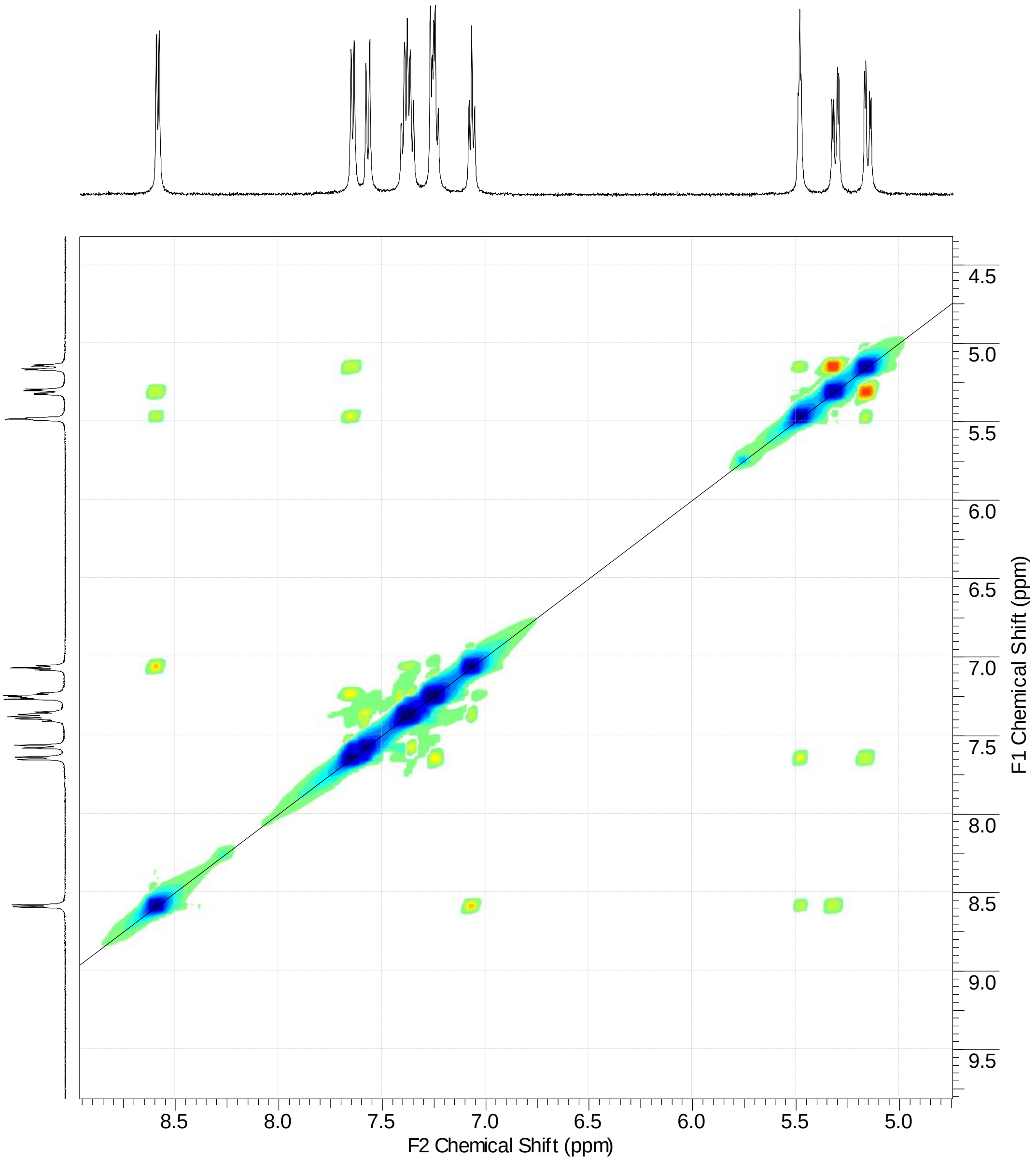

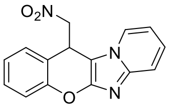

5a NOESY

LK-3773.002.001.2rr.esp

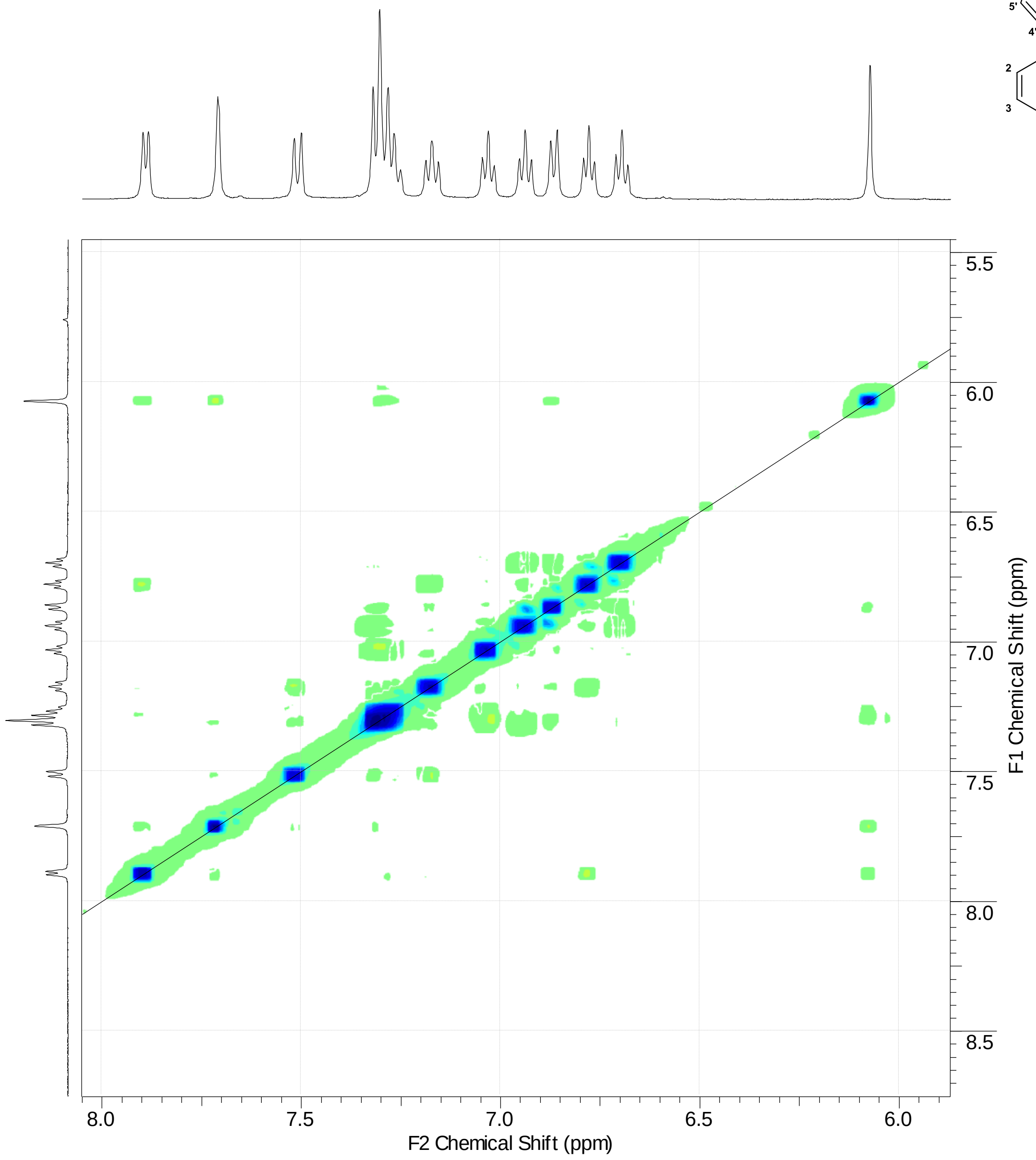

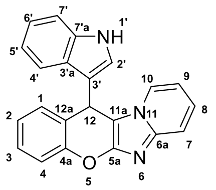

6a NOESY

LK-3773.002.001.2rr.esp

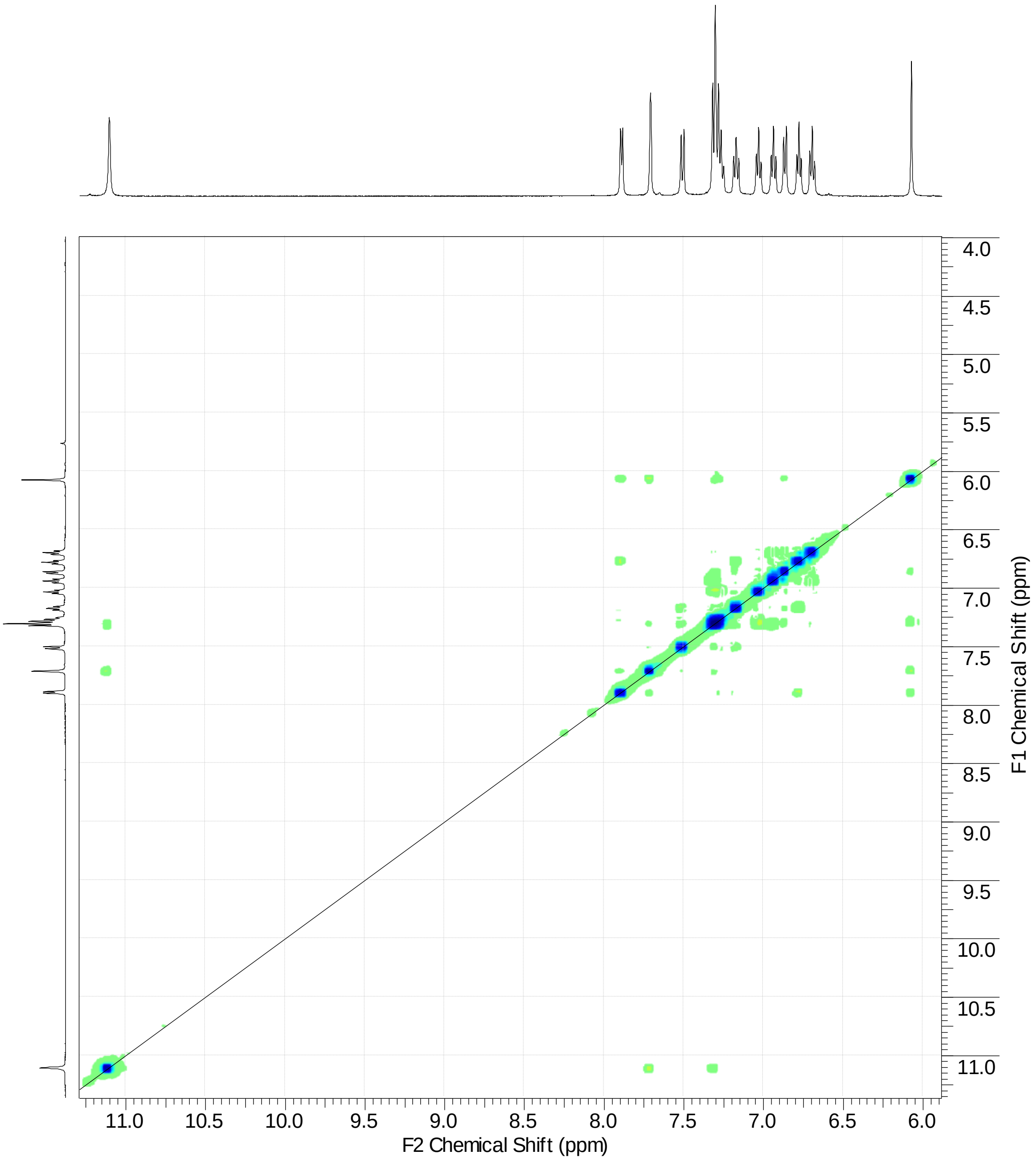

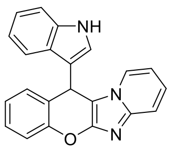

6a NOESY

LK-3773.003.001.2rr.esp

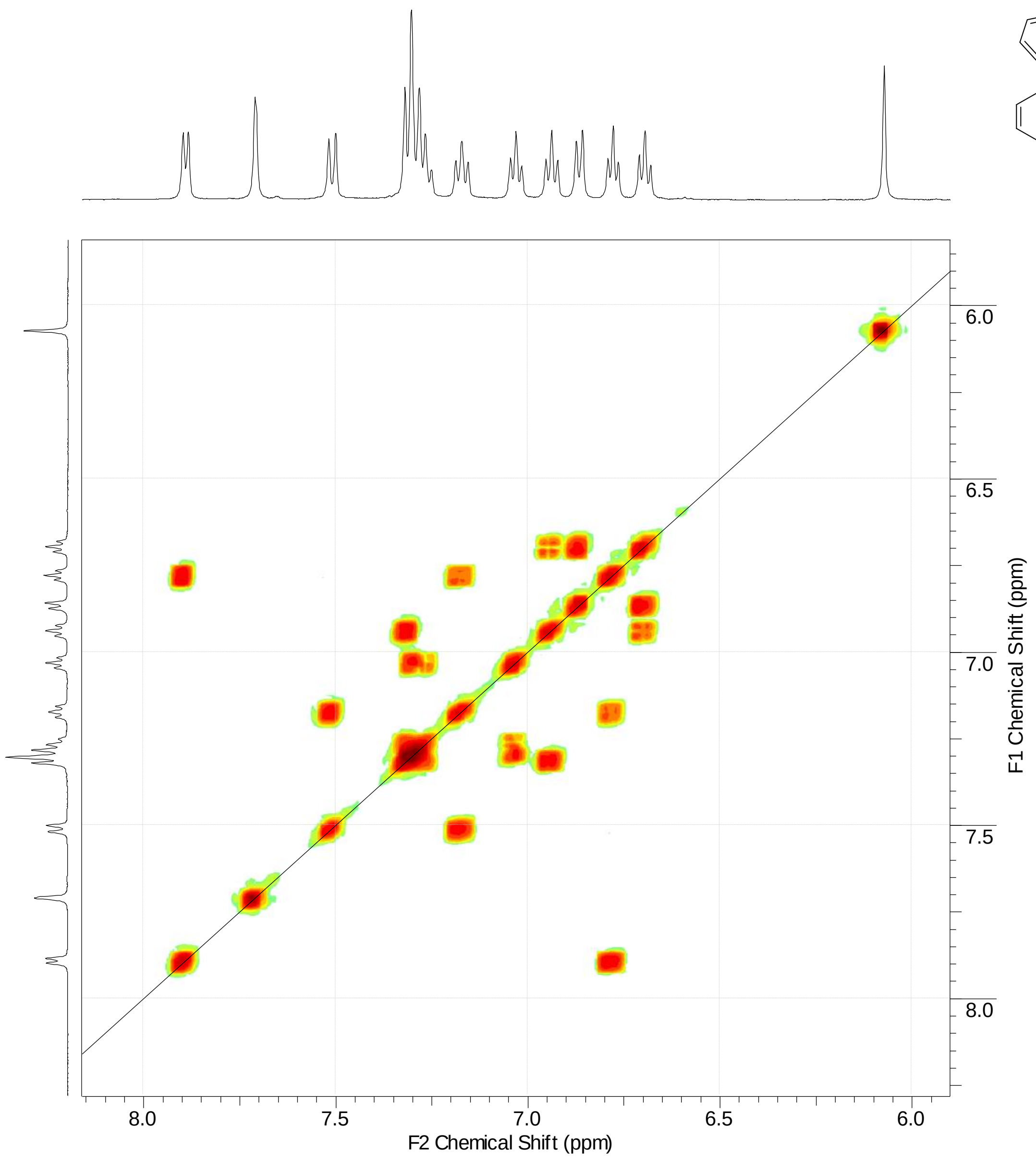

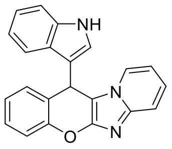

6a COSY

LK-3773.003.001.2rr.esp

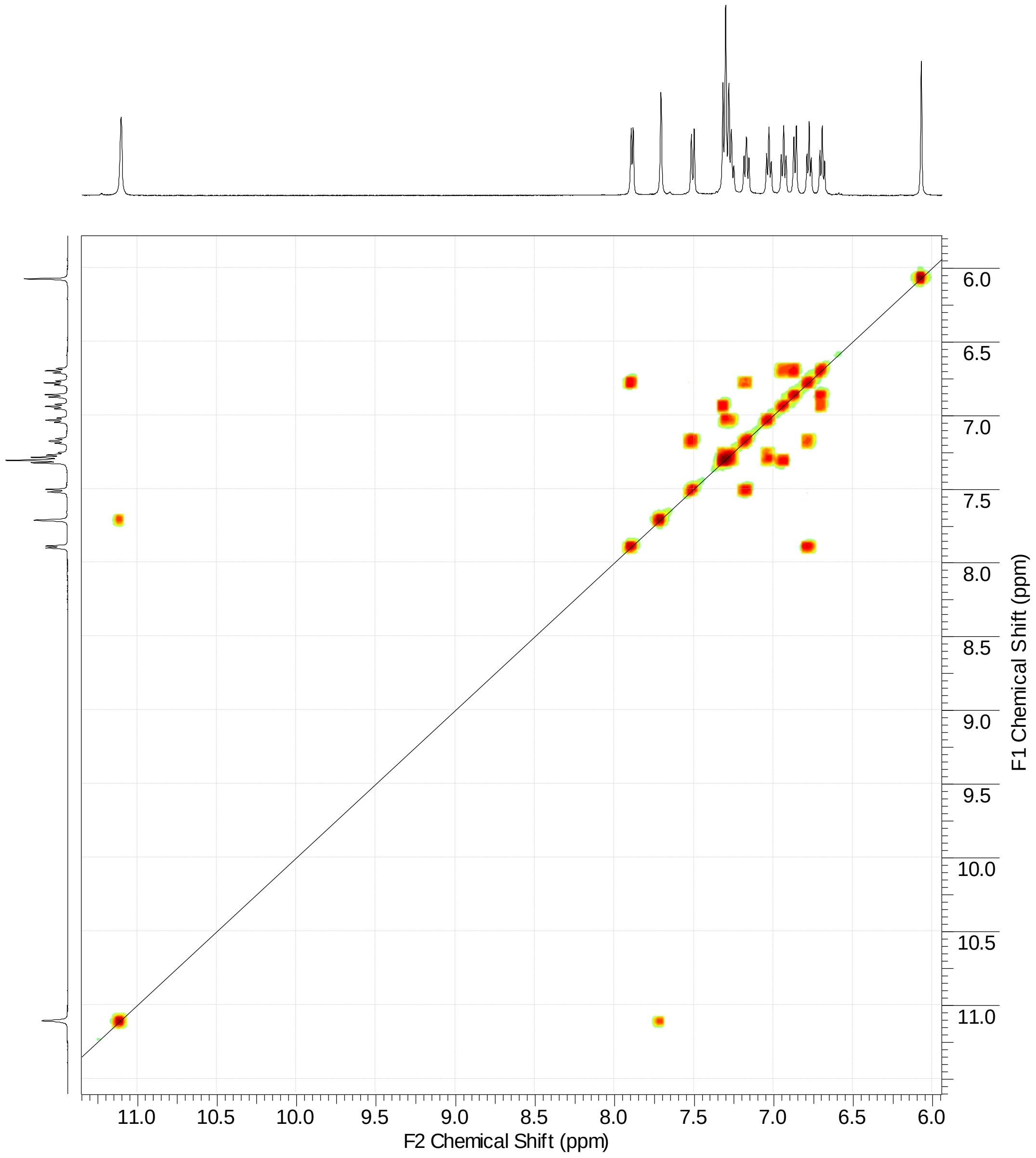

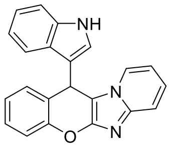

6a COSY

LK-3773.004.001.2rr.esp

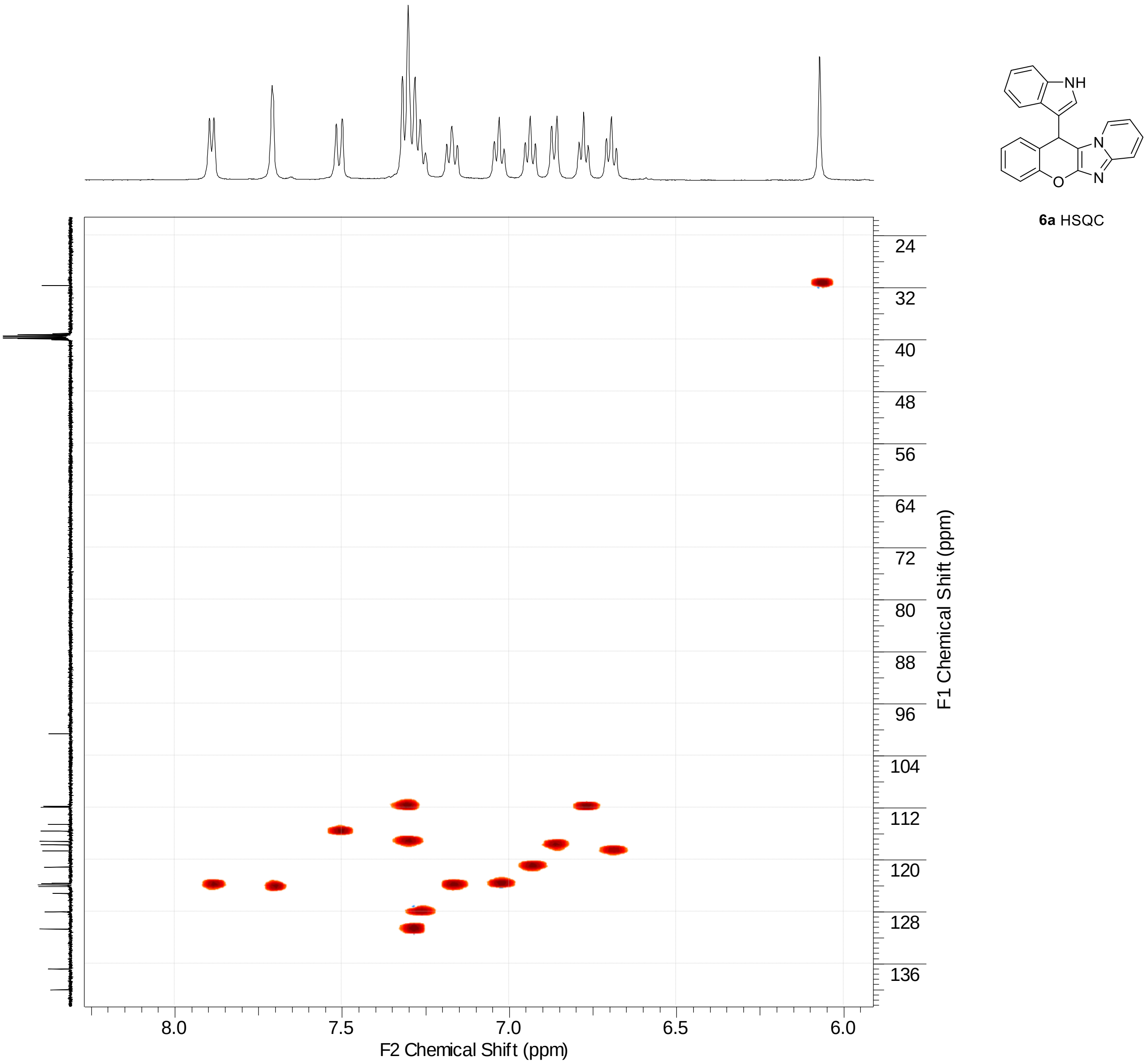

6a HSQC

LK-3773.004.001.2rr.esp

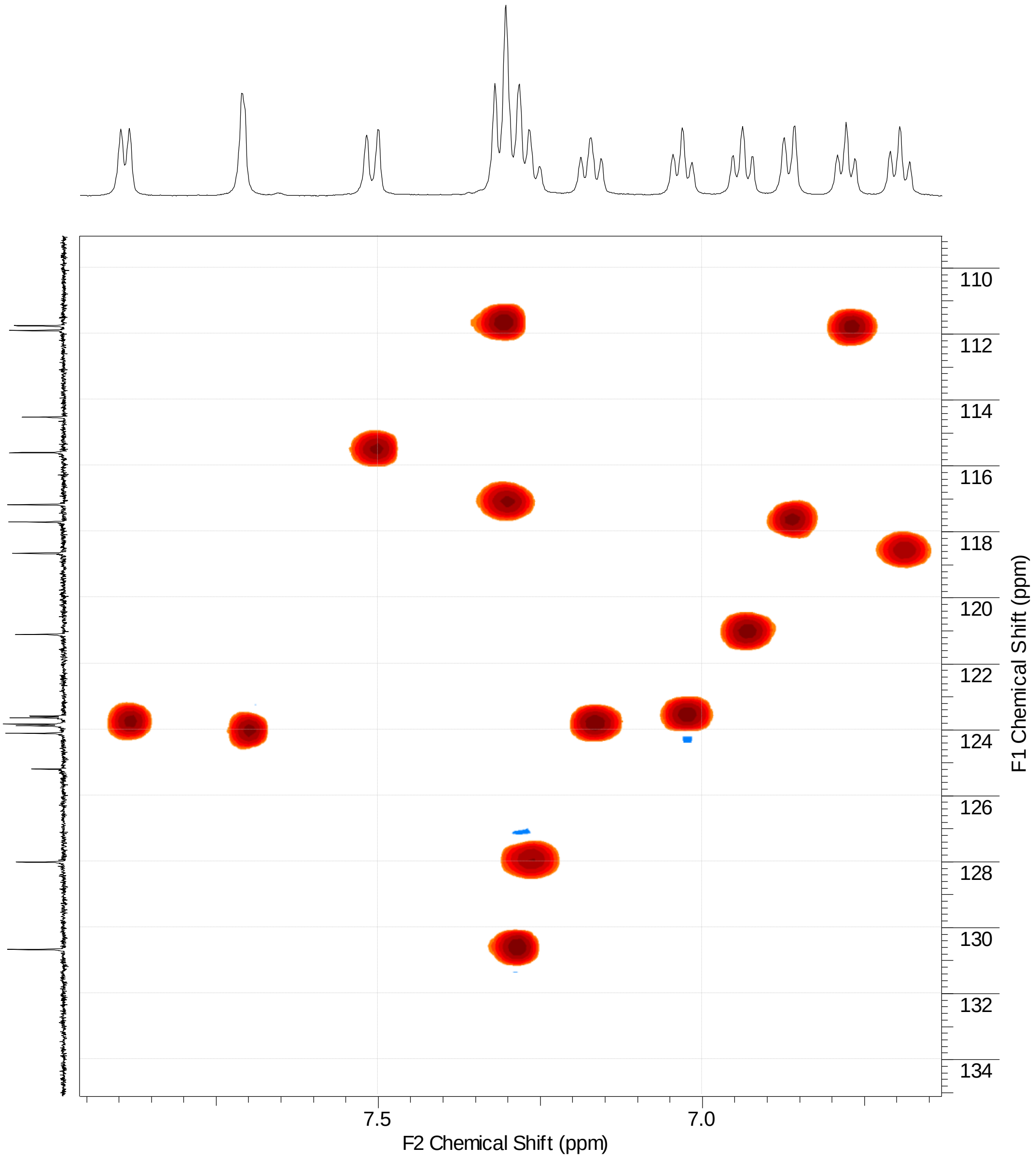

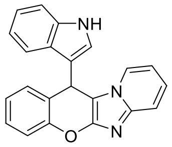

6a HSQC

LK-3773.004.001.2rr.esp

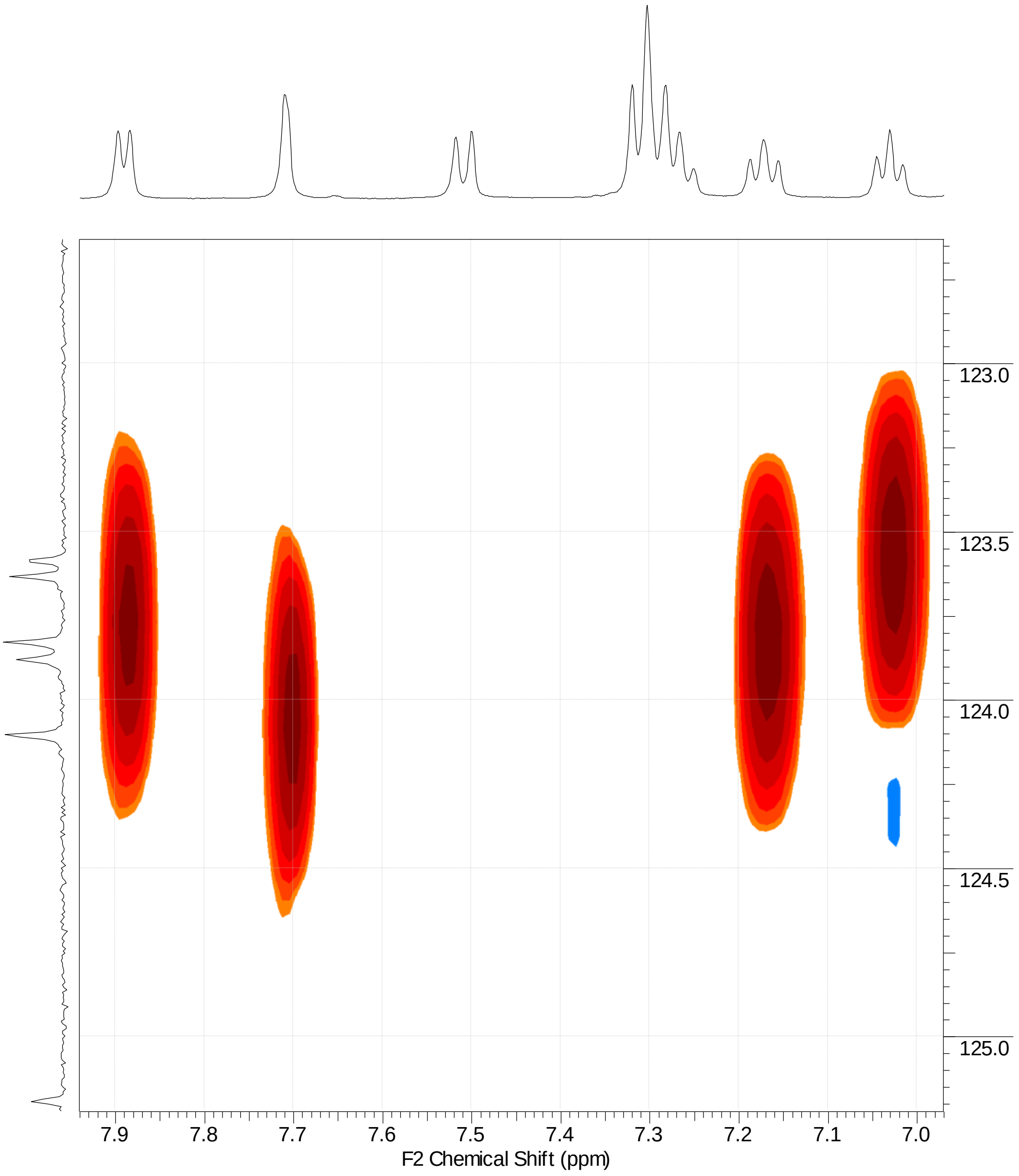

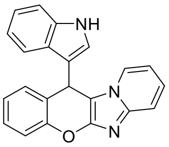

6a HSQC

LK-3773.005.001.2rr.esp

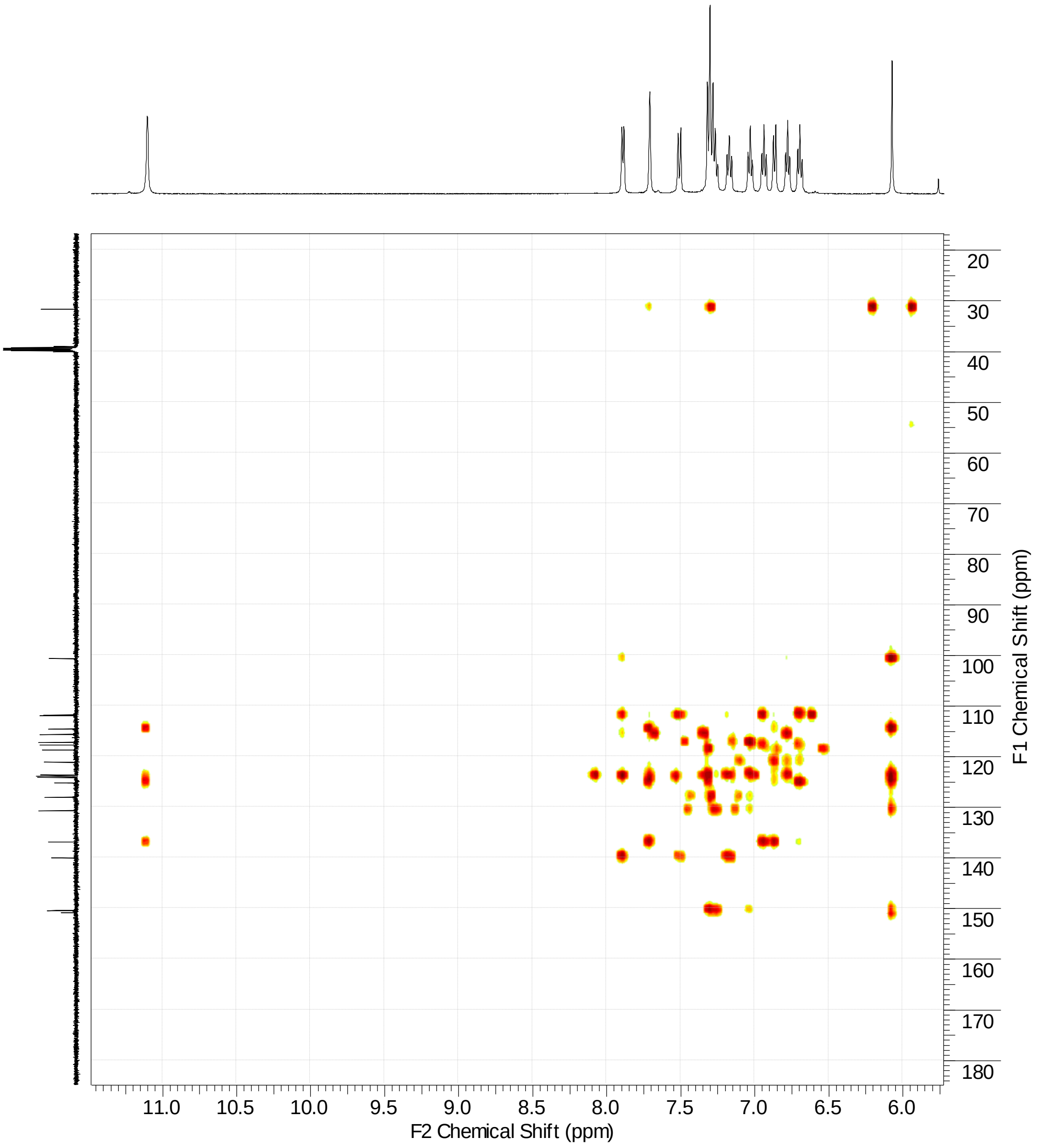

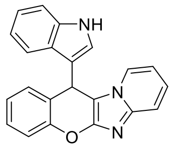

6a HMBC

LK-3773.005.001.2rr.esp

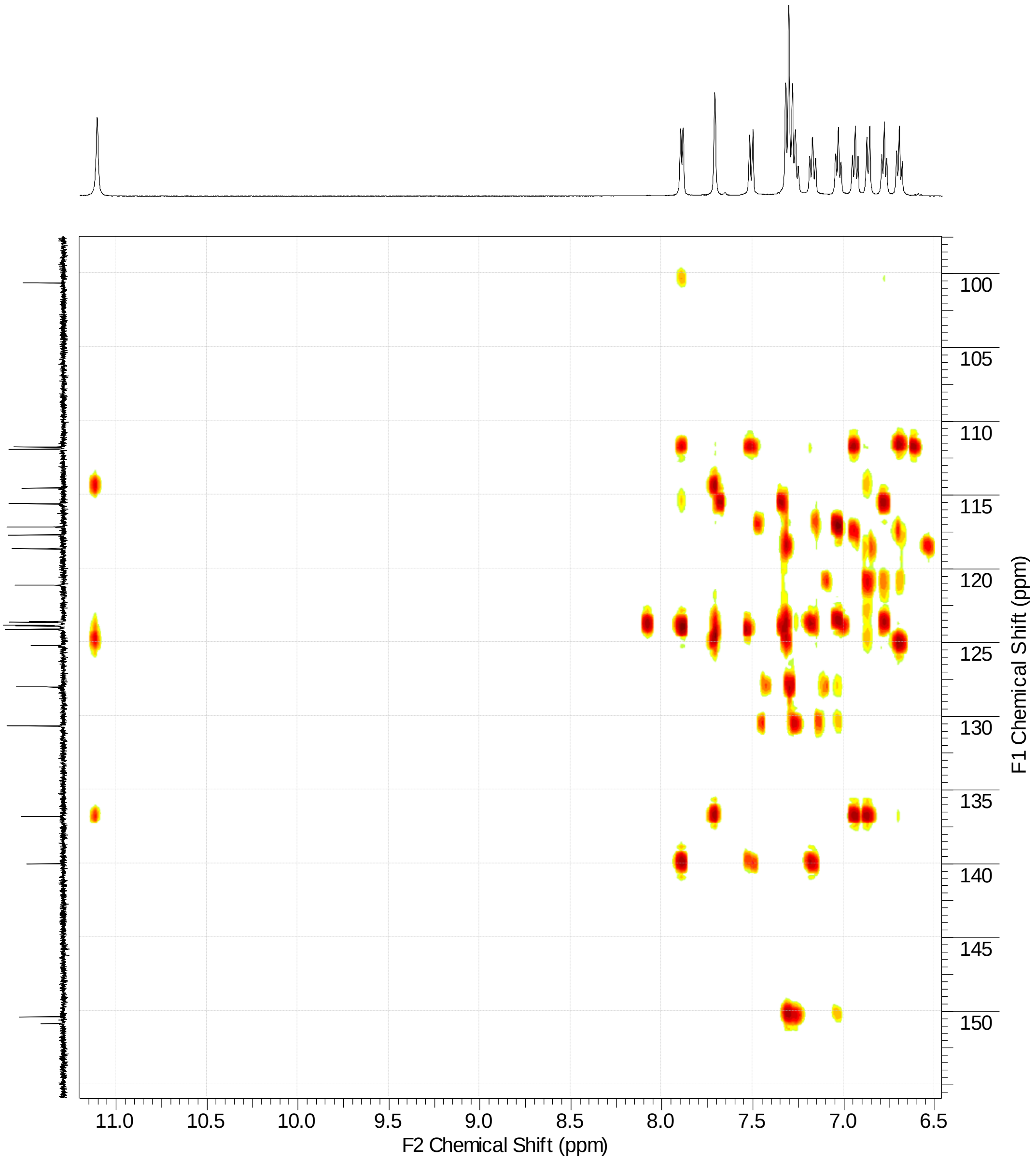

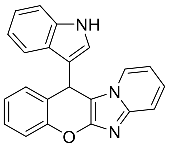

6a HMBC

LK-3773.005.001.2rr.esp

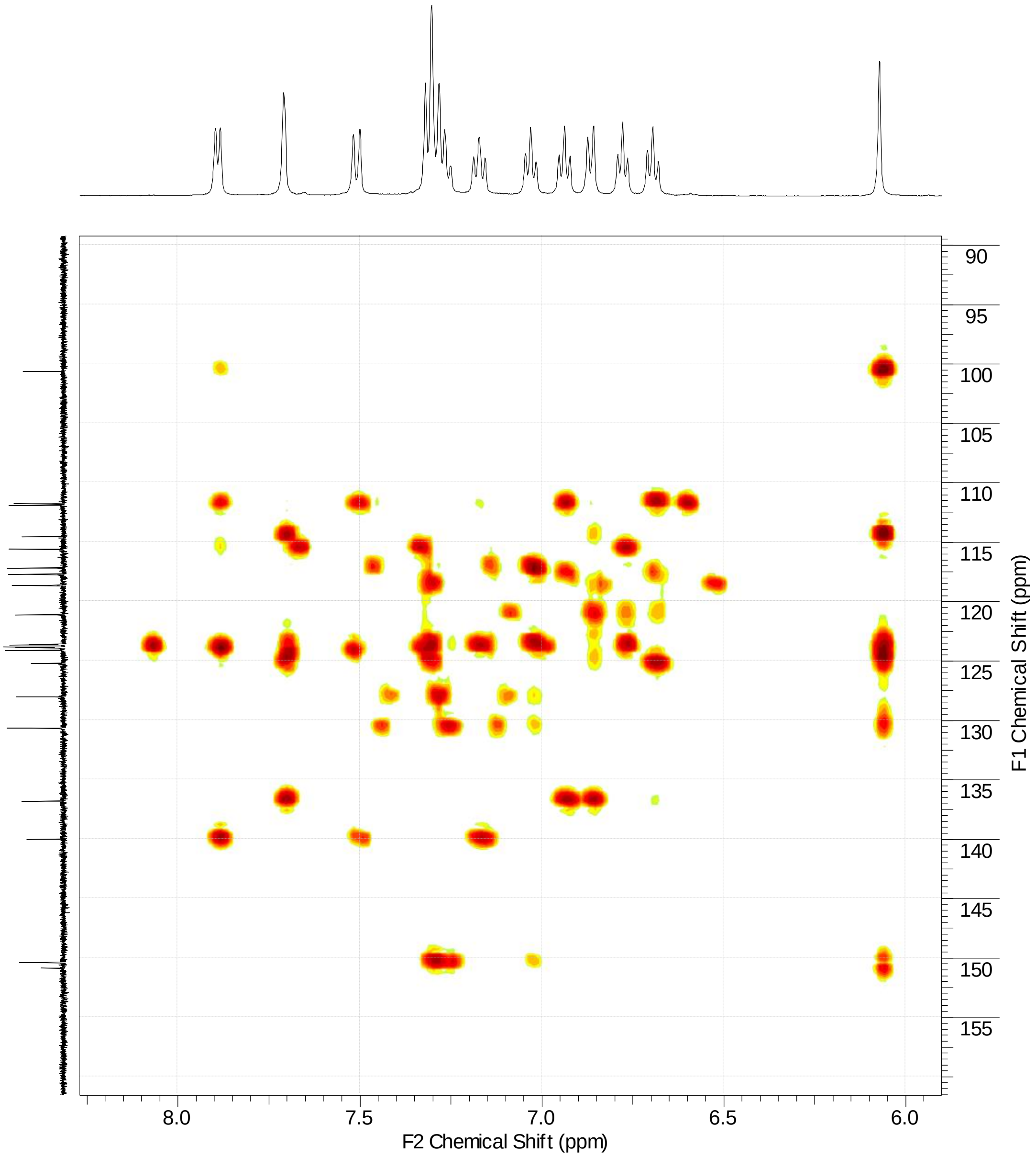

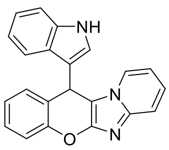

6a HMBC

LK-3773.005.001.2rr.esp

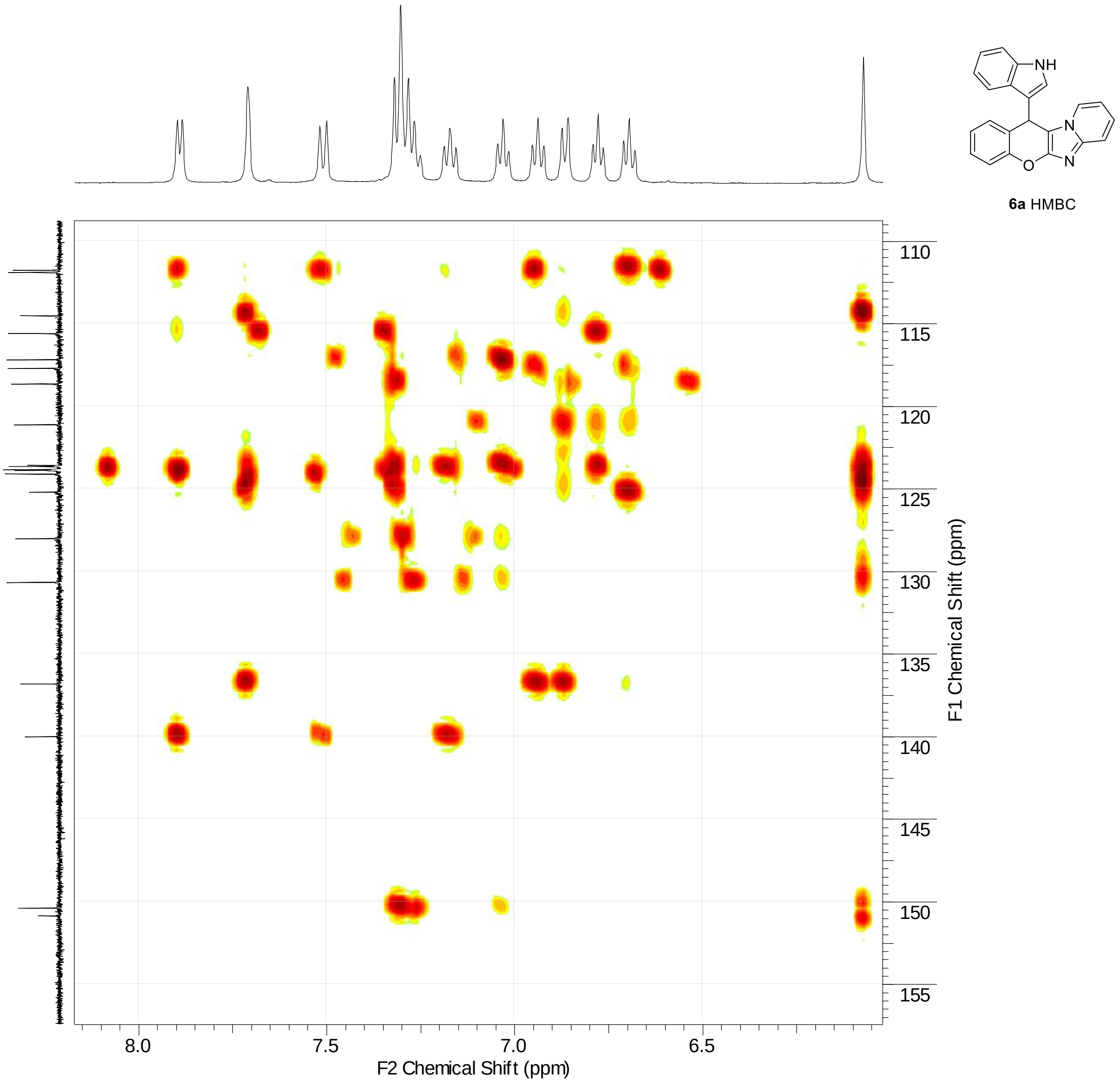

6a HMBC

LK-3773.005.001.2rr.esp

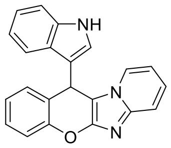

6a HMBC

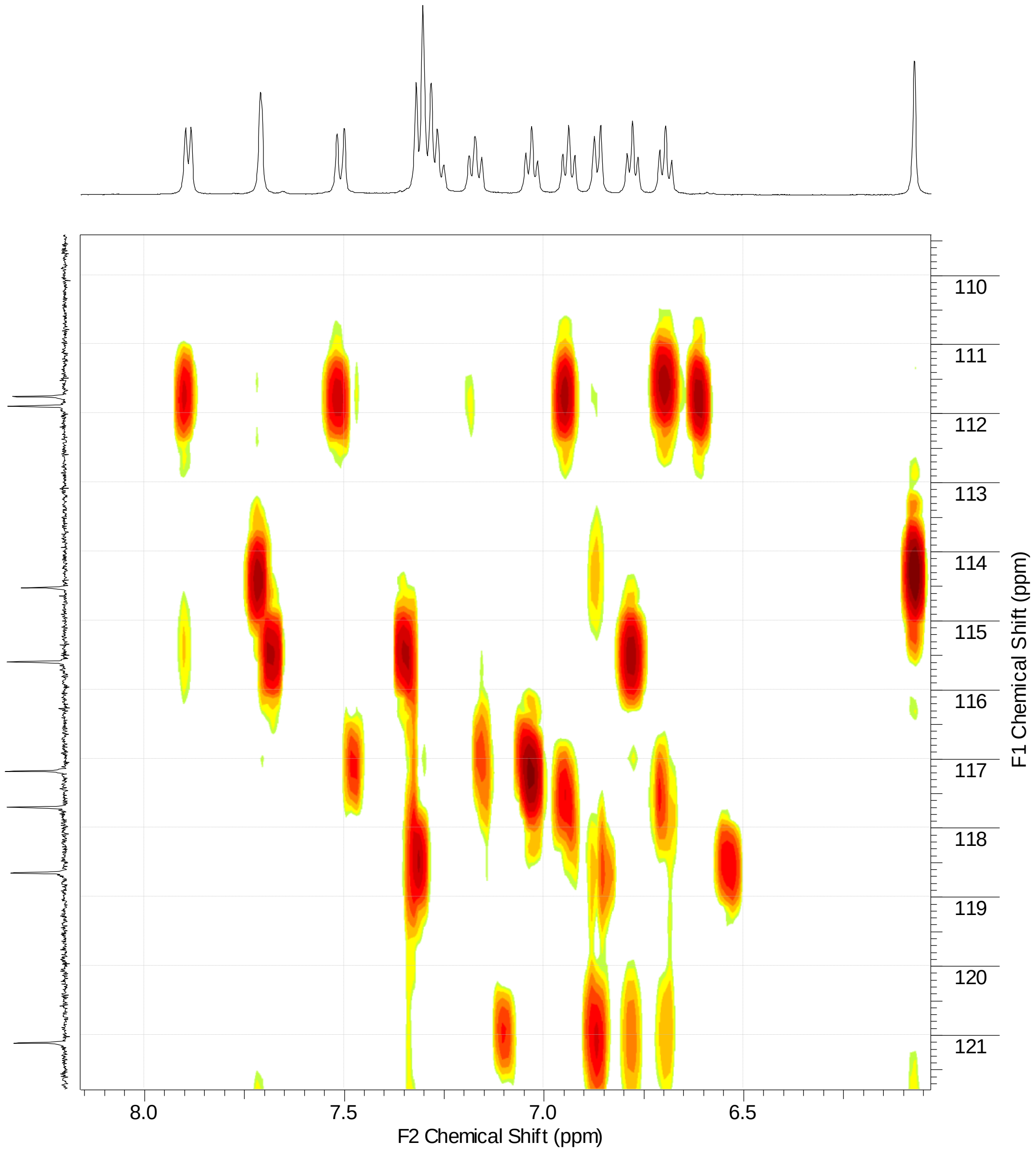

LK-3773.005.001.2rr.esp

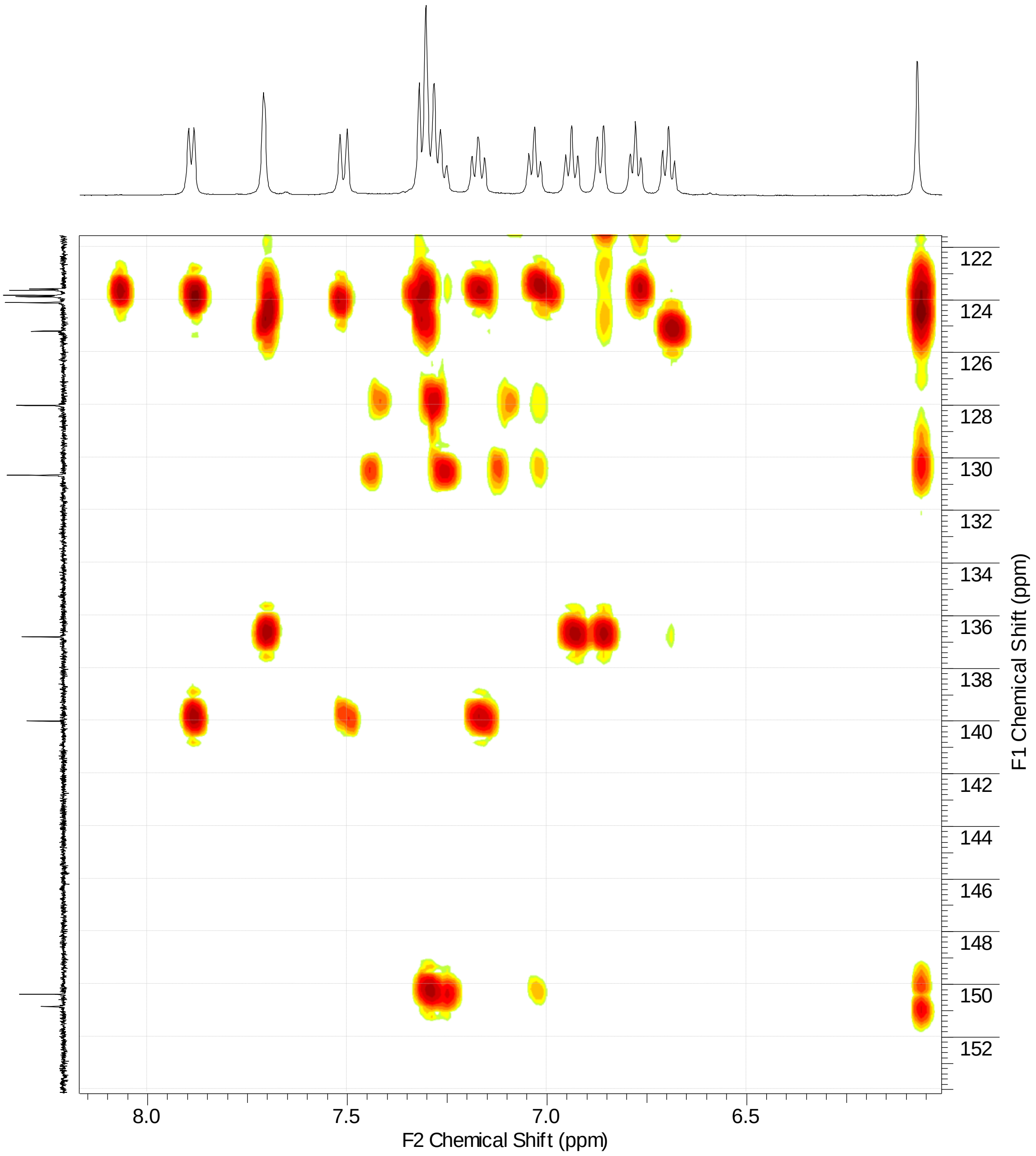

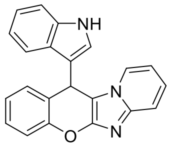

6a HMBC

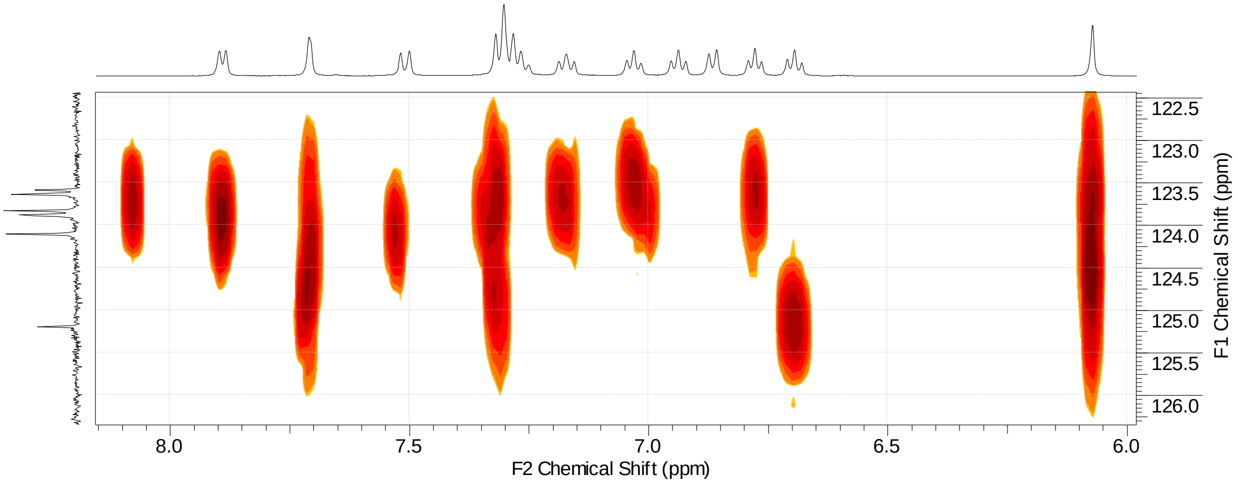

X-Ray diffraction study of compound 7b

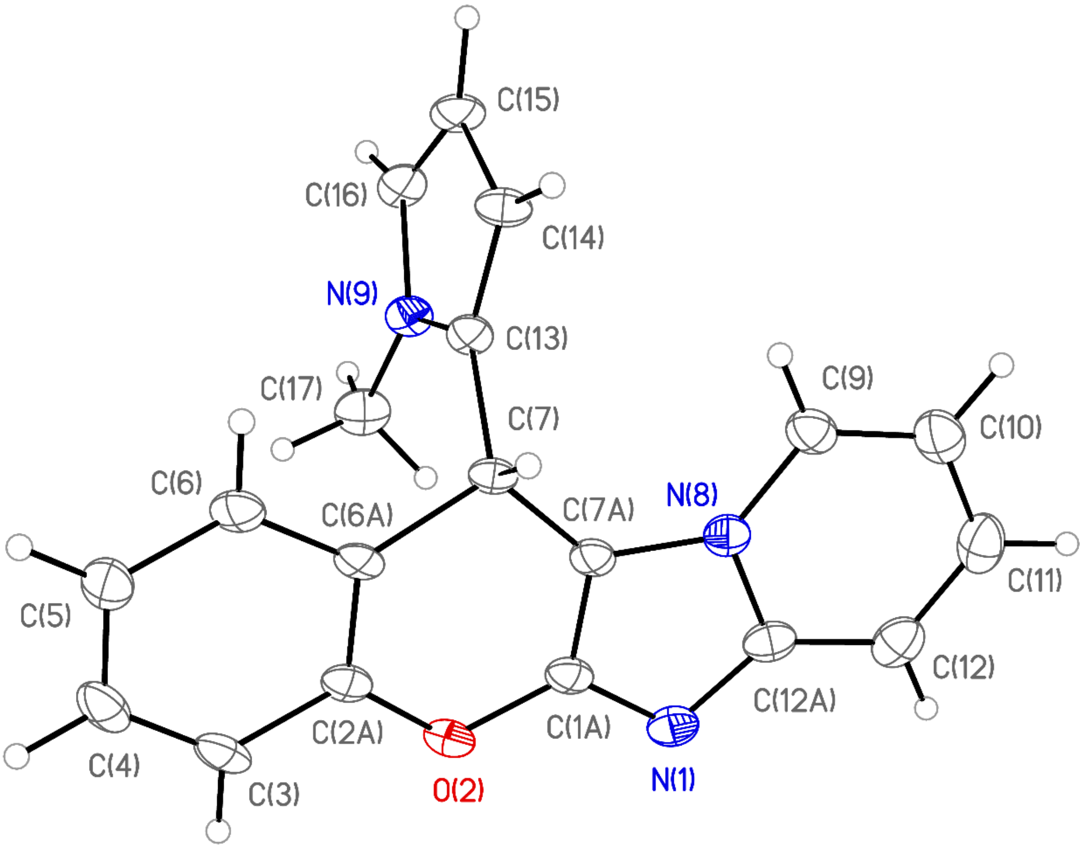

Fig. 1 General view of the molecule **7b** in crystal. Anisotropic displacement parameters are drawn at 50% probability.

Experimental:

X-ray diffraction data for **7b** were collected on a Bruker APEX DUO diffractometer (λ(MoKα) = 0.71073 Å, ω-scans, $2\theta < 56°$). Yellow crystals of $C_{19}H_{15}N_3O$ at 120(2) K are triclinic, space group P-1, a = 6.3429(17), b = 7.690(2), c = 15.591(4) Å, α = 76.642(5), β = 80.743(5), γ = 86.295(5)°, V = 730.0(3) Å^3, Z = 2, d_{calc} = 1.371 g cm^{-3}. Intensities of 3546 independent reflections (R_{int} = 0.0454) out of 8375 collected were used in structure solution and refinement.

The structure was solved by direct methods and refined by the full-matrix least-squares technique against F^2 in anisotropic approximation. Hydrogen atoms were placed in calculated positions and refined in the riding model with U_{iso}(H) equal to 1.5 U_{eq}(Cm) and 1.2 U_{eq}(Ci) of the connected methyl and other carbon atoms. The refinement converged to R_1 = 0.0568 (calculated for 2476 observed reflections with $I>2\sigma(I)$), wR_2 = 0.1430 and GOF = 0.982. All calculations were performed with SHELX software package [SH]. Atomic coordinates, bond lengths and angles and thermal parameters have been deposited at the Cambridge Crystallographic Data Center (CCDC), reference number 1849215.

[SH] G. M. Sheldrick, *Acta Crystallogr. C*, 2015, 71, 3–8, doi: 10.1107/S2053229614024218

Table 1. Crystal data and structure refinement for **7b**.

Identification code	**7b**	
Empirical formula	C19 H15 N3 O	
Formula weight	301.34	
Temperature	120(2) K	
Wavelength	0.71073 Å	
Crystal system	Triclinic	
Space group	P-1	
Unit cell dimensions	a = 6.3429(17) Å	α = 76.642(5)°.
	b = 7.690(2) Å	β = 80.743(5)°.
	c = 15.591(4) Å	γ = 86.295(5)°.
Volume	730.0(3) $Å^3$	
Z	2	
Density (calculated)	1.371 Mg/m^3	
Absorption coefficient	0.087 mm^{-1}	
F(000)	316	
Crystal size	0.270 x 0.230 x 0.080 mm^3	
Theta range for data collection	2.716 to 28.087°.	
Index ranges	-8<=h<=8, -10<=k<=10, -20<=l<=20	
Reflections collected	8375	
Independent reflections	3546 [R(int) = 0.0454]	
Completeness to theta = 25.242°	99.9 %	
Absorption correction	Semi-empirical from equivalents	
Max. and min. transmission	0.862 and 0.660	
Refinement method	Full-matrix least-squares on F^2	
Data / restraints / parameters	3546 / 0 / 209	
Goodness-of-fit on F^2	0.982	
Final R indices for 2476 refl. with [I>2sigma(I)]	R1 = 0.0568, wR2 = 0.1295	
R indices (all data)	R1 = 0.0821, wR2 = 0.1430	
Extinction coefficient	n/a	
Largest diff. peak and hole	0.387 and -0.325 e.$Å^{-3}$	

Table 2. Atomic coordinates (x 10^4) and equivalent isotropic displacement parameters ($Å^2$x 10^3) for **7b**. U(eq) is defined as one third of the trace of the orthogonalized U^{ij} tensor.

	x	y	z	U(eq)
O(2)	3762(2)	6045(2)	2252(1)	24(1)
N(1)	2393(3)	4768(2)	3750(1)	23(1)
N(8)	5415(2)	3292(2)	4156(1)	20(1)
N(9)	8014(2)	1466(2)	2012(1)	20(1)
C(1A)	4023(3)	4995(2)	3070(1)	20(1)
C(2A)	5624(3)	6333(2)	1643(1)	20(1)
C(3)	5379(3)	7481(2)	828(1)	25(1)
C(4)	7112(3)	7862(2)	168(1)	27(1)
C(5)	9109(3)	7105(2)	311(1)	27(1)
C(6A)	7622(3)	5544(2)	1804(1)	18(1)
C(6)	9335(3)	5954(2)	1121(1)	22(1)
C(7A)	5900(3)	4130(2)	3268(1)	19(1)
C(7)	7972(3)	4217(2)	2667(1)	18(1)
C(9)	6681(3)	2268(2)	4729(1)	24(1)
C(10)	5805(4)	1626(3)	5585(1)	29(1)
C(11)	3639(4)	1992(3)	5885(1)	31(1)
C(12A)	3265(3)	3711(2)	4428(1)	23(1)
C(12)	2378(3)	3023(3)	5316(1)	28(1)
C(13)	8884(3)	2433(2)	2502(1)	18(1)
C(14)	10674(3)	1514(2)	2773(1)	23(1)
C(15)	10916(3)	-66(2)	2434(1)	26(1)
C(16)	9273(3)	-47(2)	1968(1)	24(1)
C(17)	6103(3)	1906(3)	1593(1)	27(1)

Table 3. Bond lengths [Å] and angles [°] for **7b**.

O(2)-C(1A)	1.371(2)
O(2)-C(2A)	1.386(2)
N(1)-C(1A)	1.345(2)
N(1)-C(12A)	1.348(3)
N(8)-C(9)	1.375(2)
N(8)-C(7A)	1.380(2)
N(8)-C(12A)	1.401(2)
N(9)-C(13)	1.375(2)
N(9)-C(16)	1.377(2)
N(9)-C(17)	1.453(3)
C(1A)-C(7A)	1.370(3)
C(2A)-C(3)	1.395(3)
C(2A)-C(6A)	1.403(3)
C(3)-C(4)	1.375(3)
C(4)-C(5)	1.389(3)
C(5)-C(6)	1.387(3)
C(6A)-C(6)	1.390(3)
C(6A)-C(7)	1.528(3)
C(7A)-C(7)	1.481(3)
C(7)-C(13)	1.516(2)
C(9)-C(10)	1.350(3)
C(10)-C(11)	1.410(3)
C(11)-C(12)	1.370(3)
C(12A)-C(12)	1.402(3)
C(13)-C(14)	1.371(3)
C(14)-C(15)	1.423(3)
C(15)-C(16)	1.360(3)
C(1A)-O(2)-C(2A)	114.71(14)
C(1A)-N(1)-C(12A)	103.25(16)
C(9)-N(8)-C(7A)	130.46(17)
C(9)-N(8)-C(12A)	122.52(16)
C(7A)-N(8)-C(12A)	107.00(15)
C(13)-N(9)-C(16)	108.60(16)
C(13)-N(9)-C(17)	127.71(15)
C(16)-N(9)-C(17)	123.69(16)
N(1)-C(1A)-C(7A)	115.08(17)
N(1)-C(1A)-O(2)	120.69(16)
C(7A)-C(1A)-O(2)	124.23(17)
O(2)-C(2A)-C(3)	114.71(16)
O(2)-C(2A)-C(6A)	124.30(16)
C(3)-C(2A)-C(6A)	120.99(18)
C(4)-C(3)-C(2A)	120.01(18)
C(3)-C(4)-C(5)	120.19(18)
C(6)-C(5)-C(4)	119.42(19)
C(6)-C(6A)-C(2A)	117.39(17)
C(6)-C(6A)-C(7)	119.15(16)
C(2A)-C(6A)-C(7)	123.42(16)
C(5)-C(6)-C(6A)	122.00(18)
C(1A)-C(7A)-N(8)	103.57(16)
C(1A)-C(7A)-C(7)	126.80(17)
N(8)-C(7A)-C(7)	129.50(16)
C(7A)-C(7)-C(13)	114.93(14)
C(7A)-C(7)-C(6A)	106.15(14)
C(13)-C(7)-C(6A)	112.15(15)
C(10)-C(9)-N(8)	118.50(19)
C(9)-C(10)-C(11)	120.9(2)
C(12)-C(11)-C(10)	120.71(19)
N(1)-C(12A)-N(8)	111.10(17)
N(1)-C(12A)-C(12)	130.67(19)
N(8)-C(12A)-C(12)	118.23(18)

C(11)-C(12)-C(12A)	119.11(19)
C(14)-C(13)-N(9)	107.87(15)
C(14)-C(13)-C(7)	128.38(16)
N(9)-C(13)-C(7)	123.73(16)
C(13)-C(14)-C(15)	107.73(17)
C(16)-C(15)-C(14)	106.85(17)
C(15)-C(16)-N(9)	108.95(17)

Table 4. Anisotropic displacement parameters ($Å^2 x 10^3$) for **7b**. The anisotropic displacement factor exponent takes the form: $-2p^2[h^2 a^{*2}U^{11} + ... + 2 h k a^* b^* U^{12}]$

	U^{11}	U^{22}	U^{33}	U^{23}	U^{13}	U^{12}
O(2)	24(1)	17(1)	28(1)	-3(1)	-7(1)	5(1)
N(1)	23(1)	17(1)	30(1)	-8(1)	-3(1)	0(1)
N(8)	26(1)	13(1)	23(1)	-6(1)	-3(1)	-1(1)
N(9)	24(1)	11(1)	25(1)	-6(1)	-3(1)	-2(1)
C(1A)	24(1)	12(1)	25(1)	-6(1)	-6(1)	1(1)
C(2A)	25(1)	10(1)	26(1)	-6(1)	-5(1)	0(1)
C(3)	34(1)	12(1)	31(1)	-4(1)	-13(1)	4(1)
C(4)	44(1)	13(1)	26(1)	-1(1)	-11(1)	-1(1)
C(5)	36(1)	16(1)	27(1)	-4(1)	-1(1)	-3(1)
C(6A)	25(1)	6(1)	24(1)	-5(1)	-6(1)	0(1)
C(6)	26(1)	11(1)	29(1)	-6(1)	-4(1)	1(1)
C(7A)	25(1)	12(1)	21(1)	-5(1)	-5(1)	2(1)
C(7)	21(1)	9(1)	24(1)	-5(1)	-4(1)	1(1)
C(9)	29(1)	18(1)	27(1)	-6(1)	-8(1)	1(1)
C(10)	41(1)	21(1)	28(1)	-4(1)	-9(1)	-2(1)
C(11)	43(1)	25(1)	24(1)	-5(1)	1(1)	-9(1)
C(12A)	23(1)	16(1)	31(1)	-10(1)	-4(1)	-1(1)
C(12)	31(1)	22(1)	32(1)	-10(1)	2(1)	-5(1)
C(13)	22(1)	10(1)	23(1)	-4(1)	-2(1)	-1(1)
C(14)	25(1)	12(1)	32(1)	-6(1)	-6(1)	3(1)
C(15)	31(1)	10(1)	33(1)	-4(1)	0(1)	6(1)
C(16)	32(1)	8(1)	29(1)	-5(1)	2(1)	-1(1)
C(17)	28(1)	22(1)	36(1)	-12(1)	-8(1)	-2(1)

Table 5. Hydrogen coordinates (x 10^4) and isotropic displacement parameters ($Å^2$x 10^3) for **7b**.

	x	y	z	U(eq)
H(3A)	4014	7999	728	30
H(4A)	6943	8645	-386	33
H(5A)	10311	7372	-143	32
H(6A)	10703	5431	1211	26
H(7A)	9029	4736	2943	21
H(9A)	8139	2017	4529	29
H(10A)	6660	918	5990	35
H(11A)	3050	1519	6488	38
H(12A)	920	3268	5521	34
H(14A)	11590	1869	3124	27
H(15A)	12015	-961	2516	31
H(16A)	9033	-934	1663	28
H(17A)	4944	2274	2016	41
H(17B)	5681	855	1413	41
H(17C)	6396	2887	1066	41

Table 6. Torsion angles [°] for **7b**.

C(12A)-N(1)-C(1A)-C(7A)	0.6(2)
C(12A)-N(1)-C(1A)-O(2)	-178.71(15)
C(2A)-O(2)-C(1A)-N(1)	172.85(15)
C(2A)-O(2)-C(1A)-C(7A)	-6.3(2)
C(1A)-O(2)-C(2A)-C(3)	-176.64(15)
C(1A)-O(2)-C(2A)-C(6A)	4.1(2)
O(2)-C(2A)-C(3)-C(4)	-179.68(16)
C(6A)-C(2A)-C(3)-C(4)	-0.4(3)
C(2A)-C(3)-C(4)-C(5)	0.1(3)
C(3)-C(4)-C(5)-C(6)	0.4(3)
O(2)-C(2A)-C(6A)-C(6)	179.35(16)
C(3)-C(2A)-C(6A)-C(6)	0.1(2)
O(2)-C(2A)-C(6A)-C(7)	1.8(3)
C(3)-C(2A)-C(6A)-C(7)	-177.37(16)
C(4)-C(5)-C(6)-C(6A)	-0.7(3)
C(2A)-C(6A)-C(6)-C(5)	0.4(3)
C(7)-C(6A)-C(6)-C(5)	178.01(16)
N(1)-C(1A)-C(7A)-N(8)	-0.5(2)
O(2)-C(1A)-C(7A)-N(8)	178.71(15)
N(1)-C(1A)-C(7A)-C(7)	-176.57(16)
O(2)-C(1A)-C(7A)-C(7)	2.7(3)
C(9)-N(8)-C(7A)-C(1A)	-177.85(17)
C(12A)-N(8)-C(7A)-C(1A)	0.25(18)
C(9)-N(8)-C(7A)-C(7)	-1.9(3)
C(12A)-N(8)-C(7A)-C(7)	176.16(17)
C(1A)-C(7A)-C(7)-C(13)	-121.5(2)
N(8)-C(7A)-C(7)-C(13)	63.4(2)
C(1A)-C(7A)-C(7)-C(6A)	3.0(2)
N(8)-C(7A)-C(7)-C(6A)	-171.98(17)
C(6)-C(6A)-C(7)-C(7A)	177.48(15)
C(2A)-C(6A)-C(7)-C(7A)	-5.1(2)
C(6)-C(6A)-C(7)-C(13)	-56.2(2)
C(2A)-C(6A)-C(7)-C(13)	121.22(18)
C(7A)-N(8)-C(9)-C(10)	178.63(17)
C(12A)-N(8)-C(9)-C(10)	0.8(3)
N(8)-C(9)-C(10)-C(11)	0.2(3)
C(9)-C(10)-C(11)-C(12)	-0.6(3)
C(1A)-N(1)-C(12A)-N(8)	-0.36(19)
C(1A)-N(1)-C(12A)-C(12)	179.20(19)
C(9)-N(8)-C(12A)-N(1)	178.35(16)
C(7A)-N(8)-C(12A)-N(1)	0.1(2)
C(9)-N(8)-C(12A)-C(12)	-1.3(3)
C(7A)-N(8)-C(12A)-C(12)	-179.55(16)
C(10)-C(11)-C(12)-C(12A)	0.1(3)
N(1)-C(12A)-C(12)-C(11)	-178.73(19)
N(8)-C(12A)-C(12)-C(11)	0.8(3)
C(16)-N(9)-C(13)-C(14)	-0.6(2)
C(17)-N(9)-C(13)-C(14)	179.67(18)
C(16)-N(9)-C(13)-C(7)	177.92(16)
C(17)-N(9)-C(13)-C(7)	-1.8(3)
C(7A)-C(7)-C(13)-C(14)	-112.0(2)
C(6A)-C(7)-C(13)-C(14)	126.6(2)
C(7A)-C(7)-C(13)-N(9)	69.8(2)
C(6A)-C(7)-C(13)-N(9)	-51.6(2)
N(9)-C(13)-C(14)-C(15)	0.3(2)
C(7)-C(13)-C(14)-C(15)	-178.18(17)
C(13)-C(14)-C(15)-C(16)	0.2(2)
C(14)-C(15)-C(16)-N(9)	-0.6(2)
C(13)-N(9)-C(16)-C(15)	0.7(2)
C(17)-N(9)-C(16)-C(15)	-179.53(17)

References

1. Proença, M. F.; Costa, M. *Tetrahedron* **2010,** *66*, 4542–4550. doi:10.1016/j.tet.2010.04.059
2. Eloy, F.; Deryckere, A. *Bull. Soc. Chim. Belg.* **1970,** *79*, 301–312. doi:10.1002/bscb.19700790505
3. Voskressensky, L. G.; Storozhenko, O. A.; Festa, A. A.; Khrustalev, V. N.; Dang, T. T. A.; Nhuyen, V. T.; Varlamov, A. V. *Tetrahedron Lett.* **2015,** *56*, 6475–6477. doi:10.1016/j.tetlet.2015.10.003
